# Incidence, prevalence, and global burden of attention-deficit/hyperactivity disorder from 1990 to 2021 across 204 countries in individuals under age 20: data, with critical appraisal, from the 2021 Global Burden of Disease study

**DOI:** 10.1038/s41380-026-03683-4

**Published:** 2026-06-16

**Authors:** Samuele Cortese, Min Seo Kim, Jong Hoon Han, Sarah Soyeon Oh, Dong Keon Yon, Jae II Shin, Marco Solmi

**Affiliations:** 1 Centre for Innovation in Mental Health, School of Psychology, Faculty of Environmental and Life Sciences, University of Southampton, Southampton, UK; 2 Hampshire and Isle of Wight Healthcare NHS Foundation Trust, Southampton, UK; 3 Clinical and Experimental Sciences (CNS and Psychiatry), Faculty of Medicine, University of Southampton, Southampton, UK; 4 Hassenfeld Children’s Hospital at NYU Langone, New York University Child Study Center, New York, NY USA; 5 DiMePRe-J-Department of Precision and Regenerative Medicine-Jonic Area, University of Bari “Aldo Moro”, Bari, Italy; 6 Broad Institute of MIT and Harvard, Cambridge, MA USA; 7 Yonsei University College of Medicine, Seoul, Republic of Korea; 8 Institute for Global Engagement & Empowerment, Yonsei University, Seoul, Republic of Korea; 9 Department of Pediatrics, Kyung Hee University Medical Center, Kyung Hee University College of Medicine, Seoul, Republic of Korea; 10 Department of Pediatrics, Yonsei University College of Medicine, Seoul, Republic of Korea; 11 Severance Underwood Meta-Research Center, Institute of Convergence Science, Yonsei University, Seoul, Republic of Korea; 12 Department of Psychiatry, University of Ottawa, Ottawa, ON Canada; 13 Regional Centre for the Treatment of Eating Disorders and on Track, The Champlain First Episode Psychosis Program, Department of Mental Health, The Ottawa Hospital, Ottawa, ON Canada; 14 Ottawa Hospital Research Institute (OHRI), Ottawa, ON Canada; 15 Department of Child and Adolescent Psychiatry, Charité Universitätsmedizin, Berlin, Germany

**Keywords:** ADHD, Psychiatric disorders

## Abstract

Attention-deficit/hyperactivity disorder (ADHD) is a common neurodevelopmental condition in  children and young people worldwide. Robust estimates of its incidence, prevalence, and burden are essential for informing public health policy and planning. Using data from the Global Burden of Disease Study 2021 (GBD 2021), this global population-based analysis assessed ADHD among individuals under 20 years of age across 204 countries and territories from 1990 to 2021. The study examined incidence, prevalence, and disability-adjusted life years (DALYs) associated with ADHD. In 2021, there were an estimated 46,890,733 (95% uncertainty interval [UI]: 32,136,904–67,271,064) prevalent cases and 4,111,621 (2,775,203–5,954,941) incident cases globally in individuals under 20 years. ADHD accounted for 574,979 (294,277–977,557) DALYs, with a global prevalence rate of 1.78% (1.22–2.55%) and an incidence rate of 0.16% (0.11–0.23%). The global DALY rate was 21.8 (11.2–37.1) per 100,000 population. Prevalence and incidence were highest in Australia, with rates of 5.62% (4.16–7.46%) and 0.49% (0.34–0.66%), respectively. Between 1990 and 2021, global prevalence and incidence rates decreased modestly by 6.0 and 5.81%, respectively. Across all GBD regions, prevalence was higher in males than females (2.52 vs 0.99%) and increased with higher socio-demographic index levels. Overall, the GBD 2021 study provides the most comprehensive global estimates of ADHD burden in young people. These findings are important for guiding policymakers and stakeholders, although potential methodological limitations suggest that the prevalence, incidence, and burden of ADHD may be underestimated.

## Introduction

Attention-deficit/hyperactivity disorder (ADHD), defined by developmentally inappropriate, persistent, and impairing inattention and/or hyperactivity impulsivity [1], is the most common neurodevelopmental disorder. ADHD is frequently associated with other neuropsychiatric disorders, such as neurodevelopmental, conduct, anxiety, and mood disorders, as well as physical conditions, such as obesity or asthma [2–4], which adds to the burden of ADHD. Estimating the prevalence and societal impact of ADHD has important implications in terms of public health preventive strategies. The most recent estimates of the global prevalence, incidence, and burden of ADHD were reported based on the Global Burden of Disease Study (GBD) 2019 [5, 6].

GBD estimates disease burden by summing up the estimated years of life lived with a disability (YLDs), as a measure of nonfatal burden, and the years of life lost (YLLs), related to mortality. The sum of YLDs and YLLs is referred to as disability-adjusted life years (DALYs), with one DALY equalling the loss of one healthy year of life. These estimates for ADHD have been reported across the lifespan. Updated data on the global prevalence, incidence, and burden of ADHD specifically in children and young people are lacking. The present study aimed to fill this gap by reporting the prevalence, incidence, and burden of ADHD in children and youths under 20 years of age relying on data provided by the latest edition of the GBD, namely GBD 2021.

## Methods

### Data source

This manuscript was produced as part of the GBD Collaborator Network and in accordance with the GBD Protocol. The GBD 2021 results are available on the Global Health Data Exchange (GHDx) website provided by the Institute for Health Metrics and Evaluation (http://ghdx.healthdata.org). The results of GBD 2021 include estimates for the prevalence, incidence, mortality, YLLs, YLDs, and DALYs related to diseases and injuries in 204 countries and territories. Details on the methodology are provided in the eMethods in Supplement 1 and previous studies, e.g., [7]. We followed the Guidelines for Accurate and Transparent Health Estimates Reporting (GATHER) statement (eTable 1 in Supplement 1).

The inputted data for the estimation model were gathered through a systematic literature review of studies on the prevalence of ADHD, conducted in three stages: electronic searches of peer-reviewed literature (via PsycInfo, Embase, and PubMed/MEDLINE), of grey literature, and expert consultation. In GBD 2021, the electronic search database was not updated from the one used in GBD 2019. Studies with an acceptable case definition and a study population representative of the general population were included. Acceptable definitions were those based on the diagnostic criteria for ADHD according to the Diagnostic and Statistical Manual of Mental Disorders (DSM) (II onwards) or the International Classification of Diseases (ICD) (9 onwards), including “hyperkinetic disorder” in the International Classification of Diseases (ICD)-10 [8]. Studies based on questionnaires with children or their parents, followed or not by structured or semi-structured interviews for diagnosis, were included. Methods used in the systematic review can be found elsewhere in more detail [7].

### Measures

Raw measures and age-standardized estimates of incidence, prevalence, and DALYs of ADHD were extracted with 95% uncertainty intervals (UI). In GBD 2021, mortality related to ADHD was not included in the calculations and YLLs, a metric indicating premature death, was thus assumed to be zero, as increased mortality was not assumed to be directly caused by ADHD. YLDs were calculated by multiplying the incidence of the condition, its specific disability weight, and the average duration of the condition, to represent the burden ADHD places on quality of life. DALYs are defined as the sum of YLLs and YLDs and are equal to the YLDs for ADHD.

### Statistical analysis

There were no significant changes in the modeling strategy from GBD 2019 and, similar to GBD 2019, no cross-walking for biases was conducted in GBD 2021. Estimates for each location, year, age group, and sex were generated using the DisMod-MR 2.1 model, a Bayesian meta-regression tool developed for the Global Burden of Disease study. Further details and formulation of DisMod-MR 2.1 can be found in the eMethods in Supplement 1 and previous studies.

Based on expert consultation and current literature, it was assumed that there was no incidence of ADHD before the age of 3 years. Additionally, a maximum age of onset of 12 years was established, following the latest Diagnostic and Statistical Manual of Mental Disorders (DSM)-5 criteria, and remission was set to zero prior to this age to align with these restrictions.

The disability weight for ADHD was determined through the GBD disability weight survey assessments. Severity was classified based on the proportion of time ADHD patients spent symptomatic versus asymptomatic, which was derived from the results of the Great Smoky Mountains Study [9]. The detailed methodology regarding severity split can be found elsewhere, e.g. [10].

As for GBD 2019, the 204 countries and territories were categorized into five subgroups based on the Socio-demographic Index (SDI) for GBD 2021. The SDI was calculated as a geometric mean of three parameters, each converted to a 0 to 1 index: the total fertility rate for those under 25 years old (TFU25), mean education for individuals aged 15 and older (EDU15+), and lag-distributed income (LDI) per capita. Analyses were completed with R software (version 3.5.0; R Foundation, Vienna, Austria).

## Results

### Global burden and temporal trend in ADHD in individuals under the age of 20

**Fig. 1:**
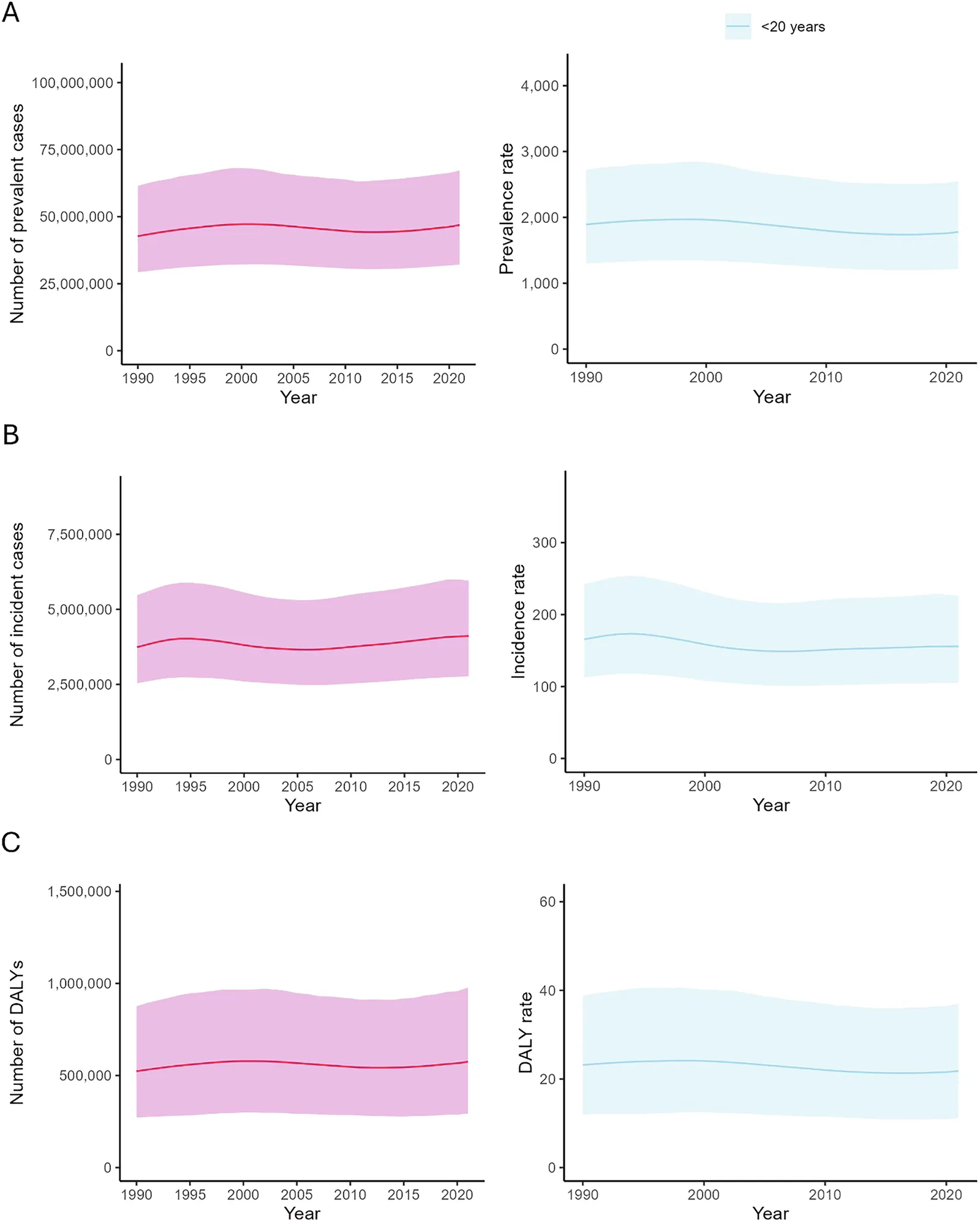
Global burden of ADHD in ages under 20 by year, 1990–2021. **A** Prevalence. **B** Incidence. **C** DALYs. DALYs indicate disability-adjusted life-years.

Globally, in 2021, there were a total of 46,890,733 (95% uncertainty interval [UI] 32,136,904 to 67,271,064) prevalent cases and 4,111,621 (95% UI 2,775,203 to 5,954,941) incident cases of ADHD in individuals under 20 (Fig. 1, Table 1, eTable 3a). A total of 574,979 (95% UI 294,277 to 977,557) DALYs were caused by ADHD 20 (Fig. 1, eTable 3b). The prevalence and incidence rates of ADHD were 1.78% (95% UI 1.22 to 2.55) and 0.16% (95% UI 0.11 to 0.23), respectively (Fig. 1, Table 1, eTable 3a). The DALY rate of ADHD was 21.8 (95% UI 11.2 to 37.1) per 100,000 population (Fig. 1, eTable 3b).

**Table 1:** Prevalence of ADHD in ages under 20 by region, 1990–2021.

	Prevalence of attention-deficit/hyperactivity disorder					
	Counts (<20 years)			Rate per 100,000 persons (<20 years)		
Region	1990 (95% UI)	2021 (95% UI)	Percentage change from 1990 to 2021 (%)	1990 (95% UI)	2021 (95% UI)	Percentage change from 1990 to 2021 (%)
Global	42742759 (29295029 to 61474615)	46890733 (32136904 to 67271064)	9.7 (6.81 to 12.68)	1892 (1297 to 2722)	1779 (1219 to 2552)	−6 (−8.48 to −3.45)
High-income Asia Pacific	1197468 (808502 to 1723217)	719705 (494654 to 1039400)	−39.9 (−42.1 to −37.04)	2379 (1607 to 3424)	2338 (1607 to 3376)	−1.76 (−5.37 to 2.91)
High-income North America	2626253 (1771092 to 3797232)	3248386 (2154251 to 4639606)	23.69 (11.75 to 33.92)	3213 (2167 to 4646)	3627 (2405 to 5181)	12.88 (1.98 to 22.22)
Western Europe	2378130 (1657164 to 3324169)	2320982 (1560149 to 3287210)	−2.4 (−8.85 to 3.25)	2418 (1685 to 3380)	2531 (1701 to 3584)	4.66 (−2.25 to 10.72)
Australasia	322192 (224879 to 449822)	404699 (296904 to 539136)	25.61 (10.25 to 46.83)	5136 (3585 to 7171)	5366 (3937 to 7149)	4.48 (−8.3 to 22.13)
Andean Latin America	622383 (420248 to 900864)	804774 (543647 to 1164003)	29.31 (28.8 to 29.74)	3283 (2217 to 4752)	3399 (2296 to 4917)	3.54 (3.13 to 3.89)
Tropical Latin America	2334728 (1605921 to 3437608)	2227234 (1520258 to 3221298)	−4.6 (−13.06 to 5.14)	3371 (2319 to 4963)	3345 (2283 to 4838)	−0.77 (−9.56 to 9.36)
Central Latin America	1841484 (1253201 to 2650234)	2015032 (1374784 to 2898430)	9.42 (6.47 to 12.4)	2229 (1517 to 3207)	2363 (1612 to 3399)	6.02 (3.16 to 8.9)
Southern Latin America	416672 (278877 to 612637)	442487 (299173 to 642188)	6.2 (−4.64 to 16.2)	2150 (1439 to 3161)	2268 (1533 to 3292)	5.49 (−5.28 to 15.43)
Caribbean	699005 (487690 to 987469)	731015 (504345 to 1043717)	4.58 (2.06 to 6.43)	4629 (3230 to 6540)	4790 (3304 to 6838)	3.46 (0.96 to 5.29)
Central Europe	658129 (448634 to 948066)	394499 (268968 to 568460)	−40.06 (−40.12 to −39.99)	1676 (1142 to 2414)	1675 (1142 to 2413)	−0.08 (−0.19 to 0.03)
Eastern Europe	1111138 (742566 to 1615665)	801572 (534701 to 1164564)	−27.86 (−28.07 to −27.6)	1652 (1104 to 2402)	1737 (1158 to 2523)	5.14 (4.83 to 5.52)
Central Asia	474990 (322822 to 695555)	533501 (362199 to 781863)	12.32 (12.07 to 12.62)	1504 (1022 to 2203)	1541 (1046 to 2258)	2.44 (2.21 to 2.72)
North Africa and Middle East	3453559 (2383286 to 4967004)	4394111 (3031049 to 6359998)	27.23 (21.95 to 32.82)	1954 (1348 to 2810)	1858 (1282 to 2689)	−4.9 (−8.84 to −0.72)
South Asia	4730362 (3123142 to 6918362)	6406755 (4252521 to 9255394)	35.44 (25.05 to 45.73)	872 (576 to 1275)	937 (622 to 1354)	7.49 (−0.76 to 15.65)
Southeast Asia	3400825 (2321988 to 4859740)	3427714 (2344277 to 4943959)	0.79 (−4.53 to 3.6)	1547 (1056 to 2210)	1495 (1022 to 2156)	−3.33 (−8.43 to −0.64)
East Asia	14316211 (9908736 to 20582252)	13018254 (9117581 to 18633515)	−9.07 (−16.38 to −1.41)	3111 (2153 to 4473)	3774 (2643 to 5402)	21.3 (11.54 to 31.51)
Oceania	57188 (39398 to 83519)	108036 (74443 to 157839)	88.92 (88.79 to 89.04)	1699 (1170 to 2481)	1692 (1166 to 2471)	−0.42 (−0.49 to −0.35)
Western Sub-Saharan Africa	823000 (547750 to 1239291)	2215071 (1473274 to 3266019)	169.15 (154.26 to 183.55)	766 (510 to 1153)	825 (549 to 1216)	7.72 (1.77 to 13.49)
Eastern Sub-Saharan Africa	838331 (558812 to 1235096)	1832457 (1224936 to 2689085)	118.58 (117.31 to 119.69)	756 (504 to 1114)	805 (538 to 1182)	6.51 (5.89 to 7.05)
Central Sub-Saharan Africa	229546 (153911 to 338148)	584196 (391757 to 860482)	154.5 (153.97 to 154.87)	741 (497 to 1091)	794 (533 to 1170)	7.21 (6.99 to 7.37)
Southern Sub-Saharan Africa	211166 (140903 to 312415)	260254 (173740 to 384849)	23.25 (22.83 to 23.7)	798 (533 to 1181)	832 (556 to 1231)	4.31 (3.96 to 4.7)

*UI* uncertainty interval.

While the number of global prevalent cases in individuals under 20 increased by 9.7% (95% UI 6.81 to 12.68) between 1990 and 2021, the prevalence rate decreased by −6% (95% UI −8.48 to −3.45) in the same period (Fig. 1, Table 1), due to change in the size of the total population. Likewise, there was a 9.92% (95% UI 7.13 to 13.16) increase in the number of incident cases while there was a −5.81% (95% UI −8.21 to −3.04) decrease in the incidence rate (Fig. 1, eTable 3a). Similarly, raw DALYs increased by 9.85% (95% UI 6.84 to 12.86) and DALY rate decreased by −5.88% (95% UI −8.45 to −3.29) (Fig. 1, eTable 3b).

### Variation at national and regional level

**Fig. 2:**
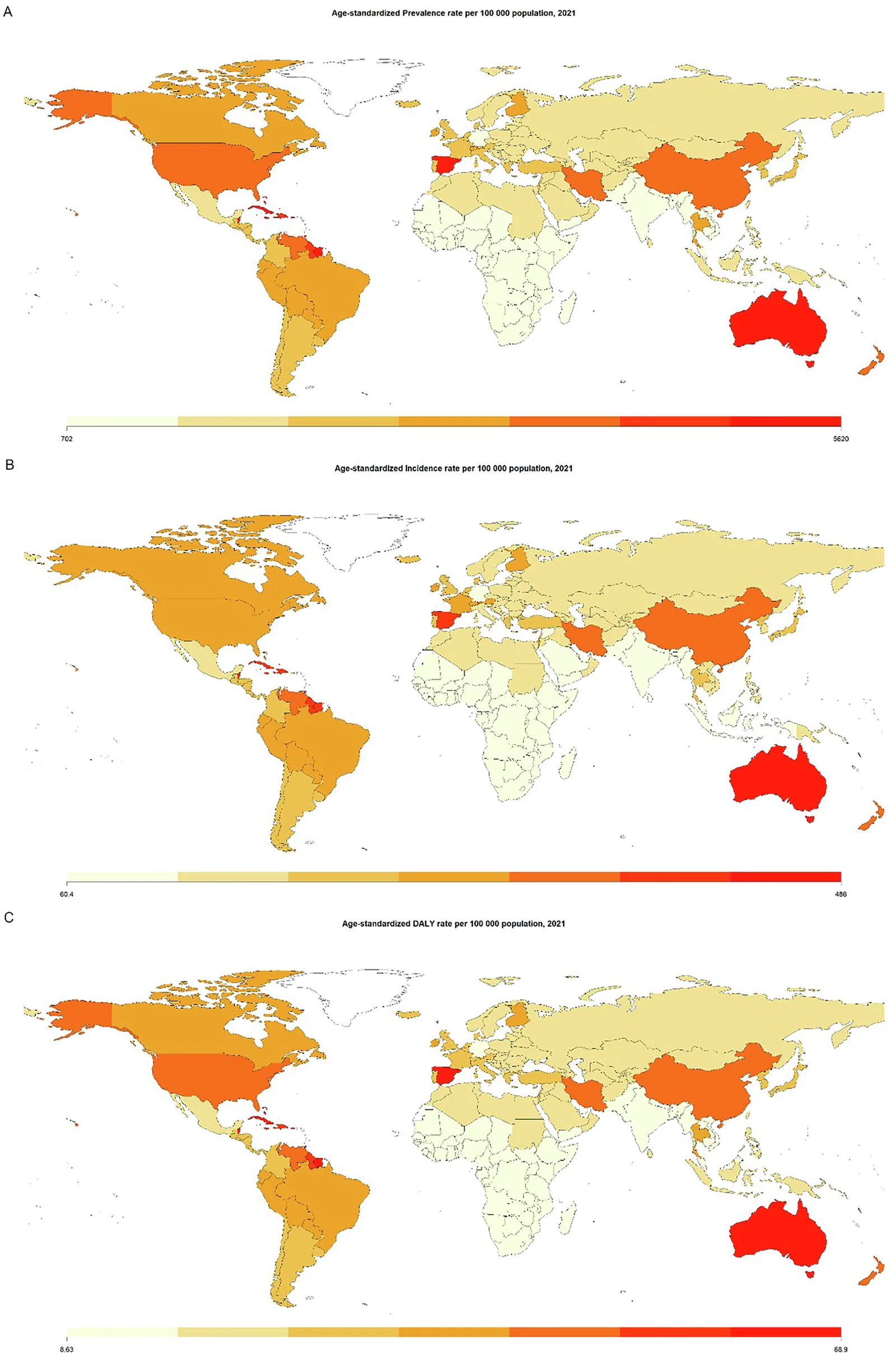
Geographical distribution of age-standardized prevalence, incidence and DALYs rate of ADHD, 2021. **A** Age-standardized prevalence rate per 100000 population, 2021. **B** Age-standardized incidence rate per 100000 population, 2021. **C** Age-standardized DALY rate per 100000 population, 2021.

**Fig. 3:**
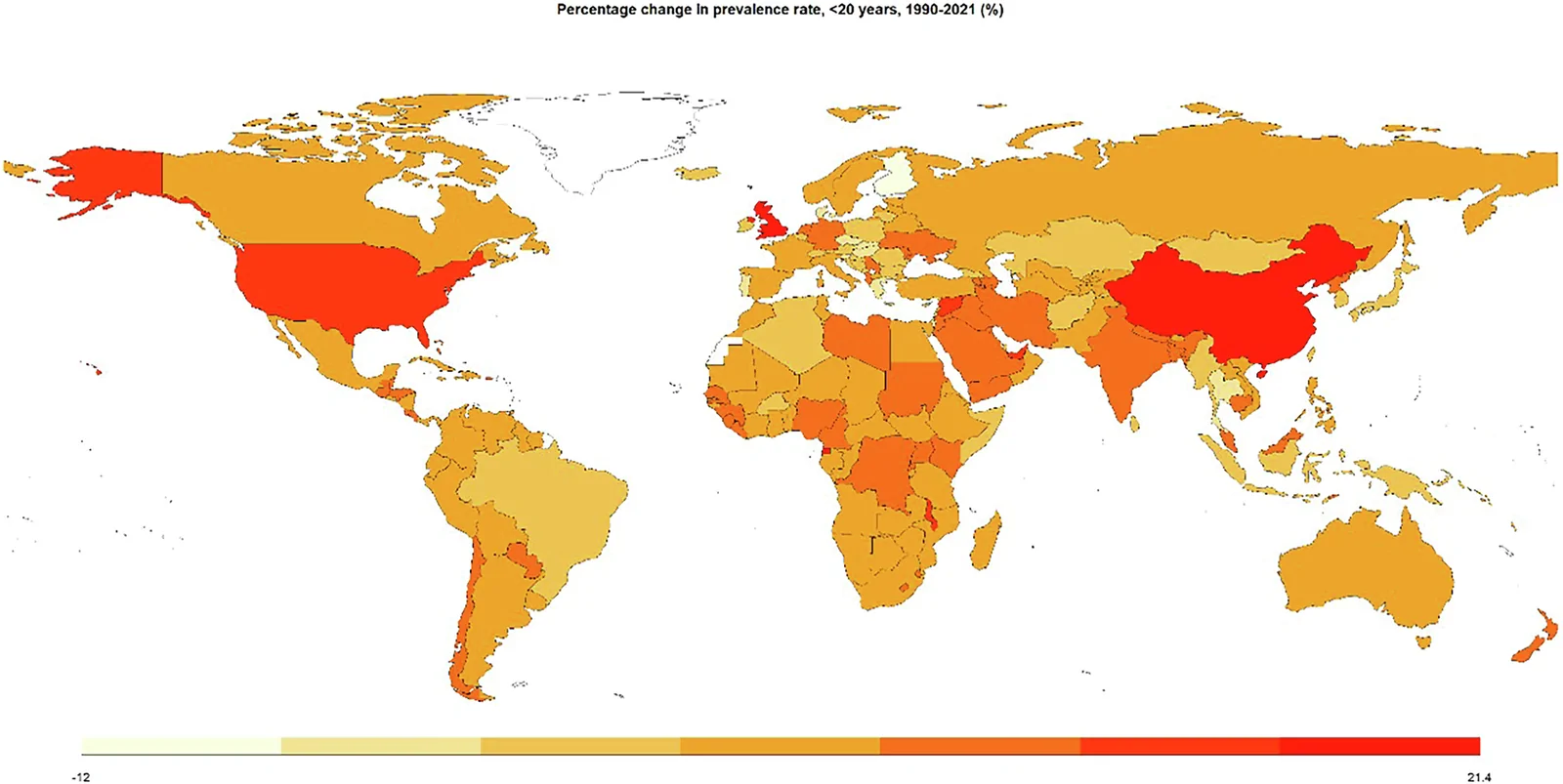
Geographical variation of percentage change in ADHD prevalence rate in under 20, 1990-2021. .

At the nation level, prevalence and incidence of ADHD in individuals under 20 were the highest in Australia at 5.62% (95% UI 4.16 to 7.46) and 0.49% (95% UI 0.34 to 0.66), respectively (Fig. 2, eTable 4a, 4b). The largest increase in prevalence of ADHD in individuals under 20 between 1990 and 2021 was observed in China (21.44%, 95% UI 11.42 to 31.85). The largest decrease was observed in Finland (−12%, 95% UI −38.49 to 9.34) (Fig. 3, eTable 4a). The largest increase in incidence occurred in China (41.46%, 95% UI 30.08 to 54.22) and the largest decrease in incidence occurred in Syrian Arab Republic −27.29% (95% UI −27.32 to −27.25) (eTable 4b).

Across GBD regions, the highest prevalence (5.37%, 95% UI 3.94 to 7.15), incidence (0.46%, 95% UI 0.32 to 0.64), and DALY rate (65.8 per 100,000 population, 95% UI 35.1 to 104.4) of ADHD in individuals under 20 were observed in Australasia. On all three measures, the Caribbean and East Asia followed (Table 1, eTable 3a, 3b, 3c, eFigure 3a, 3b, 3c). The largest increase in prevalence (21.3%, 95% UI 11.54 to 31.51), incidence (40.62%, 95% UI 29.55 to 53.05), and DALY rate (21.61%, 95% UI 12.09 to 32.76) were observed in East Asia. Meanwhile, significant decrease in prevalence was observed in North Africa and Middle East and Southeast Asia. The largest decrease in incidence occurred in South Asia (−11.56%, 95% UI −18.42 to −5.05) (Table 1, eTable 7a, 7b, 7c, eFigure 4a, 4b, 4c). The number of prevalent cases and prevalence rate of ADHD under the age of 20 by GBD region for each year from 1990 to 2021 are reported in eTable 5a.

Within the five SDI subgroups, the prevalence of ADHD was higher with increasing SDI, reaching as high as 2.88% (95% UI 1.94 to 4.09) in the high SDI subgroup (eTable 6a, eFigure 5). Meanwhile, the incidence was highest in the high-middle SDI group, showing a 20.53% (95% UI 14.61 to 27.36) increase compared to 1990 (eTable 6b, eFigure 5).

### Variation by sex and age

In 2021, the prevalence of ADHD in males under the age of 20 was 2.52% (95% UI 1.72 to 3.62), which was significantly higher than the prevalence in females, 0.99% (95% UI 0.68 to 1.44). Prevalence was higher in males than in females in all GBD regions (eTable 7a).

**Fig. 4:**
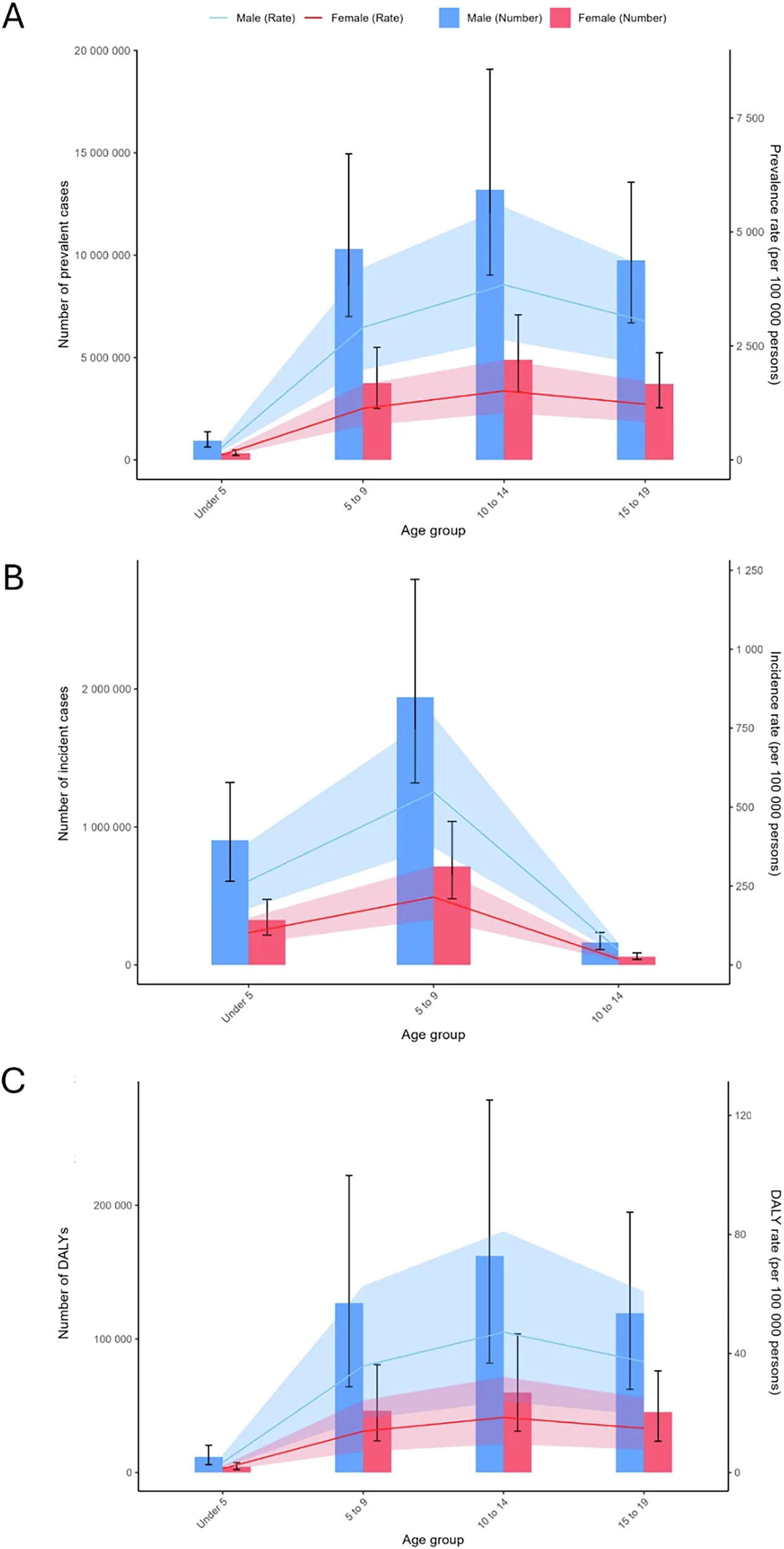
Global burden of ADHD by age group and sex, 2021. **A** Prevalence. **B** Incidence.  **C** DALYs. DALYs indicate disability-weighted life-years.

For both males and females, the prevalence and DALY rate of ADHD peaked at 10–14 years of age and the incidence of ADHD was highest at 5–9 years of age (Fig. 4). Among the age groups, the decline in ADHD prevalence from 1990 to 2021 was larger with increasing age. While the percentage change in prevalence was −2.26% (95% UI −4.88 to 0.69) in ages under 5, there was a decrease of −14.97% (95% UI −17.39 to −12.55) in ages 15–19. Similarly, the incidence of ADHD decreased at a higher rate with increasing age, reaching −7.57% (95% UI −9.75 to −4.92) in ages 15–19.

## Discussion

To our knowledge, this is the most comprehensive report on the prevalence, incidence, and burden of ADHD in children and young people in the past 30 years, based on data from the GBD 2021. We estimated that approximately 47 million children and young people under 20 years of age were living with ADHD worldwide in 2021, corresponding to an overall prevalence of 1.78%. These estimates reflect model-based population prevalence derived primarily from population-representative epidemiological studies applying standardized DSM or ICD diagnostic criteria, and represent the overall population burden of ADHD rather than administrative counts of diagnosed cases.

However, the estimated prevalence is considerably lower than the prevalence estimated in previous meta-analyses, such as the ~7% prevalence reported in the meta-analysis by Thomas et al. [11]. However, that meta-analysis pooled data exclusively from studies relying on a diagnosis of ADHD based on the DSM. In contrast, data from the GBD include also estimates from studies based on the ICD-10 (or earlier versions), which includes “Hyperkinetic Disorder” rather than ADHD. Hyperkinetic disorder refers to a more restricted and severe group of individuals compared to those defined by ADHD according to the DSM. As such, the prevalence of hyperkinetic disorder is expected to be lower than that of ADHD. Furthermore, that meta-analysis incorporated studies that drew samples from school populations, with no input from clinicians, and did not involve interviews. These factors could have contributed to discrepancies when compared to the GBD estimates.

However, earlier meta-analyses, including studies based on both DSM and ICD definitions, have consistently reported higher prevalence estimates, such as around 5% in the meta-analysis by Polanczyk et al. [12]. Notably, unlike previous meta-analyses, the GBD study imputes missing data for countries lacking prevalence data. A re-analysis of the GBD 2019 data, focusing only on directly estimated data (rather than pooling both actual and imputed data), suggested that the global prevalence of ADHD may be underestimated, largely due to the GBD group’s imputation methods [5]. While other researchers have endorsed this interpretation [13], further studies are needed to confirm it. However, this potential issue does not affect the comparative estimation of differences across countries, which is a key opportunity offered by the GBD.

Recent findings indicate an increase in administrative prevalence (i.e., that based on data from diagnostic codes) of ADHD, along with higher prescription rates for ADHD medications during the pandemic [14]. Furthermore, it has been speculated that the residual effects of COVID-19 might selectively affect brain regions associated with attention and motivation deficits, potentially increasing the risk for an infection-triggered etiological subtype of ADHD [15]. However, the GBD 2021 is not suitable for testing this hypothesis, as it extrapolates 2020 and 2021 data without incorporating new studies beyond those included in the GBD 2019, which relied on research conducted up to 2017.

While the administrative prevalence, reflecting the actual diagnoses made in clinical services, has increased over the past decades, possibly due to greater awareness and recognition of the disorder, the GBD 2021 showed that the actual prevalence of the disorder, as diagnosed using standardized tools in the general population, has not increased (if anything, it has slightly decreased). This is consistent with the findings of a previous meta-regression analysis by Polanczyk et al., [16] which showed that, across 154 original studies, the year of the study was not associated with variability in ADHD prevalence estimates.

We did find differences in the prevalence and incidence of ADHD across countries, with the highest prevalence and incidence observed in Australia. The etiology of ADHD is complex and multifactorial, involving a range of genetic and environmental factors that interact in ways specific to each individual. However, we are not aware of any solid evidence demonstrating meaningful differences in genetic and environmental factors between populations. The hypothesis suggesting a role for the level of different light exposure across different countries —which may contribute to explain differences across countries— has been criticized [17] and requires further evidence. Rather, variation in the prevalence of ADHD across countries may be influenced by differing perceptions of impairment [18] associated with the disorder, which is a necessary criterion for diagnosis and is likely shaped by cultural factors. This may also explain another finding of this study—namely, that countries with a high SDI tend to have higher prevalence rates of ADHD. This could be due to a lower threshold for perceiving impairment caused by ADHD symptoms in these settings.

Furthermore, access to child psychiatric care and diagnostic services vary across regions and may have influenced detection rates. In many settings, mental health services for children and young people remain unavailable, underfunded, and/or of inadequate quality [19]. Access to child psychiatric care is particularly constrained in Africa, where child and adolescent mental health receives less than 2% of national health expenditure, and severe workforce shortages persist, with several countries reporting fewer than 0.1 child psychiatrics per 100,000 children [20]. In the Middle East and North African (MENA) region, child psychiatrics, developmental-behavioural paediatricians, and educational psychologists are particularly scarce [21], especially outside major urban areas [19]. These shortages may be compounded by weak referral systems and limited integration of mental health services into primary care, reducing opportunities for early identification [20]. Even where services exist, long waiting lists for specialist appointments pose significant barriers, with some families experiencing delays of several months before obtaining assessments, thereby postponing critical interventions during key developmental periods [19]. Geographic and socioeconomic disparities create additional barriers to access to child psychiatric care, as specialized centres for developmental and behavioural evaluations may be limited or entirely absent rural and low-income areas [19]. Infrastructural challenges, including transportation barriers and inconsistent digital connectivity further restrict access to child psychiatric care and hinder the expansion of more cost-effective, telemedicine-based solutions designed to reach underserved populations. Given these persistent barriers to child psychiatric care access, we encourage further studies to collect direct data on the prevalence and incidence of ADHD, particularly in low- and middle-income countries, where epidemiological data have traditionally been limited.

We also found that the prevalence and incidence of ADHD were substantially higher in males compared to females across all GBD regions. This finding contributes to our understanding of sex differences in the presentation of ADHD. Previous evidence suggests that males, particularly during childhood and adolescence, are overrepresented in clinical services [22]. This is likely because their more disruptive behaviors lead to earlier and more frequent referrals compared to females. However, since the GBD study utilized population-based representative samples, our findings indicate a genuine difference in ADHD prevalence and incidence in non-clinical populations, consistent with other population-based research [23]. Cultural expectations may influence how ADHD symptoms are reported, especially in certain geographic regions. Women may be less likely to report their symptoms or may perceive them as less disabling compared to men, which could result in not meeting diagnostic criteria. Interestingly, a recent meta-analysis found no significant sex differences in symptom severity when assessments were conducted through clinical interviews, as opposed to questionnaires in children [23]. The reasons for the discrepancy between findings from rating scales and clinical diagnostic interviews remain unclear. However, these findings suggest that the GBD 2021 results on sex differences in ADHD prevalence and incidence might have been different if the GBD study had relied solely on studies using semi-structured interviews.

The estimate of the burden associated with ADHD been criticised [5]. First, phrasing of the survey questions used to derive the weights may have overly focused on the core symptoms of ADHD, thereby minimizing the importance of other dimensions, such as emotional dysregulation, which affect a significant portion of individuals with ADHD. Second, the breakdown of symptom severity, based on the proportion of time spent symptomatic versus asymptomatic, relied on a study conducted over 20 years ago. Recent evidence indicates that ADHD symptoms tend to fluctuate, suggesting that remission may often be temporary [24, 25]. Even when symptoms fall below the diagnostic threshold, considerable impairment frequently persists. Third, similar to the GBD 2019, the GBD 2021 set mortality at zero for ADHD, as mortality in the calculation of DALYs related to mental conditions was only included for eating disorders—on the grounds that these are directly linked to mortality. However, setting mortality to zero is questionable. Meta-analytic evidence [26] has shown increased mortality rates associated with ADHD, with findings indicating that pharmacological treatment for ADHD reduces the risk of mortality. Furthermore, one could argue that mortality in eating disorders is mediated by physical events, much like in ADHD, where deaths due to suicide or unintentional injuries follow a similar pattern. While the increased risk of mortality from unnatural causes, such as accidents, is particularly relevant for adults, it may also affect young adults, as considered in this analysis. Therefore, we conclude that the burden of ADHD in children and young people was likely underestimated by the GBD 2021.

Despite the potential underestimation of ADHD prevalence due to imputation methods, we believe that standardized methodology applied across countries and time provide invaluable comparability for policy assessment. The creators of the GBD have been transparent about prioritizing comparability over absolute accuracy; as Murray notes, “From the beginning of the GBD, we have seen the value of emphasizing comparability over time and across place.” [27] This commitment to comparability is reflected in the GBD’s approach of recomputing the entire historical time series for each disease with every release, and for decision-makers that may rely on such results to understand the relative burdens and temporal trends for their respective communities, understanding these estimates may be crucial for resource allocation and intervention prioritization where more comparable data across countries and territories are unavailable [28].

Finally, some countries showed wide uncertainty intervals that cross the zero threshold, indicating insufficient evidence to definitively establish the direction of prevalence change. For instance, Finland displayed a UI of −38.49 to 9.34, which likely reflects genuine temporal variation in ADHD detection and diagnostic practices rather than limited data. Finland showed the largest decrease in incidence, prevalence, and burden from 1990 to 2019 in our previous analysis of GBD 2019 data [5]. Recent nationwide studies from Finland have documented considerable volatility in ADHD diagnostic patterns, with new ADHD diagnoses doubling during the pandemic period (2020–2022) and lifetime prevalence increasing from 1.80% in 2020 to 2.76% in 2022 [29]. Additionally, diagnostic practices in Finland have shifted considerably over recent decades, with ADHD diagnostics moving largely from specialized healthcare to schools and primary care units [29]. These documented temporal fluctuations in detection rates contribute to wider uncertainty bounds in modeling estimates, particularly as GBD 2021 relies on studies conducted up to 2017 and cannot capture more recent shifts. Countries with UIs crossing zero should be interpreted as having insufficient evidence to determine the direction of change with statistical confidence, highlighting the need for continued epidemiological surveillance in settings experiencing rapid changes in clinical practice and service delivery models.

The GBD 2021 has notable strengths. First, it relies on a comprehensive search of the literature. Second, like the GBD 2019, but unlike previous versions, GBD 2021 estimates for ADHD represent a substantial advance over earlier GBD estimates, with the inclusion of new data from 18 locations (Argentina, Australia, Austria, Brazil, China, Colombia, Cyprus, Finland, United Kingdom, India, Iran, Lebanon, Nigeria, Saudi Arabia, South Korea, Spain, Taiwan, and United States). Third, the GBD study relies on population-based representative samples, ensuring that the estimates are not limited to clinical populations.

However, the findings of this study should be interpreted with consideration of several limitations. First, although extrapolations were made for 2020 and 2021, the search methods and methodology for the GBD 2019 remained consistent with those used in the 2019 version. Second, the GBD’s definition of disability focuses on health outcomes but does not account for welfare loss, meaning it fails to capture the full psychosocial impact of mental disorders. Third, the estimated burden did not include the impact of comorbidities, which are common in ADHD rather than exceptional. The effects of comorbidities are difficult to separate from those of ADHD itself. Fourth, results characterized by large UIs, reflecting a paucity of data, should be interpreted with caution due to the uncertainty of the estimate.

Despite these limitations, this report represents the most comprehensive account of the prevalence, incidence, and burden of ADHD in children and young people. It should inform individuals with lived experience, clinicians, and policymakers globally.

## Supplementary information

APPENDIX

## Data Availability

All data downloaded from GBD database (https://www.healthdata.org/researchanalysis/gbd).
