# Supplementary material for: Incidence, prevalence, and global burden of attention-deficit/hyperactivity disorder from 1990 to 2021 across 204 countries in individuals under age 20: data, with critical appraisal, from the 2021 Global Burden of Disease study
Source: Mol Psychiatry. 2026 Jun 16;31(10):5931–40. doi: 10.1038/s41380-026-03683-4 (PMC13569453; doi:10.1038/s41380-026-03683-4)
Supplement: Supplementary file 1 [file 41380_2026_3683_MOESM1_ESM.docx]

**Supplemental Online Content for:**

**Incidence, prevalence, and global burden of attention-deficit/hyperactivity disorder from 1990 to 2021 across 204 countries in individuals under age 20: data, with critical appraisal, from the 2021 Global Burden of Disease study**

**eMethods**

**eReferences**

**eTable 1.** GATHER checklist of information that should be included in new reports of global health estimates.

**eTable 2:** Data inputs for ADHD morbidity modelling by parameter.

**eTable 3a**. Incidence of ADHD by region, <20 years, 1990-2021.

**eTable 3b**. DALYs caused by ADHD by region, <20 years, 1990-2021.

**eTable 4a**. Prevalence of ADHD by country, <20 years, 1990-2021.

**eTable 4b**. Incidence of ADHD by country, <20 years, 1990-2021.

**eTable 4c**. DALYs caused by ADHD by country, <20 years, 1990-2021.

**eTable 5a**. Prevalence of ADHD by region and year, <20 years, 1990-2021.

**eTable 5b**. Incidence of ADHD by region and year, <20 years, 1990-2021.

**eTable 5c**. DALYs caused by ADHD by region and year, <20 years, 1990-2021.

**eTable 6a**. Prevalence of ADHD by SDI group and year, <20 years, 1990-2021.

**eTable** **6b**. Incidence of ADHD by SDI group and year, <20 years, 1990-2021.

**eTable** **6c**. DALYs caused by ADHD by SDI group and year, <20 years, 1990-2021.

**eTable** **7a**. Prevalence of ADHD by region and sex, <20 years, 1990-2021.

**eTable** **7b**. Incidence of ADHD by region and sex, <20 years, 1990-2021.

**eTable** **7c**. DALYs caused by ADHD by region and sex, <20 years, 1990-2021.

**eFigure 1.** Flowchart demonstrating non-fatal estimation for ADHD.

**eFigure** **2**: Burden of ADHD by sex and year, <20 years, 1990-2021. (A) Prevalence. (B) Incidence. (B) DALYs.

**eFigure** **3a**: Prevalence rate of ADHD in ages under 20 by sex and region, 2021.

**eFigure** **3b**: Incidence rate of ADHD in ages under 20 by sex and region, 2021.

**eFigure** **3c**: DALY rate of ADHD in ages under 20 by sex and region, 2021.

**eFigure** **4a**: Percentage change in prevalence rate of ADHD in ages under 20 by region, 1990-2021.

**eFigure** **4b**: Percentage change in incidence rate of ADHD in ages under 20 by region, 1990-2021.

**eFigure** **4c**: Percentage change in DALY rate of ADHD in ages under 20 by region, 1990-2021.

**eFigure** **5**: (A) Prevalence, (B) incidence, and (C) DALY rate in ages under 20 of ADHD by SDI group and year, 1990-2021.

**eMethods**

This section provides a summary of the detailed methods used in the estimation of the burden of attention-deficit/hyperactivity disorder (ADHD) in the Global Burden of Disease, Injuries and Risk Factors study (GBD) 2021. Most of the information presented in this section is a reproduction of the methods demonstrated in the supplementary appendix of a GBD 2021 capstone paper “Global incidence, prevalence, years lived with disability (YLDs), disability-adjusted life-years (DALYs), and healthy life expectancy (HALE) for 371 diseases and injuries in 204 countries and territories and 811 subnational locations, 1990–2021: A systematic analysis for the Global Burden of Disease Study 2021”. [1] This study complies with the Guidelines for Accurate and Transparent Health Estimates Reporting (GATHER). eTable 1 shows the GATHER checklist for this study.

**Overview**

GBD study is an analysis of the burden of diseases, injuries, and risk factors stratified by age, sex, time period, and geographical locations across the world. It is being updated by The Institute for Health Metrics and Evaluation (IHME), an international network of researchers. In the latest update, GBD 2021, more than 11,000 IHME collaborators from more than 160 countries and territories participated in providing, reviewing, and analyzing data.

As in the previous GBD 2019, 204 countries and territories were included, grouped into 21 regions and 7 super-regions in GBD 2021. Some countries were analyzed at subnational levels, adding up to total 983 different geographic locations when divided at the most detailed level. Prevalence, incidence, years lived with disability (YLDs), and disability-adjusted life-years (DALYs) from 1990 to 2021 were estimated.

The list of non-fatal causes list increased from 364 causes in GBD 2019 to 365 causes in GBD 2021. The list of causes of death also increased from 286 causes in GBD 2019 to 288 causes in GBD 2021. In GBD 2021, the causes of death and non-fatal causes combined encompassed 371 causes. These causes were organized in a hierarchical manner, with Level 1 categories being the most general groupings and Level 3 or Level 4 categories containing individual causes. Under each cause, its sequelae were assigned at Level 5 and 6 categories of the hierarchy. The sequelae for each cause were mutually exclusive. As a result, total 3499 mutually exclusive and collectively exhaustive sequelae were incorporated.

The input data into the estimation model came from various sources including surveys, censuses, vital registries, verbal autopsies, and administration data. The input sources for specific GBD components, causes and risks, and locations can be accessed through the GBD Sources Tool in the Global Health Data Exchange (GHDx) website: <http://ghdx.healthdata.org/gbd-2021/sources>.

**Non-fatal estimation process**

Non-fatal estimation process was divided into 8 steps: (1) data compilation; (2) data adjustments; (3) estimation of prevalence and incidence by cause and sequelae; (4) estimation by impairment; (5) severity distributions; (6) incorporation of disability weights (DWs); (7) comorbidity adjustment; and (8) estimation of YLDs by sequelae and causes. Detailed estimation process varied between causes.

Data were collected from systematic reviews of the literature, surveys, disease registries, case notifications, administrative claims, inpatient hospital data, and outpatient data. Systematic reviews for 77 causes and risk factors, including ADHD, were conducted for GBD 2021. For causes and sequelae that lacks sufficient literature for conducting systemic reviews, other data sources predominated.

Then the data went under adjustment processes. The collected data was corrected by ‘crosswalking’ when the data had a known consistent bias in terms of case definition or study methods, compared with the standard GBD definition and study methods. A mixed effects meta-regression model was constructed to predict the difference between the two observations in log or logit space using several covariates. Data used to fit the model were compiled from both within-study comparisons and between-study comparisons. Covariates were chosen if there were systematic differences between the observations by key demographics (location, age, sex, year). Other factors were chosen if there was a strong rationale for variation by such characteristics. When there were more than one alternative case definitions or study methods, network analysis was conducted. In network analysis, a design matrix that contains comparisons between the datasets were passed to the mixed effects meta-regression model.

The model used in crosswalking process is MR-BRT (Meta-regression—Bayesian, regularised, trimmed), a modelling tool developed at IHME. MR-BRT is equipped with many features beyond the classical mixed effects meta regression model. The source code for MR-BRT is publicly uploaded on GitHub as the Python package mrtool (ihmeuw-msca , 2023).

Before modelling, a Dismod-MR 2.1 model was run to estimate some countries’ age patterns. Retrieved age patterns were applied to split aggregated all-age data.

Then, spatiotemporal Gaussian process regression (ST-GPR) modelling was performed to smooth data over age, time, and locations in locations with incomplete datasets. GPR model is a stochastic model which assumes that the variable of interest follows a Gaussian process, a process defined by a mean function and a covariance function. The mean function is determined as a sum of the components of a hierarchical mixed-effects linear regression and a smoothing function, a locally weighted polynomial regression that is fit using the residuals derived from the linear model, and lets the model borrow strength across time, age, and space patterns. The covariance function is defined as a Matern-Euclidian covariance function that incorporates distance function, marginal variance, smoothness parameter, and length scale. The Gaussian process was integrated over time to predict a full time series of the parameter. Further details on ST-GPR model can be found elsewhere. [2]

After the smoothing process, prevalence and incidence by cause and sequelae were estimated using DisMod-MR 2.1. DisMod-MR was first introduced in GBD 2010 as DisMod-MR 1.0 to produce estimates from multiple data sources by using Bayesian statistical methods and went through updates in subsequent iterations. DisMod-MR produces estimates at five levels: global, super-region, region, country, and subnational location. The upper-level estimates are combined with mixed-effects to generate lower-level estimates, forming a cascade of estimation. Detailed formulation of DisMod MR 1.0 can be found in the GBD 2010 capstone paper. [3] For GBD 2013, the improved DisMod-MR 2.0 was developed to have increased computational speed by switching to a model of log rates instead of a negative binomial model. [4] In GBD 2015, updated DisMod-MR 2.1 was employed so that the country covariates are re-estimated at each level of cascade. While DisMod-MR 2.1 was adopted for GBD 2019 and 2021 but there were changes in the way it was used. Random effects were applied on cause-specific mortality rates (CSMR) and excess mortality rate (EMR). The functionality in DisMod-MR 2.1 that estimates coefficients for study and sex covariates was no longer used due to the crosswalking step preceding estimation. Also, priors were passed down with a wider setting of minimum coefficient of variation since GBD 2019 demonstrated that such manipulation improved coverage and reduced errors.

Prevalence of nine impairments that result from GBD causes as sequelae were estimated for GBD 2021 by using DisMod-MR 2.1: anemia, epilepsy, hearing loss, heart failure, intellectual disability, infertility, vision loss, Guillain-Barré syndrome, and pelvic inflammatory disease.

Next, sequelae for 236 causes defined in terms of severity and the severity levels under each cause were distributed. For some causes, the distribution of severity levels was estimated using DisMod-MR 2.1 or ST-GPR. For other causes, severity levels were allocated by meta-analyses of the literature. For causes with insufficient data on severity found from the literature, three population surveys were used as references: the MEPS 2000–2014, the [US] National Epidemiological Survey on Alcohol and Related Conditions (NESARC) 2000–2001 and 2004–2005, and the Australian National Survey of Mental Health and Wellbeing of Adults (NSMHWB) 1997 (Medical Expenditure Panel Survey Home, n.d.) (Mental Health and Wellbeing: Profile of Adults, Australia, 1998) that report 12-Item Short Form Health Surveys (SF-12) filled in by individuals. SF-12 scores were converted to disability weights (DW) using the relationship between SF-12 score and disability weight established by a locally estimated scatterplot smoothing (LOESS) regression. The regression was based on the responses of a convenience sample of respondents in GBD DW surveys who were asked to fill in SF-12 for the hypothetical individual living in a health state described by GBD DW surveys. Then, models that predict disability weights of an individual suffering from multiple causes were built by using least absolute shrinkage and selection operator (LASSO) regression.

Then, DWs for each health state caused by the disease and injury sequelae were measured to compute YLDs. For GBD 2010, Disability Weights Measurement Study was conducted. Personal face-to-face computer-assisted interviews or computer-assisted telephone interviews were done in five countries (Bangladesh, Indonesia, Peru, Tanzania, and the USA) and a web-based survey was done subsequently. The survey consisted of paired comparison questions that presented two hypothetical people, each with a particular health state, and asked which person they regarded as healthier. For GBD 2013, European Disability Weights Measurement Study covering the expanded the list of disease and injury sequelae was conducted in Hungary, Italy, Netherlands, and Sweden, following the protocol for the GBD 2010 DWs measurement study with some modifications in health state descriptions.

As the final step for computing YLDs, comorbidity correction (COMO) was performed, where 20,000 individuals were simulated in each location-age-sex-year combination as having each of the sequelae included in GBD by the probability based on prevalence independently. The cumulative DW for each simulant was calculated and the DWs attributable to each sequela were yielded. Finally, YLDs per capita was obtained by adding up the attributable DWs across simulants.

For GBD 2021, socio-demographic index (SDI) was computed for 1075 national and subnational locations for the time period 1950-2021. SDI is defined as a geometric mean of 0 to 1 indices of three parameters: total fertility rate for those younger than 25 years old (TFU25), mean education for those 15 years old and older (EDU15+), and lag-distributed income (LDI) per capita. Each parameter was converted into a 0 to 1 index based on the minimum and maximum of the scale. The minimum and maximum were determined by the points of limiting returns at high and low values occurring in the relationships each of the parameters had with life expectancy at birth and under-5 mortality.

DALYs were measured as the sum of YLDs and years of life lost (YLLs). 500 draws of DALYs were calculated by adding YLDs and YLLs from 500 draws of each. The mean value of the draws was assigned the final of DALYs.

**Non-fatal estimation for ADHD**

**Case Definition**

Cases meeting diagnostic criteria for ADHD according to the Diagnostic and Statistical Manual of Mental Disorders (DSM) [5] or the International Classification of Diseases (ICD) [6] (referred to as “hyperkinetic disorder” in ICD) were included. These were cases coded as 314.0, 314.01 in DSM-IV-TR and F90 in ICD-10. Different versions of DSM (DSM-III, DSM-III-R, DSM-IV, DSM-IV-TR, DSM-5, and DSM-5-TR) and ICD (ICD-9, ICD-10, and ICD-11) were also accepted.

**Input data**

Systemic literature review on the epidemiology of ADHD was conducted in three stages: electronic searches of the peer-reviewed literature (via PsycInfo, Embase, and PubMed), the grey literature, and expert consultation. For GBD 2021 an updated electronic search was not required. The grey literature searches and expert consultation were done.

The inclusion criteria for data sources were specified as follows: 1) the publication year must be from 1980 onward; 2) case definition must be based on diagnostic criteria established by the DSM or ICD; 3) sufficient explanation of study method and sample characteristics must be provided to allow for study quality assessment; 4) study sample must be representative of the general population (i.e., inpatient or pharmacological treatment samples, case studies, veterans, or refugee samples were excluded). eTable 2 shows the number of data sources included in the systematic review by parameter. The citations for data sources can be found at the GBD Sources Tool in the GHDx website: <http://ghdx.healthdata.org/gbd-2021/sources>

**Age-sex splitting**

The extracted data went through age-sex splitting processes. First, where possible, the estimates were further split by sex and age based on the available data. Second, MR-BRT analysis was used to split the remaining data for both sexes. Sex-specific estimates were matched by location, age, and year for each parameter and were fed to a MR-BRT network meta-analysis to generate pooled sex ratio estimates. The male-to-female prevalence ratio was 2.38 (95% uncertainty interval [UI]: 1.24–3.51).

**Bias corrections/crosswalks**

Crosswalks were not applied to the ADHD dataset.

**Modelling strategy**

There were no substantive changes in the modelling strategy from GBD 2019. DisMod-MR 2.1 was used to estimate the burden of ADHD. Where appropriate, adjustments to model priors or the dataset were made and where outliers were identified in the data, a decision was made to exclude or include the data after a reassessment on the study’s methodology and quality. It was assumed that there is no ADHD incidence prior to 3 years of age and from 12 years of age onward. The minimum age limit was obtained from consultation with experts and review of current literature. The maximum age limit was set to reflect the latest DSM-5 criteria. In line with the age restriction on incidence, remission rate was set to zero prior to 12 years of age. Excess mortality was set to zero given the lack of data supporting an elevated mortality risk in ADHD patients.

**Severity splits and disability weights**

In the GBD disability weight survey assessments, the lay description assigned to ADHD was "Is hyperactive and has difficulty concentrating, remembering things, and completing tasks." The disability weight attributed to this condition was 0.045 (95% uncertainty interval: 0.028–0.066). For severity split, the proportion of time spent symptomatic versus asymptomatic gained from the Great Smoky Mountains Study [7] was used. The Great Smoky Mountains study assessed the levels of disability found in children and adolescents with mental disorders. At the time of survey, 48% of those with ADHD reported disability while 20% of individuals with no diagnosis reported disability. The latter proportion was subtracted from the former proportion to give an adjusted proportion of 28%. Detailed descriptions of the methodology can be found elsewhere. [8]

**References**

[1] GBD 2021 Diseases and Injuries Collaborators. Global incidence, prevalence, years lived with disability (YLDs), disability-adjusted life-years (DALYs), and healthy life expectancy (HALE) for 371 diseases and injuries in 204 countries and territories and 811 subnational locations, 1990-2021: a systematic analysis for the Global Burden of Disease Study 2021. Lancet. 2024;403(10440):2133-2161.

[2] GBD 2019 Risk Factors Collaborators. Global burden of 87 risk factors in 204 countries and territories, 1990-2019: a systematic analysis for the Global Burden of Disease Study 2019. Lancet. 2020;396(10258):1223-1249.

[3] Vos T, Flaxman AD, Naghavi M, et al. Years lived with disability (YLDs) for 1160 sequelae of 289 diseases and injuries 1990-2010: a systematic analysis for the Global Burden of Disease Study 2010 [published correction appears in Lancet. 2013 Feb 23;381(9867):628.

[4] Global Burden of Disease Study 2013 Collaborators. Global, regional, and national incidence, prevalence, and years lived with disability for 301 acute and chronic diseases and injuries in 188 countries, 1990-2013: a systematic analysis for the Global Burden of Disease Study 2013. Lancet. 2015;386(9995):743-800.

[5] American Psychiatric Association. Diagnostic and Statistical Manual of Mental Disorders (DSM-IV-TR). 4th, Text Revision ed. Washington DC: American Psychiatric Association; 2000.

[6] World Health Organization. ICD-10 Classification of Mental and Behavioural Disorders: Clinical descriptions and diagnostic guidelines. Geneva: World Health Organization; 1992.

[7] Ezpeleta L, Keeler G, Erkanli A, Costello EJ, Angold A. Epidemiology of Psychiatric Disability in Childhood and Adolescence. J Child Psychol Psychiatry 2001; 42(7): 901-14.

[8] Erskine HE, Ferrari AJ, Polanczyk GV, et al. The global burden of conduct disorder and attention-deficit/hyperactivity disorder in 2010. J Child Psychol Psychiatry 2014; 55(4): 328-36.

**Supplementary Tables and Figures**

**eTable 1. GATHER checklist of information that should be included in new reports of global health estimates.**

| **Item**  **#** | **Checklist item** | **Reported**  **On page #** |
| --- | --- | --- |
| **Objectives and funding** | | |
| **1** | Define the indicator(s), populations (including age, sex, and geographic entities), and time period(s) for which estimates were made. | Main text (Methods) and supplementary methods |
| **2** | List the funding sources for the work. | Main text (Funding) |
| **Data Inputs** | | |
| *For all data inputs from multiple sources that are synthesized as part of the study:* | | |
| **3** | Describe how the data were identified and how the data were accessed. | Main text (Methods) and supplementary methods |
| **4** | Specify the inclusion and exclusion criteria. Identify all ad‐hoc exclusions. | Main text (Methods) and supplementary methods |
| **5** | Provide information on all included data sources and their main characteristics. For each data source used, report reference information or contact name/institution, population represented, data collection method, year(s) of data collection, sex and age range, diagnostic criteria or measurement method, and sample size, as relevant. | Online data sources tools: https://ghdx.healthdata.org/gbd-2021/sources |
| **6** | Identify and describe any categories of input data that have potentially important biases (e.g., based on characteristics listed in item 5). | Main text (Discussion) and supplementary methods |
| *For data inputs that contribute to the analysis but were not synthesized as part of the study:* | | |
| **7** | Describe and give sources for any other data inputs. | Online data sources tools: https://ghdx.healthdata.org/gbd-2021/sources |
| *For all data inputs:* | | |
| **8** | Provide all data inputs in a file format from which data can be efficiently extracted (e.g., a spreadsheet rather than a PDF), including all relevant meta‐data listed in item 5. For any data inputs that cannot be shared because of ethical or legal reasons, such as third‐party ownership, provide a contact name or the name of the institution that retains the right to the data. | Online data visualisation tools, data query tools, and the Global Health Data Exchange |
| **Data analysis** | | |
| **9** | Provide a conceptual overview of the data analysis method. A diagram may be helpful. | Main text (Methods) and supplementary methods |
| **10** | Provide a detailed description of all steps of the analysis, including mathematical formulae. This description should cover, as relevant, data cleaning, data pre‐processing, data adjustments and weighting of data sources, and mathematical or statistical model(s). | Main text (Methods) and supplementary methods |
| **11** | Describe how candidate models were evaluated and how the final model(s) were selected. | Main text (Methods) and supplementary methods |
| **12** | Provide the results of an evaluation of model performance, if done, as well as the results of any relevant sensitivity analysis. | Main text (Methods) and supplementary methods |
| **13** | Describe methods for calculating uncertainty of the estimates. State which sources of uncertainty were, and were not, accounted for in the uncertainty analysis. | Main text (Methods) and supplementary methods |
| **14** | State how analytic or statistical source code used to generate estimates can be accessed. | https://ghdx.healthdata.org/gbd-2021/code |
| **Results and Discussion** | | |
| **15** | Provide published estimates in a file format from which data can be efficiently extracted. | Main text (Results) and online data visualisation tools, data query tools, and the Global Health Data Exchange |
| **16** | Report a quantitative measure of the uncertainty of the estimates (e.g. uncertainty intervals). | Main text (Results) and online data visualisation tools, data query tools, and the Global Health Data Exchange |
| **17** | Interpret results in light of existing evidence. If updating a previous set of estimates, describe the reasons for changes in estimates. | Main text (Methods and Discussion) |
| **18** | Discuss limitations of the estimates. Include a discussion of any modelling assumptions or data limitations that affect interpretation of the estimates. | Main text (Discussion) |

**eTable 2: Data inputs for ADHD morbidity modelling by parameter.**

| **Parameter** | **Countries with data** | **New sources** | **Total sources** |
| --- | --- | --- | --- |
| **Incidence** | 1 | 0 | 2 |
| **Prevalence** | 49 | 0 | 172 |
| **Remission** | 6 | 0 | 14 |
| **Excess mortality** | 2 | 0 | 3 |

**eTable** **3a. Incidence of ADHD by region, <20 years, 1990-2021.**

|  | **Incidence of ADHD** | | | | | | | | | | |  |
| --- | --- | --- | --- | --- | --- | --- | --- | --- | --- | --- | --- | --- |
|  | **Counts (<20 years)** | | | | |  | **Rate per 100,000 persons (<20 years)** | | | | |  |
| **Region** | **1990 (95% UI)** |  | **2021 (95% UI)** |  | **Percentage change from 1990 to 2021 (%)** |  | **1990 (95% UI)** |  | **2021 (95% UI)** |  | **Percentage change from 1990 to 2021 (%)** | |
| Global | 3740573 (2543085 to 5471097) |  | 4111621 (2775203 to 5954941) |  | 9.92 (7.13 to 13.16) |  | 165.62 (112.6 to 242.24) |  | 155.99 (105.29 to 225.92) |  | -5.81 (-8.21 to -3.04) | |
| High-income Asia Pacific | 88200 (58368 to 131104) |  | 56470 (38086 to 83674) |  | -35.97 (-38.48 to -32.59) |  | 175.25 (115.98 to 260.51) |  | 183.41 (123.7 to 271.77) |  | 4.65 (0.55 to 10.19) | |
| High-income North America | 236579 (156298 to 349636) |  | 260879 (170399 to 390296) |  | 10.27 (-0.16 to 19.5) |  | 289.47 (191.24 to 427.8) |  | 291.3 (190.27 to 435.8) |  | 0.63 (-8.88 to 9.05) | |
| Western Europe | 199791 (135233 to 293277) |  | 203223 (135369 to 298926) |  | 1.72 (-4.5 to 7.43) |  | 203.15 (137.51 to 298.21) |  | 221.59 (147.6 to 325.94) |  | 9.08 (2.41 to 15.2) | |
| Australasia | 26678 (18342 to 38230) |  | 34767 (24322 to 48285) |  | 30.32 (13.96 to 53.27) |  | 425.27 (292.39 to 609.42) |  | 460.99 (322.5 to 640.23) |  | 8.4 (-5.21 to 27.49) | |
| Andean Latin America | 56153 (38054 to 83133) |  | 69087 (46823 to 102346) |  | 23.03 (22.88 to 23.18) |  | 296.22 (200.75 to 438.56) |  | 291.83 (197.78 to 432.32) |  | -1.48 (-1.61 to -1.37) | |
| Tropical Latin America | 198998 (135032 to 291627) |  | 184936 (124007 to 276248) |  | -7.07 (-15.49 to 1.88) |  | 287.31 (194.96 to 421.05) |  | 277.73 (186.23 to 414.86) |  | -3.33 (-12.1 to 5.98) | |
| Central Latin America | 166039 (111432 to 241925) |  | 163567 (111756 to 240136) |  | -1.49 (-3.95 to 0.94) |  | 200.94 (134.85 to 292.78) |  | 191.79 (131.04 to 281.57) |  | -4.55 (-6.94 to -2.2) | |
| Southern Latin America | 38569 (25867 to 56936) |  | 37936 (25087 to 54948) |  | -1.64 (-11.94 to 8.18) |  | 199.02 (133.48 to 293.8) |  | 194.45 (128.59 to 281.64) |  | -2.3 (-12.53 to 7.46) | |
| Caribbean | 59736 (40991 to 88200) |  | 60858 (41055 to 90770) |  | 1.88 (-0.46 to 3.54) |  | 395.63 (271.48 to 584.15) |  | 398.74 (268.99 to 594.72) |  | 0.79 (-1.53 to 2.43) | |
| Central Europe | 52533 (35249 to 77759) |  | 31546 (21175 to 46692) |  | -39.95 (-40.01 to -39.9) |  | 133.78 (89.76 to 198.02) |  | 133.91 (89.89 to 198.21) |  | 0.1 (0 to 0.18) | |
| Eastern Europe | 96689 (63795 to 144916) |  | 66512 (43875 to 99892) |  | -31.21 (-31.41 to -31.03) |  | 143.72 (94.83 to 215.41) |  | 144.09 (95.05 to 216.41) |  | 0.26 (-0.04 to 0.53) | |
| Central Asia | 45815 (30666 to 68254) |  | 51366 (34393 to 76565) |  | 12.12 (11.9 to 12.29) |  | 145.08 (97.11 to 216.13) |  | 148.35 (99.33 to 221.13) |  | 2.26 (2.06 to 2.42) | |
| North Africa and Middle East | 331103 (227615 to 493290) |  | 392269 (267369 to 582116) |  | 18.47 (13.68 to 23.95) |  | 187.3 (128.76 to 279.05) |  | 165.87 (113.06 to 246.15) |  | -11.44 (-15.03 to -7.35) | |
| South Asia | 458767 (303134 to 692596) |  | 511250 (339697 to 756376) |  | 11.44 (2.8 to 19.65) |  | 84.58 (55.88 to 127.68) |  | 74.8 (49.7 to 110.66) |  | -11.56 (-18.42 to -5.05) | |
| Southeast Asia | 302200 (210111 to 443989) |  | 286808 (191716 to 424134) |  | -5.09 (-9.81 to -2.5) |  | 137.43 (95.55 to 201.9) |  | 125.1 (83.62 to 184.99) |  | -8.97 (-13.5 to -6.48) | |
| East Asia | 1162373 (800239 to 1736965) |  | 1225314 (835309 to 1772637) |  | 5.41 (-2.88 to 14.74) |  | 252.61 (173.91 to 377.49) |  | 355.21 (242.15 to 513.88) |  | 40.62 (29.55 to 53.05) | |
| Oceania | 5595 (3803 to 8376) |  | 10615 (7215 to 15889) |  | 89.71 (89.69 to 89.72) |  | 166.21 (112.98 to 248.8) |  | 166.21 (112.97 to 248.8) |  | 0 (-0.01 to 0.01) | |
| Western Sub-Saharan Africa | 85672 (56242 to 129714) |  | 215764 (140538 to 322844) |  | 151.85 (138.26 to 164.36) |  | 79.7 (52.32 to 120.67) |  | 80.34 (52.33 to 120.21) |  | 0.8 (-4.64 to 5.81) | |
| Eastern Sub-Saharan Africa | 85601 (56078 to 127383) |  | 169212 (111067 to 251831) |  | 97.67 (96.49 to 99.03) |  | 77.19 (50.57 to 114.87) |  | 74.35 (48.8 to 110.65) |  | -3.68 (-4.26 to -3.02) | |
| Central Sub-Saharan Africa | 24027 (15846 to 35523) |  | 56589 (37326 to 83653) |  | 135.53 (135.42 to 135.64) |  | 77.53 (51.13 to 114.64) |  | 76.93 (50.74 to 113.72) |  | -0.78 (-0.83 to -0.74) | |
| Southern Sub-Saharan Africa | 19456 (12645 to 29026) |  | 22652 (14720 to 33838) |  | 16.43 (16.07 to 16.87) |  | 73.53 (47.79 to 109.7) |  | 72.45 (47.08 to 108.23) |  | -1.46 (-1.76 to -1.09) | |

Abbreviations: UI, uncertainty interval.

**eTable** **3b. DALYs caused by ADHD by region, <20 years, 1990-2021.**

|  | **DALYs from ADHD** | | | | | | | | | | |
| --- | --- | --- | --- | --- | --- | --- | --- | --- | --- | --- | --- |
|  | **Counts (<20 years)** | | | | |  | **Rate per 100,000 persons (<20 years)** | | | | |
| **Region** | **1990 (95% UI)** |  | **2021 (95% UI)** |  | **Percentage change from 1990 to 2021 (%)** |  | **1990 (95% UI)** |  | **2021 (95% UI)** |  | **Percentage change from 1990 to 2021 (%)** |
| Global | 523435 (271668 to 876127) |  | 574979 (294277 to 977557) |  | 9.85 (6.84 to 12.86) |  | 23.18 (12.03 to 38.79) |  | 21.81 (11.16 to 37.09) |  | -5.88 (-8.45 to -3.29) |
| High-income Asia Pacific | 14682 (7308 to 24954) |  | 8840 (4473 to 15054) |  | -39.79 (-42.73 to -36.58) |  | 29.17 (14.52 to 49.58) |  | 28.71 (14.53 to 48.89) |  | -1.59 (-6.4 to 3.67) |
| High-income North America | 32194 (16774 to 54869) |  | 39803 (19988 to 69498) |  | 23.63 (11.49 to 34.5) |  | 39.39 (20.52 to 67.13) |  | 44.44 (22.32 to 77.6) |  | 12.83 (1.75 to 22.75) |
| Western Europe | 29163 (15606 to 48725) |  | 28442 (14530 to 47980) |  | -2.47 (-8.89 to 4.06) |  | 29.65 (15.87 to 49.55) |  | 31.01 (15.84 to 52.32) |  | 4.58 (-2.3 to 11.58) |
| Australasia | 3947 (2077 to 6750) |  | 4959 (2644 to 7877) |  | 25.63 (8.99 to 49.36) |  | 62.92 (33.11 to 107.6) |  | 65.75 (35.06 to 104.44) |  | 4.5 (-9.34 to 24.24) |
| Andean Latin America | 7611 (3906 to 13080) |  | 9857 (5074 to 16759) |  | 29.5 (23.89 to 34.98) |  | 40.15 (20.61 to 69) |  | 41.64 (21.43 to 70.79) |  | 3.7 (-0.8 to 8.08) |
| Tropical Latin America | 28494 (14704 to 48214) |  | 27197 (14287 to 46898) |  | -4.55 (-13.46 to 4.89) |  | 41.14 (21.23 to 69.61) |  | 40.84 (21.46 to 70.43) |  | -0.72 (-9.98 to 9.11) |
| Central Latin America | 22565 (11856 to 38108) |  | 24707 (13016 to 40968) |  | 9.49 (5.84 to 13.56) |  | 27.31 (14.35 to 46.12) |  | 28.97 (15.26 to 48.04) |  | 6.09 (2.55 to 10.02) |
| Southern Latin America | 5111 (2632 to 8737) |  | 5425 (2797 to 9159) |  | 6.14 (-4.76 to 18.13) |  | 26.37 (13.58 to 45.08) |  | 27.81 (14.34 to 46.95) |  | 5.43 (-5.4 to 17.34) |
| Caribbean | 8531 (4488 to 14381) |  | 8929 (4679 to 15251) |  | 4.66 (0.76 to 8.15) |  | 56.5 (29.72 to 95.25) |  | 58.5 (30.66 to 99.93) |  | 3.54 (-0.32 to 6.99) |
| Central Europe | 8078 (4026 to 13867) |  | 4842 (2455 to 8267) |  | -40.06 (-41.78 to -38.07) |  | 20.57 (10.25 to 35.31) |  | 20.56 (10.42 to 35.09) |  | -0.08 (-2.95 to 3.23) |
| Eastern Europe | 13650 (7069 to 23653) |  | 9850 (5071 to 17085) |  | -27.84 (-29.77 to -26.02) |  | 20.29 (10.51 to 35.16) |  | 21.34 (10.98 to 37.01) |  | 5.17 (2.36 to 7.83) |
| Central Asia | 5815 (2864 to 9951) |  | 6545 (3219 to 11480) |  | 12.55 (6.99 to 17.97) |  | 18.41 (9.07 to 31.51) |  | 18.9 (9.3 to 33.15) |  | 2.65 (-2.42 to 7.6) |
| North Africa and Middle East | 42267 (21432 to 72004) |  | 53893 (27302 to 91288) |  | 27.51 (21.52 to 34.04) |  | 23.91 (12.12 to 40.73) |  | 22.79 (11.54 to 38.6) |  | -4.69 (-9.17 to 0.19) |
| South Asia | 57538 (29944 to 97866) |  | 78275 (38853 to 132257) |  | 36.04 (25.19 to 47.18) |  | 10.61 (5.52 to 18.04) |  | 11.45 (5.68 to 19.35) |  | 7.96 (-0.65 to 16.8) |
| Southeast Asia | 41668 (21140 to 70660) |  | 42070 (20872 to 71570) |  | 0.96 (-4.58 to 5.07) |  | 18.95 (9.61 to 32.13) |  | 18.35 (9.1 to 31.22) |  | -3.16 (-8.48 to 0.77) |
| East Asia | 175894 (91414 to 296914) |  | 160363 (85161 to 272639) |  | -8.83 (-15.97 to -0.48) |  | 38.23 (19.87 to 64.53) |  | 46.49 (24.69 to 79.04) |  | 21.61 (12.09 to 32.76) |
| Oceania | 698 (360 to 1201) |  | 1320 (670 to 2284) |  | 89.04 (73.86 to 104.17) |  | 20.75 (10.68 to 35.68) |  | 20.67 (10.49 to 35.77) |  | -0.35 (-8.35 to 7.62) |
| Western Sub-Saharan Africa | 9998 (5038 to 17184) |  | 27012 (13543 to 45474) |  | 170.19 (154.77 to 186.66) |  | 9.3 (4.69 to 15.99) |  | 10.06 (5.04 to 16.93) |  | 8.14 (1.97 to 14.73) |
| Eastern Sub-Saharan Africa | 10167 (5168 to 17685) |  | 22332 (11524 to 38475) |  | 119.66 (111.26 to 128.14) |  | 9.17 (4.66 to 15.95) |  | 9.81 (5.06 to 16.91) |  | 7.03 (2.94 to 11.17) |
| Central Sub-Saharan Africa | 2787 (1421 to 4733) |  | 7136 (3644 to 12316) |  | 156.1 (131.93 to 184.56) |  | 8.99 (4.58 to 15.27) |  | 9.7 (4.95 to 16.74) |  | 7.88 (-2.3 to 19.87) |
| Southern Sub-Saharan Africa | 2576 (1314 to 4466) |  | 3181 (1628 to 5547) |  | 23.49 (17.27 to 30.79) |  | 9.74 (4.97 to 16.88) |  | 10.18 (5.21 to 17.74) |  | 4.52 (-0.75 to 10.69) |

Abbreviations: DALYs, disability-adjusted life-years; UI, uncertainty interval.

**eTable** **4a. Prevalence of ADHD by country, <20 years, 1990-2021.**

|  | **Prevalence of ADHD** | | | | | |
| --- | --- | --- | --- | --- | --- | --- |
|  | **Counts (<20 years)** | | | **Rates per 100,000 persons (<20 years)** | | |
| **Country** | **1990 (95% UI)** | **2021 (95% UI)** | **Percentage change from 1990 to 2021 (%)** | **1990 (95% UI)** | **2021 (95% UI)** | **Percentage change from 1990 to 2021 (%)** |
| Global | 42742759 (29295029 to 61474615) | 46890733 (32136904 to 67271064) | 9.7 (6.81 to 12.68) | 1892.47 (1297.06 to 2721.83) | 1778.97 (1219.23 to 2552.17) | -6 (-8.48 to -3.45) |
| Central Europe | 658129 (448634 to 948066) | 394499 (268968 to 568460) | -40.06 (-40.12 to -39.99) | 1675.98 (1142.48 to 2414.33) | 1674.64 (1141.76 to 2413.1) | -0.08 (-0.19 to 0.03) |
| Albania | 22544 (15323 to 32999) | 10309 (7007 to 14971) | -54.27 (-54.73 to -53.99) | 1559.14 (1059.73 to 2282.2) | 1670.45 (1135.45 to 2425.96) | 7.14 (6.08 to 7.81) |
| Bosnia and Herzegovina | 24349 (16558 to 35443) | 11096 (7545 to 16153) | -54.43 (-54.45 to -54.41) | 1643.24 (1117.44 to 2391.93) | 1668.32 (1134.45 to 2428.78) | 1.53 (1.49 to 1.56) |
| Bulgaria | 39504 (26861 to 57436) | 21480 (14608 to 31337) | -45.63 (-45.76 to -45.45) | 1671.58 (1136.6 to 2430.36) | 1659.6 (1128.61 to 2421.19) | -0.72 (-0.96 to -0.4) |
| Croatia | 22098 (15030 to 32203) | 13490 (9173 to 19641) | -38.95 (-39.01 to -38.91) | 1665.95 (1133.07 to 2427.77) | 1674.26 (1138.52 to 2437.68) | 0.5 (0.4 to 0.57) |
| Czechia | 51880 (35269 to 75286) | 35961 (24444 to 52587) | -30.68 (-31.08 to -30.15) | 1704.41 (1158.7 to 2473.37) | 1619.48 (1100.82 to 2368.22) | -4.98 (-5.52 to -4.25) |
| Hungary | 49625 (33742 to 72104) | 30699 (20875 to 44678) | -38.14 (-38.21 to -38.04) | 1709.91 (1162.61 to 2484.45) | 1636.95 (1113.09 to 2382.32) | -4.27 (-4.37 to -4.11) |
| Montenegro | 3496 (2378 to 5100) | 2479 (1685 to 3611) | -29.08 (-29.28 to -28.94) | 1629.67 (1108.41 to 2377.59) | 1648.57 (1120.72 to 2401.03) | 1.16 (0.88 to 1.36) |
| North Macedonia | 11356 (7723 to 16575) | 7467 (5077 to 10868) | -34.25 (-34.47 to -34.09) | 1629.37 (1108.14 to 2378.27) | 1672.08 (1136.83 to 2433.79) | 2.62 (2.26 to 2.87) |
| Poland | 212445 (141447 to 307808) | 130760 (87151 to 189586) | -38.45 (-38.55 to -38.38) | 1706.45 (1136.17 to 2472.45) | 1698.49 (1132.03 to 2462.6) | -0.47 (-0.63 to -0.35) |
| Romania | 124377 (84582 to 180925) | 67232 (45721 to 97917) | -45.94 (-45.99 to -45.89) | 1657.72 (1127.33 to 2411.41) | 1663.79 (1131.46 to 2423.17) | 0.37 (0.28 to 0.46) |
| Serbia | 47529 (32325 to 69159) | 33054 (22465 to 47964) | -30.46 (-31.03 to -30.07) | 1638.3 (1114.25 to 2383.9) | 1763.93 (1198.85 to 2559.65) | 7.67 (6.78 to 8.27) |
| Slovakia | 29038 (19750 to 42365) | 18089 (12300 to 26415) | -37.7 (-37.74 to -37.66) | 1654.4 (1125.24 to 2413.68) | 1613.53 (1097.15 to 2356.19) | -2.47 (-2.53 to -2.4) |
| Slovenia | 9364 (6369 to 13642) | 6641 (4514 to 9719) | -29.08 (-29.31 to -28.77) | 1681.85 (1143.86 to 2450.23) | 1640.57 (1114.95 to 2400.83) | -2.45 (-2.77 to -2.03) |
| Eastern Europe | 1111138 (742566 to 1615665) | 801572 (534701 to 1164564) | -27.86 (-28.07 to -27.6) | 1651.63 (1103.77 to 2401.57) | 1736.52 (1158.37 to 2522.91) | 5.14 (4.83 to 5.52) |
| Belarus | 50076 (34052 to 73209) | 33538 (22780 to 49135) | -33.03 (-33.22 to -32.78) | 1595.78 (1085.15 to 2332.98) | 1655.96 (1124.8 to 2426.08) | 3.77 (3.48 to 4.15) |
| Estonia | 7302 (4966 to 10656) | 4579 (3113 to 6696) | -37.28 (-37.41 to -37.12) | 1589.64 (1081.15 to 2320.03) | 1633.74 (1110.51 to 2388.67) | 2.77 (2.57 to 3.05) |
| Latvia | 11819 (8037 to 17242) | 6335 (4306 to 9269) | -46.4 (-46.54 to -46.21) | 1570.82 (1068.21 to 2291.55) | 1636.65 (1112.45 to 2394.47) | 4.19 (3.92 to 4.55) |
| Lithuania | 17590 (11962 to 25628) | 8705 (5919 to 12721) | -50.51 (-50.62 to -50.36) | 1588.04 (1079.91 to 2313.78) | 1624.71 (1104.75 to 2374.18) | 2.31 (2.07 to 2.61) |
| Republic of Moldova | 24757 (16827 to 36252) | 11701 (7958 to 17073) | -52.74 (-52.96 to -52.59) | 1565.62 (1064.16 to 2292.57) | 1680.62 (1142.95 to 2452.18) | 7.35 (6.85 to 7.67) |
| Russian Federation | 747900 (498277 to 1083695) | 583216 (388079 to 844863) | -22.02 (-22.15 to -21.83) | 1655.19 (1102.75 to 2398.35) | 1726.02 (1148.51 to 2500.36) | 4.28 (4.11 to 4.54) |
| Ukraine | 251695 (167889 to 364910) | 153497 (102303 to 222592) | -39.01 (-39.12 to -38.86) | 1672.2 (1115.42 to 2424.38) | 1817.56 (1211.37 to 2635.72) | 8.69 (8.51 to 8.97) |
| Central Asia | 474990 (322822 to 695555) | 533501 (362199 to 781863) | 12.32 (12.07 to 12.62) | 1504.09 (1022.24 to 2202.53) | 1540.81 (1046.07 to 2258.11) | 2.44 (2.21 to 2.72) |
| Armenia | 20410 (13869 to 29894) | 12626 (8572 to 18510) | -38.14 (-38.35 to -37.98) | 1537.14 (1044.51 to 2251.37) | 1654.58 (1123.33 to 2425.53) | 7.64 (7.26 to 7.92) |
| Azerbaijan | 48166 (32743 to 70469) | 50946 (34583 to 74693) | 5.77 (5.43 to 6.12) | 1538.79 (1046.06 to 2251.33) | 1672.12 (1135.06 to 2451.53) | 8.66 (8.31 to 9.03) |
| Georgia | 28670 (19499 to 41856) | 14949 (10145 to 21916) | -47.86 (-48.13 to -47.53) | 1595.61 (1085.21 to 2329.46) | 1608.59 (1091.73 to 2358.35) | 0.81 (0.29 to 1.44) |
| Kazakhstan | 102586 (69741 to 150157) | 103156 (70027 to 151180) | 0.56 (0.19 to 1.04) | 1544.25 (1049.83 to 2260.34) | 1534.13 (1041.43 to 2248.34) | -0.66 (-1.02 to -0.18) |
| Kyrgyzstan | 31584 (21465 to 46250) | 43687 (29656 to 64022) | 38.32 (37.94 to 38.79) | 1495.89 (1016.63 to 2190.49) | 1547.62 (1050.56 to 2268.01) | 3.46 (3.18 to 3.81) |
| Mongolia | 17138 (11648 to 25096) | 19817 (13430 to 29059) | 15.63 (14.94 to 16.54) | 1509.77 (1026.11 to 2210.87) | 1509.65 (1023.05 to 2213.68) | -0.01 (-0.61 to 0.77) |
| Tajikistan | 41492 (28185 to 60774) | 67479 (45808 to 98896) | 62.63 (62.3 to 62.89) | 1443.48 (980.53 to 2114.27) | 1514.64 (1028.2 to 2219.83) | 4.93 (4.72 to 5.1) |
| Turkmenistan | 28003 (19030 to 41010) | 30770 (20911 to 45051) | 9.88 (9.54 to 10.12) | 1485.41 (1009.44 to 2175.32) | 1569.45 (1066.59 to 2297.86) | 5.66 (5.33 to 5.89) |
| Uzbekistan | 156939 (106633 to 229836) | 190071 (129059 to 278520) | 21.11 (20.84 to 21.32) | 1470.05 (998.83 to 2152.87) | 1507.29 (1023.45 to 2208.7) | 2.53 (2.3 to 2.71) |
| Australasia | 322192 (224879 to 449822) | 404699 (296904 to 539136) | 25.61 (10.25 to 46.83) | 5136.04 (3584.79 to 7170.58) | 5366.09 (3936.79 to 7148.66) | 4.48 (-8.3 to 22.13) |
| Australia | 280868 (196833 to 391160) | 350565 (259231 to 465536) | 24.82 (7.1 to 49.15) | 5430.48 (3805.69 to 7562.94) | 5619.56 (4155.47 to 7462.53) | 3.48 (-11.21 to 23.65) |
| New Zealand | 41324 (27738 to 59516) | 54134 (36726 to 76953) | 31 (16.75 to 53.37) | 3753.01 (2519.14 to 5405.21) | 4153.03 (2817.51 to 5903.7) | 10.66 (-1.38 to 29.56) |
| High-income Asia Pacific | 1197468 (808502 to 1723217) | 719705 (494654 to 1039400) | -39.9 (-42.1 to -37.04) | 2379.39 (1606.51 to 3424.06) | 2337.54 (1606.59 to 3375.88) | -1.76 (-5.37 to 2.91) |
| Brunei Darussalam | 2377 (1564 to 3522) | 2907 (1917 to 4268) | 22.27 (20.99 to 23.32) | 2066.7 (1359.7 to 3061.67) | 2250.79 (1484.57 to 3305.2) | 8.91 (7.77 to 9.85) |
| Japan | 807011 (547083 to 1177252) | 504755 (341501 to 737385) | -37.45 (-37.73 to -37.06) | 2418.84 (1639.76 to 3528.55) | 2377.2 (1608.33 to 3472.79) | -1.72 (-2.16 to -1.1) |
| Republic of Korea | 367032 (242175 to 538239) | 190052 (133085 to 273477) | -48.22 (-55.62 to -38.98) | 2304.45 (1520.52 to 3379.39) | 2267.04 (1587.51 to 3262.19) | -1.62 (-15.69 to 15.93) |
| Singapore | 21047 (13893 to 30819) | 21991 (14484 to 32530) | 4.49 (3.2 to 6.12) | 2285.32 (1508.53 to 3346.4) | 2107.66 (1388.16 to 3117.66) | -7.77 (-8.91 to -6.33) |
| High-income North America | 2626253 (1771092 to 3797232) | 3248386 (2154251 to 4639606) | 23.69 (11.75 to 33.92) | 3213.33 (2167.01 to 4646.08) | 3627.13 (2405.43 to 5180.56) | 12.88 (1.98 to 22.22) |
| Canada | 231319 (172940 to 309239) | 259261 (171174 to 380288) | 12.08 (-10.78 to 36.28) | 3017.97 (2256.32 to 4034.58) | 3150.62 (2080.17 to 4621.39) | 4.4 (-16.9 to 26.93) |
| Greenland | 549 (372 to 800) | 499 (338 to 725) | -9.11 (-9.59 to -8.76) | 3067.46 (2078.61 to 4473.15) | 3245.49 (2199.72 to 4713.68) | 5.8 (5.24 to 6.2) |
| United States of America | 2394326 (1586163 to 3497682) | 2988575 (1979170 to 4285776) | 24.82 (12.91 to 36.53) | 3233.59 (2142.15 to 4723.7) | 3675.43 (2434.03 to 5270.76) | 13.66 (2.82 to 24.33) |
| Southern Latin America | 416672 (278877 to 612637) | 442487 (299173 to 642188) | 6.2 (-4.64 to 16.2) | 2150.09 (1439.05 to 3161.3) | 2268.04 (1533.46 to 3291.64) | 5.49 (-5.28 to 15.43) |
| Argentina | 281081 (188021 to 413719) | 310068 (209349 to 457945) | 10.31 (-5.77 to 25.17) | 2157.71 (1443.34 to 3175.9) | 2261.37 (1526.81 to 3339.86) | 4.8 (-10.47 to 18.92) |
| Chile | 111951 (75050 to 164084) | 111704 (74801 to 163900) | -0.22 (-0.37 to -0.04) | 2122.47 (1422.87 to 3110.87) | 2282.59 (1528.5 to 3349.18) | 7.54 (7.38 to 7.73) |
| Uruguay | 23620 (15811 to 34711) | 20690 (13870 to 30276) | -12.41 (-12.78 to -12.15) | 2193.1 (1468.02 to 3222.83) | 2290.42 (1535.47 to 3351.58) | 4.44 (3.99 to 4.74) |
| Western Europe | 2378130 (1657164 to 3324169) | 2320982 (1560149 to 3287210) | -2.4 (-8.85 to 3.25) | 2418.14 (1685.04 to 3380.09) | 2530.76 (1701.16 to 3584.32) | 4.66 (-2.25 to 10.72) |
| Andorra | 327 (219 to 472) | 360 (241 to 519) | 10.08 (9.91 to 10.3) | 2431.83 (1628.08 to 3504.83) | 2505.77 (1677.45 to 3608.45) | 3.04 (2.88 to 3.24) |
| Austria | 50853 (33956 to 72968) | 48040 (32593 to 68357) | -5.53 (-17.75 to 10.63) | 2714.76 (1812.74 to 3895.38) | 2737.48 (1857.26 to 3895.23) | 0.84 (-12.2 to 18.09) |
| Belgium | 56973 (38069 to 82161) | 59720 (39849 to 86164) | 4.82 (4.31 to 5.46) | 2304.99 (1540.19 to 3324.04) | 2347.24 (1566.25 to 3386.63) | 1.83 (1.33 to 2.46) |
| Cyprus | 6008 (4006 to 8678) | 6489 (4327 to 9373) | 8.01 (7.98 to 8.04) | 2318.97 (1546.16 to 3349.76) | 2276.14 (1517.71 to 3287.67) | -1.85 (-1.88 to -1.82) |
| Denmark | 29591 (20051 to 43426) | 28586 (19075 to 41022) | -3.4 (-20.67 to 12.95) | 2366.78 (1603.69 to 3473.31) | 2208.74 (1473.9 to 3169.64) | -6.68 (-23.36 to 9.12) |
| Finland | 43845 (28784 to 58943) | 34927 (23568 to 50227) | -20.34 (-44.32 to -1.02) | 3455.72 (2268.65 to 4645.71) | 3041.04 (2052 to 4373.17) | -12 (-38.49 to 9.34) |
| France | 425118 (286295 to 620419) | 430948 (289794 to 624516) | 1.37 (-12.42 to 18.79) | 2633.63 (1773.61 to 3843.53) | 2735.89 (1839.77 to 3964.76) | 3.88 (-10.25 to 21.74) |
| Germany | 180395 (119494 to 263213) | 183030 (118536 to 269335) | 1.46 (-13.31 to 16.86) | 1040.92 (689.51 to 1518.79) | 1147.74 (743.31 to 1688.94) | 10.26 (-5.79 to 27) |
| Greece | 68554 (45829 to 98801) | 45267 (30259 to 65217) | -33.97 (-34.02 to -33.9) | 2439.17 (1630.59 to 3515.36) | 2375.85 (1588.13 to 3422.95) | -2.6 (-2.67 to -2.5) |
| Iceland | 2246 (1366 to 3427) | 2395 (1453 to 3658) | 6.61 (6.44 to 6.85) | 2657.66 (1616.12 to 4055.02) | 2687.34 (1630.56 to 4104.95) | 1.12 (0.95 to 1.34) |
| Ireland | 42266 (27984 to 62239) | 41691 (27592 to 61337) | -1.36 (-1.59 to -1.06) | 3180.74 (2105.93 to 4683.81) | 3154.56 (2087.78 to 4641.13) | -0.82 (-1.05 to -0.52) |
| Israel | 51296 (34809 to 71337) | 83891 (54474 to 120226) | 63.54 (37.7 to 90.34) | 2556.73 (1734.98 to 3555.62) | 2486.82 (1614.81 to 3563.9) | -2.73 (-18.1 to 13.2) |
| Italy | 280961 (188904 to 403492) | 228978 (149815 to 330798) | -18.5 (-28.23 to -7.34) | 2062.91 (1386.99 to 2962.57) | 2184.98 (1429.59 to 3156.59) | 5.92 (-6.73 to 20.42) |
| Luxembourg | 1993 (1330 to 2876) | 3129 (2088 to 4514) | 56.95 (56.72 to 57.22) | 2259.28 (1507.81 to 3260.11) | 2318.98 (1547.93 to 3345.53) | 2.64 (2.49 to 2.82) |
| Malta | 2678 (1787 to 3865) | 1909 (1273 to 2757) | -28.72 (-28.79 to -28.63) | 2323.79 (1550.31 to 3353.89) | 2273.67 (1516.29 to 3283.73) | -2.16 (-2.24 to -2.03) |
| Monaco | 112 (75 to 162) | 156 (104 to 225) | 38.77 (38.46 to 39.14) | 2318.04 (1549.63 to 3343.14) | 2302.02 (1537.25 to 3319.64) | -0.69 (-0.92 to -0.43) |
| Netherlands | 92658 (61723 to 131615) | 98865 (65950 to 139631) | 6.7 (-8.11 to 23.02) | 2417.34 (1610.27 to 3433.68) | 2678.49 (1786.74 to 3782.96) | 10.8 (-4.57 to 27.75) |
| Norway | 18803 (12203 to 27918) | 21758 (14069 to 32258) | 15.72 (14.97 to 16.65) | 1690.08 (1096.81 to 2509.36) | 1752.6 (1133.27 to 2598.44) | 3.7 (3.03 to 4.54) |
| Portugal | 72819 (48728 to 104919) | 45287 (30313 to 65279) | -37.81 (-37.94 to -37.71) | 2442.8 (1634.64 to 3519.63) | 2362.68 (1581.46 to 3405.69) | -3.28 (-3.49 to -3.12) |
| San Marino | 144 (96 to 208) | 154 (103 to 221) | 6.64 (6.1 to 7.32) | 2407.73 (1612.97 to 3471.25) | 2461.95 (1648.34 to 3547.72) | 2.25 (1.74 to 2.91) |
| Spain | 538539 (405604 to 713366) | 454565 (305861 to 644757) | -15.59 (-32.47 to 4.05) | 4831.23 (3638.67 to 6399.6) | 5161.2 (3472.78 to 7320.66) | 6.83 (-14.53 to 31.69) |
| Sweden | 41421 (28169 to 59087) | 49643 (33783 to 71187) | 19.85 (5.67 to 35.25) | 1965.86 (1336.91 to 2804.3) | 2055.35 (1398.72 to 2947.32) | 4.55 (-7.82 to 17.99) |
| Switzerland | 44775 (31213 to 62604) | 49596 (33449 to 71770) | 10.77 (-4.19 to 29.36) | 2838.97 (1979.04 to 3969.39) | 2813.82 (1897.69 to 4071.87) | -0.89 (-14.27 to 15.75) |
| United Kingdom | 323798 (215077 to 475836) | 399559 (266674 to 588808) | 23.4 (11.34 to 37.43) | 2182.81 (1449.9 to 3207.75) | 2552.33 (1703.48 to 3761.23) | 16.93 (5.5 to 30.22) |
| Andean Latin America | 622383 (420248 to 900864) | 804774 (543647 to 1164003) | 29.31 (28.8 to 29.74) | 3283.29 (2216.95 to 4752.37) | 3399.46 (2296.43 to 4916.89) | 3.54 (3.13 to 3.89) |
| Bolivia (Plurinational State of) | 106595 (71893 to 154440) | 152894 (103265 to 221137) | 43.43 (42.71 to 44.02) | 3183.01 (2146.78 to 4611.71) | 3362.68 (2271.16 to 4863.59) | 5.64 (5.11 to 6.08) |
| Ecuador | 164214 (110888 to 237661) | 226317 (152836 to 327363) | 37.82 (37.53 to 38.05) | 3312.41 (2236.76 to 4793.95) | 3420.61 (2310 to 4947.85) | 3.27 (3.05 to 3.44) |
| Peru | 351575 (237411 to 508763) | 425563 (287547 to 615503) | 21.04 (20.44 to 21.6) | 3301.26 (2229.27 to 4777.25) | 3401.64 (2298.44 to 4919.89) | 3.04 (2.53 to 3.52) |
| Caribbean | 699005 (487690 to 987469) | 731015 (504345 to 1043717) | 4.58 (2.06 to 6.43) | 4629.48 (3229.95 to 6539.97) | 4789.6 (3304.46 to 6838.41) | 3.46 (0.96 to 5.29) |
| Antigua and Barbuda | 1138 (785 to 1627) | 1164 (804 to 1655) | 2.35 (1.74 to 2.8) | 4761.91 (3285.08 to 6809.5) | 4983.41 (3439.29 to 7081.57) | 4.65 (4.03 to 5.12) |
| Bahamas | 5238 (3614 to 7480) | 6018 (4152 to 8542) | 14.89 (14.21 to 15.42) | 4865.28 (3356.98 to 6947.39) | 5221.07 (3602.05 to 7410.93) | 7.31 (6.67 to 7.8) |
| Barbados | 4186 (2889 to 5961) | 3404 (2349 to 4830) | -18.68 (-18.94 to -18.47) | 4930.81 (3402.97 to 7020.95) | 5128.49 (3539.29 to 7276.97) | 4.01 (3.67 to 4.28) |
| Belize | 4670 (3219 to 6717) | 8353 (5764 to 11886) | 78.88 (77.02 to 80.24) | 4552.43 (3137.92 to 6548.12) | 4941.15 (3409.77 to 7031.04) | 8.54 (7.41 to 9.36) |
| Bermuda | 743 (513 to 1060) | 575 (397 to 819) | -22.65 (-22.76 to -22.57) | 4674.85 (3226.62 to 6663.08) | 5001.3 (3450.97 to 7122.73) | 6.98 (6.83 to 7.08) |
| Cuba | 177300 (122153 to 250002) | 119761 (82628 to 170748) | -32.45 (-32.97 to -31.76) | 4854.84 (3344.81 to 6845.57) | 4987.57 (3441.11 to 7110.96) | 2.73 (1.95 to 3.78) |
| Dominica | 1567 (1081 to 2237) | 1025 (707 to 1455) | -34.6 (-34.99 to -34.32) | 4781.42 (3297.79 to 6825.24) | 5275.3 (3640.82 to 7487.86) | 10.33 (9.68 to 10.81) |
| Dominican Republic | 159100 (109769 to 227626) | 183323 (126491 to 261688) | 15.23 (14.93 to 15.52) | 4542.92 (3134.34 to 6499.61) | 4711.84 (3251.13 to 6726.02) | 3.72 (3.45 to 3.98) |
| Grenada | 1937 (1335 to 2783) | 1508 (1041 to 2143) | -22.13 (-22.94 to -21.53) | 4594.74 (3167.29 to 6601.01) | 5010.35 (3458.09 to 7118.34) | 9.05 (7.91 to 9.89) |
| Guyana | 17183 (11855 to 24556) | 13216 (9118 to 18883) | -23.09 (-23.21 to -22.98) | 4503.03 (3106.74 to 6435.34) | 4707.94 (3248.05 to 6726.95) | 4.55 (4.38 to 4.7) |
| Haiti | 144769 (99798 to 208551) | 258265 (178125 to 369939) | 78.4 (77.46 to 79.2) | 4326.09 (2982.23 to 6232.04) | 4605.49 (3176.41 to 6596.92) | 6.46 (5.9 to 6.94) |
| Jamaica | 51871 (35780 to 74222) | 41382 (28558 to 58780) | -20.22 (-20.78 to -19.81) | 4747.87 (3275.05 to 6793.72) | 5075.28 (3502.48 to 7209) | 6.9 (6.15 to 7.45) |
| Puerto Rico | 64245 (48913 to 82277) | 35233 (24303 to 49852) | -45.16 (-56.14 to -33.62) | 4822.88 (3671.88 to 6176.56) | 5391.5 (3719.01 to 7628.7) | 11.79 (-10.59 to 35.31) |
| Saint Kitts and Nevis | 875 (603 to 1252) | 702 (484 to 994) | -19.74 (-20.52 to -19.14) | 4760.55 (3283.32 to 6816.7) | 4991.7 (3441.46 to 7069.94) | 4.86 (3.84 to 5.64) |
| Saint Lucia | 3131 (2159 to 4484) | 2133 (1472 to 3025) | -31.86 (-32.49 to -31.38) | 4688.39 (3233.64 to 6715.03) | 5084.13 (3507.87 to 7209.68) | 8.44 (7.44 to 9.21) |
| Saint Vincent and the Grenadines | 2621 (1808 to 3753) | 1702 (1174 to 2428) | -35.06 (-35.28 to -34.9) | 4876.4 (3362.97 to 6981.06) | 5050.05 (3484.07 to 7204.14) | 3.56 (3.22 to 3.82) |
| Suriname | 8126 (5606 to 11616) | 9399 (6481 to 13429) | 15.67 (15.48 to 15.84) | 4752.2 (3278.75 to 6793.49) | 4950.56 (3413.93 to 7073.37) | 4.17 (4.01 to 4.33) |
| Trinidad and Tobago | 24665 (17006 to 35416) | 18191 (12548 to 25985) | -26.25 (-26.61 to -25.97) | 4748.15 (3273.74 to 6817.58) | 4997.86 (3447.38 to 7139.13) | 5.26 (4.74 to 5.66) |
| United States Virgin Islands | 1971 (1360 to 2819) | 923 (636 to 1315) | -53.2 (-53.35 to -53.08) | 4725.91 (3259.38 to 6758.67) | 5063.68 (3493.2 to 7218.1) | 7.15 (6.81 to 7.42) |
| Central Latin America | 1841484 (1253201 to 2650234) | 2015032 (1374784 to 2898430) | 9.42 (6.47 to 12.4) | 2228.56 (1516.62 to 3207.3) | 2362.7 (1611.99 to 3398.52) | 6.02 (3.16 to 8.9) |
| Colombia | 370933 (251177 to 531892) | 373319 (266615 to 520999) | 0.64 (-12.09 to 14.07) | 2464.21 (1668.64 to 3533.5) | 2564.11 (1831.22 to 3578.43) | 4.05 (-9.11 to 17.94) |
| Costa Rica | 34755 (23680 to 49807) | 36254 (24789 to 51764) | 4.31 (3.58 to 4.86) | 2450.72 (1669.78 to 3512.08) | 2648.22 (1810.76 to 3781.24) | 8.06 (7.3 to 8.62) |
| El Salvador | 68050 (46407 to 97391) | 61075 (41705 to 87334) | -10.25 (-10.51 to -10.05) | 2469.71 (1684.21 to 3534.57) | 2563.94 (1750.8 to 3666.31) | 3.82 (3.52 to 4.05) |
| Guatemala | 115020 (78155 to 165258) | 171952 (117565 to 245487) | 49.5 (47.63 to 50.96) | 2342.19 (1591.51 to 3365.23) | 2599.46 (1777.27 to 3711.1) | 10.98 (9.6 to 12.07) |
| Honduras | 64876 (44116 to 93159) | 111434 (76174 to 159138) | 71.76 (70.04 to 73.07) | 2390.78 (1625.76 to 3433.03) | 2561.4 (1750.93 to 3657.92) | 7.14 (6.06 to 7.95) |
| Mexico | 752609 (509477 to 1103899) | 801033 (544716 to 1172423) | 6.43 (5.83 to 7.11) | 1735.43 (1174.8 to 2545.47) | 1856.36 (1262.35 to 2717.03) | 6.97 (6.36 to 7.65) |
| Nicaragua | 54174 (36853 to 77736) | 66950 (45727 to 95716) | 23.58 (22.57 to 24.38) | 2407.43 (1637.72 to 3454.53) | 2575.75 (1759.23 to 3682.42) | 6.99 (6.11 to 7.68) |
| Panama | 27467 (18755 to 39265) | 38993 (26620 to 55774) | 41.96 (41.85 to 42.07) | 2526.78 (1725.33 to 3612.09) | 2587.08 (1766.14 to 3700.43) | 2.39 (2.31 to 2.46) |
| Venezuela (Bolivarian Republic of) | 353601 (238865 to 513383) | 354021 (239526 to 514218) | 0.12 (-0.2 to 0.35) | 3895.97 (2631.81 to 5656.45) | 4045.13 (2736.88 to 5875.57) | 3.83 (3.49 to 4.07) |
| Tropical Latin America | 2334728 (1605921 to 3437608) | 2227234 (1520258 to 3221298) | -4.6 (-13.06 to 5.14) | 3370.88 (2318.63 to 4963.22) | 3344.82 (2283.1 to 4837.69) | -0.77 (-9.56 to 9.36) |
| Brazil | 2271118 (1562983 to 3353965) | 2138424 (1455231 to 3097721) | -5.84 (-14.53 to 4.15) | 3380.01 (2326.12 to 4991.57) | 3345.63 (2276.76 to 4846.49) | -1.02 (-10.15 to 9.49) |
| Paraguay | 63610 (43788 to 91493) | 88810 (61532 to 127216) | 39.62 (38.35 to 40.48) | 3074.3 (2116.26 to 4421.88) | 3325.39 (2303.99 to 4763.44) | 8.17 (7.19 to 8.84) |
| North Africa and Middle East | 3453559 (2383286 to 4967004) | 4394111 (3031049 to 6359998) | 27.23 (21.95 to 32.82) | 1953.68 (1348.22 to 2809.83) | 1858.04 (1281.67 to 2689.31) | -4.9 (-8.84 to -0.72) |
| Afghanistan | 80455 (54775 to 116487) | 258788 (176289 to 376817) | 221.66 (220.07 to 223.65) | 1443.66 (982.87 to 2090.22) | 1455.77 (991.68 to 2119.72) | 0.84 (0.34 to 1.46) |
| Algeria | 204119 (139035 to 297109) | 246040 (167639 to 358976) | 20.54 (20.27 to 20.87) | 1510.14 (1028.63 to 2198.11) | 1495.79 (1019.15 to 2182.38) | -0.95 (-1.17 to -0.68) |
| Bahrain | 2897 (1974 to 4230) | 6563 (4475 to 9509) | 126.58 (123.05 to 129.52) | 1447.57 (986.7 to 2114.11) | 1626.44 (1108.92 to 2356.48) | 12.36 (10.61 to 13.81) |
| Egypt | 409375 (278757 to 595415) | 705497 (480496 to 1027157) | 72.34 (72.19 to 72.52) | 1466.1 (998.32 to 2132.37) | 1515.56 (1032.21 to 2206.55) | 3.37 (3.29 to 3.48) |
| Iran (Islamic Republic of) | 1187774 (816220 to 1711295) | 1051097 (732248 to 1500399) | -11.51 (-20.46 to -2.33) | 3769.57 (2590.39 to 5431.04) | 4065.42 (2832.18 to 5803.22) | 7.85 (-3.07 to 19.03) |
| Iraq | 164885 (112816 to 243761) | 308271 (214044 to 446916) | 86.96 (64.35 to 114.8) | 1601.06 (1095.46 to 2366.95) | 1747.87 (1213.61 to 2533.98) | 9.17 (-4.03 to 25.43) |
| Jordan | 31468 (21424 to 45686) | 80342 (54724 to 116396) | 155.31 (153.85 to 156.36) | 1500.89 (1021.84 to 2179) | 1631.29 (1111.12 to 2363.34) | 8.69 (8.07 to 9.14) |
| Kuwait | 10037 (6839 to 14632) | 17406 (11846 to 25273) | 73.42 (72.27 to 74.29) | 1463.2 (997.02 to 2133.04) | 1588.88 (1081.4 to 2307.06) | 8.59 (7.87 to 9.14) |
| Lebanon | 20027 (13635 to 29095) | 26641 (18130 to 38690) | 33.02 (32.87 to 33.16) | 1495.51 (1018.21 to 2172.64) | 1598.85 (1088.09 to 2321.99) | 6.91 (6.78 to 7.02) |
| Libya | 34652 (23589 to 50268) | 34718 (23665 to 50308) | 0.19 (-0.83 to 1.01) | 1501.2 (1021.91 to 2177.74) | 1663.12 (1133.61 to 2409.95) | 10.79 (9.65 to 11.69) |
| Morocco | 185286 (126159 to 268937) | 202061 (137554 to 292655) | 9.05 (8.62 to 9.41) | 1484.68 (1010.9 to 2154.97) | 1564.47 (1065.02 to 2265.89) | 5.37 (4.95 to 5.72) |
| Oman | 14259 (9720 to 20876) | 22243 (15163 to 32519) | 55.99 (55.76 to 56.24) | 1422.66 (969.74 to 2082.86) | 1490.96 (1016.38 to 2179.81) | 4.8 (4.65 to 4.97) |
| Palestine | 16885 (11506 to 24616) | 37556 (25564 to 54535) | 122.43 (121.36 to 123.21) | 1414.63 (963.97 to 2062.34) | 1559.73 (1061.72 to 2264.9) | 10.26 (9.73 to 10.65) |
| Qatar | 2182 (1486 to 3182) | 8661 (5902 to 12659) | 297 (295.23 to 298.97) | 1425.09 (970.73 to 2078.85) | 1450.45 (988.45 to 2120.17) | 1.78 (1.33 to 2.28) |
| Saudi Arabia | 108434 (73855 to 159796) | 145848 (99460 to 214293) | 34.5 (30.65 to 38.22) | 1320.52 (899.42 to 1946.03) | 1439.28 (981.51 to 2114.72) | 8.99 (5.87 to 12) |
| Sudan | 158789 (108171 to 231302) | 330827 (225188 to 480452) | 108.34 (107.41 to 109.03) | 1437.51 (979.27 to 2093.96) | 1547.72 (1053.51 to 2247.72) | 7.67 (7.18 to 8.02) |
| Syrian Arab Republic | 108756 (74090 to 158513) | 92238 (62779 to 133069) | -15.19 (-17.06 to -13.77) | 1479.28 (1007.76 to 2156.08) | 1695.53 (1154 to 2446.08) | 14.62 (12.09 to 16.53) |
| Tunisia | 61100 (41597 to 88725) | 56647 (38560 to 82294) | -7.29 (-7.37 to -7.19) | 1531.3 (1042.51 to 2223.65) | 1579.87 (1075.43 to 2295.16) | 3.17 (3.08 to 3.28) |
| Türkiye | 554014 (381549 to 793218) | 541042 (373128 to 781079) | -2.34 (-14.62 to 12.86) | 2076.39 (1430.01 to 2972.9) | 2189.37 (1509.9 to 3160.7) | 5.44 (-7.81 to 21.86) |
| United Arab Emirates | 4274 (2903 to 6438) | 11901 (7910 to 17058) | 178.47 (145.38 to 219.18) | 604.46 (410.67 to 910.57) | 702.07 (466.63 to 1006.3) | 16.15 (2.35 to 33.13) |
| Yemen | 92004 (61245 to 138692) | 205629 (137100 to 308188) | 123.5 (121.14 to 125.21) | 1094.23 (728.41 to 1649.5) | 1176.9 (784.68 to 1763.89) | 7.55 (6.42 to 8.38) |
| South Asia | 4730362 (3123142 to 6918362) | 6406755 (4252521 to 9255394) | 35.44 (25.05 to 45.73) | 872.07 (575.77 to 1275.44) | 937.35 (622.17 to 1354.13) | 7.49 (-0.76 to 15.65) |
| Bangladesh | 645319 (436953 to 946266) | 721220 (488136 to 1053073) | 11.76 (10.69 to 12.59) | 1069.13 (723.92 to 1567.72) | 1182.54 (800.37 to 1726.66) | 10.61 (9.55 to 11.42) |
| Bhutan | 3265 (2203 to 4790) | 2520 (1706 to 3700) | -22.81 (-23.3 to -22.41) | 958.52 (646.91 to 1406.16) | 988.43 (668.96 to 1450.95) | 3.12 (2.46 to 3.65) |
| India | 3412191 (2232192 to 4976576) | 4484311 (2953124 to 6514550) | 31.42 (16.8 to 46.23) | 831.4 (543.88 to 1212.57) | 895.84 (589.95 to 1301.42) | 7.75 (-4.23 to 19.89) |
| Nepal | 92549 (62355 to 135596) | 121830 (82394 to 178770) | 31.64 (29.91 to 32.91) | 894.3 (602.53 to 1310.26) | 978.2 (661.56 to 1435.38) | 9.38 (7.95 to 10.44) |
| Pakistan | 577039 (383965 to 836221) | 1076873 (717521 to 1559204) | 86.62 (85.9 to 87.13) | 946.53 (629.83 to 1371.68) | 985.93 (656.93 to 1427.53) | 4.16 (3.76 to 4.45) |
| East Asia | 14316211 (9908736 to 20582252) | 13018254 (9117581 to 18633515) | -9.07 (-16.38 to -1.41) | 3111.27 (2153.42 to 4473.04) | 3773.94 (2643.15 to 5401.78) | 21.3 (11.54 to 31.51) |
| China | 14020452 (9712792 to 20165858) | 12789703 (8960362 to 18298692) | -8.78 (-16.3 to -0.96) | 3150.37 (2182.45 to 4531.23) | 3825.77 (2680.31 to 5473.67) | 21.44 (11.42 to 31.85) |
| Democratic People's Republic of Korea | 165879 (112301 to 238123) | 157164 (106350 to 224767) | -5.25 (-6.13 to -4.49) | 2129.57 (1441.73 to 3057.04) | 2380.45 (1610.8 to 3404.39) | 11.78 (10.75 to 12.69) |
| Taiwan (Province of China) | 129880 (86975 to 191112) | 71387 (47827 to 104956) | -45.04 (-45.39 to -44.79) | 1777 (1189.98 to 2614.77) | 1764.61 (1182.23 to 2594.41) | -0.7 (-1.33 to -0.26) |
| Southeast Asia | 3400825 (2321988 to 4859740) | 3427714 (2344277 to 4943959) | 0.79 (-4.53 to 3.6) | 1546.52 (1055.92 to 2209.97) | 1495.05 (1022.49 to 2156.38) | -3.33 (-8.43 to -0.64) |
| Cambodia | 71658 (47980 to 106113) | 91192 (61364 to 134731) | 27.26 (26.11 to 28.22) | 1258.6 (842.72 to 1863.79) | 1375.21 (925.39 to 2031.8) | 9.27 (8.28 to 10.09) |
| Indonesia | 1246600 (846327 to 1816339) | 1312755 (891627 to 1906901) | 5.31 (4.71 to 5.74) | 1425.28 (967.64 to 2076.69) | 1455.1 (988.31 to 2113.66) | 2.09 (1.52 to 2.51) |
| Lao People's Democratic Republic | 28930 (19383 to 42828) | 40179 (27069 to 59335) | 38.88 (37.66 to 39.88) | 1275.3 (854.44 to 1887.96) | 1343.09 (904.86 to 1983.45) | 5.32 (4.39 to 6.07) |
| Malaysia | 53191 (35091 to 77280) | 72059 (46891 to 107940) | 35.47 (21.23 to 52.35) | 637.55 (420.61 to 926.29) | 702.66 (457.24 to 1052.54) | 10.21 (-1.37 to 23.94) |
| Maldives | 1616 (1082 to 2394) | 1849 (1243 to 2732) | 14.37 (13.34 to 15.21) | 1259.41 (842.84 to 1865.55) | 1418.66 (954.15 to 2096.32) | 12.64 (11.64 to 13.47) |
| Mauritius | 6063 (4083 to 8952) | 4292 (2902 to 6316) | -29.2 (-30.08 to -28.53) | 1407.37 (947.63 to 2077.88) | 1445.73 (977.4 to 2127.26) | 2.73 (1.45 to 3.7) |
| Myanmar | 260223 (175161 to 384406) | 287968 (194189 to 424937) | 10.66 (10.19 to 11.03) | 1361.62 (916.53 to 2011.41) | 1386.58 (935.03 to 2046.1) | 1.83 (1.4 to 2.18) |
| Philippines | 437055 (296683 to 636725) | 646124 (438766 to 940222) | 47.84 (47.22 to 48.27) | 1363.55 (925.61 to 1986.5) | 1442.96 (979.87 to 2099.75) | 5.82 (5.38 to 6.14) |
| Seychelles | 430 (290 to 634) | 421 (284 to 622) | -1.9 (-2.02 to -1.76) | 1375.31 (927.1 to 2030.24) | 1380.7 (929.94 to 2038.99) | 0.39 (0.27 to 0.53) |
| Sri Lanka | 101445 (68319 to 149817) | 98731 (66589 to 145609) | -2.68 (-3.12 to -2.34) | 1401.15 (943.62 to 2069.26) | 1430.89 (965.07 to 2110.3) | 2.12 (1.66 to 2.47) |
| Thailand | 732732 (509428 to 978738) | 417851 (291385 to 603469) | -42.97 (-54.39 to -33.43) | 3221.25 (2239.56 to 4302.74) | 3061.59 (2134.98 to 4421.61) | -4.96 (-23.98 to 10.95) |
| Timor-Leste | 5045 (3375 to 7476) | 9357 (6307 to 13814) | 85.46 (83.35 to 86.99) | 1240.61 (829.91 to 1838.29) | 1365.1 (920.12 to 2015.33) | 10.03 (8.78 to 10.94) |
| Viet Nam | 450918 (302808 to 666874) | 440155 (295400 to 651098) | -2.39 (-2.58 to -2.22) | 1339.35 (899.42 to 1980.8) | 1392.06 (934.25 to 2059.2) | 3.94 (3.73 to 4.11) |
| Oceania | 57188 (39398 to 83519) | 108036 (74443 to 157839) | 88.92 (88.79 to 89.04) | 1698.78 (1170.33 to 2480.96) | 1691.67 (1165.65 to 2471.49) | -0.42 (-0.49 to -0.35) |
| American Samoa | 396 (273 to 578) | 370 (255 to 542) | -6.39 (-7.05 to -5.95) | 1653.17 (1139.6 to 2415.65) | 1935.66 (1333.5 to 2832.97) | 17.09 (16.27 to 17.65) |
| Cook Islands | 153 (106 to 224) | 96 (66 to 141) | -37.24 (-37.59 to -36.97) | 1782.68 (1229.58 to 2607.2) | 1858.71 (1280.28 to 2718.98) | 4.26 (3.68 to 4.72) |
| Fiji | 6335 (4365 to 9247) | 6216 (4285 to 9077) | -1.88 (-2.07 to -1.76) | 1766.09 (1216.99 to 2577.94) | 1772.71 (1222.12 to 2588.82) | 0.38 (0.19 to 0.5) |
| Guam | 913 (630 to 1335) | 859 (592 to 1257) | -5.88 (-6.07 to -5.75) | 1685.32 (1163.22 to 2465.51) | 1771.31 (1221.15 to 2592.28) | 5.1 (4.89 to 5.25) |
| Kiribati | 596 (411 to 869) | 936 (645 to 1366) | 57.07 (56.65 to 57.37) | 1626.49 (1120.71 to 2371.99) | 1752.1 (1207.2 to 2556.54) | 7.72 (7.43 to 7.93) |
| Marshall Islands | 464 (319 to 676) | 415 (286 to 607) | -10.45 (-11.68 to -9.57) | 1732.27 (1190.32 to 2522.66) | 1800.63 (1241.3 to 2632.41) | 3.95 (2.53 to 4.97) |
| Micronesia (Federated States of) | 1009 (694 to 1471) | 766 (528 to 1122) | -24.03 (-24.97 to -23.36) | 1754.14 (1207.37 to 2559.22) | 1848.71 (1274.29 to 2706.83) | 5.39 (4.09 to 6.33) |
| Nauru | 86 (59 to 125) | 90 (62 to 131) | 5.11 (4.35 to 5.65) | 1641.61 (1130.39 to 2392.31) | 1752.71 (1208.69 to 2561.87) | 6.77 (6 to 7.32) |
| Niue | 18 (13 to 27) | 10 (7 to 15) | -46.01 (-46.7 to -45.54) | 1807.81 (1245.33 to 2640.49) | 1889.47 (1302.78 to 2772) | 4.52 (3.18 to 5.44) |
| Northern Mariana Islands | 266 (183 to 388) | 285 (196 to 417) | 7.15 (6.43 to 8.06) | 1640.59 (1130.69 to 2394.1) | 1900.91 (1309.39 to 2782.03) | 15.87 (15.09 to 16.85) |
| Palau | 112 (77 to 164) | 84 (58 to 122) | -25.54 (-25.6 to -25.47) | 1819.85 (1254.79 to 2664.91) | 1886.48 (1299.82 to 2762.3) | 3.66 (3.58 to 3.77) |
| Papua New Guinea | 35945 (24760 to 52500) | 82158 (56603 to 120032) | 128.57 (128.45 to 128.68) | 1688.62 (1163.19 to 2466.36) | 1677.02 (1155.39 to 2450.11) | -0.69 (-0.74 to -0.64) |
| Samoa | 1617 (1115 to 2367) | 1726 (1190 to 2520) | 6.73 (6.34 to 7.23) | 1765.78 (1217.26 to 2584.72) | 1710.09 (1178.45 to 2496.73) | -3.15 (-3.51 to -2.7) |
| Solomon Islands | 3263 (2248 to 4764) | 5692 (3924 to 8317) | 74.44 (74 to 74.75) | 1676.65 (1154.93 to 2447.64) | 1720.98 (1186.3 to 2514.77) | 2.64 (2.39 to 2.83) |
| Tokelau | 13 (9 to 20) | 10 (7 to 15) | -25.69 (-26.14 to -25.39) | 1771.67 (1220.69 to 2585.05) | 1951.65 (1344.25 to 2854.71) | 10.16 (9.5 to 10.61) |
| Tonga | 921 (635 to 1346) | 850 (586 to 1242) | -7.73 (-7.82 to -7.59) | 1726.1 (1189.96 to 2522.67) | 1714.06 (1181.55 to 2504.27) | -0.7 (-0.8 to -0.55) |
| Tuvalu | 67 (46 to 98) | 88 (61 to 129) | 31.32 (29.95 to 32.26) | 1569.57 (1081.21 to 2290.13) | 1792.72 (1236.86 to 2625.57) | 14.22 (13.03 to 15.04) |
| Vanuatu | 1365 (940 to 1991) | 2534 (1746 to 3700) | 85.6 (84.76 to 86.21) | 1635.92 (1125.85 to 2385.59) | 1718.51 (1184.3 to 2509.21) | 5.05 (4.57 to 5.39) |
| Central sub-Saharan Africa | 229546 (153911 to 338148) | 584196 (391757 to 860482) | 154.5 (153.97 to 154.87) | 740.75 (496.68 to 1091.21) | 794.17 (532.56 to 1169.76) | 7.21 (6.99 to 7.37) |
| Angola | 42522 (28508 to 62645) | 144004 (96463 to 212278) | 238.66 (237.98 to 239.43) | 737.45 (494.42 to 1086.46) | 771.08 (516.52 to 1136.65) | 4.56 (4.35 to 4.8) |
| Central African Republic | 11068 (7420 to 16303) | 22839 (15323 to 33632) | 106.35 (105.36 to 107.03) | 739.27 (495.64 to 1088.91) | 789.19 (529.49 to 1162.12) | 6.75 (6.24 to 7.1) |
| Congo | 10313 (6921 to 15190) | 20547 (13779 to 30258) | 99.24 (98.85 to 99.53) | 777.33 (521.67 to 1144.98) | 826.23 (554.07 to 1216.73) | 6.29 (6.08 to 6.45) |
| Democratic Republic of the Congo | 160002 (107278 to 235699) | 383285 (257097 to 564425) | 139.55 (138.75 to 140.11) | 739.04 (495.51 to 1088.67) | 800.06 (536.66 to 1178.17) | 8.26 (7.9 to 8.51) |
| Equatorial Guinea | 1759 (1179 to 2591) | 6838 (4572 to 10045) | 288.86 (283.42 to 294.06) | 732.79 (491.1 to 1079.47) | 887.94 (593.71 to 1304.35) | 21.17 (19.48 to 22.79) |
| Gabon | 3882 (2604 to 5720) | 6683 (4485 to 9840) | 72.13 (71.33 to 72.75) | 763.97 (512.5 to 1125.54) | 808 (542.26 to 1189.74) | 5.76 (5.28 to 6.14) |
| Eastern sub-Saharan Africa | 838331 (558812 to 1235096) | 1832457 (1224936 to 2689085) | 118.58 (117.31 to 119.69) | 755.95 (503.9 to 1113.73) | 805.18 (538.23 to 1181.58) | 6.51 (5.89 to 7.05) |
| Burundi | 23021 (15419 to 33935) | 56421 (37825 to 83151) | 145.08 (143.9 to 145.91) | 729.65 (488.68 to 1075.55) | 775.6 (519.97 to 1143.04) | 6.3 (5.78 to 6.66) |
| Comoros | 2030 (1361 to 2990) | 2590 (1736 to 3811) | 27.62 (26.81 to 28.21) | 767.92 (514.95 to 1131.07) | 825.12 (553.03 to 1213.79) | 7.45 (6.76 to 7.94) |
| Djibouti | 1814 (1215 to 2668) | 4419 (2960 to 6498) | 143.6 (142.81 to 144.47) | 808.9 (541.88 to 1189.69) | 836.41 (560.14 to 1229.77) | 3.4 (3.07 to 3.77) |
| Eritrea | 15182 (10176 to 22353) | 25841 (17328 to 38027) | 70.21 (69.48 to 70.72) | 771.93 (517.39 to 1136.51) | 803.35 (538.71 to 1182.2) | 4.07 (3.62 to 4.39) |
| Ethiopia | 225384 (148955 to 331631) | 467612 (310186 to 685173) | 107.47 (105.62 to 108.8) | 764.86 (505.49 to 1125.42) | 817.53 (542.3 to 1197.9) | 6.89 (5.93 to 7.57) |
| Kenya | 106694 (70508 to 156916) | 210500 (139756 to 308107) | 97.29 (95.85 to 98.34) | 773.6 (511.23 to 1137.74) | 855.67 (568.1 to 1252.44) | 10.61 (9.8 to 11.2) |
| Madagascar | 50845 (34088 to 74904) | 120036 (80545 to 176785) | 136.08 (135.17 to 136.72) | 755.95 (506.8 to 1113.65) | 803.25 (538.99 to 1183) | 6.26 (5.85 to 6.55) |
| Malawi | 40774 (27341 to 60074) | 86853 (58229 to 127742) | 113.01 (111.32 to 114.15) | 731.59 (490.57 to 1077.89) | 820.08 (549.8 to 1206.15) | 12.09 (11.2 to 12.7) |
| Mozambique | 56902 (38111 to 83884) | 138495 (92846 to 204086) | 143.39 (142.78 to 143.85) | 751.61 (503.4 to 1108.01) | 780.07 (522.95 to 1149.51) | 3.79 (3.53 to 3.98) |
| Rwanda | 30783 (20632 to 45369) | 51933 (34827 to 76443) | 68.71 (67.19 to 69.79) | 743.8 (498.51 to 1096.23) | 806.66 (540.95 to 1187.35) | 8.45 (7.48 to 9.15) |
| Somalia | 36754 (24595 to 54113) | 97732 (65547 to 143851) | 165.91 (164.76 to 166.85) | 776.66 (519.72 to 1143.46) | 764.46 (512.71 to 1125.21) | -1.57 (-2 to -1.22) |
| South Sudan | 25717 (17246 to 37847) | 44416 (29760 to 65365) | 72.71 (72.22 to 73.07) | 782.61 (524.82 to 1151.73) | 809.43 (542.34 to 1191.2) | 3.43 (3.13 to 3.64) |
| Uganda | 74471 (49937 to 109718) | 197279 (132322 to 290475) | 164.91 (164.01 to 165.71) | 723.17 (484.93 to 1065.45) | 790.9 (530.48 to 1164.53) | 9.37 (8.99 to 9.7) |
| United Republic of Tanzania | 112064 (75138 to 165113) | 243513 (163397 to 358663) | 117.3 (116.74 to 117.7) | 750.94 (503.5 to 1106.42) | 789.13 (529.51 to 1162.29) | 5.09 (4.82 to 5.28) |
| Zambia | 35295 (23689 to 51992) | 83221 (55830 to 122588) | 135.78 (135.57 to 136.03) | 750.89 (503.97 to 1106.09) | 796.05 (534.04 to 1172.61) | 6.01 (5.92 to 6.13) |
| Southern sub-Saharan Africa | 211166 (140903 to 312415) | 260254 (173740 to 384849) | 23.25 (22.83 to 23.7) | 798.04 (532.5 to 1180.68) | 832.43 (555.71 to 1230.95) | 4.31 (3.96 to 4.7) |
| Botswana | 5806 (3893 to 8556) | 7578 (5079 to 11141) | 30.53 (29.65 to 31.27) | 784.06 (525.8 to 1155.47) | 826.41 (553.85 to 1214.97) | 5.4 (4.69 to 6) |
| Eswatini | 3661 (2454 to 5396) | 4430 (2969 to 6518) | 21 (20.11 to 21.76) | 767.32 (514.34 to 1130.84) | 827.99 (554.88 to 1218.35) | 7.91 (7.11 to 8.59) |
| Lesotho | 6526 (4369 to 9622) | 7045 (4720 to 10345) | 7.95 (6.61 to 8.98) | 779.36 (521.74 to 1149.14) | 839.83 (562.67 to 1233.31) | 7.76 (6.42 to 8.78) |
| Namibia | 5924 (3975 to 8723) | 8773 (5882 to 12912) | 48.1 (47.74 to 48.36) | 777.71 (521.87 to 1145.28) | 818.86 (549.05 to 1205.12) | 5.29 (5.04 to 5.47) |
| South Africa | 142156 (94262 to 208331) | 168511 (111832 to 246752) | 18.54 (18.29 to 18.79) | 806.71 (534.92 to 1182.24) | 846.02 (561.46 to 1238.84) | 4.87 (4.66 to 5.09) |
| Zimbabwe | 47094 (31583 to 69385) | 63917 (42885 to 94146) | 35.72 (35.46 to 35.91) | 781.98 (524.42 to 1152.13) | 800.55 (537.12 to 1179.15) | 2.37 (2.18 to 2.51) |
| Western sub-Saharan Africa | 823000 (547750 to 1239291) | 2215071 (1473274 to 3266019) | 169.15 (154.26 to 183.55) | 765.62 (509.56 to 1152.89) | 824.75 (548.55 to 1216.05) | 7.72 (1.77 to 13.49) |
| Benin | 21684 (14257 to 32095) | 59037 (38898 to 87286) | 172.26 (169.98 to 173.86) | 755.62 (496.82 to 1118.41) | 785.45 (517.51 to 1161.29) | 3.95 (3.08 to 4.56) |
| Burkina Faso | 43358 (28539 to 64171) | 98862 (65141 to 146179) | 128.02 (126.76 to 128.98) | 766.41 (504.47 to 1134.32) | 772.01 (508.68 to 1141.5) | 0.73 (0.18 to 1.16) |
| Cabo Verde | 1538 (1014 to 2274) | 1696 (1116 to 2508) | 10.23 (8.93 to 11.13) | 791.11 (521.26 to 1169.58) | 879.82 (579.02 to 1301.25) | 11.21 (9.91 to 12.13) |
| Cameroon | 45104 (29713 to 66701) | 137957 (90875 to 203941) | 205.87 (204.55 to 206.77) | 759.69 (500.46 to 1123.46) | 816.18 (537.63 to 1206.55) | 7.43 (6.97 to 7.75) |
| Chad | 25991 (17117 to 38445) | 83569 (55051 to 123593) | 221.53 (221.17 to 221.84) | 737.27 (485.55 to 1090.56) | 763.21 (502.77 to 1128.74) | 3.52 (3.4 to 3.62) |
| Côte d'Ivoire | 52798 (34773 to 78074) | 116161 (76510 to 171714) | 120.01 (119.03 to 120.79) | 763.14 (502.61 to 1128.48) | 807.53 (531.89 to 1193.73) | 5.82 (5.35 to 6.19) |
| Gambia | 4284 (2822 to 6336) | 10342 (6812 to 15283) | 141.42 (139.79 to 142.54) | 762.54 (502.4 to 1127.77) | 816.53 (537.79 to 1206.62) | 7.08 (6.36 to 7.57) |
| Ghana | 64639 (42586 to 95565) | 133822 (88135 to 197751) | 107.03 (106.21 to 107.59) | 782.59 (515.59 to 1157.02) | 820.29 (540.24 to 1212.16) | 4.82 (4.4 to 5.1) |
| Guinea | 24425 (16075 to 36137) | 59512 (39214 to 88013) | 143.65 (142.51 to 144.4) | 743.03 (489.02 to 1099.32) | 797.27 (525.34 to 1179.1) | 7.3 (6.8 to 7.63) |
| Guinea-Bissau | 4547 (2996 to 6726) | 8988 (5922 to 13289) | 97.68 (97.02 to 98.19) | 773.16 (509.38 to 1143.66) | 803.59 (529.41 to 1188.06) | 3.94 (3.59 to 4.21) |
| Liberia | 10277 (6767 to 15199) | 23203 (15280 to 34292) | 125.78 (123.87 to 127.14) | 752.22 (495.32 to 1112.55) | 831.62 (547.68 to 1229.09) | 10.56 (9.62 to 11.22) |
| Mali | 36922 (24306 to 54624) | 110637 (72894 to 163570) | 199.65 (198.41 to 200.56) | 746.52 (491.43 to 1104.44) | 777.33 (512.14 to 1149.22) | 4.13 (3.69 to 4.44) |
| Mauritania | 8734 (5755 to 12912) | 19076 (12567 to 28201) | 118.41 (117.74 to 118.84) | 770.26 (507.51 to 1138.73) | 817.95 (538.85 to 1209.21) | 6.19 (5.86 to 6.4) |
| Niger | 35848 (23589 to 53045) | 119853 (78967 to 177237) | 234.34 (232.97 to 235.4) | 738.89 (486.22 to 1093.36) | 768.94 (506.63 to 1137.1) | 4.07 (3.64 to 4.4) |
| Nigeria | 375432 (252602 to 560413) | 1092688 (716446 to 1605444) | 191.05 (158.65 to 223.89) | 773.78 (520.62 to 1155.04) | 855.18 (560.72 to 1256.49) | 10.52 (-1.78 to 22.99) |
| Sao Tome and Principe | 566 (373 to 837) | 876 (577 to 1294) | 54.77 (53.48 to 55.65) | 809.54 (533.44 to 1197.49) | 860.07 (566.31 to 1271.47) | 6.24 (5.36 to 6.85) |
| Senegal | 33707 (22208 to 49861) | 67731 (44595 to 100080) | 100.94 (99.21 to 102.35) | 757.99 (499.39 to 1121.24) | 834.61 (549.52 to 1233.22) | 10.11 (9.16 to 10.88) |
| Sierra Leone | 16343 (10768 to 24161) | 36411 (23980 to 53793) | 122.79 (121.36 to 123.74) | 740.94 (488.21 to 1095.39) | 800.12 (526.96 to 1182.09) | 7.99 (7.29 to 8.45) |
| Togo | 16777 (11054 to 24811) | 34626 (22805 to 51179) | 106.39 (105.35 to 107.19) | 779.35 (513.51 to 1152.55) | 829.28 (546.17 to 1225.73) | 6.41 (5.87 to 6.82) |

Abbreviations: UI, uncertainty interval.

**eTable** **4b. Incidence of ADHD by country, <20 years, 1990-2021.**

|  | **Incidence of ADHD** | | | | | |
| --- | --- | --- | --- | --- | --- | --- |
|  | **Counts (<20 years)** | | | **Rates per 100,000 persons (<20 years)** | | |
| **Country** | **1990 (95% UI)** | **2021 (95% UI)** | **Percentage change from 1990 to 2021 (%)** | **1990 (95% UI)** | **2021 (95% UI)** | **Percentage change from 1990 to 2021 (%)** |
| Global | 3740573 (2543085 to 5471097) | 4111621 (2775203 to 5954941) | 9.92 (7.13 to 13.16) | 165.62 (112.6 to 242.24) | 155.99 (105.29 to 225.92) | -5.81 (-8.21 to -3.04) |
| Central Europe | 52533 (35249 to 77759) | 31546 (21175 to 46692) | -39.95 (-40.01 to -39.9) | 133.78 (89.76 to 198.02) | 133.91 (89.89 to 198.21) | 0.1 (0 to 0.18) |
| Albania | 2073 (1388 to 3091) | 800 (536 to 1193) | -61.38 (-61.41 to -61.35) | 143.35 (95.99 to 213.79) | 129.7 (86.86 to 193.36) | -9.52 (-9.58 to -9.45) |
| Bosnia and Herzegovina | 1974 (1322 to 2941) | 883 (591 to 1315) | -55.29 (-55.32 to -55.27) | 133.23 (89.21 to 198.5) | 132.71 (88.87 to 197.77) | -0.39 (-0.45 to -0.34) |
| Bulgaria | 3023 (2024 to 4504) | 1728 (1157 to 2575) | -42.83 (-42.85 to -42.81) | 127.92 (85.66 to 190.6) | 133.54 (89.43 to 198.99) | 4.39 (4.35 to 4.42) |
| Croatia | 1775 (1188 to 2644) | 1049 (702 to 1563) | -40.9 (-40.9 to -40.9) | 133.79 (89.6 to 199.36) | 130.18 (87.18 to 193.97) | -2.7 (-2.7 to -2.7) |
| Czechia | 3716 (2488 to 5536) | 3048 (2041 to 4541) | -17.97 (-17.99 to -17.96) | 122.08 (81.75 to 181.87) | 137.26 (91.9 to 204.51) | 12.44 (12.42 to 12.46) |
| Hungary | 3533 (2366 to 5264) | 2471 (1654 to 3681) | -30.08 (-30.11 to -30.05) | 121.75 (81.53 to 181.37) | 131.74 (88.21 to 196.29) | 8.21 (8.16 to 8.25) |
| Montenegro | 293 (196 to 437) | 200 (134 to 298) | -31.77 (-31.86 to -31.69) | 136.65 (91.5 to 203.6) | 133 (89.07 to 198.25) | -2.67 (-2.8 to -2.56) |
| North Macedonia | 953 (638 to 1420) | 595 (398 to 887) | -37.59 (-37.62 to -37.57) | 136.77 (91.59 to 203.81) | 133.21 (89.21 to 198.52) | -2.6 (-2.64 to -2.57) |
| Poland | 17906 (11782 to 26813) | 10681 (7027 to 15997) | -40.35 (-40.38 to -40.33) | 143.83 (94.64 to 215.37) | 138.74 (91.27 to 207.8) | -3.54 (-3.59 to -3.5) |
| Romania | 9626 (6445 to 14341) | 5282 (3537 to 7870) | -45.13 (-45.16 to -45.1) | 128.3 (85.9 to 191.14) | 130.72 (87.53 to 194.77) | 1.88 (1.82 to 1.93) |
| Serbia | 3736 (2500 to 5558) | 2264 (1517 to 3376) | -39.4 (-39.93 to -38.95) | 128.78 (86.19 to 191.57) | 120.82 (80.94 to 180.15) | -6.18 (-7 to -5.48) |
| Slovakia | 2349 (1573 to 3500) | 1522 (1019 to 2268) | -35.21 (-35.23 to -35.19) | 133.86 (89.63 to 199.41) | 135.78 (90.91 to 202.32) | 1.44 (1.4 to 1.48) |
| Slovenia | 735 (492 to 1096) | 564 (378 to 841) | -23.24 (-23.27 to -23.21) | 132.06 (88.44 to 196.76) | 139.42 (93.37 to 207.76) | 5.57 (5.53 to 5.61) |
| Eastern Europe | 96689 (63795 to 144916) | 66512 (43875 to 99892) | -31.21 (-31.41 to -31.03) | 143.72 (94.83 to 215.41) | 144.09 (95.05 to 216.41) | 0.26 (-0.04 to 0.53) |
| Belarus | 4466 (2990 to 6654) | 2963 (1985 to 4415) | -33.66 (-33.71 to -33.61) | 142.33 (95.29 to 212.05) | 146.31 (97.99 to 218.01) | 2.8 (2.71 to 2.87) |
| Estonia | 637 (426 to 949) | 383 (256 to 570) | -39.92 (-39.97 to -39.88) | 138.67 (92.84 to 206.59) | 136.52 (91.41 to 203.41) | -1.55 (-1.63 to -1.49) |
| Latvia | 1057 (708 to 1575) | 547 (366 to 815) | -48.24 (-48.31 to -48.19) | 140.51 (94.06 to 209.34) | 141.36 (94.66 to 210.66) | 0.6 (0.48 to 0.71) |
| Lithuania | 1513 (1013 to 2254) | 753 (504 to 1122) | -50.22 (-50.25 to -50.2) | 136.58 (91.44 to 203.48) | 140.55 (94.11 to 209.42) | 2.91 (2.84 to 2.96) |
| Republic of Moldova | 2285 (1530 to 3404) | 941 (630 to 1403) | -58.8 (-58.85 to -58.77) | 144.5 (96.74 to 215.27) | 135.2 (90.55 to 201.45) | -6.44 (-6.54 to -6.35) |
| Russian Federation | 65539 (43117 to 98162) | 49384 (32498 to 73943) | -24.65 (-24.7 to -24.6) | 145.05 (95.42 to 217.24) | 146.15 (96.18 to 218.83) | 0.76 (0.69 to 0.83) |
| Ukraine | 21191 (13941 to 31739) | 11541 (7597 to 17279) | -45.54 (-45.58 to -45.49) | 140.79 (92.62 to 210.86) | 136.66 (89.95 to 204.6) | -2.93 (-3.02 to -2.84) |
| Central Asia | 45815 (30666 to 68254) | 51366 (34393 to 76565) | 12.12 (11.9 to 12.29) | 145.08 (97.11 to 216.13) | 148.35 (99.33 to 221.13) | 2.26 (2.06 to 2.42) |
| Armenia | 1942 (1300 to 2894) | 1100 (737 to 1641) | -43.36 (-43.52 to -43.22) | 146.27 (97.92 to 217.96) | 144.16 (96.57 to 215) | -1.44 (-1.73 to -1.21) |
| Azerbaijan | 4496 (3010 to 6701) | 4422 (2963 to 6595) | -1.64 (-1.99 to -1.36) | 143.64 (96.16 to 214.07) | 145.15 (97.24 to 216.44) | 1.05 (0.69 to 1.34) |
| Georgia | 2500 (1674 to 3725) | 1391 (932 to 2074) | -44.36 (-44.48 to -44.27) | 139.16 (93.16 to 207.32) | 149.71 (100.26 to 223.18) | 7.58 (7.35 to 7.76) |
| Kazakhstan | 9443 (6321 to 14067) | 9916 (6639 to 14777) | 5.01 (4.87 to 5.12) | 142.15 (95.15 to 211.76) | 147.47 (98.74 to 219.76) | 3.74 (3.61 to 3.85) |
| Kyrgyzstan | 3077 (2059 to 4583) | 4255 (2849 to 6339) | 38.29 (38.12 to 38.42) | 145.72 (97.53 to 217.07) | 150.72 (100.91 to 224.58) | 3.43 (3.31 to 3.54) |
| Mongolia | 1626 (1088 to 2422) | 2084 (1395 to 3105) | 28.19 (28.02 to 28.33) | 143.22 (95.86 to 213.35) | 158.76 (106.29 to 236.57) | 10.85 (10.7 to 10.97) |
| Tajikistan | 4264 (2854 to 6352) | 6650 (4453 to 9912) | 55.98 (55.7 to 56.2) | 148.33 (99.27 to 220.98) | 149.28 (99.94 to 222.49) | 0.64 (0.46 to 0.78) |
| Turkmenistan | 2743 (1836 to 4086) | 2795 (1871 to 4165) | 1.91 (1.77 to 2.03) | 145.47 (97.37 to 216.72) | 142.56 (95.45 to 212.44) | -2 (-2.14 to -1.89) |
| Uzbekistan | 15724 (10524 to 23425) | 18752 (12555 to 27955) | 19.26 (18.97 to 19.49) | 147.29 (98.58 to 219.42) | 148.71 (99.56 to 221.69) | 0.96 (0.72 to 1.16) |
| Australasia | 26678 (18342 to 38230) | 34767 (24322 to 48285) | 30.32 (13.96 to 53.27) | 425.27 (292.39 to 609.42) | 460.99 (322.5 to 640.23) | 8.4 (-5.21 to 27.49) |
| Australia | 23405 (16211 to 33409) | 30318 (21223 to 40943) | 29.54 (10.59 to 55.94) | 452.52 (313.43 to 645.94) | 486 (340.2 to 656.32) | 7.4 (-8.31 to 29.29) |
| New Zealand | 3273 (2146 to 4894) | 4449 (2957 to 6694) | 35.93 (20.38 to 59.77) | 297.25 (194.91 to 444.5) | 341.31 (226.88 to 513.56) | 14.82 (1.69 to 34.96) |
| High-income Asia Pacific | 88200 (58368 to 131104) | 56470 (38086 to 83674) | -35.97 (-38.48 to -32.59) | 175.25 (115.98 to 260.51) | 183.41 (123.7 to 271.77) | 4.65 (0.55 to 10.19) |
| Brunei Darussalam | 240 (157 to 359) | 244 (160 to 365) | 1.87 (1.81 to 1.93) | 208.43 (136.08 to 311.93) | 189.13 (123.54 to 282.89) | -9.26 (-9.31 to -9.21) |
| Japan | 57223 (38056 to 84412) | 39142 (26030 to 57736) | -31.6 (-31.61 to -31.59) | 171.51 (114.06 to 253.01) | 184.34 (122.59 to 271.91) | 7.48 (7.46 to 7.5) |
| Republic of Korea | 29159 (19052 to 43599) | 14886 (10314 to 22668) | -48.95 (-56.1 to -38.96) | 183.08 (119.62 to 273.74) | 177.57 (123.03 to 270.4) | -3.01 (-16.59 to 15.96) |
| Singapore | 1578 (1032 to 2359) | 2198 (1436 to 3288) | 39.29 (39.17 to 39.4) | 171.37 (112.01 to 256.13) | 210.69 (137.62 to 315.1) | 22.95 (22.84 to 23.04) |
| High-income North America | 236579 (156298 to 349636) | 260879 (170399 to 390296) | 10.27 (-0.16 to 19.5) | 289.47 (191.24 to 427.8) | 291.3 (190.27 to 435.8) | 0.63 (-8.88 to 9.05) |
| Canada | 21073 (14975 to 29681) | 22258 (14530 to 33453) | 5.62 (-16.1 to 27.81) | 274.94 (195.37 to 387.25) | 270.48 (176.58 to 406.53) | -1.62 (-21.86 to 19.05) |
| Greenland | 57 (38 to 88) | 47 (31 to 71) | -18.66 (-18.7 to -18.63) | 319.78 (210.08 to 490.33) | 302.78 (198.91 to 464.15) | -5.32 (-5.36 to -5.28) |
| United States of America | 215444 (140331 to 322690) | 238570 (155745 to 357134) | 10.73 (0.18 to 21.03) | 290.96 (189.52 to 435.8) | 293.4 (191.54 to 439.21) | 0.84 (-8.77 to 10.21) |
| Southern Latin America | 38569 (25867 to 56936) | 37936 (25087 to 54948) | -1.64 (-11.94 to 8.18) | 199.02 (133.48 to 293.8) | 194.45 (128.59 to 281.64) | -2.3 (-12.53 to 7.46) |
| Argentina | 26166 (17549 to 38623) | 26688 (17633 to 39777) | 2 (-13.18 to 16.46) | 200.86 (134.71 to 296.49) | 194.64 (128.6 to 290.1) | -3.1 (-17.51 to 10.65) |
| Chile | 10318 (6920 to 15235) | 9496 (6371 to 14015) | -7.97 (-8.03 to -7.92) | 195.62 (131.19 to 288.83) | 194.04 (130.19 to 286.38) | -0.81 (-0.87 to -0.76) |
| Uruguay | 2083 (1397 to 3075) | 1750 (1174 to 2582) | -16.01 (-16.07 to -15.97) | 193.42 (129.74 to 285.53) | 193.68 (129.96 to 285.87) | 0.14 (0.07 to 0.19) |
| Western Europe | 199791 (135233 to 293277) | 203223 (135369 to 298926) | 1.72 (-4.5 to 7.43) | 203.15 (137.51 to 298.21) | 221.59 (147.6 to 325.94) | 9.08 (2.41 to 15.2) |
| Andorra | 26 (17 to 40) | 27 (17 to 41) | 3.72 (3.53 to 3.94) | 191.23 (125.24 to 293.57) | 185.66 (121.68 to 284.98) | -2.91 (-3.09 to -2.71) |
| Austria | 4519 (2940 to 6736) | 4345 (2898 to 6498) | -3.86 (-17.63 to 12.59) | 241.25 (156.93 to 359.57) | 247.58 (165.14 to 370.27) | 2.62 (-12.08 to 20.18) |
| Belgium | 5033 (3295 to 7730) | 5357 (3508 to 8226) | 6.42 (6.4 to 6.45) | 203.64 (133.32 to 312.75) | 210.54 (137.87 to 323.3) | 3.39 (3.36 to 3.41) |
| Cyprus | 565 (370 to 867) | 623 (408 to 957) | 10.36 (10.31 to 10.4) | 218.01 (142.77 to 334.73) | 218.62 (143.14 to 335.79) | 0.28 (0.24 to 0.32) |
| Denmark | 2374 (1537 to 3541) | 2477 (1586 to 3726) | 4.37 (-15.05 to 24.97) | 189.86 (122.91 to 283.24) | 191.43 (122.53 to 287.93) | 0.83 (-17.94 to 20.73) |
| Finland | 4093 (2528 to 5616) | 2938 (1993 to 4411) | -28.23 (-51.54 to -9.66) | 322.6 (199.28 to 442.63) | 255.78 (173.54 to 384.03) | -20.72 (-46.46 to -0.21) |
| France | 39789 (25612 to 60625) | 39785 (25726 to 59356) | -0.01 (-14.16 to 17.28) | 246.5 (158.67 to 375.57) | 252.58 (163.32 to 376.83) | 2.47 (-12.03 to 20.19) |
| Germany | 17389 (11255 to 27010) | 17731 (11183 to 27017) | 1.97 (-13.3 to 17.89) | 100.34 (64.94 to 155.85) | 111.19 (70.13 to 169.42) | 10.81 (-5.78 to 28.11) |
| Greece | 5632 (3689 to 8645) | 3755 (2459 to 5767) | -33.32 (-33.35 to -33.28) | 200.39 (131.27 to 307.59) | 197.1 (129.07 to 302.68) | -1.64 (-1.68 to -1.59) |
| Iceland | 204 (121 to 325) | 212 (126 to 339) | 4 (3.97 to 4.03) | 241.75 (143.51 to 385.12) | 238.47 (141.53 to 379.96) | -1.36 (-1.38 to -1.33) |
| Ireland | 3633 (2324 to 5435) | 3656 (2339 to 5469) | 0.63 (0.61 to 0.66) | 273.41 (174.91 to 408.99) | 276.63 (176.96 to 413.84) | 1.18 (1.16 to 1.21) |
| Israel | 4864 (3194 to 7207) | 8373 (5453 to 12504) | 72.16 (44.58 to 100.15) | 242.43 (159.19 to 359.23) | 248.22 (161.64 to 370.67) | 2.39 (-14.01 to 19.04) |
| Italy | 19825 (13136 to 29612) | 17969 (11665 to 27108) | -9.36 (-21.99 to 4.12) | 145.56 (96.45 to 217.42) | 171.46 (111.31 to 258.67) | 17.79 (1.38 to 35.31) |
| Luxembourg | 186 (122 to 286) | 285 (186 to 437) | 52.95 (52.91 to 52.98) | 211.01 (138.14 to 324.09) | 211.06 (138.19 to 324.12) | 0.02 (0 to 0.04) |
| Malta | 245 (161 to 377) | 183 (120 to 282) | -25.27 (-25.34 to -25.21) | 212.92 (139.4 to 326.99) | 218.42 (143.01 to 335.48) | 2.58 (2.49 to 2.67) |
| Monaco | 10 (6 to 15) | 14 (9 to 21) | 41.23 (40.77 to 41.75) | 203.79 (133.47 to 312.91) | 205.96 (134.91 to 316.34) | 1.07 (0.74 to 1.44) |
| Netherlands | 7990 (5281 to 11954) | 8492 (5658 to 12601) | 6.27 (-8.81 to 21.16) | 208.46 (137.77 to 311.87) | 230.06 (153.3 to 341.4) | 10.36 (-5.3 to 25.82) |
| Norway | 1543 (977 to 2366) | 1814 (1150 to 2781) | 17.57 (17.42 to 17.75) | 138.66 (87.83 to 212.67) | 146.09 (92.61 to 224.03) | 5.36 (5.22 to 5.53) |
| Portugal | 5650 (3701 to 8673) | 3668 (2401 to 5633) | -35.08 (-35.11 to -35.04) | 189.52 (124.16 to 290.93) | 191.36 (125.29 to 293.91) | 0.97 (0.92 to 1.03) |
| San Marino | 11 (7 to 17) | 12 (8 to 18) | 10.92 (10.74 to 11.07) | 180.66 (118.32 to 277.38) | 192.13 (125.87 to 294.93) | 6.35 (6.19 to 6.5) |
| Spain | 39240 (28763 to 53707) | 37269 (24798 to 54372) | -5.02 (-23.48 to 17.4) | 352.02 (258.03 to 481.81) | 423.16 (281.56 to 617.35) | 20.21 (-3.16 to 48.58) |
| Sweden | 3405 (2297 to 4971) | 4133 (2756 to 6057) | 21.37 (7.67 to 36.06) | 161.61 (109.01 to 235.94) | 171.11 (114.09 to 250.76) | 5.88 (-6.07 to 18.69) |
| Switzerland | 4025 (2762 to 5977) | 4594 (2974 to 6822) | 14.12 (-1.69 to 33.01) | 255.23 (175.15 to 378.96) | 260.62 (168.75 to 387.03) | 2.11 (-12.03 to 19.02) |
| United Kingdom | 29375 (19242 to 44097) | 35333 (22918 to 53047) | 20.28 (8.2 to 34.55) | 198.03 (129.72 to 297.27) | 225.7 (146.4 to 338.86) | 13.98 (2.53 to 27.49) |
| Andean Latin America | 56153 (38054 to 83133) | 69087 (46823 to 102346) | 23.03 (22.88 to 23.18) | 296.22 (200.75 to 438.56) | 291.83 (197.78 to 432.32) | -1.48 (-1.61 to -1.37) |
| Bolivia (Plurinational State of) | 10249 (6944 to 15178) | 13286 (9004 to 19673) | 29.64 (29.57 to 29.69) | 306.03 (207.35 to 453.22) | 292.2 (198.04 to 432.68) | -4.52 (-4.57 to -4.48) |
| Ecuador | 14606 (9899 to 21623) | 19252 (13049 to 28503) | 31.81 (31.77 to 31.84) | 294.62 (199.68 to 436.16) | 290.98 (197.23 to 430.8) | -1.24 (-1.26 to -1.21) |
| Peru | 31298 (21211 to 46332) | 36549 (24769 to 54152) | 16.78 (16.54 to 17.01) | 293.89 (199.17 to 435.06) | 292.15 (197.99 to 432.86) | -0.59 (-0.79 to -0.39) |
| Caribbean | 59736 (40991 to 88200) | 60858 (41055 to 90770) | 1.88 (-0.46 to 3.54) | 395.63 (271.48 to 584.15) | 398.74 (268.99 to 594.72) | 0.79 (-1.53 to 2.43) |
| Antigua and Barbuda | 97 (65 to 144) | 86 (58 to 129) | -10.55 (-10.64 to -10.46) | 404.11 (272.81 to 602.46) | 369.62 (249.44 to 551.06) | -8.53 (-8.62 to -8.45) |
| Bahamas | 427 (288 to 637) | 396 (267 to 589) | -7.36 (-7.44 to -7.28) | 396.71 (267.82 to 591.23) | 343.28 (231.98 to 511.11) | -13.47 (-13.55 to -13.4) |
| Barbados | 321 (217 to 479) | 238 (161 to 355) | -25.8 (-25.88 to -25.73) | 378.39 (255.46 to 563.95) | 359.1 (242.33 to 535.24) | -5.1 (-5.19 to -5.01) |
| Belize | 442 (298 to 659) | 635 (429 to 946) | 43.61 (43.48 to 43.72) | 430.83 (290.64 to 642.72) | 375.44 (253.49 to 559.54) | -12.86 (-12.94 to -12.8) |
| Bermuda | 63 (42 to 94) | 43 (29 to 64) | -31.42 (-31.49 to -31.35) | 395.44 (266.77 to 589.99) | 375.09 (253.19 to 559.09) | -5.15 (-5.25 to -5.06) |
| Cuba | 13072 (8811 to 19514) | 9541 (6435 to 14228) | -27.01 (-27.1 to -26.94) | 357.94 (241.25 to 534.33) | 397.36 (268 to 592.53) | 11.01 (10.88 to 11.12) |
| Dominica | 131 (88 to 195) | 70 (47 to 104) | -46.72 (-46.86 to -46.58) | 398.65 (268.6 to 595.02) | 358.36 (242.13 to 533.49) | -10.11 (-10.34 to -9.88) |
| Dominican Republic | 14007 (9450 to 20899) | 15621 (10534 to 23308) | 11.52 (11.4 to 11.64) | 399.95 (269.83 to 596.74) | 401.49 (270.75 to 599.06) | 0.39 (0.28 to 0.49) |
| Grenada | 181 (122 to 269) | 113 (76 to 169) | -37.39 (-37.45 to -37.33) | 428.34 (288.9 to 639.06) | 375.57 (253.35 to 560.06) | -12.32 (-12.4 to -12.24) |
| Guyana | 1485 (1002 to 2216) | 1139 (768 to 1700) | -23.26 (-23.36 to -23.17) | 389.1 (262.48 to 580.75) | 405.88 (273.74 to 605.56) | 4.31 (4.18 to 4.44) |
| Haiti | 14324 (9668 to 21371) | 23103 (15585 to 34466) | 61.28 (61.05 to 61.51) | 428.05 (288.91 to 638.62) | 411.98 (277.92 to 614.61) | -3.75 (-3.9 to -3.62) |
| Jamaica | 4420 (2983 to 6589) | 2985 (2016 to 4449) | -32.45 (-32.5 to -32.41) | 404.54 (273.06 to 603.15) | 366.14 (247.2 to 545.61) | -9.49 (-9.56 to -9.43) |
| Puerto Rico | 5063 (3781 to 6688) | 2221 (1500 to 3305) | -56.14 (-65.39 to -46.27) | 380.1 (283.87 to 502.06) | 339.8 (229.56 to 505.82) | -10.6 (-29.44 to 9.53) |
| Saint Kitts and Nevis | 75 (51 to 112) | 51 (34 to 76) | -32.23 (-32.25 to -32.21) | 409.95 (276.67 to 611.25) | 362.97 (245.04 to 541.04) | -11.46 (-11.48 to -11.44) |
| Saint Lucia | 272 (184 to 406) | 154 (104 to 229) | -43.47 (-43.56 to -43.38) | 407.55 (275.13 to 607.7) | 366.66 (247.44 to 546.52) | -10.04 (-10.18 to -9.9) |
| Saint Vincent and the Grenadines | 218 (147 to 325) | 132 (89 to 197) | -39.47 (-39.49 to -39.45) | 406.27 (274.31 to 605.45) | 392.22 (264.85 to 584.36) | -3.46 (-3.49 to -3.42) |
| Suriname | 698 (471 to 1040) | 755 (509 to 1125) | 8.18 (7.96 to 8.39) | 408 (275.53 to 608.1) | 397.51 (268.18 to 592.65) | -2.57 (-2.77 to -2.38) |
| Trinidad and Tobago | 2253 (1521 to 3359) | 1446 (976 to 2155) | -35.83 (-35.88 to -35.78) | 433.74 (292.75 to 646.66) | 397.26 (268.15 to 592.02) | -8.41 (-8.48 to -8.34) |
| United States Virgin Islands | 165 (111 to 246) | 70 (47 to 105) | -57.46 (-57.58 to -57.33) | 395.02 (266.54 to 589.19) | 384.78 (259.33 to 573.92) | -2.59 (-2.88 to -2.31) |
| Central Latin America | 166039 (111432 to 241925) | 163567 (111756 to 240136) | -1.49 (-3.95 to 0.94) | 200.94 (134.85 to 292.78) | 191.79 (131.04 to 281.57) | -4.55 (-6.94 to -2.2) |
| Colombia | 33429 (21997 to 48462) | 29430 (20774 to 42603) | -11.96 (-22.92 to -0.22) | 222.08 (146.13 to 321.95) | 202.14 (142.69 to 292.62) | -8.98 (-20.31 to 3.16) |
| Costa Rica | 3310 (2221 to 4888) | 2938 (1972 to 4339) | -11.24 (-11.3 to -11.18) | 233.42 (156.64 to 344.68) | 214.61 (144.06 to 316.95) | -8.06 (-8.11 to -7.99) |
| El Salvador | 6109 (4100 to 9024) | 5350 (3592 to 7895) | -12.42 (-12.51 to -12.34) | 221.72 (148.79 to 327.51) | 224.61 (150.79 to 331.43) | 1.31 (1.2 to 1.4) |
| Guatemala | 11888 (7975 to 17579) | 14425 (9681 to 21315) | 21.34 (21.27 to 21.4) | 242.07 (162.39 to 357.98) | 218.06 (146.36 to 322.22) | -9.92 (-9.97 to -9.87) |
| Honduras | 6484 (4351 to 9578) | 9385 (6299 to 13860) | 44.74 (44.69 to 44.77) | 238.94 (160.33 to 352.98) | 215.71 (144.78 to 318.57) | -9.72 (-9.75 to -9.7) |
| Mexico | 65314 (43532 to 96897) | 63417 (42314 to 93923) | -2.9 (-3.35 to -2.39) | 150.61 (100.38 to 223.43) | 146.97 (98.06 to 217.66) | -2.42 (-2.87 to -1.9) |
| Nicaragua | 5368 (3602 to 7934) | 5754 (3862 to 8493) | 7.18 (7.05 to 7.3) | 238.55 (160.06 to 352.59) | 221.36 (148.59 to 326.74) | -7.21 (-7.32 to -7.11) |
| Panama | 2410 (1617 to 3559) | 3387 (2274 to 4999) | 40.54 (40.47 to 40.61) | 221.69 (148.78 to 327.42) | 224.71 (150.84 to 331.68) | 1.36 (1.31 to 1.41) |
| Venezuela (Bolivarian Republic of) | 31727 (21313 to 46370) | 29482 (19809 to 43076) | -7.08 (-7.11 to -7.04) | 349.57 (234.82 to 510.9) | 336.87 (226.34 to 492.2) | -3.63 (-3.67 to -3.6) |
| Tropical Latin America | 198998 (135032 to 291627) | 184936 (124007 to 276248) | -7.07 (-15.49 to 1.88) | 287.31 (194.96 to 421.05) | 277.73 (186.23 to 414.86) | -3.33 (-12.1 to 5.98) |
| Brazil | 192751 (130305 to 282482) | 177651 (118939 to 265216) | -7.83 (-16.48 to 1.39) | 286.86 (193.93 to 420.41) | 277.94 (186.08 to 414.94) | -3.11 (-12.2 to 6.59) |
| Paraguay | 6247 (4311 to 9281) | 7286 (5030 to 10819) | 16.62 (16.54 to 16.69) | 301.93 (208.37 to 448.57) | 272.8 (188.34 to 405.11) | -9.65 (-9.71 to -9.6) |
| North Africa and Middle East | 331103 (227615 to 493290) | 392269 (267369 to 582116) | 18.47 (13.68 to 23.95) | 187.3 (128.76 to 279.05) | 165.87 (113.06 to 246.15) | -11.44 (-15.03 to -7.35) |
| Afghanistan | 7320 (4906 to 10980) | 25722 (17242 to 38597) | 251.4 (251.06 to 251.77) | 131.35 (88.03 to 197.02) | 144.69 (96.99 to 217.12) | 10.16 (10.06 to 10.28) |
| Algeria | 19593 (13134 to 29387) | 24572 (16473 to 36863) | 25.42 (25.35 to 25.48) | 144.95 (97.17 to 217.41) | 149.39 (100.15 to 224.11) | 3.06 (3.01 to 3.11) |
| Bahrain | 300 (201 to 450) | 531 (356 to 796) | 77.03 (76.93 to 77.13) | 149.92 (100.5 to 224.92) | 131.61 (88.24 to 197.39) | -12.21 (-12.26 to -12.17) |
| Egypt | 39360 (26382 to 59051) | 67512 (45261 to 101288) | 71.52 (71.47 to 71.57) | 140.96 (94.48 to 211.48) | 145.03 (97.23 to 217.59) | 2.89 (2.86 to 2.92) |
| Iran (Islamic Republic of) | 117388 (80888 to 172892) | 92619 (63724 to 134403) | -21.1 (-28.88 to -12.05) | 372.55 (256.71 to 548.7) | 358.23 (246.47 to 519.84) | -3.84 (-13.33 to 7.19) |
| Iraq | 16294 (10940 to 24332) | 26510 (17797 to 39341) | 62.69 (41.06 to 88.22) | 158.22 (106.23 to 236.26) | 150.31 (100.91 to 223.06) | -5 (-17.63 to 9.91) |
| Jordan | 2920 (1958 to 4381) | 6299 (4223 to 9447) | 115.68 (115.61 to 115.74) | 139.29 (93.37 to 208.95) | 127.89 (85.75 to 191.82) | -8.18 (-8.21 to -8.16) |
| Kuwait | 1024 (687 to 1537) | 1517 (1017 to 2276) | 48.09 (48.01 to 48.18) | 149.33 (100.09 to 224) | 138.48 (92.85 to 207.72) | -7.27 (-7.32 to -7.21) |
| Lebanon | 1867 (1251 to 2801) | 2300 (1542 to 3451) | 23.2 (23.15 to 23.25) | 139.39 (93.45 to 209.18) | 138.02 (92.56 to 207.11) | -0.98 (-1.03 to -0.95) |
| Libya | 3161 (2118 to 4737) | 2558 (1716 to 3837) | -19.05 (-19.2 to -18.88) | 136.93 (91.76 to 205.24) | 122.56 (82.19 to 183.81) | -10.49 (-10.66 to -10.3) |
| Morocco | 17260 (11569 to 25885) | 17355 (11635 to 26033) | 0.55 (0.47 to 0.64) | 138.31 (92.7 to 207.41) | 134.37 (90.09 to 201.56) | -2.84 (-2.92 to -2.76) |
| Oman | 1529 (1025 to 2293) | 2306 (1546 to 3459) | 50.88 (50.82 to 50.94) | 152.52 (102.22 to 228.75) | 154.61 (103.65 to 231.89) | 1.37 (1.33 to 1.41) |
| Palestine | 1750 (1173 to 2625) | 3345 (2243 to 5018) | 91.17 (91.09 to 91.26) | 146.61 (98.26 to 219.96) | 138.93 (93.15 to 208.39) | -5.23 (-5.28 to -5.19) |
| Qatar | 223 (149 to 334) | 893 (598 to 1339) | 300.65 (300.5 to 300.8) | 145.56 (97.55 to 218.36) | 149.51 (100.22 to 224.24) | 2.72 (2.68 to 2.75) |
| Saudi Arabia | 10622 (7119 to 16243) | 12073 (8124 to 18293) | 13.66 (10.15 to 17.1) | 129.36 (86.7 to 197.81) | 119.14 (80.18 to 180.52) | -7.9 (-10.74 to -5.11) |
| Sudan | 15812 (10600 to 23728) | 29645 (19875 to 44475) | 87.48 (87.4 to 87.55) | 143.15 (95.96 to 214.81) | 138.69 (92.98 to 208.07) | -3.12 (-3.15 to -3.08) |
| Syrian Arab Republic | 10743 (7201 to 16115) | 5780 (3876 to 8667) | -46.2 (-46.22 to -46.17) | 146.12 (97.95 to 219.2) | 106.25 (71.24 to 159.33) | -27.29 (-27.32 to -27.25) |
| Tunisia | 5565 (3731 to 8348) | 5032 (3374 to 7551) | -9.58 (-9.66 to -9.48) | 139.48 (93.51 to 209.21) | 140.35 (94.11 to 210.59) | 0.63 (0.53 to 0.73) |
| Türkiye | 47642 (32545 to 70404) | 45235 (31060 to 67932) | -5.05 (-17.6 to 10.01) | 178.56 (121.98 to 263.87) | 183.05 (125.69 to 274.89) | 2.51 (-11.04 to 18.78) |
| United Arab Emirates | 490 (325 to 753) | 1157 (760 to 1722) | 136.22 (106.69 to 172.28) | 69.3 (45.92 to 106.46) | 68.28 (44.84 to 101.58) | -1.47 (-13.79 to 13.57) |
| Yemen | 10058 (6626 to 15262) | 18940 (12478 to 28724) | 88.32 (88.17 to 88.47) | 119.62 (78.81 to 181.52) | 108.4 (71.42 to 164.4) | -9.38 (-9.45 to -9.3) |
| South Asia | 458767 (303134 to 692596) | 511250 (339697 to 756376) | 11.44 (2.8 to 19.65) | 84.58 (55.88 to 127.68) | 74.8 (49.7 to 110.66) | -11.56 (-18.42 to -5.05) |
| Bangladesh | 67169 (44314 to 99667) | 60177 (39709 to 89248) | -10.41 (-10.48 to -10.32) | 111.28 (73.42 to 165.12) | 98.67 (65.11 to 146.34) | -11.33 (-11.4 to -11.24) |
| Bhutan | 290 (192 to 427) | 203 (134 to 298) | -30.06 (-30.17 to -29.96) | 85.09 (56.48 to 125.26) | 79.5 (52.74 to 116.97) | -6.57 (-6.72 to -6.43) |
| India | 325826 (211979 to 491534) | 343996 (224529 to 511394) | 5.58 (-6.33 to 17.22) | 79.39 (51.65 to 119.76) | 68.72 (44.85 to 102.16) | -13.44 (-23.2 to -3.89) |
| Nepal | 9463 (6280 to 13930) | 10145 (6734 to 14934) | 7.21 (7.18 to 7.25) | 91.44 (60.68 to 134.61) | 81.46 (54.07 to 119.91) | -10.91 (-10.94 to -10.89) |
| Pakistan | 56019 (36571 to 84991) | 96729 (63152 to 146746) | 72.67 (72.66 to 72.69) | 91.89 (59.99 to 139.41) | 88.56 (57.82 to 134.35) | -3.62 (-3.63 to -3.62) |
| East Asia | 1162373 (800239 to 1736965) | 1225314 (835309 to 1772637) | 5.41 (-2.88 to 14.74) | 252.61 (173.91 to 377.49) | 355.21 (242.15 to 513.88) | 40.62 (29.55 to 53.05) |
| China | 1134707 (781431 to 1695760) | 1205791 (822094 to 1743907) | 6.26 (-2.29 to 15.84) | 254.97 (175.59 to 381.03) | 360.69 (245.91 to 521.65) | 41.46 (30.08 to 54.22) |
| Democratic People's Republic of Korea | 16436 (10822 to 24661) | 13125 (8647 to 19681) | -20.15 (-20.2 to -20.1) | 211.01 (138.93 to 316.6) | 198.8 (130.97 to 298.1) | -5.79 (-5.85 to -5.73) |
| Taiwan (Province of China) | 11229 (7350 to 17289) | 6398 (4187 to 9854) | -43.02 (-43.04 to -43) | 153.64 (100.56 to 236.55) | 158.16 (103.49 to 243.57) | 2.94 (2.91 to 2.98) |
| Southeast Asia | 302200 (210111 to 443989) | 286808 (191716 to 424134) | -5.09 (-9.81 to -2.5) | 137.43 (95.55 to 201.9) | 125.1 (83.62 to 184.99) | -8.97 (-13.5 to -6.48) |
| Cambodia | 7622 (4894 to 11544) | 8255 (5301 to 12496) | 8.32 (8.2 to 8.42) | 133.87 (85.96 to 202.76) | 124.5 (79.94 to 188.44) | -7 (-7.1 to -6.91) |
| Indonesia | 109393 (73178 to 161387) | 107031 (71588 to 157881) | -2.16 (-2.18 to -2.13) | 125.07 (83.67 to 184.52) | 118.64 (79.35 to 175) | -5.15 (-5.17 to -5.12) |
| Lao People's Democratic Republic | 2989 (1919 to 4526) | 3701 (2376 to 5603) | 23.82 (23.78 to 23.85) | 131.76 (84.6 to 199.51) | 123.71 (79.44 to 187.29) | -6.11 (-6.13 to -6.08) |
| Malaysia | 5236 (3357 to 7813) | 6194 (3957 to 9461) | 18.29 (5.38 to 34.16) | 62.76 (40.24 to 93.65) | 60.4 (38.58 to 92.25) | -3.77 (-14.27 to 9.14) |
| Maldives | 175 (112 to 265) | 164 (105 to 248) | -6.42 (-6.5 to -6.35) | 136.4 (87.57 to 206.53) | 125.73 (80.74 to 190.27) | -7.83 (-7.91 to -7.76) |
| Mauritius | 507 (326 to 768) | 318 (204 to 482) | -37.22 (-37.22 to -37.21) | 117.71 (75.6 to 178.21) | 107.24 (68.87 to 162.36) | -8.9 (-8.91 to -8.9) |
| Myanmar | 23612 (15165 to 35759) | 24887 (15982 to 37672) | 5.4 (5.32 to 5.47) | 123.55 (79.35 to 187.11) | 119.83 (76.95 to 181.39) | -3.01 (-3.08 to -2.95) |
| Philippines | 40500 (27086 to 59759) | 54493 (36464 to 80413) | 34.55 (34.42 to 34.64) | 126.36 (84.51 to 186.44) | 121.7 (81.43 to 179.58) | -3.69 (-3.78 to -3.62) |
| Seychelles | 38 (24 to 57) | 37 (24 to 57) | -1.25 (-1.26 to -1.25) | 121.34 (77.92 to 183.71) | 122.63 (78.75 to 185.64) | 1.06 (1.05 to 1.06) |
| Sri Lanka | 8868 (5696 to 13427) | 8041 (5164 to 12173) | -9.33 (-9.34 to -9.32) | 122.48 (78.67 to 185.45) | 116.53 (74.85 to 176.43) | -4.86 (-4.87 to -4.85) |
| Thailand | 58546 (39811 to 79190) | 31115 (21395 to 46244) | -46.85 (-57.91 to -37.7) | 257.38 (175.02 to 348.14) | 227.98 (156.76 to 338.83) | -11.42 (-29.85 to 3.83) |
| Timor-Leste | 552 (354 to 836) | 829 (532 to 1255) | 50.16 (50.09 to 50.23) | 135.8 (87.17 to 205.57) | 120.98 (77.68 to 183.12) | -10.91 (-10.95 to -10.87) |
| Viet Nam | 43724 (28076 to 66186) | 41342 (26544 to 62559) | -5.45 (-5.6 to -5.31) | 129.87 (83.39 to 196.59) | 130.75 (83.95 to 197.85) | 0.68 (0.51 to 0.82) |
| Oceania | 5595 (3803 to 8376) | 10615 (7215 to 15889) | 89.71 (89.69 to 89.72) | 166.21 (112.98 to 248.8) | 166.21 (112.97 to 248.8) | 0 (-0.01 to 0.01) |
| American Samoa | 39 (27 to 59) | 29 (19 to 43) | -27.28 (-27.4 to -27.17) | 164.32 (111.68 to 246.01) | 149.47 (101.68 to 223.78) | -9.04 (-9.19 to -8.9) |
| Cook Islands | 14 (9 to 21) | 8 (5 to 11) | -45.16 (-45.21 to -45.12) | 159.44 (108.39 to 238.65) | 145.25 (98.78 to 217.46) | -8.9 (-8.98 to -8.82) |
| Fiji | 592 (403 to 886) | 567 (385 to 849) | -4.25 (-4.26 to -4.24) | 165.02 (112.21 to 247.14) | 161.65 (109.92 to 242.1) | -2.05 (-2.06 to -2.04) |
| Guam | 85 (58 to 127) | 73 (50 to 110) | -13.71 (-13.79 to -13.62) | 156.97 (106.72 to 235.25) | 151.26 (102.83 to 226.52) | -3.64 (-3.72 to -3.54) |
| Kiribati | 62 (42 to 93) | 87 (59 to 131) | 40.21 (40.15 to 40.28) | 170.19 (115.69 to 254.99) | 163.65 (111.27 to 245.06) | -3.84 (-3.88 to -3.79) |
| Marshall Islands | 47 (32 to 71) | 36 (24 to 53) | -24.71 (-24.71 to -24.71) | 176.3 (119.88 to 264.05) | 154.08 (104.78 to 230.78) | -12.6 (-12.61 to -12.6) |
| Micronesia (Federated States of) | 97 (66 to 145) | 62 (42 to 93) | -36.28 (-36.34 to -36.23) | 168.72 (114.71 to 252.54) | 149.15 (101.43 to 223.32) | -11.6 (-11.68 to -11.52) |
| Nauru | 9 (6 to 13) | 8 (6 to 12) | -7.93 (-8.03 to -7.8) | 172.58 (117.33 to 258.57) | 161.42 (109.73 to 241.61) | -6.47 (-6.58 to -6.34) |
| Niue | 2 (1 to 3) | 1 (1 to 1) | -55.56 (-55.57 to -55.55) | 171.86 (116.89 to 257.48) | 147.86 (100.57 to 221.49) | -13.97 (-13.98 to -13.95) |
| Northern Mariana Islands | 24 (16 to 35) | 23 (15 to 34) | -4.7 (-5.02 to -4.32) | 145.6 (98.94 to 218.35) | 150.04 (102.03 to 224.5) | 3.05 (2.71 to 3.46) |
| Palau | 9 (6 to 14) | 7 (4 to 10) | -29.23 (-29.27 to -29.17) | 149.36 (101.57 to 223.7) | 147.18 (100.09 to 220.29) | -1.46 (-1.53 to -1.38) |
| Papua New Guinea | 3552 (2415 to 5317) | 8193 (5569 to 12264) | 130.64 (130.62 to 130.66) | 166.89 (113.43 to 249.78) | 167.24 (113.67 to 250.33) | 0.21 (0.2 to 0.22) |
| Samoa | 146 (99 to 218) | 167 (114 to 250) | 14.59 (14.47 to 14.69) | 159.28 (108.27 to 238.36) | 165.61 (112.59 to 248) | 3.98 (3.87 to 4.07) |
| Solomon Islands | 322 (219 to 482) | 541 (368 to 810) | 68.02 (68 to 68.04) | 165.48 (112.48 to 247.76) | 163.61 (111.21 to 244.91) | -1.13 (-1.15 to -1.12) |
| Tokelau | 1 (1 to 2) | 1 (1 to 1) | -38.96 (-39.04 to -38.85) | 176.85 (120.27 to 264.86) | 160.04 (108.86 to 239.35) | -9.51 (-9.63 to -9.34) |
| Tonga | 86 (58 to 128) | 82 (56 to 123) | -4.37 (-4.43 to -4.31) | 160.72 (109.25 to 240.65) | 165.41 (112.43 to 247.59) | 2.92 (2.86 to 2.99) |
| Tuvalu | 7 (5 to 11) | 8 (5 to 12) | 5.22 (5.09 to 5.35) | 172.03 (116.86 to 257.38) | 157.44 (107.03 to 235.56) | -8.48 (-8.6 to -8.37) |
| Vanuatu | 143 (97 to 214) | 247 (168 to 369) | 72.17 (72.05 to 72.29) | 171.69 (116.69 to 257.02) | 167.3 (113.74 to 250.51) | -2.56 (-2.62 to -2.49) |
| Central sub-Saharan Africa | 24027 (15846 to 35523) | 56589 (37326 to 83653) | 135.53 (135.42 to 135.64) | 77.53 (51.13 to 114.64) | 76.93 (50.74 to 113.72) | -0.78 (-0.83 to -0.74) |
| Angola | 4428 (2920 to 6546) | 14800 (9761 to 21881) | 234.28 (234.16 to 234.4) | 76.79 (50.64 to 113.53) | 79.25 (52.27 to 117.16) | 3.21 (3.17 to 3.24) |
| Central African Republic | 1170 (772 to 1730) | 2166 (1428 to 3201) | 85.08 (85.05 to 85.12) | 78.15 (51.54 to 115.54) | 74.83 (49.36 to 110.62) | -4.25 (-4.27 to -4.23) |
| Congo | 989 (652 to 1462) | 1832 (1209 to 2708) | 85.31 (85.13 to 85.48) | 74.53 (49.15 to 110.19) | 73.68 (48.6 to 108.91) | -1.14 (-1.23 to -1.05) |
| Democratic Republic of the Congo | 16864 (11122 to 24934) | 36613 (24151 to 54122) | 117.11 (116.95 to 117.27) | 77.89 (51.37 to 115.17) | 76.42 (50.41 to 112.97) | -1.89 (-1.96 to -1.81) |
| Equatorial Guinea | 189 (125 to 279) | 573 (378 to 847) | 203.24 (201.81 to 204.7) | 78.78 (51.96 to 116.46) | 74.44 (49.11 to 109.98) | -5.51 (-5.95 to -5.05) |
| Gabon | 387 (255 to 572) | 605 (399 to 894) | 56.18 (56.06 to 56.3) | 76.18 (50.24 to 112.63) | 73.1 (48.21 to 108.13) | -4.04 (-4.11 to -3.96) |
| Eastern sub-Saharan Africa | 85601 (56078 to 127383) | 169212 (111067 to 251831) | 97.67 (96.49 to 99.03) | 77.19 (50.57 to 114.87) | 74.35 (48.8 to 110.65) | -3.68 (-4.26 to -3.02) |
| Burundi | 2526 (1666 to 3736) | 5582 (3682 to 8253) | 120.95 (120.93 to 120.97) | 80.08 (52.81 to 118.4) | 76.74 (50.61 to 113.46) | -4.17 (-4.18 to -4.16) |
| Comoros | 201 (133 to 297) | 228 (150 to 336) | 13.1 (13.05 to 13.15) | 76.13 (50.22 to 112.55) | 72.49 (47.82 to 107.16) | -4.78 (-4.82 to -4.74) |
| Djibouti | 164 (109 to 243) | 405 (267 to 599) | 146.23 (145.83 to 146.63) | 73.35 (48.39 to 108.4) | 76.66 (50.58 to 113.28) | 4.51 (4.35 to 4.69) |
| Eritrea | 1530 (1010 to 2262) | 2440 (1610 to 3607) | 59.45 (59.41 to 59.51) | 77.82 (51.33 to 115.03) | 75.87 (50.05 to 112.14) | -2.5 (-2.53 to -2.47) |
| Ethiopia | 23121 (15048 to 34915) | 41435 (26968 to 62563) | 79.21 (79.14 to 79.28) | 78.46 (51.07 to 118.49) | 72.44 (47.15 to 109.38) | -7.67 (-7.71 to -7.64) |
| Kenya | 10524 (6849 to 15888) | 17338 (11285 to 26171) | 64.75 (64.67 to 64.83) | 76.3 (49.66 to 115.2) | 70.48 (45.87 to 106.39) | -7.64 (-7.68 to -7.59) |
| Madagascar | 5187 (3421 to 7668) | 11243 (7416 to 16621) | 116.77 (116.72 to 116.82) | 77.11 (50.86 to 114.01) | 75.24 (49.63 to 111.22) | -2.43 (-2.46 to -2.41) |
| Malawi | 4239 (2796 to 6268) | 7554 (4982 to 11167) | 78.18 (78.14 to 78.23) | 76.07 (50.16 to 112.47) | 71.33 (47.04 to 105.44) | -6.23 (-6.26 to -6.21) |
| Mozambique | 5893 (3887 to 8713) | 13807 (9106 to 20412) | 134.29 (134.26 to 134.31) | 77.84 (51.34 to 115.08) | 77.77 (51.29 to 114.97) | -0.1 (-0.11 to -0.08) |
| Rwanda | 3274 (2159 to 4842) | 4708 (3106 to 6960) | 43.81 (43.64 to 44) | 79.11 (52.17 to 116.99) | 73.13 (48.24 to 108.11) | -7.55 (-7.67 to -7.43) |
| Somalia | 3738 (2466 to 5525) | 10055 (6632 to 14863) | 168.97 (168.45 to 169.48) | 78.99 (52.12 to 116.75) | 78.65 (51.88 to 116.26) | -0.44 (-0.63 to -0.25) |
| South Sudan | 2501 (1650 to 3697) | 3983 (2627 to 5886) | 59.23 (59.16 to 59.31) | 76.11 (50.21 to 112.5) | 72.58 (47.88 to 107.27) | -4.64 (-4.69 to -4.6) |
| Uganda | 7858 (5182 to 11619) | 19063 (12575 to 28179) | 142.6 (142.22 to 142.99) | 76.31 (50.32 to 112.83) | 76.43 (50.41 to 112.97) | 0.16 (0 to 0.32) |
| United Republic of Tanzania | 11336 (7476 to 16760) | 23290 (15361 to 34431) | 105.46 (105.43 to 105.48) | 75.96 (50.1 to 112.31) | 75.48 (49.78 to 111.58) | -0.64 (-0.65 to -0.63) |
| Zambia | 3446 (2273 to 5096) | 7933 (5232 to 11727) | 130.19 (130.07 to 130.32) | 73.32 (48.35 to 108.41) | 75.88 (50.05 to 112.18) | 3.5 (3.45 to 3.56) |
| Southern sub-Saharan Africa | 19456 (12645 to 29026) | 22652 (14720 to 33838) | 16.43 (16.07 to 16.87) | 73.53 (47.79 to 109.7) | 72.45 (47.08 to 108.23) | -1.46 (-1.76 to -1.09) |
| Botswana | 558 (368 to 825) | 659 (435 to 974) | 18.07 (17.93 to 18.23) | 75.39 (49.72 to 111.46) | 71.88 (47.41 to 106.25) | -4.66 (-4.78 to -4.53) |
| Eswatini | 368 (243 to 545) | 394 (260 to 583) | 7.05 (6.77 to 7.33) | 77.22 (50.93 to 114.17) | 73.72 (48.63 to 108.96) | -4.53 (-4.78 to -4.28) |
| Lesotho | 656 (433 to 970) | 592 (390 to 875) | -9.8 (-9.84 to -9.75) | 78.36 (51.69 to 115.84) | 70.56 (46.54 to 104.3) | -9.96 (-10 to -9.91) |
| Namibia | 565 (373 to 835) | 775 (511 to 1146) | 37.26 (37.23 to 37.28) | 74.17 (48.92 to 109.66) | 72.38 (47.74 to 106.99) | -2.42 (-2.44 to -2.41) |
| South Africa | 12720 (8279 to 19201) | 14237 (9267 to 21491) | 11.92 (11.83 to 12.02) | 72.19 (46.98 to 108.96) | 71.48 (46.53 to 107.9) | -0.98 (-1.07 to -0.89) |
| Zimbabwe | 4588 (3026 to 6783) | 5994 (3953 to 8861) | 30.64 (30.59 to 30.7) | 76.18 (50.24 to 112.62) | 75.07 (49.51 to 110.98) | -1.46 (-1.5 to -1.41) |
| Western sub-Saharan Africa | 85672 (56242 to 129714) | 215764 (140538 to 322844) | 151.85 (138.26 to 164.36) | 79.7 (52.32 to 120.67) | 80.34 (52.33 to 120.21) | 0.8 (-4.64 to 5.81) |
| Benin | 2485 (1607 to 3755) | 6048 (3910 to 9138) | 143.36 (143.1 to 143.71) | 86.61 (56 to 130.86) | 80.47 (52.03 to 121.57) | -7.08 (-7.19 to -6.95) |
| Burkina Faso | 4692 (3034 to 7089) | 10186 (6585 to 15388) | 117.08 (116.95 to 117.25) | 82.94 (53.62 to 125.31) | 79.54 (51.42 to 120.16) | -4.1 (-4.16 to -4.03) |
| Cabo Verde | 158 (102 to 239) | 138 (89 to 208) | -12.83 (-12.89 to -12.78) | 81.26 (52.54 to 122.76) | 71.47 (46.21 to 107.95) | -12.05 (-12.11 to -12) |
| Cameroon | 4849 (3135 to 7327) | 13375 (8648 to 20206) | 175.81 (175.77 to 175.84) | 81.68 (52.8 to 123.41) | 79.13 (51.16 to 119.54) | -3.12 (-3.14 to -3.11) |
| Chad | 2863 (1850 to 4325) | 8960 (5793 to 13538) | 213.02 (212.82 to 213.18) | 81.2 (52.49 to 122.68) | 81.83 (52.9 to 123.64) | 0.78 (0.72 to 0.83) |
| Côte d'Ivoire | 5736 (3709 to 8667) | 11599 (7500 to 17526) | 102.21 (102.03 to 102.35) | 82.91 (53.6 to 125.27) | 80.64 (52.14 to 121.84) | -2.75 (-2.83 to -2.68) |
| Gambia | 455 (294 to 687) | 969 (626 to 1463) | 112.98 (112.88 to 113.07) | 80.97 (52.34 to 122.32) | 76.48 (49.45 to 115.54) | -5.53 (-5.58 to -5.5) |
| Ghana | 6693 (4327 to 10112) | 12829 (8295 to 19381) | 91.68 (91.66 to 91.7) | 81.03 (52.39 to 122.43) | 78.64 (50.84 to 118.8) | -2.96 (-2.97 to -2.95) |
| Guinea | 2743 (1773 to 4145) | 6038 (3904 to 9122) | 120.11 (120.08 to 120.13) | 83.45 (53.95 to 126.09) | 80.89 (52.3 to 122.2) | -3.07 (-3.08 to -3.06) |
| Guinea-Bissau | 476 (308 to 719) | 892 (577 to 1348) | 87.34 (87.13 to 87.51) | 80.99 (52.36 to 122.34) | 79.77 (51.58 to 120.52) | -1.5 (-1.61 to -1.41) |
| Liberia | 1130 (730 to 1707) | 2134 (1380 to 3224) | 88.89 (88.69 to 89.05) | 82.7 (53.47 to 124.94) | 76.49 (49.46 to 115.56) | -7.51 (-7.6 to -7.43) |
| Mali | 4076 (2635 to 6159) | 11476 (7419 to 17338) | 181.51 (181.5 to 181.53) | 82.42 (53.28 to 124.53) | 80.63 (52.13 to 121.82) | -2.18 (-2.18 to -2.17) |
| Mauritania | 919 (594 to 1388) | 1841 (1191 to 2782) | 100.41 (100.32 to 100.54) | 81.03 (52.39 to 122.43) | 78.96 (51.05 to 119.28) | -2.56 (-2.6 to -2.49) |
| Niger | 4025 (2602 to 6082) | 12713 (8219 to 19208) | 215.81 (215.63 to 215.95) | 82.97 (53.64 to 125.35) | 81.56 (52.73 to 123.23) | -1.7 (-1.76 to -1.66) |
| Nigeria | 37181 (24461 to 55501) | 103437 (67394 to 157299) | 178.2 (146.79 to 210.3) | 76.63 (50.42 to 114.39) | 80.95 (52.75 to 123.11) | 5.64 (-6.29 to 17.83) |
| Sao Tome and Principe | 56 (36 to 85) | 75 (49 to 114) | 34.49 (34.48 to 34.51) | 80.15 (51.82 to 121.07) | 74 (47.85 to 111.77) | -7.68 (-7.68 to -7.67) |
| Senegal | 3594 (2324 to 5430) | 6255 (4045 to 9451) | 74.04 (73.57 to 74.41) | 80.83 (52.25 to 122.1) | 77.08 (49.84 to 116.46) | -4.63 (-4.89 to -4.43) |
| Sierra Leone | 1778 (1149 to 2686) | 3497 (2261 to 5283) | 96.68 (96.63 to 96.73) | 80.61 (52.11 to 121.79) | 76.85 (49.68 to 116.09) | -4.67 (-4.69 to -4.64) |
| Togo | 1758 (1136 to 2655) | 3299 (2133 to 4983) | 87.67 (87.49 to 87.81) | 81.65 (52.79 to 123.35) | 79 (51.08 to 119.35) | -3.24 (-3.34 to -3.17) |

Abbreviations: UI, uncertainty interval.

**eTable** 4**c. DALYs caused by ADHD by country, <20 years, 1990-2021.**

|  | **DALYs from ADHD** | | | | | |
| --- | --- | --- | --- | --- | --- | --- |
|  | **Counts (<20 years)** | | | **Rates per 100,000 persons (<20 years)** | | |
| **Country** | **1990 (95% UI)** | **2021 (95% UI)** | **Percentage change from 1990 to 2021 (%)** | **1990 (95% UI)** | **2021 (95% UI)** | **Percentage change from 1990 to 2021 (%)** |
| Global | 523435 (271668 to 876127) | 574979 (294277 to 977557) | 9.85 (6.84 to 12.86) | 23.18 (12.03 to 38.79) | 21.81 (11.16 to 37.09) | -5.88 (-8.45 to -3.29) |
| Central Europe | 8078 (4026 to 13867) | 4842 (2455 to 8267) | -40.06 (-41.78 to -38.07) | 20.57 (10.25 to 35.31) | 20.56 (10.42 to 35.09) | -0.08 (-2.95 to 3.23) |
| Albania | 277 (139 to 482) | 127 (64 to 222) | -54.36 (-58.91 to -49.38) | 19.17 (9.6 to 33.32) | 20.5 (10.4 to 35.92) | 6.94 (-3.73 to 18.6) |
| Bosnia and Herzegovina | 298 (144 to 506) | 136 (68 to 236) | -54.48 (-58.83 to -49.42) | 20.14 (9.68 to 34.18) | 20.43 (10.29 to 35.46) | 1.42 (-8.28 to 12.7) |
| Bulgaria | 484 (246 to 836) | 264 (130 to 452) | -45.58 (-51 to -39.78) | 20.5 (10.41 to 35.37) | 20.37 (10.04 to 34.89) | -0.62 (-10.53 to 9.95) |
| Croatia | 271 (132 to 473) | 165 (82 to 286) | -39.06 (-44.88 to -32.81) | 20.46 (9.98 to 35.7) | 20.53 (10.13 to 35.54) | 0.32 (-9.27 to 10.62) |
| Czechia | 638 (316 to 1102) | 442 (218 to 778) | -30.72 (-37.75 to -22.8) | 20.95 (10.38 to 36.2) | 19.89 (9.8 to 35.03) | -5.03 (-14.67 to 5.83) |
| Hungary | 608 (295 to 1043) | 377 (188 to 640) | -38.01 (-44.57 to -30.9) | 20.93 (10.17 to 35.92) | 20.08 (10.04 to 34.11) | -4.07 (-14.23 to 6.94) |
| Montenegro | 43 (21 to 74) | 30 (15 to 51) | -28.98 (-36.06 to -20.8) | 20 (9.99 to 34.52) | 20.26 (9.85 to 34.13) | 1.31 (-8.79 to 12.98) |
| North Macedonia | 139 (68 to 241) | 92 (46 to 159) | -34.33 (-40.81 to -27.2) | 20.01 (9.75 to 34.63) | 20.51 (10.36 to 35.52) | 2.49 (-7.62 to 13.61) |
| Poland | 2606 (1350 to 4486) | 1605 (837 to 2739) | -38.44 (-40.54 to -36.46) | 20.94 (10.84 to 36.03) | 20.84 (10.87 to 35.57) | -0.44 (-3.85 to 2.75) |
| Romania | 1528 (724 to 2635) | 825 (412 to 1411) | -45.99 (-51.76 to -39.66) | 20.36 (9.65 to 35.12) | 20.42 (10.2 to 34.91) | 0.29 (-10.43 to 12.04) |
| Serbia | 584 (288 to 1024) | 406 (200 to 692) | -30.48 (-37.27 to -22.13) | 20.13 (9.92 to 35.29) | 21.67 (10.66 to 36.93) | 7.64 (-2.88 to 20.56) |
| Slovakia | 357 (178 to 622) | 223 (110 to 382) | -37.61 (-43.53 to -30.44) | 20.32 (10.13 to 35.44) | 19.85 (9.8 to 34.11) | -2.33 (-11.59 to 8.91) |
| Slovenia | 115 (57 to 195) | 82 (42 to 139) | -29.15 (-35.81 to -20.8) | 20.66 (10.28 to 35.1) | 20.13 (10.3 to 34.4) | -2.56 (-11.72 to 8.93) |
| Eastern Europe | 13650 (7069 to 23653) | 9850 (5071 to 17085) | -27.84 (-29.77 to -26.02) | 20.29 (10.51 to 35.16) | 21.34 (10.98 to 37.01) | 5.17 (2.36 to 7.83) |
| Belarus | 615 (307 to 1074) | 412 (208 to 716) | -33 (-39.23 to -25.96) | 19.61 (9.79 to 34.23) | 20.36 (10.27 to 35.33) | 3.82 (-5.84 to 14.72) |
| Estonia | 90 (43 to 155) | 56 (28 to 96) | -37.37 (-43.11 to -29.6) | 19.52 (9.43 to 33.69) | 20.03 (9.95 to 34.07) | 2.63 (-6.77 to 15.36) |
| Latvia | 145 (72 to 246) | 78 (39 to 132) | -46.39 (-51.94 to -40.01) | 19.29 (9.58 to 32.71) | 20.11 (10.01 to 34.21) | 4.21 (-6.59 to 16.6) |
| Lithuania | 217 (107 to 368) | 107 (51 to 185) | -50.54 (-55.82 to -44.54) | 19.55 (9.66 to 33.24) | 19.99 (9.6 to 34.48) | 2.24 (-8.66 to 14.65) |
| Republic of Moldova | 305 (146 to 524) | 144 (72 to 245) | -52.86 (-57.47 to -47.65) | 19.28 (9.23 to 33.13) | 20.64 (10.31 to 35.23) | 7.06 (-3.41 to 18.9) |
| Russian Federation | 9183 (4737 to 15800) | 7166 (3683 to 12354) | -21.97 (-23.46 to -20.57) | 20.32 (10.48 to 34.97) | 21.21 (10.9 to 36.56) | 4.34 (2.35 to 6.22) |
| Ukraine | 3095 (1587 to 5405) | 1887 (959 to 3285) | -39.03 (-45.16 to -32.44) | 20.57 (10.55 to 35.91) | 22.35 (11.36 to 38.9) | 8.67 (-2.26 to 20.41) |
| Central Asia | 5815 (2864 to 9951) | 6545 (3219 to 11480) | 12.55 (6.99 to 17.97) | 18.41 (9.07 to 31.51) | 18.9 (9.3 to 33.15) | 2.65 (-2.42 to 7.6) |
| Armenia | 250 (125 to 434) | 154 (75 to 267) | -38.26 (-44.21 to -31.96) | 18.83 (9.45 to 32.69) | 20.23 (9.8 to 35.03) | 7.43 (-2.92 to 18.38) |
| Azerbaijan | 590 (296 to 1011) | 624 (304 to 1069) | 5.65 (-5.02 to 18.98) | 18.86 (9.46 to 32.29) | 20.47 (9.98 to 35.09) | 8.54 (-2.43 to 22.24) |
| Georgia | 352 (176 to 605) | 183 (91 to 315) | -47.89 (-53.06 to -42.35) | 19.58 (9.77 to 33.66) | 19.73 (9.79 to 33.9) | 0.75 (-9.24 to 11.47) |
| Kazakhstan | 1254 (612 to 2133) | 1266 (639 to 2181) | 0.97 (-8.11 to 11.89) | 18.87 (9.21 to 32.11) | 18.83 (9.51 to 32.44) | -0.24 (-9.21 to 10.54) |
| Kyrgyzstan | 388 (192 to 662) | 537 (266 to 943) | 38.26 (24.89 to 52.97) | 18.39 (9.1 to 31.34) | 19.02 (9.42 to 33.41) | 3.42 (-6.58 to 14.42) |
| Mongolia | 210 (104 to 362) | 244 (126 to 413) | 16.16 (4.18 to 27.97) | 18.48 (9.18 to 31.91) | 18.56 (9.63 to 31.45) | 0.44 (-9.92 to 10.66) |
| Tajikistan | 507 (255 to 885) | 827 (407 to 1417) | 62.96 (46.62 to 80.84) | 17.65 (8.88 to 30.78) | 18.56 (9.14 to 31.82) | 5.14 (-5.4 to 16.68) |
| Turkmenistan | 342 (171 to 584) | 377 (192 to 657) | 10.28 (-1.19 to 21.98) | 18.15 (9.07 to 31) | 19.25 (9.8 to 33.52) | 6.04 (-4.99 to 17.3) |
| Uzbekistan | 1921 (974 to 3290) | 2333 (1149 to 4115) | 21.41 (8.44 to 34.51) | 18 (9.12 to 30.82) | 18.5 (9.11 to 32.63) | 2.79 (-8.19 to 13.87) |
| Australasia | 3947 (2077 to 6750) | 4959 (2644 to 7877) | 25.63 (8.99 to 49.36) | 62.92 (33.11 to 107.6) | 65.75 (35.06 to 104.44) | 4.5 (-9.34 to 24.24) |
| Australia | 3442 (1809 to 5882) | 4296 (2338 to 6808) | 24.81 (5.47 to 50.91) | 66.55 (34.98 to 113.72) | 68.87 (37.48 to 109.14) | 3.48 (-12.55 to 25.12) |
| New Zealand | 505 (261 to 883) | 663 (336 to 1125) | 31.26 (14.31 to 55.67) | 45.86 (23.69 to 80.24) | 50.85 (25.81 to 86.3) | 10.88 (-3.44 to 31.5) |
| High-income Asia Pacific | 14682 (7308 to 24954) | 8840 (4473 to 15054) | -39.79 (-42.73 to -36.58) | 29.17 (14.52 to 49.58) | 28.71 (14.53 to 48.89) | -1.59 (-6.4 to 3.67) |
| Brunei Darussalam | 29 (15 to 50) | 36 (18 to 61) | 22.66 (11.9 to 34.15) | 25.38 (12.89 to 43.67) | 27.73 (13.82 to 47.34) | 9.26 (-0.33 to 19.49) |
| Japan | 9902 (4989 to 17106) | 6199 (3091 to 10748) | -37.39 (-38.77 to -36.17) | 29.68 (14.95 to 51.27) | 29.2 (14.56 to 50.62) | -1.63 (-3.79 to 0.3) |
| Republic of Korea | 4493 (2252 to 7625) | 2334 (1226 to 3952) | -48.05 (-56.54 to -37.89) | 28.21 (14.14 to 47.87) | 27.84 (14.62 to 47.15) | -1.31 (-17.43 to 18) |
| Singapore | 259 (130 to 434) | 271 (138 to 471) | 4.85 (-5.79 to 15.69) | 28.08 (14.12 to 47.13) | 25.98 (13.2 to 45.1) | -7.45 (-16.85 to 2.12) |
| High-income North America | 32194 (16774 to 54869) | 39803 (19988 to 69498) | 23.63 (11.49 to 34.5) | 39.39 (20.52 to 67.13) | 44.44 (22.32 to 77.6) | 12.83 (1.75 to 22.75) |
| Canada | 2835 (1566 to 4443) | 3182 (1658 to 5583) | 12.25 (-10.98 to 38.8) | 36.99 (20.43 to 57.97) | 38.67 (20.15 to 67.85) | 4.55 (-17.09 to 29.28) |
| Greenland | 7 (3 to 12) | 6 (3 to 11) | -8.93 (-15.24 to -2.89) | 37.55 (19.34 to 66.83) | 39.8 (20.6 to 70.02) | 6.01 (-1.33 to 13.04) |
| United States of America | 29352 (15154 to 50476) | 36614 (18236 to 63941) | 24.74 (12.49 to 36.47) | 39.64 (20.47 to 68.17) | 45.03 (22.43 to 78.64) | 13.59 (2.44 to 24.27) |
| Southern Latin America | 5111 (2632 to 8737) | 5425 (2797 to 9159) | 6.14 (-4.76 to 18.13) | 26.37 (13.58 to 45.08) | 27.81 (14.34 to 46.95) | 5.43 (-5.4 to 17.34) |
| Argentina | 3445 (1759 to 5865) | 3797 (1949 to 6481) | 10.2 (-6.93 to 27.71) | 26.45 (13.5 to 45.02) | 27.69 (14.22 to 47.27) | 4.7 (-11.58 to 21.33) |
| Chile | 1376 (704 to 2383) | 1373 (709 to 2364) | -0.23 (-8.14 to 8.91) | 26.09 (13.34 to 45.19) | 28.06 (14.49 to 48.31) | 7.54 (-1 to 17.39) |
| Uruguay | 289 (147 to 502) | 255 (127 to 449) | -11.96 (-19.03 to -3.38) | 26.87 (13.62 to 46.59) | 28.2 (14.1 to 49.7) | 4.97 (-3.46 to 15.2) |
| Western Europe | 29163 (15606 to 48725) | 28442 (14530 to 47980) | -2.47 (-8.89 to 4.06) | 29.65 (15.87 to 49.55) | 31.01 (15.84 to 52.32) | 4.58 (-2.3 to 11.58) |
| Andorra | 4 (2 to 7) | 4 (2 to 8) | 10.12 (0.68 to 20.67) | 29.79 (15.23 to 52.07) | 30.71 (15.72 to 53.82) | 3.08 (-5.75 to 12.96) |
| Austria | 624 (332 to 1094) | 589 (310 to 1018) | -5.64 (-20.96 to 11.69) | 33.34 (17.75 to 58.4) | 33.58 (17.67 to 58.01) | 0.73 (-15.63 to 19.22) |
| Belgium | 699 (349 to 1205) | 731 (367 to 1279) | 4.65 (-4.45 to 14.41) | 28.27 (14.12 to 48.74) | 28.74 (14.41 to 50.26) | 1.67 (-7.17 to 11.15) |
| Cyprus | 74 (38 to 127) | 80 (39 to 141) | 8.14 (-1.4 to 17.14) | 28.41 (14.52 to 49.2) | 27.92 (13.79 to 49.52) | -1.73 (-10.4 to 6.45) |
| Denmark | 363 (185 to 619) | 349 (178 to 609) | -3.7 (-21.02 to 14.85) | 29.01 (14.82 to 49.51) | 26.99 (13.76 to 47.08) | -6.97 (-23.7 to 10.95) |
| Finland | 537 (259 to 920) | 428 (222 to 731) | -20.24 (-44.84 to 1.49) | 42.33 (20.45 to 72.51) | 37.3 (19.34 to 63.62) | -11.88 (-39.07 to 12.12) |
| France | 5221 (2646 to 9192) | 5286 (2672 to 9026) | 1.25 (-12.54 to 19.85) | 32.34 (16.39 to 56.95) | 33.56 (16.97 to 57.3) | 3.76 (-10.38 to 22.82) |
| Germany | 2213 (1157 to 3873) | 2244 (1121 to 3882) | 1.4 (-16.02 to 21.73) | 12.77 (6.68 to 22.35) | 14.07 (7.03 to 24.34) | 10.19 (-8.73 to 32.29) |
| Greece | 840 (418 to 1447) | 554 (279 to 947) | -34 (-39.55 to -27.64) | 29.88 (14.88 to 51.5) | 29.09 (14.62 to 49.71) | -2.64 (-10.83 to 6.73) |
| Iceland | 27 (13 to 48) | 29 (14 to 53) | 6.63 (-2.14 to 15.74) | 32.51 (15.83 to 57.22) | 32.88 (15.89 to 59.05) | 1.14 (-7.18 to 9.78) |
| Ireland | 517 (263 to 878) | 509 (260 to 877) | -1.39 (-9.1 to 6) | 38.88 (19.79 to 66.08) | 38.55 (19.7 to 66.34) | -0.85 (-8.61 to 6.58) |
| Israel | 630 (335 to 1071) | 1030 (526 to 1805) | 63.54 (35.26 to 90.8) | 31.38 (16.72 to 53.38) | 30.52 (15.58 to 53.51) | -2.74 (-19.56 to 13.48) |
| Italy | 3444 (1762 to 5958) | 2805 (1382 to 4835) | -18.57 (-29.16 to -7.12) | 25.29 (12.94 to 43.75) | 26.77 (13.19 to 46.14) | 5.83 (-7.93 to 20.71) |
| Luxembourg | 24 (12 to 43) | 38 (19 to 66) | 56.66 (43.65 to 71.48) | 27.7 (14.15 to 48.2) | 28.38 (14.4 to 49) | 2.45 (-6.06 to 12.14) |
| Malta | 33 (16 to 57) | 23 (12 to 40) | -28.62 (-34.06 to -22.3) | 28.44 (14.12 to 49.14) | 27.87 (13.9 to 48.1) | -2.01 (-9.47 to 6.66) |
| Monaco | 1 (1 to 2) | 2 (1 to 3) | 38.3 (26.58 to 50.73) | 28.47 (14.52 to 49.21) | 28.18 (14.13 to 48.46) | -1.03 (-9.42 to 7.87) |
| Netherlands | 1137 (577 to 1944) | 1210 (621 to 2062) | 6.39 (-11.11 to 25.39) | 29.68 (15.06 to 50.73) | 32.78 (16.81 to 55.85) | 10.48 (-7.69 to 30.22) |
| Norway | 230 (119 to 395) | 267 (135 to 459) | 15.8 (11.4 to 20.15) | 20.71 (10.65 to 35.49) | 21.49 (10.89 to 37) | 3.77 (-0.17 to 7.68) |
| Portugal | 890 (452 to 1529) | 554 (286 to 967) | -37.79 (-42.7 to -32.77) | 29.87 (15.17 to 51.3) | 28.9 (14.9 to 50.48) | -3.24 (-10.89 to 4.56) |
| San Marino | 2 (1 to 3) | 2 (1 to 3) | 6.78 (-2.37 to 16.96) | 29.46 (14.83 to 50.98) | 30.17 (15.63 to 52.81) | 2.39 (-6.38 to 12.15) |
| Spain | 6609 (3725 to 10508) | 5568 (2832 to 9461) | -15.76 (-32.24 to 4.13) | 59.29 (33.42 to 94.27) | 63.22 (32.15 to 107.42) | 6.62 (-14.24 to 31.79) |
| Sweden | 507 (261 to 877) | 609 (305 to 1045) | 20.13 (5.42 to 36.08) | 24.07 (12.4 to 41.64) | 25.23 (12.64 to 43.28) | 4.8 (-8.03 to 18.71) |
| Switzerland | 548 (284 to 942) | 607 (313 to 1019) | 10.77 (-7.7 to 31.72) | 34.72 (18 to 59.75) | 34.41 (17.74 to 57.84) | -0.88 (-17.41 to 17.87) |
| United Kingdom | 3964 (2002 to 6863) | 4897 (2495 to 8239) | 23.53 (11.63 to 37.48) | 26.72 (13.5 to 46.26) | 31.28 (15.94 to 52.63) | 17.06 (5.78 to 30.27) |
| Andean Latin America | 7611 (3906 to 13080) | 9857 (5074 to 16759) | 29.5 (23.89 to 34.98) | 40.15 (20.61 to 69) | 41.64 (21.43 to 70.79) | 3.7 (-0.8 to 8.08) |
| Bolivia (Plurinational State of) | 1301 (662 to 2183) | 1867 (973 to 3123) | 43.43 (34.07 to 54.46) | 38.86 (19.76 to 65.18) | 41.06 (21.39 to 68.69) | 5.64 (-1.25 to 13.77) |
| Ecuador | 2012 (1030 to 3450) | 2775 (1443 to 4739) | 37.91 (28.61 to 47.7) | 40.59 (20.77 to 69.6) | 41.94 (21.8 to 71.63) | 3.33 (-3.63 to 10.67) |
| Peru | 4297 (2158 to 7362) | 5215 (2625 to 8915) | 21.35 (11.96 to 30.61) | 40.35 (20.27 to 69.13) | 41.68 (20.98 to 71.26) | 3.3 (-4.69 to 11.19) |
| Caribbean | 8531 (4488 to 14381) | 8929 (4679 to 15251) | 4.66 (0.76 to 8.15) | 56.5 (29.72 to 95.25) | 58.5 (30.66 to 99.93) | 3.54 (-0.32 to 6.99) |
| Antigua and Barbuda | 14 (7 to 24) | 14 (7 to 24) | 2.25 (-3.37 to 8.31) | 58.37 (30.81 to 100.38) | 61.03 (31.63 to 104.04) | 4.55 (-1.19 to 10.75) |
| Bahamas | 64 (34 to 109) | 74 (38 to 126) | 15.07 (8.53 to 21.75) | 59.56 (31.13 to 101.55) | 64.01 (33.06 to 109.47) | 7.47 (1.37 to 13.72) |
| Barbados | 51 (27 to 86) | 42 (22 to 70) | -18.6 (-23.13 to -13.54) | 60.28 (31.63 to 101.19) | 62.77 (32.72 to 105.66) | 4.12 (-1.69 to 10.59) |
| Belize | 57 (29 to 98) | 102 (53 to 171) | 78.99 (67.6 to 89.71) | 55.69 (28.57 to 95.21) | 60.48 (31.46 to 101.41) | 8.61 (1.69 to 15.11) |
| Bermuda | 9 (5 to 15) | 7 (4 to 12) | -22.54 (-27.23 to -17.83) | 57.25 (29.82 to 97) | 61.33 (32.48 to 104.05) | 7.13 (0.64 to 13.64) |
| Cuba | 2165 (1124 to 3703) | 1469 (765 to 2509) | -32.18 (-36.22 to -28.07) | 59.29 (30.78 to 101.4) | 61.16 (31.86 to 104.5) | 3.16 (-2.99 to 9.41) |
| Dominica | 19 (10 to 33) | 13 (7 to 21) | -34.71 (-38.33 to -30.93) | 58.52 (30.26 to 100.15) | 64.47 (33.63 to 110.53) | 10.15 (4.05 to 16.52) |
| Dominican Republic | 1947 (1026 to 3273) | 2248 (1180 to 3857) | 15.46 (9.07 to 23.04) | 55.59 (29.29 to 93.46) | 57.77 (30.33 to 99.14) | 3.93 (-1.82 to 10.76) |
| Grenada | 24 (12 to 41) | 18 (10 to 31) | -22.06 (-26.73 to -17.02) | 56.25 (28.88 to 96.7) | 61.4 (31.63 to 104.46) | 9.15 (2.6 to 16.2) |
| Guyana | 210 (110 to 353) | 162 (86 to 275) | -22.98 (-27.25 to -18.35) | 55 (28.89 to 92.55) | 57.58 (30.53 to 97.86) | 4.69 (-1.11 to 11) |
| Haiti | 1754 (926 to 2928) | 3136 (1637 to 5380) | 78.78 (67.78 to 89.78) | 52.41 (27.66 to 87.5) | 55.92 (29.19 to 95.93) | 6.69 (0.12 to 13.25) |
| Jamaica | 635 (328 to 1086) | 507 (264 to 863) | -20.29 (-25.06 to -15.18) | 58.17 (30.01 to 99.38) | 62.13 (32.39 to 105.85) | 6.81 (0.42 to 13.65) |
| Puerto Rico | 786 (451 to 1223) | 432 (227 to 729) | -45.11 (-56.6 to -33.38) | 59.02 (33.85 to 91.8) | 66.03 (34.73 to 111.59) | 11.89 (-11.52 to 35.79) |
| Saint Kitts and Nevis | 11 (6 to 18) | 9 (4 to 15) | -19.6 (-24.22 to -14.53) | 58.26 (30.17 to 99.45) | 61.2 (31.93 to 105.02) | 5.03 (-1 to 11.67) |
| Saint Lucia | 38 (20 to 65) | 26 (14 to 44) | -31.77 (-35.59 to -27.25) | 57.26 (29.86 to 97.77) | 62.18 (32.76 to 105.54) | 8.59 (2.51 to 15.79) |
| Saint Vincent and the Grenadines | 32 (17 to 54) | 21 (11 to 36) | -34.94 (-38.49 to -30.99) | 59.66 (31.03 to 101.11) | 61.91 (32.58 to 105.4) | 3.76 (-1.89 to 10.06) |
| Suriname | 99 (51 to 169) | 115 (60 to 197) | 15.98 (9.26 to 23.99) | 58.06 (29.9 to 98.67) | 60.64 (31.54 to 103.6) | 4.45 (-1.6 to 11.67) |
| Trinidad and Tobago | 302 (157 to 518) | 223 (116 to 381) | -26.24 (-30.81 to -21.97) | 58.19 (30.26 to 99.8) | 61.25 (31.76 to 104.65) | 5.27 (-1.25 to 11.37) |
| United States Virgin Islands | 24 (13 to 41) | 11 (6 to 19) | -53.15 (-55.94 to -50.42) | 57.88 (30.49 to 98.52) | 62.08 (31.96 to 105.1) | 7.26 (0.89 to 13.51) |
| Central Latin America | 22565 (11856 to 38108) | 24707 (13016 to 40968) | 9.49 (5.84 to 13.56) | 27.31 (14.35 to 46.12) | 28.97 (15.26 to 48.04) | 6.09 (2.55 to 10.02) |
| Colombia | 4556 (2425 to 7965) | 4565 (2460 to 7522) | 0.22 (-12.94 to 15.88) | 30.26 (16.11 to 52.91) | 31.36 (16.9 to 51.66) | 3.61 (-9.99 to 19.81) |
| Costa Rica | 426 (216 to 747) | 444 (222 to 760) | 4.29 (-3.89 to 13.28) | 30.03 (15.21 to 52.7) | 32.45 (16.21 to 55.52) | 8.03 (-0.43 to 17.35) |
| El Salvador | 830 (426 to 1429) | 750 (379 to 1298) | -9.7 (-17.09 to -2.35) | 30.14 (15.47 to 51.85) | 31.48 (15.92 to 54.47) | 4.45 (-4.09 to 12.95) |
| Guatemala | 1404 (699 to 2466) | 2108 (1067 to 3585) | 50.09 (36.73 to 63.2) | 28.6 (14.24 to 50.21) | 31.86 (16.13 to 54.19) | 11.42 (1.51 to 21.16) |
| Honduras | 795 (390 to 1375) | 1368 (693 to 2345) | 72.12 (58.39 to 87.51) | 29.3 (14.36 to 50.67) | 31.45 (15.94 to 53.91) | 7.36 (-1.21 to 16.96) |
| Mexico | 9230 (4808 to 15329) | 9836 (5088 to 16485) | 6.57 (4.18 to 9.13) | 21.28 (11.09 to 35.35) | 22.79 (11.79 to 38.2) | 7.1 (4.7 to 9.67) |
| Nicaragua | 663 (340 to 1168) | 822 (414 to 1432) | 24.04 (13.56 to 34.47) | 29.44 (15.09 to 51.89) | 31.62 (15.94 to 55.09) | 7.38 (-1.69 to 16.42) |
| Panama | 337 (172 to 591) | 478 (240 to 838) | 41.88 (31.58 to 53.68) | 31 (15.8 to 54.38) | 31.72 (15.94 to 55.61) | 2.33 (-5.1 to 10.84) |
| Venezuela (Bolivarian Republic of) | 4324 (2255 to 7096) | 4336 (2252 to 7335) | 0.26 (-6.22 to 6.67) | 47.65 (24.84 to 78.19) | 49.54 (25.74 to 83.81) | 3.98 (-2.74 to 10.62) |
| Tropical Latin America | 28494 (14704 to 48214) | 27197 (14287 to 46898) | -4.55 (-13.46 to 4.89) | 41.14 (21.23 to 69.61) | 40.84 (21.46 to 70.43) | -0.72 (-9.98 to 9.11) |
| Brazil | 27716 (14272 to 46934) | 26108 (13716 to 45289) | -5.8 (-14.87 to 3.83) | 41.25 (21.24 to 69.85) | 40.85 (21.46 to 70.86) | -0.97 (-10.5 to 9.15) |
| Paraguay | 777 (383 to 1324) | 1089 (555 to 1860) | 40.04 (30.28 to 49.85) | 37.58 (18.49 to 63.98) | 40.77 (20.76 to 69.66) | 8.49 (0.94 to 16.09) |
| North Africa and Middle East | 42267 (21432 to 72004) | 53893 (27302 to 91288) | 27.51 (21.52 to 34.04) | 23.91 (12.12 to 40.73) | 22.79 (11.54 to 38.6) | -4.69 (-9.17 to 0.19) |
| Afghanistan | 981 (491 to 1706) | 3160 (1546 to 5497) | 222.13 (188.54 to 258.72) | 17.6 (8.81 to 30.62) | 17.77 (8.69 to 30.92) | 0.99 (-9.54 to 12.46) |
| Algeria | 2505 (1265 to 4269) | 3034 (1582 to 5237) | 21.12 (8.2 to 35.3) | 18.53 (9.36 to 31.58) | 18.45 (9.62 to 31.84) | -0.47 (-11.08 to 11.18) |
| Bahrain | 36 (18 to 64) | 81 (41 to 139) | 126.84 (102.86 to 151.98) | 17.77 (8.96 to 32.07) | 19.99 (10.07 to 34.5) | 12.49 (0.59 to 24.95) |
| Egypt | 4995 (2504 to 8760) | 8677 (4352 to 15410) | 73.71 (56.35 to 92.25) | 17.89 (8.97 to 31.37) | 18.64 (9.35 to 33.1) | 4.2 (-6.22 to 15.32) |
| Iran (Islamic Republic of) | 14542 (7361 to 25005) | 12896 (6642 to 21601) | -11.32 (-20.38 to -1.76) | 46.15 (23.36 to 79.36) | 49.88 (25.69 to 83.55) | 8.08 (-2.96 to 19.73) |
| Iraq | 2021 (1015 to 3509) | 3779 (1922 to 6327) | 86.97 (57.54 to 121.26) | 19.62 (9.86 to 34.07) | 21.42 (10.9 to 35.88) | 9.18 (-8.01 to 29.2) |
| Jordan | 386 (198 to 671) | 989 (508 to 1736) | 156.19 (130.49 to 185.64) | 18.41 (9.44 to 32) | 20.07 (10.31 to 35.24) | 9.06 (-1.88 to 21.6) |
| Kuwait | 123 (65 to 215) | 214 (109 to 369) | 73.62 (56.47 to 90.91) | 17.98 (9.52 to 31.37) | 19.54 (9.95 to 33.67) | 8.72 (-2.02 to 19.54) |
| Lebanon | 246 (122 to 434) | 327 (169 to 576) | 33.06 (19.55 to 48.02) | 18.34 (9.08 to 32.39) | 19.61 (10.12 to 34.56) | 6.94 (-3.92 to 18.96) |
| Libya | 424 (211 to 736) | 426 (209 to 743) | 0.42 (-8.87 to 12.16) | 18.36 (9.14 to 31.88) | 20.38 (10 to 35.62) | 11.04 (0.77 to 24.02) |
| Morocco | 2274 (1173 to 3901) | 2478 (1231 to 4315) | 8.95 (-1.87 to 20.16) | 18.22 (9.4 to 31.26) | 19.19 (9.53 to 33.41) | 5.27 (-5.19 to 16.1) |
| Oman | 175 (90 to 300) | 273 (143 to 473) | 55.88 (40.2 to 76.75) | 17.46 (8.94 to 29.89) | 18.28 (9.57 to 31.68) | 4.73 (-5.8 to 18.75) |
| Palestine | 207 (106 to 368) | 461 (232 to 775) | 123.05 (100.08 to 150.09) | 17.33 (8.85 to 30.84) | 19.16 (9.64 to 32.2) | 10.56 (-0.82 to 23.97) |
| Qatar | 27 (14 to 47) | 106 (52 to 189) | 298.05 (253.6 to 344.68) | 17.47 (8.84 to 30.67) | 17.83 (8.72 to 31.72) | 2.05 (-9.35 to 14) |
| Saudi Arabia | 1327 (686 to 2274) | 1788 (905 to 3041) | 34.77 (19.77 to 51.33) | 16.16 (8.35 to 27.69) | 17.64 (8.93 to 30.01) | 9.21 (-2.94 to 22.63) |
| Sudan | 1945 (987 to 3377) | 4059 (2158 to 7226) | 108.72 (87.14 to 131.86) | 17.6 (8.93 to 30.57) | 18.99 (10.1 to 33.81) | 7.86 (-3.29 to 19.82) |
| Syrian Arab Republic | 1330 (677 to 2316) | 1127 (576 to 1941) | -15.3 (-24.67 to -6.26) | 18.1 (9.21 to 31.5) | 20.71 (10.59 to 35.69) | 14.46 (1.8 to 26.69) |
| Tunisia | 748 (378 to 1285) | 695 (349 to 1203) | -7.09 (-17.73 to 3.57) | 18.75 (9.47 to 32.21) | 19.38 (9.73 to 33.55) | 3.39 (-8.45 to 15.26) |
| Türkiye | 6781 (3423 to 11723) | 6627 (3438 to 11267) | -2.27 (-16.9 to 14.98) | 25.41 (12.83 to 43.94) | 26.82 (13.91 to 45.59) | 5.52 (-10.28 to 24.15) |
| United Arab Emirates | 53 (28 to 91) | 146 (72 to 248) | 178.59 (120.57 to 251.84) | 7.43 (3.92 to 12.87) | 8.63 (4.27 to 14.63) | 16.2 (-8 to 46.75) |
| Yemen | 1120 (556 to 1969) | 2501 (1217 to 4336) | 123.42 (97.43 to 151.74) | 13.32 (6.62 to 23.42) | 14.32 (6.97 to 24.82) | 7.52 (-4.99 to 21.15) |
| South Asia | 57538 (29944 to 97866) | 78275 (38853 to 132257) | 36.04 (25.19 to 47.18) | 10.61 (5.52 to 18.04) | 11.45 (5.68 to 19.35) | 7.96 (-0.65 to 16.8) |
| Bangladesh | 7875 (3942 to 13382) | 8860 (4462 to 15414) | 12.51 (-0.15 to 27.43) | 13.05 (6.53 to 22.17) | 14.53 (7.32 to 25.27) | 11.35 (-1.18 to 26.12) |
| Bhutan | 40 (20 to 68) | 31 (15 to 53) | -22.32 (-32.96 to -10.19) | 11.63 (5.83 to 19.9) | 12.06 (6.06 to 20.88) | 3.77 (-10.44 to 19.97) |
| India | 41485 (21432 to 71370) | 54747 (27053 to 92927) | 31.97 (17.6 to 47.35) | 10.11 (5.22 to 17.39) | 10.94 (5.4 to 18.56) | 8.2 (-3.58 to 20.81) |
| Nepal | 1123 (569 to 1943) | 1487 (760 to 2522) | 32.43 (15.65 to 50.63) | 10.85 (5.5 to 18.77) | 11.94 (6.11 to 20.25) | 10.04 (-3.9 to 25.17) |
| Pakistan | 7015 (3553 to 12420) | 13150 (6860 to 23531) | 87.45 (72.49 to 102.21) | 11.51 (5.83 to 20.37) | 12.04 (6.28 to 21.54) | 4.62 (-3.72 to 12.87) |
| East Asia | 175894 (91414 to 296914) | 160363 (85161 to 272639) | -8.83 (-15.97 to -0.48) | 38.23 (19.87 to 64.53) | 46.49 (24.69 to 79.04) | 21.61 (12.09 to 32.76) |
| China | 172257 (89521 to 290847) | 157553 (83629 to 268081) | -8.54 (-15.87 to 0.07) | 38.71 (20.12 to 65.35) | 47.13 (25.02 to 80.19) | 21.76 (12 to 33.22) |
| Democratic People's Republic of Korea | 2038 (1048 to 3454) | 1932 (994 to 3300) | -5.19 (-13.57 to 3.34) | 26.16 (13.46 to 44.34) | 29.26 (15.06 to 49.98) | 11.85 (1.97 to 21.92) |
| Taiwan (Province of China) | 1600 (833 to 2784) | 878 (448 to 1520) | -45.1 (-50.28 to -39.25) | 21.89 (11.4 to 38.1) | 21.71 (11.09 to 37.57) | -0.81 (-10.18 to 9.76) |
| Southeast Asia | 41668 (21140 to 70660) | 42070 (20872 to 71570) | 0.96 (-4.58 to 5.07) | 18.95 (9.61 to 32.13) | 18.35 (9.1 to 31.22) | -3.16 (-8.48 to 0.77) |
| Cambodia | 873 (433 to 1508) | 1117 (565 to 1935) | 27.95 (14.5 to 42.93) | 15.33 (7.6 to 26.49) | 16.84 (8.52 to 29.19) | 9.86 (-1.69 to 22.72) |
| Indonesia | 15280 (7592 to 26907) | 16127 (8029 to 28166) | 5.54 (2.07 to 9.16) | 17.47 (8.68 to 30.76) | 17.88 (8.9 to 31.22) | 2.32 (-1.04 to 5.83) |
| Lao People's Democratic Republic | 353 (174 to 625) | 493 (248 to 870) | 39.75 (25.99 to 56.51) | 15.56 (7.65 to 27.56) | 16.49 (8.3 to 29.09) | 5.98 (-4.46 to 18.68) |
| Malaysia | 651 (324 to 1112) | 885 (441 to 1542) | 35.84 (11.32 to 61.17) | 7.81 (3.89 to 13.32) | 8.63 (4.3 to 15.04) | 10.51 (-9.44 to 31.12) |
| Maldives | 20 (10 to 36) | 23 (11 to 40) | 14.91 (1.45 to 29.19) | 15.38 (7.75 to 27.67) | 17.41 (8.75 to 30.83) | 13.18 (-0.08 to 27.25) |
| Mauritius | 74 (37 to 129) | 52 (26 to 90) | -29.29 (-36.92 to -20.86) | 17.23 (8.51 to 29.95) | 17.68 (8.69 to 30.45) | 2.6 (-8.47 to 14.83) |
| Myanmar | 3175 (1567 to 5503) | 3524 (1756 to 6011) | 10.99 (-1.44 to 24.58) | 16.61 (8.2 to 28.79) | 16.97 (8.46 to 28.94) | 2.14 (-9.3 to 14.64) |
| Philippines | 5345 (2673 to 9353) | 7927 (3938 to 13916) | 48.31 (45.27 to 51.42) | 16.68 (8.34 to 29.18) | 17.7 (8.79 to 31.08) | 6.16 (3.99 to 8.39) |
| Seychelles | 5 (3 to 9) | 5 (3 to 9) | -1.82 (-13 to 9.63) | 16.85 (8.55 to 28.83) | 16.93 (8.42 to 29.21) | 0.48 (-10.97 to 12.19) |
| Sri Lanka | 1245 (623 to 2166) | 1214 (613 to 2109) | -2.48 (-13.14 to 9.3) | 17.19 (8.61 to 29.92) | 17.59 (8.88 to 30.57) | 2.33 (-8.86 to 14.69) |
| Thailand | 9002 (4723 to 14693) | 5123 (2601 to 8762) | -43.08 (-54.32 to -32.2) | 39.57 (20.77 to 64.59) | 37.54 (19.06 to 64.2) | -5.14 (-23.86 to 13.01) |
| Timor-Leste | 61 (30 to 109) | 115 (57 to 201) | 87.23 (67.98 to 109.34) | 15.09 (7.29 to 26.72) | 16.76 (8.36 to 29.31) | 11.08 (-0.34 to 24.2) |
| Viet Nam | 5523 (2771 to 9384) | 5406 (2705 to 9451) | -2.12 (-13.93 to 10.36) | 16.41 (8.23 to 27.87) | 17.1 (8.56 to 29.89) | 4.22 (-8.35 to 17.51) |
| Oceania | 698 (360 to 1201) | 1320 (670 to 2284) | 89.04 (73.86 to 104.17) | 20.75 (10.68 to 35.68) | 20.67 (10.49 to 35.77) | -0.35 (-8.35 to 7.62) |
| American Samoa | 5 (3 to 8) | 5 (2 to 8) | -6.34 (-16.22 to 4.27) | 20.3 (10.52 to 34.93) | 23.78 (12.08 to 41.31) | 17.15 (4.79 to 30.42) |
| Cook Islands | 2 (1 to 3) | 1 (1 to 2) | -37.21 (-42.5 to -30.18) | 21.88 (11.24 to 37.8) | 22.83 (11.67 to 38.68) | 4.32 (-4.48 to 15.99) |
| Fiji | 78 (40 to 134) | 76 (38 to 133) | -1.58 (-11.12 to 8.17) | 21.61 (11.05 to 37.3) | 21.76 (10.96 to 38.02) | 0.69 (-9.07 to 10.66) |
| Guam | 11 (5 to 19) | 11 (5 to 18) | -6.32 (-15.77 to 3.41) | 20.72 (10.07 to 35.63) | 21.68 (10.72 to 37.26) | 4.61 (-5.94 to 15.48) |
| Kiribati | 7 (4 to 13) | 11 (6 to 20) | 57.23 (41.22 to 74.35) | 19.88 (10.13 to 34.41) | 21.44 (10.92 to 37.35) | 7.83 (-3.15 to 19.57) |
| Marshall Islands | 6 (3 to 10) | 5 (3 to 9) | -10.25 (-18.98 to -0.68) | 21.2 (10.98 to 37.37) | 22.09 (11.06 to 38.21) | 4.18 (-5.95 to 15.29) |
| Micronesia (Federated States of) | 12 (6 to 21) | 9 (5 to 16) | -24.13 (-31.51 to -15.72) | 21.52 (10.92 to 36.92) | 22.65 (11.63 to 39.45) | 5.27 (-4.98 to 16.92) |
| Nauru | 1 (1 to 2) | 1 (1 to 2) | 5.25 (-4.63 to 15.37) | 20.15 (10.12 to 34.78) | 21.54 (10.97 to 36.67) | 6.92 (-3.12 to 17.19) |
| Niue | 0 (0 to 0) | 0 (0 to 0) | -46.16 (-51.19 to -40.72) | 22.23 (11.48 to 38.37) | 23.17 (11.85 to 39.58) | 4.23 (-5.51 to 14.77) |
| Northern Mariana Islands | 3 (2 to 6) | 3 (2 to 6) | 7.13 (-2.78 to 18.72) | 20.13 (10.43 to 35.12) | 23.32 (11.87 to 40.69) | 15.84 (5.13 to 28.38) |
| Palau | 1 (1 to 2) | 1 (1 to 2) | -25.64 (-32.29 to -17.49) | 22.37 (11.26 to 38.16) | 23.16 (12.05 to 39.24) | 3.53 (-5.73 to 14.87) |
| Papua New Guinea | 438 (227 to 755) | 1003 (510 to 1739) | 128.98 (104.5 to 153.76) | 20.58 (10.65 to 35.45) | 20.48 (10.41 to 35.49) | -0.51 (-11.15 to 10.26) |
| Samoa | 20 (10 to 34) | 21 (11 to 36) | 6.58 (-3.47 to 17.93) | 21.68 (10.78 to 37.3) | 20.97 (10.87 to 35.91) | -3.29 (-12.41 to 7.01) |
| Solomon Islands | 40 (21 to 70) | 70 (36 to 121) | 74.01 (57.3 to 90.27) | 20.55 (10.56 to 36.04) | 21.04 (10.79 to 36.5) | 2.39 (-7.44 to 11.96) |
| Tokelau | 0 (0 to 0) | 0 (0 to 0) | -25.89 (-32.85 to -18.05) | 21.77 (10.88 to 38.68) | 23.92 (12.4 to 41.36) | 9.87 (-0.45 to 21.49) |
| Tonga | 11 (6 to 19) | 10 (5 to 18) | -7.96 (-16.32 to 0.96) | 21.19 (10.9 to 36.04) | 20.99 (10.89 to 36.05) | -0.94 (-9.94 to 8.66) |
| Tuvalu | 1 (0 to 1) | 1 (1 to 2) | 31.5 (20.01 to 46.28) | 19.24 (9.83 to 32.97) | 22.01 (11.08 to 38.26) | 14.37 (4.39 to 27.23) |
| Vanuatu | 17 (9 to 29) | 31 (16 to 53) | 85.06 (66.92 to 105.31) | 20.07 (10.63 to 34.17) | 21.03 (10.62 to 36.19) | 4.74 (-5.52 to 16.2) |
| Central sub-Saharan Africa | 2787 (1421 to 4733) | 7136 (3644 to 12316) | 156.1 (131.93 to 184.56) | 8.99 (4.58 to 15.27) | 9.7 (4.95 to 16.74) | 7.88 (-2.3 to 19.87) |
| Angola | 518 (260 to 893) | 1763 (893 to 3029) | 240.04 (190.96 to 293.76) | 8.99 (4.51 to 15.49) | 9.44 (4.78 to 16.22) | 4.99 (-10.17 to 21.57) |
| Central African Republic | 135 (68 to 233) | 278 (139 to 483) | 106.52 (75.29 to 138.25) | 8.99 (4.57 to 15.55) | 9.61 (4.79 to 16.67) | 6.84 (-9.32 to 23.25) |
| Congo | 125 (65 to 216) | 252 (129 to 415) | 101.17 (70.19 to 135.8) | 9.43 (4.87 to 16.25) | 10.12 (5.18 to 16.68) | 7.32 (-9.21 to 25.79) |
| Democratic Republic of the Congo | 1940 (1000 to 3328) | 4679 (2351 to 8129) | 141.22 (110.76 to 176.57) | 8.96 (4.62 to 15.37) | 9.77 (4.91 to 16.97) | 9.01 (-4.75 to 24.99) |
| Equatorial Guinea | 21 (11 to 36) | 83 (41 to 144) | 290.45 (238 to 352.8) | 8.9 (4.46 to 14.91) | 10.83 (5.31 to 18.65) | 21.67 (5.33 to 41.1) |
| Gabon | 47 (24 to 82) | 81 (41 to 138) | 72.07 (50.62 to 100.17) | 9.32 (4.73 to 16.06) | 9.85 (5.02 to 16.64) | 5.73 (-7.45 to 22.99) |
| Eastern sub-Saharan Africa | 10167 (5168 to 17685) | 22332 (11524 to 38475) | 119.66 (111.26 to 128.14) | 9.17 (4.66 to 15.95) | 9.81 (5.06 to 16.91) | 7.03 (2.94 to 11.17) |
| Burundi | 280 (138 to 472) | 689 (344 to 1209) | 145.93 (112.51 to 183.01) | 8.88 (4.36 to 14.95) | 9.47 (4.73 to 16.62) | 6.66 (-7.83 to 22.75) |
| Comoros | 25 (12 to 43) | 32 (16 to 56) | 28.25 (10.41 to 48.14) | 9.37 (4.69 to 16.26) | 10.11 (5.04 to 17.83) | 7.98 (-7.04 to 24.73) |
| Djibouti | 22 (11 to 38) | 54 (27 to 93) | 144.43 (113.4 to 182.13) | 9.87 (4.94 to 16.85) | 10.24 (5.13 to 17.54) | 3.75 (-9.42 to 19.75) |
| Eritrea | 184 (90 to 314) | 315 (162 to 538) | 71.15 (47.52 to 101.11) | 9.35 (4.6 to 15.98) | 9.78 (5.03 to 16.73) | 4.65 (-9.8 to 22.97) |
| Ethiopia | 2731 (1414 to 4810) | 5699 (2915 to 10046) | 108.68 (92.02 to 127.39) | 9.27 (4.8 to 16.32) | 9.96 (5.1 to 17.56) | 7.51 (-1.07 to 17.15) |
| Kenya | 1302 (679 to 2274) | 2576 (1336 to 4513) | 97.83 (92.2 to 103.12) | 9.44 (4.93 to 16.49) | 10.47 (5.43 to 18.35) | 10.91 (7.75 to 13.88) |
| Madagascar | 616 (306 to 1055) | 1464 (731 to 2561) | 137.84 (102.65 to 178.17) | 9.15 (4.55 to 15.69) | 9.8 (4.89 to 17.14) | 7.05 (-8.79 to 25.2) |
| Malawi | 495 (246 to 866) | 1055 (526 to 1798) | 113.25 (86.26 to 145.96) | 8.87 (4.41 to 15.54) | 9.96 (4.96 to 16.98) | 12.22 (-1.98 to 29.43) |
| Mozambique | 691 (350 to 1198) | 1691 (841 to 2913) | 144.78 (113.02 to 186.69) | 9.13 (4.63 to 15.82) | 9.53 (4.74 to 16.41) | 4.38 (-9.16 to 22.25) |
| Rwanda | 375 (189 to 642) | 634 (326 to 1067) | 69.38 (47.04 to 92.72) | 9.05 (4.56 to 15.52) | 9.85 (5.06 to 16.58) | 8.88 (-5.48 to 23.89) |
| Somalia | 447 (223 to 764) | 1186 (610 to 2034) | 165.19 (129.16 to 209.94) | 9.45 (4.71 to 16.15) | 9.28 (4.77 to 15.91) | -1.83 (-15.17 to 14.73) |
| South Sudan | 313 (156 to 541) | 540 (269 to 924) | 72.63 (49.15 to 98.74) | 9.51 (4.74 to 16.47) | 9.84 (4.9 to 16.84) | 3.38 (-10.68 to 19.02) |
| Uganda | 904 (454 to 1642) | 2408 (1205 to 4191) | 166.29 (131.83 to 206.36) | 8.78 (4.41 to 15.94) | 9.65 (4.83 to 16.8) | 9.94 (-4.29 to 26.48) |
| United Republic of Tanzania | 1349 (692 to 2279) | 2960 (1527 to 5040) | 119.4 (89.37 to 155.85) | 9.04 (4.63 to 15.27) | 9.59 (4.95 to 16.33) | 6.1 (-8.42 to 23.73) |
| Zambia | 426 (222 to 739) | 1010 (509 to 1733) | 136.74 (103.31 to 176.44) | 9.07 (4.72 to 15.71) | 9.66 (4.87 to 16.58) | 6.44 (-8.59 to 24.29) |
| Southern sub-Saharan Africa | 2576 (1314 to 4466) | 3181 (1628 to 5547) | 23.49 (17.27 to 30.79) | 9.74 (4.97 to 16.88) | 10.18 (5.21 to 17.74) | 4.52 (-0.75 to 10.69) |
| Botswana | 71 (35 to 123) | 93 (47 to 165) | 31.04 (13.78 to 51.58) | 9.61 (4.74 to 16.65) | 10.16 (5.14 to 17.99) | 5.81 (-8.13 to 22.4) |
| Eswatini | 45 (22 to 76) | 54 (26 to 95) | 22.02 (5.85 to 38.91) | 9.33 (4.54 to 15.88) | 10.15 (4.91 to 17.75) | 8.81 (-5.6 to 23.88) |
| Lesotho | 79 (40 to 139) | 86 (42 to 151) | 8.52 (-6.91 to 23.79) | 9.49 (4.83 to 16.6) | 10.28 (5.03 to 17.95) | 8.33 (-7.08 to 23.56) |
| Namibia | 72 (36 to 128) | 107 (56 to 179) | 48.54 (29.31 to 70.58) | 9.49 (4.76 to 16.76) | 10.02 (5.2 to 16.74) | 5.6 (-8.07 to 21.27) |
| South Africa | 1736 (897 to 3053) | 2057 (1067 to 3607) | 18.48 (11.91 to 25.62) | 9.85 (5.09 to 17.32) | 10.33 (5.36 to 18.11) | 4.82 (-0.99 to 11.14) |
| Zimbabwe | 573 (291 to 950) | 783 (395 to 1401) | 36.76 (16.31 to 60.49) | 9.51 (4.83 to 15.78) | 9.81 (4.95 to 17.55) | 3.16 (-12.27 to 21.06) |
| Western sub-Saharan Africa | 9998 (5038 to 17184) | 27012 (13543 to 45474) | 170.19 (154.77 to 186.66) | 9.3 (4.69 to 15.99) | 10.06 (5.04 to 16.93) | 8.14 (1.97 to 14.73) |
| Benin | 264 (132 to 466) | 722 (355 to 1235) | 173.07 (131.06 to 220.36) | 9.21 (4.6 to 16.24) | 9.6 (4.73 to 16.42) | 4.26 (-11.78 to 22.31) |
| Burkina Faso | 525 (261 to 903) | 1206 (585 to 2071) | 129.91 (99.46 to 161.24) | 9.27 (4.6 to 15.97) | 9.42 (4.57 to 16.17) | 1.57 (-11.89 to 15.41) |
| Cabo Verde | 19 (9 to 33) | 21 (10 to 36) | 10.16 (-2.56 to 26.76) | 9.68 (4.81 to 17.21) | 10.75 (5.18 to 18.68) | 11.15 (-1.69 to 27.9) |
| Cameroon | 549 (270 to 935) | 1681 (845 to 2856) | 205.93 (166.06 to 258.66) | 9.25 (4.56 to 15.74) | 9.94 (5 to 16.9) | 7.46 (-6.55 to 25.98) |
| Chad | 316 (157 to 549) | 1016 (522 to 1802) | 221.65 (178.46 to 269.78) | 8.96 (4.46 to 15.57) | 9.28 (4.76 to 16.46) | 3.56 (-10.35 to 19.05) |
| Côte d'Ivoire | 642 (311 to 1134) | 1420 (723 to 2469) | 121.01 (90.74 to 157.6) | 9.28 (4.49 to 16.4) | 9.87 (5.02 to 17.16) | 6.3 (-8.26 to 23.9) |
| Gambia | 52 (26 to 92) | 126 (61 to 221) | 142.06 (111.41 to 183.06) | 9.28 (4.55 to 16.3) | 9.97 (4.78 to 17.42) | 7.36 (-6.23 to 25.55) |
| Ghana | 791 (394 to 1372) | 1637 (806 to 2834) | 106.97 (77 to 141.03) | 9.58 (4.77 to 16.61) | 10.04 (4.94 to 17.37) | 4.79 (-10.38 to 22.03) |
| Guinea | 297 (144 to 508) | 729 (354 to 1284) | 145.14 (110.21 to 184.48) | 9.05 (4.39 to 15.47) | 9.77 (4.74 to 17.2) | 7.95 (-7.43 to 25.28) |
| Guinea-Bissau | 55 (27 to 98) | 110 (55 to 186) | 98.62 (73.09 to 126.63) | 9.39 (4.64 to 16.71) | 9.8 (4.94 to 16.59) | 4.43 (-8.99 to 19.16) |
| Liberia | 125 (62 to 212) | 282 (138 to 501) | 125.45 (95.33 to 159.84) | 9.14 (4.54 to 15.52) | 10.09 (4.95 to 17.97) | 10.4 (-4.35 to 27.23) |
| Mali | 450 (219 to 799) | 1347 (682 to 2361) | 199.16 (153.87 to 249.64) | 9.1 (4.43 to 16.14) | 9.46 (4.79 to 16.59) | 3.95 (-11.78 to 21.5) |
| Mauritania | 107 (53 to 187) | 233 (117 to 396) | 119.03 (87.24 to 156.04) | 9.39 (4.68 to 16.51) | 10 (5.03 to 16.99) | 6.5 (-8.96 to 24.49) |
| Niger | 436 (208 to 750) | 1464 (707 to 2510) | 235.88 (191.99 to 283.2) | 8.99 (4.29 to 15.46) | 9.39 (4.54 to 16.1) | 4.55 (-9.11 to 19.28) |
| Nigeria | 4551 (2302 to 7970) | 13316 (6826 to 22964) | 192.57 (160.43 to 225.41) | 9.38 (4.74 to 16.43) | 10.42 (5.34 to 17.97) | 11.1 (-1.11 to 23.57) |
| Sao Tome and Principe | 7 (3 to 12) | 11 (5 to 19) | 55.19 (34.67 to 78.55) | 9.88 (4.93 to 17.4) | 10.53 (5.12 to 18.51) | 6.53 (-7.56 to 22.57) |
| Senegal | 408 (203 to 708) | 824 (410 to 1454) | 101.83 (73.83 to 135.35) | 9.19 (4.57 to 15.92) | 10.16 (5.06 to 17.92) | 10.6 (-4.74 to 28.96) |
| Sierra Leone | 198 (97 to 344) | 446 (222 to 774) | 125.22 (95.2 to 162.92) | 8.98 (4.41 to 15.61) | 9.8 (4.88 to 17.01) | 9.16 (-5.38 to 27.44) |
| Togo | 204 (99 to 363) | 423 (203 to 718) | 107.16 (78.61 to 138.06) | 9.48 (4.59 to 16.88) | 10.12 (4.87 to 17.2) | 6.8 (-7.91 to 22.74) |

Abbreviations: UI, uncertainty interval.

**eTable** **5a. Prevalence of ADHD by region and year, <20 years, 1990-2021.**

| **Regions** | 1990 | 1991 | 1992 | 1993 | 1994 | 1995 | 1996 | 1997 |
| --- | --- | --- | --- | --- | --- | --- | --- | --- |
| Global | 1892.47 (1297.06 to 2721.83) | 1908.99 (1308.03 to 2749.59) | 1924.56 (1318.59 to 2769.77) | 1938.14 (1328.03 to 2778.9) | 1949.55 (1336.25 to 2803.09) | 1957.34 (1342.27 to 2810.31) | 1962.58 (1342.56 to 2812.16) | 1967.32 (1343.62 to 2827.54) |
| High-income Asia Pacific | 2379.39 (1606.51 to 3424.06) | 2379.66 (1610.5 to 3404.79) | 2375.8 (1618.04 to 3397.65) | 2368.53 (1622.74 to 3391.16) | 2356.73 (1620.36 to 3378.89) | 2343.31 (1621.76 to 3364.64) | 2331.16 (1612.5 to 3355.54) | 2321.38 (1604.13 to 3350.15) |
| High-income North America | 3213.33 (2167.01 to 4646.08) | 3265.56 (2211.22 to 4706.03) | 3318.77 (2249.42 to 4762.08) | 3371.41 (2277.95 to 4867.09) | 3419.64 (2313.05 to 4982.07) | 3457.03 (2340.74 to 5035.07) | 3485.49 (2358.55 to 5082.22) | 3508.55 (2369.72 to 5126.51) |
| Western Europe | 2418.14 (1685.04 to 3380.09) | 2401.94 (1671.29 to 3358.25) | 2386.81 (1658.61 to 3338.67) | 2374.23 (1647.83 to 3326.57) | 2364.62 (1644.59 to 3314.25) | 2358.05 (1642.92 to 3305.52) | 2356.93 (1651.96 to 3286.94) | 2361.76 (1666.93 to 3281.78) |
| Australasia | 5136.04 (3584.79 to 7170.58) | 5125.1 (3572.38 to 7171.18) | 5115.39 (3561.44 to 7172.83) | 5112.13 (3555.51 to 7195.17) | 5116.12 (3554.73 to 7220.08) | 5128.18 (3559.52 to 7253.38) | 5145.44 (3575.41 to 7279.71) | 5166.66 (3594.81 to 7308.24) |
| Andean Latin America | 3283.29 (2216.95 to 4752.37) | 3285.48 (2218.61 to 4755.22) | 3286.27 (2219.16 to 4756.01) | 3286.76 (2219.52 to 4756.36) | 3288.93 (2221.02 to 4759.2) | 3293.74 (2224.31 to 4765.94) | 3300.65 (2229.02 to 4775.79) | 3309.33 (2234.93 to 4788.28) |
| Tropical Latin America | 3370.88 (2318.63 to 4963.22) | 3297.46 (2265.74 to 4799.88) | 3230.59 (2208.36 to 4654.06) | 3175.13 (2199.26 to 4550.12) | 3136.74 (2177.83 to 4478.9) | 3121.59 (2155 to 4482.88) | 3124.3 (2170.97 to 4474.31) | 3133.1 (2159.81 to 4493.38) |
| Central Latin America | 2228.56 (1516.62 to 3207.3) | 2224.81 (1521.49 to 3214.37) | 2221.93 (1520 to 3219.62) | 2220.95 (1516.81 to 3215.62) | 2222.9 (1514.47 to 3209.91) | 2228.3 (1511.82 to 3207.75) | 2235.75 (1517.07 to 3217.77) | 2243.86 (1522.72 to 3228.8) |
| Southern Latin America | 2150.09 (1439.05 to 3161.3) | 2149.69 (1438.88 to 3160.22) | 2152.8 (1440.99 to 3164.66) | 2158.56 (1444.92 to 3172.96) | 2165.49 (1449.69 to 3182.83) | 2173.35 (1455.08 to 3194.1) | 2181.39 (1460.58 to 3205.59) | 2188.64 (1465.53 to 3215.85) |
| Caribbean | 4629.48 (3229.95 to 6539.97) | 4619.34 (3223.19 to 6530.72) | 4617.86 (3222.58 to 6534.7) | 4625.45 (3228.3 to 6551.71) | 4640.11 (3238.88 to 6578.31) | 4658.34 (3251.79 to 6609.08) | 4675.38 (3265.69 to 6626.39) | 4689.73 (3277.34 to 6638.7) |
| Central Europe | 1675.98 (1142.48 to 2414.33) | 1685.2 (1149.8 to 2426.68) | 1694.89 (1157.41 to 2439.71) | 1704.73 (1165 to 2453.08) | 1714.96 (1172.71 to 2467.23) | 1725.27 (1180.28 to 2481.69) | 1734.36 (1186.85 to 2494.45) | 1742.46 (1192.73 to 2505.72) |
| Eastern Europe | 1651.63 (1103.77 to 2401.57) | 1675.24 (1119.55 to 2435.84) | 1704.35 (1138.97 to 2478.16) | 1736.24 (1160.27 to 2524.65) | 1767.05 (1180.92 to 2569.76) | 1794.76 (1199.58 to 2610.51) | 1819.13 (1216.09 to 2646.57) | 1839.03 (1229.81 to 2676.36) |
| Central Asia | 1504.09 (1022.24 to 2202.53) | 1510.46 (1026.49 to 2211.91) | 1520.01 (1032.89 to 2225.96) | 1532.51 (1041.3 to 2244.36) | 1547.59 (1051.49 to 2266.52) | 1565.22 (1063.45 to 2292.4) | 1584.63 (1076.65 to 2320.87) | 1604.59 (1090.29 to 2350.09) |
| North Africa and Middle East | 1953.68 (1348.22 to 2809.83) | 1970.71 (1361.71 to 2833.16) | 1984.01 (1372.42 to 2852.27) | 1989.34 (1377.55 to 2859.99) | 1989.61 (1379.08 to 2860.5) | 1990.92 (1381.04 to 2862.57) | 2005.73 (1391.62 to 2880.6) | 2036.18 (1412.38 to 2925.45) |
| South Asia | 872.07 (575.77 to 1275.44) | 864.24 (567.19 to 1261.69) | 857.29 (562.17 to 1250.58) | 851.55 (558.5 to 1246.42) | 847.54 (559.04 to 1244.5) | 845.81 (561.72 to 1249.7) | 845.2 (564.69 to 1243.18) | 844.58 (563.34 to 1236.83) |
| Southeast Asia | 1546.52 (1055.92 to 2209.97) | 1546.64 (1056.19 to 2210.29) | 1546.29 (1056.13 to 2209.92) | 1545.74 (1055.94 to 2209.23) | 1545.08 (1055.67 to 2208.37) | 1544.33 (1055.33 to 2207.38) | 1543.56 (1054.99 to 2206.75) | 1543.05 (1054.78 to 2206.61) |
| East Asia | 3111.27 (2153.42 to 4473.04) | 3240.35 (2235.79 to 4660.3) | 3369.94 (2326.36 to 4828.41) | 3494.47 (2405.3 to 5032.8) | 3607.34 (2474.6 to 5172) | 3696.87 (2528.98 to 5301.13) | 3765.69 (2563.63 to 5393.9) | 3826.01 (2602.74 to 5488.42) |
| Oceania | 1698.78 (1170.33 to 2480.96) | 1699.9 (1171.13 to 2482.73) | 1700.75 (1171.76 to 2484.14) | 1701.23 (1172.13 to 2485.02) | 1701.45 (1172.32 to 2485.52) | 1701.61 (1172.48 to 2485.92) | 1701.86 (1172.69 to 2486.44) | 1702.41 (1173.1 to 2487.33) |
| Western Sub-Saharan Africa | 765.62 (509.56 to 1152.89) | 767.6 (510.89 to 1155.92) | 769.46 (512.14 to 1158.78) | 771.16 (513.29 to 1161.41) | 772.81 (514.41 to 1163.83) | 774.33 (515.45 to 1166) | 775.63 (516.33 to 1167.84) | 776.74 (517.09 to 1169.39) |
| Eastern Sub-Saharan Africa | 755.95 (503.9 to 1113.73) | 757.9 (505.3 to 1117.37) | 759.53 (506.5 to 1120.22) | 760.91 (507.53 to 1122.26) | 761.37 (507.89 to 1122.97) | 761.4 (508 to 1123.01) | 761.36 (508.07 to 1123.03) | 761.18 (508.01 to 1122.76) |
| Central Sub-Saharan Africa | 740.75 (496.68 to 1091.21) | 741.74 (497.32 to 1092.68) | 742.8 (498.02 to 1094.25) | 743.83 (498.71 to 1095.78) | 744.93 (499.44 to 1097.41) | 746.08 (500.2 to 1099.12) | 747.03 (500.83 to 1100.52) | 747.79 (501.33 to 1101.66) |
| Southern Sub-Saharan Africa | 798.04 (532.5 to 1180.68) | 801.67 (534.97 to 1186.05) | 805.29 (537.4 to 1191.4) | 808.82 (539.75 to 1196.63) | 812.2 (541.99 to 1201.66) | 815.5 (544.18 to 1206.24) | 818.84 (546.41 to 1210.77) | 822.28 (548.73 to 1215.38) |

| 1998 | 1999 | 2000 | 2001 | 2002 | 2003 | 2004 | 2005 |
| --- | --- | --- | --- | --- | --- | --- | --- |
| 1970.1 (1345.04 to 2841.19) | 1969.47 (1344.16 to 2847.14) | 1964.34 (1340.14 to 2835.92) | 1955.11 (1335.64 to 2813.56) | 1942.67 (1329.04 to 2782.12) | 1927.02 (1320.1 to 2739.38) | 1908.99 (1309.2 to 2698.79) | 1889.62 (1297.03 to 2675.28) |
| 2313.22 (1593.41 to 3346.88) | 2306.91 (1584 to 3343.15) | 2302.83 (1576.4 to 3338.72) | 2300.47 (1579.6 to 3331.65) | 2301.05 (1585.45 to 3317.21) | 2304.14 (1591.29 to 3307.81) | 2309.98 (1595.44 to 3304.8) | 2319.7 (1600.24 to 3309.95) |
| 3525.8 (2390.89 to 5136.47) | 3539.23 (2418.58 to 5147.96) | 3549.16 (2400.32 to 5191.73) | 3560.85 (2412.81 to 5172.97) | 3575.33 (2409.59 to 5144.39) | 3587.81 (2393.89 to 5111.34) | 3595.95 (2403.83 to 5103.63) | 3595.45 (2415.27 to 5098.61) |
| 2369.82 (1684.34 to 3279.1) | 2376.62 (1699.42 to 3277.34) | 2380.34 (1704.38 to 3275.17) | 2385.35 (1708.9 to 3279.5) | 2395.84 (1714.92 to 3302.57) | 2406.8 (1719.09 to 3328.12) | 2414.82 (1721.92 to 3348.67) | 2417 (1723.75 to 3358.02) |
| 5191.01 (3617.39 to 7338.13) | 5217.07 (3644.14 to 7375.43) | 5241.09 (3669.69 to 7413.68) | 5268.86 (3735.75 to 7337.39) | 5303.2 (3903.92 to 7275.58) | 5333.75 (4004 to 7166.57) | 5356.26 (4041.61 to 7099.22) | 5361.83 (4030.82 to 7020.59) |
| 3319.54 (2241.86 to 4803.02) | 3330.52 (2249.3 to 4818.82) | 3342.04 (2257.08 to 4835.33) | 3354.11 (2265.19 to 4852.59) | 3367.07 (2273.9 to 4871.1) | 3380.88 (2283.17 to 4890.72) | 3394.13 (2292.09 to 4909.43) | 3405.07 (2299.48 to 4924.68) |
| 3144.97 (2167.47 to 4508.6) | 3156.7 (2171.96 to 4574.5) | 3165.21 (2180.89 to 4596.31) | 3174.68 (2194.31 to 4620.58) | 3189.8 (2193.74 to 4595.87) | 3207.61 (2200.55 to 4599.65) | 3224.5 (2213.58 to 4628.32) | 3236.37 (2240.8 to 4691.46) |
| 2252.91 (1529.02 to 3241.33) | 2262.89 (1535.95 to 3255.37) | 2273.59 (1543.38 to 3270.57) | 2283.93 (1550.13 to 3281.96) | 2292.92 (1559.35 to 3289.4) | 2300.49 (1571.09 to 3295.04) | 2307.05 (1583.82 to 3299.39) | 2313.46 (1594.49 to 3308.01) |
| 2195.5 (1470.11 to 3225.81) | 2202.4 (1474.59 to 3236.13) | 2209.44 (1479.14 to 3246.74) | 2215.34 (1477.73 to 3239.74) | 2218.95 (1482.42 to 3231.39) | 2219.89 (1492.71 to 3219.27) | 2219.3 (1501.17 to 3209.49) | 2219.55 (1501.55 to 3215.8) |
| 4703.36 (3287.55 to 6648.4) | 4717.77 (3298.24 to 6657.71) | 4733.67 (3309.88 to 6668.12) | 4750.2 (3317.33 to 6693.43) | 4765.82 (3324.01 to 6717.61) | 4780.24 (3329.91 to 6740.18) | 4793.41 (3335.14 to 6761.09) | 4804.81 (3339.53 to 6779.65) |
| 1749.2 (1197.72 to 2514.92) | 1753.69 (1201.21 to 2520.75) | 1755.8 (1203.18 to 2523.05) | 1756.13 (1203.93 to 2522.83) | 1754.64 (1203.45 to 2519.97) | 1750.68 (1201.21 to 2513.54) | 1743.88 (1196.86 to 2503.17) | 1734.01 (1190.22 to 2488.64) |
| 1853.19 (1239.97 to 2697.99) | 1862.22 (1246.88 to 2710.98) | 1865.4 (1250.63 to 2713.18) | 1861.05 (1249.41 to 2707.96) | 1848.63 (1241.56 to 2691.89) | 1829.44 (1228.79 to 2665.69) | 1806.47 (1213.42 to 2633.55) | 1781.54 (1196.69 to 2597.73) |
| 1623.65 (1103.4 to 2377.87) | 1640.86 (1115.33 to 2401.97) | 1656.04 (1125.92 to 2421.07) | 1667.94 (1134.35 to 2434.8) | 1675.55 (1139.72 to 2441.99) | 1678.39 (1141.55 to 2442.31) | 1676.62 (1140.09 to 2436.33) | 1670.34 (1135.64 to 2424.34) |
| 2069.53 (1434.75 to 2976.38) | 2093.66 (1455.02 to 3010.7) | 2098.7 (1463.8 to 3014.98) | 2087.38 (1455.11 to 3004.27) | 2068.15 (1436.85 to 2980.62) | 2044.04 (1415.8 to 2945.38) | 2020.81 (1404.55 to 2912.26) | 2000.23 (1390.1 to 2883.27) |
| 844.43 (560.29 to 1226.95) | 845.09 (557.06 to 1233.5) | 846.79 (555.99 to 1238.52) | 850.74 (560.99 to 1238.83) | 856.8 (569.44 to 1247.67) | 863.35 (576.17 to 1261.07) | 868.79 (582.84 to 1276.78) | 871.63 (587.28 to 1282.12) |
| 1542.78 (1054.71 to 2206.86) | 1542.39 (1054.58 to 2206.92) | 1541.67 (1054.24 to 2206.41) | 1540.65 (1053.7 to 2205.09) | 1539.33 (1052.99 to 2203.34) | 1537.67 (1052.11 to 2201.08) | 1535.81 (1051.13 to 2198.48) | 1533.65 (1050 to 2195.42) |
| 3875.45 (2642.79 to 5538.06) | 3911.69 (2661.92 to 5567.39) | 3932.31 (2678.37 to 5628.46) | 3930.37 (2694.02 to 5558.08) | 3907.27 (2700.21 to 5495.04) | 3872.51 (2698.85 to 5424) | 3835.34 (2674.78 to 5350.75) | 3805.58 (2654.75 to 5306.21) |
| 1703.6 (1173.95 to 2489.15) | 1705.42 (1175.22 to 2491.88) | 1707.86 (1176.91 to 2495.5) | 1711.09 (1179.14 to 2500.3) | 1715.02 (1181.85 to 2506.14) | 1719.2 (1184.73 to 2512.39) | 1723.07 (1187.41 to 2518.24) | 1726.09 (1189.52 to 2522.93) |
| 777.63 (517.7 to 1170.61) | 778.35 (518.2 to 1171.59) | 778.96 (518.63 to 1172.41) | 780.82 (520.87 to 1168.29) | 784.49 (521.96 to 1166.46) | 788.69 (524.78 to 1171.22) | 792.14 (528.55 to 1173.01) | 793.55 (527.36 to 1170.32) |
| 761.1 (507.99 to 1122.59) | 761.03 (507.94 to 1122.41) | 760.93 (507.85 to 1122.16) | 761.06 (507.91 to 1122.31) | 761.44 (508.12 to 1122.88) | 761.99 (508.45 to 1123.7) | 762.77 (508.95 to 1124.88) | 763.86 (509.65 to 1126.5) |
| 748.47 (501.78 to 1102.66) | 749.03 (502.18 to 1103.47) | 749.49 (502.52 to 1104.12) | 749.83 (502.8 to 1104.58) | 749.98 (502.94 to 1104.76) | 749.95 (502.96 to 1104.67) | 749.79 (502.87 to 1104.38) | 749.55 (502.72 to 1104.02) |
| 825.61 (550.99 to 1219.79) | 828.68 (553.1 to 1223.78) | 831.31 (554.93 to 1227.12) | 833.21 (556.29 to 1229.41) | 834.03 (556.92 to 1230.2) | 833.73 (556.81 to 1229.44) | 832.37 (555.97 to 1227.24) | 830.06 (554.5 to 1223.78) |

| 2006 | 2007 | 2008 | 2009 | 2010 | 2011 | 2012 | 2013 |
| --- | --- | --- | --- | --- | --- | --- | --- |
| 1870.75 (1284.76 to 2649.62) | 1852.64 (1272.88 to 2635.79) | 1834.36 (1260.55 to 2609.72) | 1815.63 (1247.86 to 2591.25) | 1796.95 (1235.26 to 2571.13) | 1780.44 (1224.62 to 2538.89) | 1767.04 (1216.2 to 2526.49) | 1756.34 (1209.41 to 2521.86) |
| 2327.34 (1605.42 to 3321.22) | 2330 (1606.9 to 3324.77) | 2329.82 (1606.4 to 3324.22) | 2327.08 (1604.22 to 3319.89) | 2322.45 (1600.84 to 3313.02) | 2321.65 (1595.1 to 3314.92) | 2327.03 (1592.48 to 3327.59) | 2334.13 (1591.77 to 3343.82) |
| 3586.59 (2397.21 to 5086.35) | 3575.02 (2380.65 to 5086.47) | 3565.57 (2377.48 to 5113.32) | 3561.1 (2385.32 to 5167.77) | 3561.16 (2385.43 to 5200.01) | 3559.08 (2397.43 to 5214.88) | 3550.56 (2369.73 to 5187.58) | 3538.24 (2361.3 to 5213.86) |
| 2418.52 (1721.82 to 3361.68) | 2423.61 (1722.96 to 3372.66) | 2431 (1725.39 to 3387.71) | 2440.02 (1729.32 to 3404.19) | 2449.36 (1732.88 to 3423.12) | 2461.86 (1743.38 to 3446.28) | 2481.77 (1758.67 to 3480.95) | 2504.53 (1771.74 to 3519.1) |
| 5349.38 (4027.06 to 7062.76) | 5326.46 (3995.39 to 7047.97) | 5300.35 (3950.2 to 7063.35) | 5277.14 (3907.9 to 7069.3) | 5257.68 (3863.47 to 7048.22) | 5248.26 (3857.18 to 7036.57) | 5248.13 (3857.95 to 7037.81) | 5253.78 (3862.78 to 7047.08) |
| 3412.54 (2304.54 to 4934.82) | 3415.97 (2306.9 to 4939) | 3415.26 (2306.49 to 4937.22) | 3411.73 (2304.16 to 4931.53) | 3406.67 (2300.8 to 4923.83) | 3400.98 (2297.03 to 4915.39) | 3394.95 (2293.05 to 4906.57) | 3388.77 (2289 to 4897.65) |
| 3241.86 (2219.9 to 4717.94) | 3243.67 (2216.73 to 4737.61) | 3243.06 (2219.37 to 4763.16) | 3241.29 (2209.12 to 4775.38) | 3239.55 (2216.85 to 4784.1) | 3250.73 (2218.46 to 4759.25) | 3278.82 (2226.51 to 4756.2) | 3311.94 (2250.83 to 4779.13) |
| 2319.12 (1598.35 to 3315.6) | 2322.86 (1600.79 to 3320.55) | 2325.09 (1602.1 to 3323.45) | 2325.95 (1602.41 to 3324.95) | 2325.31 (1601.61 to 3324.79) | 2321.9 (1603.22 to 3331.04) | 2315.96 (1602.68 to 3336.01) | 2309.84 (1601.2 to 3343.23) |
| 2220.5 (1502.85 to 3216.42) | 2219.58 (1502.79 to 3215.65) | 2216.45 (1501.06 to 3211.99) | 2211.86 (1498.17 to 3206.29) | 2205.28 (1493.79 to 3197.61) | 2196.96 (1488.17 to 3186.51) | 2189.14 (1482.86 to 3175.98) | 2182.63 (1478.36 to 3167.26) |
| 4814.06 (3345.41 to 6791.22) | 4820.03 (3349.12 to 6799.44) | 4820.81 (3349.33 to 6801.56) | 4816.31 (3345.93 to 6797.04) | 4815.9 (3345.48 to 6797.97) | 4814.6 (3342.3 to 6807.07) | 4806.26 (3332.02 to 6809.91) | 4799.51 (3321.87 to 6816.35) |
| 1721.77 (1181.75 to 2471.03) | 1706.76 (1171.23 to 2449.76) | 1689.78 (1159.12 to 2425.92) | 1673.29 (1147.17 to 2403.04) | 1660.17 (1137.32 to 2385.29) | 1651.72 (1130.6 to 2374.29) | 1647.94 (1127.03 to 2370.1) | 1648.29 (1126.25 to 2371.83) |
| 1754.37 (1178.35 to 2557.99) | 1724.82 (1158.33 to 2514.2) | 1694.1 (1137.46 to 2468.24) | 1663.39 (1116.18 to 2422.11) | 1634.82 (1095.99 to 2380.56) | 1611.26 (1079.18 to 2345.12) | 1595.14 (1067.42 to 2320.59) | 1587.18 (1061.21 to 2308.04) |
| 1659.14 (1128.41 to 2406.23) | 1643.75 (1118.2 to 2383.15) | 1625.46 (1105.86 to 2356.61) | 1605.69 (1092.34 to 2328.53) | 1585.26 (1078.23 to 2300.05) | 1565.36 (1064.32 to 2273.49) | 1547.71 (1052.27 to 2251.12) | 1533.4 (1042.65 to 2233.99) |
| 1978.95 (1377.48 to 2848.61) | 1955.45 (1359.84 to 2806.35) | 1931.68 (1347.96 to 2769.47) | 1908.68 (1334.67 to 2733) | 1887.52 (1321.09 to 2699.84) | 1868.64 (1305.88 to 2677.14) | 1853.38 (1292.4 to 2661.89) | 1843.06 (1283.49 to 2654.29) |
| 872.67 (588.12 to 1283.7) | 873.74 (588.98 to 1285.34) | 874.86 (589.87 to 1287.04) | 876.02 (590.58 to 1288.78) | 877.25 (591.36 to 1290.61) | 881.81 (594.7 to 1294.9) | 891.04 (598.07 to 1304.19) | 902.1 (602.57 to 1316.13) |
| 1530.39 (1048.37 to 2192.08) | 1525.67 (1046.68 to 2188.09) | 1521.64 (1045.4 to 2185.73) | 1518.79 (1042.88 to 2185.17) | 1513.05 (1036.96 to 2182.36) | 1504.64 (1033.02 to 2172.84) | 1498.11 (1028.56 to 2163.05) | 1493.8 (1024.89 to 2155.9) |
| 3793.11 (2653.33 to 5258.54) | 3791.73 (2650.4 to 5229.73) | 3790.67 (2644.85 to 5268.05) | 3783.94 (2633.75 to 5251.31) | 3774.17 (2622.26 to 5221.03) | 3757.69 (2619.82 to 5221.03) | 3724.17 (2598.76 to 5196.82) | 3678.02 (2572.13 to 5136.4) |
| 1727.63 (1190.65 to 2525.53) | 1727.35 (1190.57 to 2525.53) | 1725.37 (1189.34 to 2523.08) | 1722.01 (1187.2 to 2518.61) | 1717.59 (1184.33 to 2512.52) | 1712.41 (1180.95 to 2505.21) | 1706.93 (1177.35 to 2497.31) | 1701.67 (1173.85 to 2489.56) |
| 793.5 (527.35 to 1170.3) | 793.44 (527.35 to 1170.32) | 793.53 (527.45 to 1170.58) | 793.91 (527.75 to 1171.26) | 794.7 (528.33 to 1172.55) | 795.94 (529.2 to 1174.48) | 797.66 (530.4 to 1177.1) | 799.88 (531.93 to 1180.42) |
| 765.35 (510.61 to 1128.7) | 767.23 (511.85 to 1131.34) | 769.41 (513.3 to 1134.27) | 771.84 (514.92 to 1137.51) | 774.45 (516.68 to 1141) | 777.37 (518.66 to 1144.95) | 780.39 (520.74 to 1149.05) | 783.28 (522.72 to 1152.85) |
| 749.37 (502.6 to 1103.75) | 749.33 (502.55 to 1103.7) | 749.52 (502.66 to 1103.98) | 750.04 (502.98 to 1104.76) | 751.02 (503.62 to 1106.23) | 752.55 (504.63 to 1108.49) | 754.66 (506.02 to 1111.6) | 757.39 (507.82 to 1115.63) |
| 827.06 (552.56 to 1219.43) | 823.66 (550.35 to 1214.61) | 820.14 (548.03 to 1209.73) | 816.67 (545.74 to 1205.01) | 813.41 (543.54 to 1200.67) | 810.63 (541.65 to 1197.11) | 808.75 (540.34 to 1194.91) | 808 (539.76 to 1194.34) |

| 2014 | 2015 | 2016 | 2017 | 2018 | 2019 | 2020 | 2021 | **Percentage change from 1990 to 2021 (%)** |
| --- | --- | --- | --- | --- | --- | --- | --- | --- |
| 1748.45 (1204.61 to 2517.05) | 1742.25 (1200.94 to 2510.16) | 1737.79 (1198.26 to 2509.22) | 1737.71 (1198.4 to 2507.43) | 1742.55 (1201.86 to 2510.05) | 1750.5 (1205.5 to 2517.18) | 1758.06 (1212.01 to 2521.94) | 1778.97 (1219.23 to 2552.17) | -6 (-8.48 to -3.45) |
| 2338.46 (1591.66 to 3358.13) | 2336.47 (1588.5 to 3371.39) | 2321.31 (1590.34 to 3343.92) | 2296.53 (1587.21 to 3318.04) | 2276.35 (1581.02 to 3295.59) | 2275.92 (1577.33 to 3293.59) | 2291.44 (1587.44 to 3317.22) | 2337.54 (1606.59 to 3375.88) | -1.76 (-5.37 to 2.91) |
| 3526.03 (2340.6 to 5192.25) | 3520.54 (2322.43 to 5155.13) | 3539.41 (2334.22 to 5134.37) | 3580.72 (2374.18 to 5235.02) | 3622.64 (2400.81 to 5336.33) | 3644.03 (2414.21 to 5399.14) | 3624.34 (2403.77 to 5177.29) | 3627.13 (2405.43 to 5180.56) | 12.88 (1.98 to 22.22) |
| 2525.56 (1784.33 to 3543.37) | 2537.92 (1790.65 to 3559.15) | 2538.9 (1779.44 to 3550.61) | 2533.74 (1757.62 to 3541.42) | 2528.96 (1726.89 to 3554.04) | 2531.93 (1704.73 to 3575.18) | 2540.43 (1710.18 to 3587.52) | 2530.76 (1701.16 to 3584.32) | 4.66 (-2.25 to 10.72) |
| 5260.29 (3867.77 to 7057.57) | 5264.37 (3870.55 to 7064.79) | 5269.72 (3873.87 to 7073.25) | 5283.43 (3882.91 to 7092.79) | 5302.05 (3895.66 to 7118.46) | 5324 (3911.04 to 7148.24) | 5349.85 (3929.35 to 7183.12) | 5366.09 (3936.79 to 7148.66) | 4.48 (-8.3 to 22.13) |
| 3383.57 (2285.64 to 4890.43) | 3380.07 (2283.4 to 4885.71) | 3378.87 (2282.66 to 4884.37) | 3379.9 (2283.39 to 4886.35) | 3382.33 (2285.03 to 4890.44) | 3385.34 (2287.04 to 4895.36) | 3390.65 (2290.56 to 4903.63) | 3399.46 (2296.43 to 4916.89) | 3.54 (3.13 to 3.89) |
| 3338.88 (2270.88 to 4772.53) | 3348.97 (2244.23 to 4763.02) | 3349.66 (2278.34 to 4811.36) | 3353.04 (2269.58 to 4868.57) | 3356.24 (2264.11 to 4890.56) | 3356.23 (2255.26 to 4814.35) | 3264.51 (2233.56 to 4704.6) | 3344.82 (2283.1 to 4837.69) | -0.77 (-9.56 to 9.36) |
| 2305.51 (1600.1 to 3345.05) | 2304.57 (1597.31 to 3348.97) | 2312.41 (1599.78 to 3353.58) | 2328.55 (1607.68 to 3369.26) | 2342.78 (1608.72 to 3379.37) | 2348.2 (1602.1 to 3376.81) | 2352.46 (1604.97 to 3383.81) | 2362.7 (1611.99 to 3398.52) | 6.02 (3.16 to 8.9) |
| 2177.31 (1474.58 to 3160.19) | 2174.46 (1472.35 to 3156.59) | 2175.84 (1472.84 to 3158.96) | 2182.23 (1476.68 to 3168.35) | 2194.86 (1484.82 to 3186.39) | 2215.45 (1498.42 to 3215.63) | 2241.9 (1516 to 3253.62) | 2268.04 (1533.46 to 3291.64) | 5.49 (-5.28 to 15.43) |
| 4794.35 (3313.25 to 6824.08) | 4788.22 (3304.01 to 6826.62) | 4781.87 (3299.74 to 6817.84) | 4777.78 (3296.96 to 6813.06) | 4776.05 (3295.73 to 6812.15) | 4777.58 (3296.66 to 6816.19) | 4782.3 (3299.7 to 6825.31) | 4789.6 (3304.46 to 6838.41) | 3.46 (0.96 to 5.29) |
| 1650.72 (1126.97 to 2376.5) | 1653.22 (1128.22 to 2381.05) | 1654.59 (1128.77 to 2383.73) | 1655.55 (1129.03 to 2385.59) | 1657.79 (1130.2 to 2389.08) | 1662.06 (1132.83 to 2395.32) | 1667.76 (1136.74 to 2403.44) | 1674.64 (1141.76 to 2413.1) | -0.08 (-0.19 to 0.03) |
| 1585.77 (1059.47 to 2305.12) | 1589.93 (1061.58 to 2310.43) | 1601.17 (1068.58 to 2326.19) | 1620.58 (1081.21 to 2354.07) | 1646.61 (1098.41 to 2391.76) | 1676.53 (1118.29 to 2435.27) | 1707.35 (1138.83 to 2480.2) | 1736.52 (1158.37 to 2522.91) | 5.14 (4.83 to 5.52) |
| 1522.45 (1035.27 to 2221.92) | 1514.88 (1030.03 to 2214.79) | 1511.5 (1027.34 to 2213.34) | 1512.14 (1027.41 to 2214.85) | 1516.16 (1029.81 to 2221.21) | 1522.59 (1033.93 to 2231) | 1530.78 (1039.32 to 2243.27) | 1540.81 (1046.07 to 2258.11) | 2.44 (2.21 to 2.72) |
| 1837.78 (1280.06 to 2651.81) | 1836.7 (1279.11 to 2652.01) | 1833.52 (1272.73 to 2652.85) | 1826.67 (1265.56 to 2648.77) | 1822.64 (1260.81 to 2653.41) | 1827.95 (1259.6 to 2671.45) | 1840.83 (1269.67 to 2665.33) | 1858.04 (1281.67 to 2689.31) | -4.9 (-8.84 to -0.72) |
| 912.08 (608.7 to 1323.49) | 918.01 (608.97 to 1327.2) | 920.99 (610.99 to 1331.38) | 924.08 (613.09 to 1335.72) | 927.28 (615.28 to 1340.21) | 930.63 (617.57 to 1344.87) | 934.05 (619.91 to 1349.59) | 937.35 (622.17 to 1354.13) | 7.49 (-0.76 to 15.65) |
| 1490.9 (1022.22 to 2150.84) | 1489.61 (1019.46 to 2148.5) | 1489.35 (1019.13 to 2148.37) | 1489.65 (1019.2 to 2149) | 1490.49 (1019.66 to 2150.3) | 1491.77 (1020.42 to 2152.11) | 1493.35 (1021.41 to 2154.23) | 1495.05 (1022.49 to 2156.38) | -3.33 (-8.43 to -0.64) |
| 3634.61 (2540.85 to 5137.45) | 3593.95 (2516.44 to 5159.12) | 3553.26 (2495.36 to 5046.53) | 3532.42 (2469.35 to 5002.18) | 3545.7 (2488.3 to 5002.63) | 3583.86 (2509.45 to 5094.53) | 3645.46 (2553.54 to 5181.29) | 3773.94 (2643.15 to 5401.78) | 21.3 (11.54 to 31.51) |
| 1696.95 (1170.67 to 2482.45) | 1693.05 (1167.96 to 2476.4) | 1690.21 (1165.89 to 2471.79) | 1688.58 (1164.55 to 2468.84) | 1688.04 (1163.9 to 2467.48) | 1688.47 (1163.9 to 2467.57) | 1689.71 (1164.5 to 2468.95) | 1691.67 (1165.65 to 2471.49) | -0.42 (-0.49 to -0.35) |
| 802.51 (533.72 to 1184.31) | 805.43 (535.71 to 1188.59) | 808.56 (537.83 to 1193.13) | 811.8 (540 to 1197.78) | 815.07 (542.18 to 1202.43) | 818.33 (544.34 to 1207.04) | 821.59 (546.49 to 1211.63) | 824.75 (548.55 to 1216.05) | 7.72 (1.77 to 13.49) |
| 786.13 (524.7 to 1156.58) | 789.02 (526.72 to 1160.39) | 791.94 (528.76 to 1164.24) | 794.9 (530.83 to 1168.18) | 797.76 (532.86 to 1171.93) | 800.39 (534.76 to 1175.27) | 802.87 (536.56 to 1178.54) | 805.18 (538.23 to 1181.58) | 6.51 (5.89 to 7.05) |
| 760.76 (510.06 to 1120.63) | 764.71 (512.67 to 1126.47) | 769.07 (515.57 to 1132.92) | 773.74 (518.69 to 1139.81) | 778.68 (522.01 to 1147.09) | 783.83 (525.49 to 1154.66) | 789.06 (529.06 to 1162.31) | 794.17 (532.56 to 1169.76) | 7.21 (6.99 to 7.37) |
| 808.46 (539.98 to 1195.54) | 810.12 (540.99 to 1198.4) | 812.74 (542.65 to 1202.1) | 816.02 (544.78 to 1206.81) | 819.72 (547.21 to 1212.2) | 823.75 (549.87 to 1218.09) | 828.03 (552.74 to 1224.42) | 832.43 (555.71 to 1230.95) | 4.31 (3.96 to 4.7) |

Abbreviations: DALYs, disability-adjusted life-years; UI, uncertainty interval.

**eTable** **5b. Incidence of ADHD by region and year, <20 years, 1990-2021.**

| **Regions** | 1990 | 1991 | 1992 | 1993 | 1994 | 1995 | 1996 | 1997 |
| --- | --- | --- | --- | --- | --- | --- | --- | --- |
| Global | 165.62 (112.6 to 242.24) | 168.56 (114.56 to 246.22) | 171.29 (116.59 to 250.34) | 173.09 (117.86 to 253.12) | 173.53 (118 to 253.96) | 172.49 (117.16 to 252.49) | 170.42 (116.07 to 249.48) | 167.89 (114.67 to 245.93) |
| High-income Asia Pacific | 175.25 (115.98 to 260.51) | 174.19 (116.35 to 258.89) | 173.51 (116.75 to 257.21) | 173.44 (116.98 to 255.5) | 174.03 (117.87 to 254.33) | 175.08 (119.59 to 254.61) | 176.51 (120.56 to 256.05) | 178.19 (121.77 to 258.78) |
| High-income North America | 289.47 (191.24 to 427.8) | 296.3 (195.83 to 438.05) | 302.47 (200.63 to 447.11) | 307.15 (203.69 to 451.51) | 310.31 (207.24 to 455.69) | 311.56 (209.5 to 457.34) | 310.95 (207.51 to 454.59) | 309.51 (206.24 to 451.15) |
| Western Europe | 203.15 (137.51 to 298.21) | 202.72 (137 to 297.23) | 202.65 (136.86 to 296.8) | 202.86 (137.06 to 296.79) | 203.25 (137.38 to 297.03) | 203.53 (137.63 to 297.1) | 203.96 (138.87 to 297.92) | 204.72 (140.47 to 298.72) |
| Australasia | 425.27 (292.39 to 609.42) | 429.95 (295.86 to 616.59) | 434.53 (299.18 to 623.95) | 438.06 (301.75 to 629.02) | 441.2 (304.04 to 633.45) | 443.54 (305.78 to 636.64) | 445 (307.08 to 638.27) | 446.65 (308.56 to 640.09) |
| Andean Latin America | 296.22 (200.75 to 438.56) | 295.28 (200.11 to 437.16) | 294.58 (199.63 to 436.14) | 294.13 (199.32 to 435.5) | 293.96 (199.21 to 435.27) | 294.1 (199.3 to 435.48) | 294.45 (199.54 to 435.99) | 294.85 (199.81 to 436.57) |
| Tropical Latin America | 287.31 (194.96 to 421.05) | 275.81 (188.12 to 405.73) | 264.96 (181.94 to 390.71) | 255.47 (175.86 to 375.92) | 248.11 (169.76 to 366.45) | 243.58 (164.77 to 359.29) | 241.54 (164.46 to 353.51) | 241.05 (164.7 to 353.6) |
| Central Latin America | 200.94 (134.85 to 292.78) | 200.17 (135.22 to 290.28) | 199.58 (135.37 to 288.15) | 199.32 (135.17 to 288.45) | 199.47 (134.91 to 287.68) | 199.99 (135.3 to 287.58) | 200.68 (135.76 to 288.54) | 201.34 (136.21 to 289.46) |
| Southern Latin America | 199.02 (133.48 to 293.8) | 198.51 (133.13 to 293.06) | 198.57 (133.17 to 293.15) | 199.06 (133.5 to 293.87) | 199.67 (133.92 to 294.77) | 200.13 (134.23 to 295.43) | 200.04 (134.17 to 295.28) | 199.33 (133.71 to 294.23) |
| Caribbean | 395.63 (271.48 to 584.15) | 400.45 (274.69 to 591.38) | 404.85 (277.63 to 597.96) | 408.22 (279.9 to 602.97) | 410.34 (281.34 to 606.09) | 411.25 (281.95 to 607.38) | 410.79 (281.6 to 606.63) | 408.84 (280.19 to 604.13) |
| Central Europe | 133.78 (89.76 to 198.02) | 132.64 (89 to 196.32) | 131.53 (88.26 to 194.67) | 130.44 (87.54 to 193.04) | 129.43 (86.88 to 191.53) | 128.43 (86.22 to 190.03) | 127.33 (85.48 to 188.39) | 125.99 (84.59 to 186.4) |
| Eastern Europe | 143.72 (94.83 to 215.41) | 144.33 (95.24 to 216.29) | 144.14 (95.12 to 215.97) | 142.58 (94.1 to 213.57) | 139.77 (92.26 to 209.3) | 136.03 (89.8 to 203.63) | 131.43 (86.78 to 196.69) | 126.18 (83.32 to 188.79) |
| Central Asia | 145.08 (97.11 to 216.13) | 146.24 (97.89 to 217.87) | 147.26 (98.57 to 219.38) | 148.02 (99.09 to 220.51) | 148.25 (99.25 to 220.84) | 147.77 (98.93 to 220.12) | 146.68 (98.21 to 218.5) | 145.03 (97.11 to 216.04) |
| North Africa and Middle East | 187.3 (128.76 to 279.05) | 185.71 (127.84 to 276.38) | 183.56 (126.5 to 272.86) | 180.81 (124.72 to 268.48) | 177.83 (122.73 to 263.79) | 174.9 (120.74 to 259.3) | 173 (119.27 to 255.65) | 172.3 (118.45 to 253.79) |
| South Asia | 84.58 (55.88 to 127.68) | 83.35 (54.91 to 125.13) | 82.02 (53.95 to 122.58) | 80.65 (52.92 to 120.41) | 79.37 (51.88 to 117.65) | 78.3 (51.51 to 116.39) | 77.4 (51.37 to 115.03) | 76.57 (50.71 to 113.94) |
| Southeast Asia | 137.43 (95.55 to 201.9) | 136.46 (94.81 to 200.51) | 135.58 (94.18 to 199.23) | 134.82 (93.63 to 198.13) | 134.21 (93.19 to 197.24) | 133.75 (92.86 to 196.59) | 133.43 (92.62 to 196.18) | 133.19 (92.44 to 195.92) |
| East Asia | 252.61 (173.91 to 377.49) | 273.7 (187.75 to 404.92) | 294.94 (201.5 to 432.04) | 312.68 (212.99 to 457.71) | 323.72 (219.91 to 477.78) | 326.75 (221.38 to 483.68) | 323.59 (218.95 to 478.93) | 317.11 (213.97 to 468.58) |
| Oceania | 166.21 (112.98 to 248.8) | 165.9 (112.77 to 248.34) | 165.63 (112.58 to 247.93) | 165.42 (112.44 to 247.62) | 165.27 (112.34 to 247.39) | 165.15 (112.26 to 247.22) | 165.09 (112.22 to 247.13) | 165.08 (112.21 to 247.11) |
| Western Sub-Saharan Africa | 79.7 (52.32 to 120.67) | 79.62 (52.26 to 120.54) | 79.52 (52.19 to 120.37) | 79.4 (52.12 to 120.18) | 79.31 (52.05 to 120.02) | 79.23 (51.99 to 119.89) | 79.17 (51.95 to 119.75) | 79.12 (51.91 to 119.64) |
| Eastern Sub-Saharan Africa | 77.19 (50.57 to 114.87) | 76.8 (50.31 to 114.29) | 76.44 (50.07 to 113.76) | 76.16 (49.88 to 113.35) | 75.99 (49.77 to 113.1) | 75.82 (49.65 to 112.85) | 75.66 (49.54 to 112.61) | 75.6 (49.51 to 112.52) |
| Central Sub-Saharan Africa | 77.53 (51.13 to 114.64) | 77.6 (51.18 to 114.73) | 77.64 (51.21 to 114.8) | 77.66 (51.22 to 114.82) | 77.63 (51.2 to 114.78) | 77.56 (51.15 to 114.67) | 77.46 (51.09 to 114.53) | 77.37 (51.03 to 114.4) |
| Southern Sub-Saharan Africa | 73.53 (47.79 to 109.7) | 73.33 (47.66 to 109.4) | 73.08 (47.49 to 109.02) | 72.77 (47.3 to 108.56) | 72.42 (47.06 to 108.02) | 72.02 (46.8 to 107.42) | 71.6 (46.53 to 106.79) | 71.18 (46.25 to 106.15) |

| 1998 | 1999 | 2000 | 2001 | 2002 | 2003 | 2004 | 2005 |
| --- | --- | --- | --- | --- | --- | --- | --- |
| 164.99 (112.86 to 241.58) | 161.81 (110.55 to 236.59) | 158.55 (108.09 to 231.6) | 155.7 (106.58 to 226.94) | 153.44 (105.08 to 223.03) | 151.68 (103.8 to 220.16) | 150.35 (102.62 to 218.04) | 149.38 (101.63 to 216.57) |
| 179.9 (123.08 to 261.71) | 181.92 (124.59 to 265.26) | 184.03 (126.14 to 269.25) | 185.55 (127.19 to 270.57) | 186.35 (127.67 to 270.65) | 186.72 (127.78 to 270.6) | 186.29 (127.34 to 270.99) | 185.37 (126.8 to 269.09) |
| 307.06 (205.14 to 446.68) | 303.77 (203.32 to 442.21) | 300.38 (202.55 to 438.27) | 297.85 (197.72 to 433.42) | 296.37 (195.78 to 431.16) | 295.58 (194.47 to 430.86) | 294.91 (193.16 to 431.4) | 294.34 (193.5 to 431.27) |
| 205.52 (141.45 to 299.41) | 206.26 (141.91 to 300.2) | 206.51 (142.74 to 300.77) | 206.95 (143.27 to 300.89) | 208.14 (144.28 to 302.35) | 209.54 (145.28 to 303.44) | 211.04 (146.17 to 304.38) | 212.47 (147.07 to 305.03) |
| 447.4 (309.46 to 640.65) | 446.85 (309.48 to 639.44) | 444.81 (308.46 to 637.24) | 443.41 (310.51 to 640.41) | 443.23 (316.59 to 628.63) | 443.31 (322.62 to 616.37) | 442.77 (326.17 to 606.1) | 441.07 (322.74 to 598.15) |
| 295.04 (199.95 to 436.83) | 294.84 (199.82 to 436.52) | 294.25 (199.43 to 435.64) | 293.41 (198.86 to 434.4) | 292.26 (198.09 to 432.71) | 290.77 (197.08 to 430.51) | 289.09 (195.95 to 428.04) | 287.32 (194.75 to 425.43) |
| 241.82 (165.29 to 358.33) | 243.45 (166.37 to 362.05) | 245.42 (167.05 to 364.77) | 247.57 (168.8 to 367.14) | 249.88 (170.74 to 368.05) | 251.79 (172.07 to 371.01) | 252.92 (172.92 to 373.83) | 252.95 (173.2 to 374.37) |
| 201.88 (136.57 to 290.22) | 202.11 (136.73 to 290.55) | 201.93 (136.59 to 290.28) | 201.26 (136.58 to 290.4) | 200.14 (136.44 to 289.98) | 198.74 (136.14 to 289.35) | 197.22 (135.68 to 288.44) | 195.81 (135.08 to 287.01) |
| 198.32 (133.03 to 292.73) | 197.26 (132.32 to 291.16) | 196.09 (131.54 to 289.44) | 194.76 (130.63 to 284.24) | 193.25 (128.86 to 281.47) | 191.5 (126.9 to 278.95) | 189.74 (125.06 to 276.64) | 188.43 (124.59 to 272.95) |
| 405.69 (277.92 to 600.03) | 402.01 (275.25 to 595.11) | 398.53 (272.71 to 590.42) | 395.73 (270.64 to 586.69) | 393.69 (269.16 to 584.21) | 392.36 (268.19 to 582.78) | 391.53 (267.56 to 582.01) | 390.84 (266.99 to 581.31) |
| 124.48 (83.58 to 184.17) | 122.96 (82.56 to 181.92) | 121.45 (81.55 to 179.67) | 120.22 (80.73 to 177.85) | 119.28 (80.11 to 176.46) | 118.67 (79.7 to 175.53) | 118.43 (79.54 to 175.12) | 118.66 (79.71 to 175.41) |
| 120.79 (79.76 to 180.69) | 115.76 (76.44 to 173.15) | 111.43 (73.57 to 166.68) | 108.03 (71.32 to 161.61) | 105.95 (69.94 to 158.54) | 105.42 (69.58 to 157.81) | 106.12 (70.03 to 158.94) | 107.96 (71.23 to 161.77) |
| 142.83 (95.64 to 212.77) | 140.26 (93.92 to 208.94) | 137.32 (91.96 to 204.56) | 134.14 (89.83 to 199.83) | 131.05 (87.76 to 195.23) | 128.26 (85.89 to 191.08) | 125.9 (84.31 to 187.57) | 124.05 (83.07 to 184.84) |
| 172.11 (118.19 to 252.75) | 171.82 (117.83 to 251.64) | 170.76 (116.42 to 249.6) | 168.95 (114.97 to 247.22) | 166.97 (113.44 to 244.03) | 164.97 (111.95 to 239.77) | 163.4 (110.77 to 236.65) | 162.51 (110.03 to 234.67) |
| 75.88 (50.32 to 112.8) | 75.35 (49.89 to 112.33) | 74.98 (49.6 to 111.66) | 74.88 (49.66 to 110.66) | 75.05 (50.03 to 110.45) | 75.29 (50.08 to 111) | 75.45 (50.24 to 111.49) | 75.38 (50.26 to 111.94) |
| 132.99 (92.3 to 195.71) | 132.75 (92.14 to 195.28) | 132.4 (91.9 to 194.66) | 131.93 (91.55 to 193.95) | 131.37 (91.13 to 193.12) | 130.76 (90.69 to 192.23) | 130.14 (90.23 to 191.32) | 129.49 (89.76 to 190.38) |
| 307.92 (207.34 to 453.72) | 296.68 (200.02 to 434.12) | 284.79 (192.42 to 417.37) | 274.39 (186.62 to 397.41) | 266.27 (182.17 to 383.8) | 260.87 (178.36 to 372.61) | 258.29 (175.37 to 367.47) | 258.46 (174.6 to 369.22) |
| 165.13 (112.24 to 247.18) | 165.18 (112.27 to 247.24) | 165.15 (112.26 to 247.19) | 165.02 (112.17 to 246.99) | 164.76 (111.99 to 246.58) | 164.29 (111.67 to 245.88) | 163.59 (111.2 to 244.82) | 162.7 (110.59 to 243.48) |
| 79.1 (51.89 to 119.58) | 79.12 (51.9 to 119.57) | 79.18 (51.93 to 119.62) | 79.4 (52.12 to 119.58) | 79.82 (52.38 to 120.05) | 80.32 (52.51 to 120.42) | 80.78 (52.69 to 120.71) | 81.06 (52.79 to 121.29) |
| 75.64 (49.54 to 112.58) | 75.76 (49.62 to 112.75) | 75.9 (49.72 to 112.96) | 76.07 (49.83 to 113.2) | 76.23 (49.94 to 113.44) | 76.39 (50.04 to 113.67) | 76.53 (50.14 to 113.88) | 76.67 (50.24 to 114.08) |
| 77.29 (50.97 to 114.26) | 77.19 (50.91 to 114.13) | 77.1 (50.85 to 113.99) | 77.03 (50.8 to 113.88) | 76.98 (50.77 to 113.82) | 76.98 (50.77 to 113.81) | 77.01 (50.79 to 113.86) | 77.08 (50.84 to 113.96) |
| 70.73 (45.96 to 105.48) | 70.26 (45.65 to 104.77) | 69.76 (45.33 to 104.04) | 69.26 (45.01 to 103.3) | 68.79 (44.72 to 102.61) | 68.41 (44.47 to 102.05) | 68.15 (44.3 to 101.67) | 68.05 (44.23 to 101.53) |

| 2006 | 2007 | 2008 | 2009 | 2010 | 2011 | 2012 | 2013 |
| --- | --- | --- | --- | --- | --- | --- | --- |
| 148.85 (100.99 to 216.02) | 148.84 (100.76 to 216.38) | 149.29 (100.91 to 217.49) | 150.09 (101.35 to 219.11) | 151.01 (101.87 to 221.08) | 151.84 (102.33 to 222.2) | 152.39 (102.74 to 222.91) | 152.69 (103.05 to 223.23) |
| 184.04 (125.84 to 267.44) | 182.05 (124.36 to 264.83) | 179.89 (122.77 to 261.95) | 178.02 (121.46 to 259.46) | 175.97 (120.03 to 256.7) | 175.06 (119.22 to 255.26) | 175.93 (119.88 to 255.84) | 177.75 (121.11 to 257.94) |
| 293.72 (192.67 to 433.04) | 293.32 (192.28 to 434.93) | 293.65 (192.56 to 441.56) | 294.69 (193.37 to 447.14) | 296.07 (195.34 to 452.28) | 296.81 (194.74 to 452.25) | 296.43 (194.64 to 450.38) | 295.13 (194.94 to 448.25) |
| 214 (148.14 to 305.47) | 215.9 (149.28 to 307.02) | 218.18 (150.33 to 310.03) | 220.85 (151.72 to 313.52) | 223.56 (153.35 to 317.57) | 226.47 (155.53 to 321.93) | 229.85 (157.84 to 327.06) | 233.04 (159.84 to 331.89) |
| 439.08 (320.6 to 595.67) | 437.45 (318.28 to 594.66) | 437.57 (315.2 to 597.96) | 439.92 (312.49 to 605.23) | 443.64 (310.66 to 616.88) | 447.91 (313.63 to 622.82) | 452.75 (317.02 to 629.47) | 457.53 (320.39 to 636) |
| 285.54 (193.54 to 422.82) | 283.78 (192.35 to 420.25) | 282.12 (191.22 to 417.86) | 280.8 (190.32 to 415.95) | 280.04 (189.79 to 414.85) | 279.94 (189.71 to 414.73) | 280.59 (190.15 to 415.71) | 281.94 (191.06 to 417.73) |
| 251.86 (171.43 to 371.9) | 250.03 (170.18 to 368.02) | 247.88 (168.58 to 363.98) | 245.8 (166.61 to 362.88) | 244.32 (165.31 to 358.33) | 244.86 (165.88 to 361.9) | 247.72 (168.07 to 365.94) | 251.91 (168.87 to 373.38) |
| 194.5 (134.17 to 285.11) | 193.25 (133.3 to 283.29) | 192.19 (132.56 to 281.74) | 191.36 (131.98 to 280.52) | 190.72 (131.53 to 279.56) | 190.19 (131.05 to 278.72) | 189.85 (130.18 to 277.67) | 189.69 (129.48 to 277.54) |
| 187.48 (123.97 to 271.66) | 186.35 (123.23 to 270.09) | 185.09 (122.41 to 268.29) | 184.17 (121.8 to 266.97) | 183.95 (121.65 to 266.65) | 184.36 (121.91 to 267.25) | 185.11 (122.4 to 268.34) | 185.82 (122.86 to 269.34) |
| 390.03 (266.37 to 580.12) | 389.03 (265.63 to 578.63) | 387.93 (264.81 to 577.02) | 387 (264.1 to 575.69) | 386.26 (263.52 to 574.63) | 385.83 (262.71 to 574) | 385.85 (262.06 to 574.51) | 386.37 (261.71 to 575.89) |
| 119.21 (80.09 to 176.18) | 120.13 (80.7 to 177.51) | 121.49 (81.61 to 179.49) | 123.35 (82.87 to 182.27) | 125.81 (84.51 to 185.93) | 128.6 (86.38 to 190.11) | 131.44 (88.28 to 194.39) | 133.96 (89.97 to 198.18) |
| 110.91 (73.17 to 166.28) | 114.72 (75.68 to 172.06) | 119.13 (78.58 to 178.74) | 124.06 (81.82 to 186.2) | 129.21 (85.21 to 194) | 133.97 (88.34 to 201.23) | 137.92 (90.94 to 207.2) | 141.15 (93.07 to 212.09) |
| 122.98 (82.35 to 183.25) | 122.73 (82.18 to 182.88) | 123.3 (82.56 to 183.75) | 124.63 (83.44 to 185.73) | 126.67 (84.81 to 188.79) | 129.43 (86.65 to 192.91) | 132.57 (88.75 to 197.59) | 135.79 (90.91 to 202.41) |
| 162.06 (109.92 to 234.41) | 161.82 (109.91 to 235.54) | 161.71 (109.96 to 235.95) | 161.84 (110.16 to 236.65) | 162.3 (110.58 to 237.11) | 163.12 (111.03 to 238.43) | 164.22 (111.59 to 240.35) | 165.52 (112.23 to 242.65) |
| 75.14 (50.09 to 111.57) | 74.88 (49.91 to 111.18) | 74.63 (49.74 to 110.8) | 74.41 (49.58 to 110.45) | 74.23 (49.46 to 110.15) | 74.36 (49.68 to 110.57) | 74.87 (49.96 to 111.19) | 75.52 (50.38 to 111.61) |
| 128.77 (89.08 to 189.54) | 128.02 (88.24 to 188.96) | 127.47 (87.42 to 188.7) | 127.22 (86.86 to 188.71) | 127 (86.31 to 188.44) | 126.71 (85.84 to 187.72) | 126.53 (85.59 to 187.13) | 126.47 (85.29 to 186.98) |
| 262.03 (176.63 to 375.94) | 269.19 (180.8 to 389.27) | 279.41 (187.6 to 404.35) | 291.77 (195.81 to 422.45) | 304.88 (203.86 to 447.11) | 315.83 (211.65 to 459.59) | 321.82 (216.77 to 469.06) | 323.84 (219.48 to 474.35) |
| 161.68 (109.9 to 241.96) | 160.61 (109.17 to 240.37) | 159.63 (108.5 to 238.91) | 158.87 (107.98 to 237.78) | 158.44 (107.69 to 237.15) | 158.45 (107.69 to 237.17) | 158.92 (108.01 to 237.89) | 159.74 (108.57 to 239.12) |
| 81.22 (52.9 to 121.52) | 81.4 (53.02 to 121.79) | 81.59 (53.14 to 122.07) | 81.77 (53.26 to 122.35) | 81.95 (53.38 to 122.61) | 82.09 (53.47 to 122.82) | 82.18 (53.53 to 122.95) | 82.21 (53.55 to 122.99) |
| 76.77 (50.31 to 114.22) | 76.83 (50.35 to 114.3) | 76.87 (50.39 to 114.36) | 76.89 (50.4 to 114.37) | 76.87 (50.39 to 114.34) | 76.78 (50.34 to 114.22) | 76.62 (50.25 to 113.99) | 76.42 (50.12 to 113.7) |
| 77.2 (50.91 to 114.13) | 77.35 (51.02 to 114.37) | 77.55 (51.15 to 114.66) | 77.78 (51.3 to 115) | 78.03 (51.46 to 115.36) | 78.26 (51.62 to 115.71) | 78.48 (51.76 to 116.02) | 78.65 (51.87 to 116.27) |
| 68.13 (44.28 to 101.66) | 68.4 (44.45 to 102.07) | 68.83 (44.73 to 102.71) | 69.37 (45.08 to 103.52) | 70 (45.49 to 104.47) | 70.69 (45.94 to 105.5) | 71.4 (46.4 to 106.56) | 72.08 (46.84 to 107.57) |

| 2014 | 2015 | 2016 | 2017 | 2018 | 2019 | 2020 | 2021 | **Percentage change from 1990 to 2021 (%)** |
| --- | --- | --- | --- | --- | --- | --- | --- | --- |
| 153.13 (103.43 to 223.73) | 153.71 (103.87 to 224.59) | 154.16 (104.38 to 225.17) | 154.64 (104.45 to 226.08) | 155.27 (104.6 to 227.29) | 155.79 (104.79 to 228.99) | 155.9 (104.81 to 228.03) | 155.99 (105.29 to 225.92) | -5.81 (-8.21 to -3.04) |
| 180.06 (122.69 to 260.98) | 182.58 (124.41 to 264.76) | 183.52 (124.64 to 267.98) | 182.58 (123.79 to 268.72) | 181.29 (123.67 to 267.78) | 181.08 (124.06 to 268.57) | 181.56 (124.35 to 269.23) | 183.41 (123.7 to 271.77) | 4.65 (0.55 to 10.19) |
| 293.39 (194.59 to 445.22) | 291.92 (192.62 to 442.04) | 291.85 (193.7 to 439.75) | 293.06 (193.6 to 439.77) | 294.39 (195.74 to 439.35) | 294.62 (195.09 to 440.97) | 291.74 (190.54 to 436.47) | 291.3 (190.27 to 435.8) | 0.63 (-8.88 to 9.05) |
| 235.01 (161.29 to 335.14) | 235.43 (161.88 to 336.17) | 234.36 (160.46 to 337.08) | 232.21 (157.55 to 338.64) | 229.7 (154.42 to 338.91) | 227.36 (151.88 to 337.14) | 225.41 (150.55 to 334.21) | 221.59 (147.6 to 325.94) | 9.08 (2.41 to 15.2) |
| 461.93 (323.48 to 642.03) | 465.3 (325.85 to 646.65) | 466.31 (326.58 to 648.03) | 465.88 (326.3 to 647.39) | 465.05 (325.74 to 646.2) | 464.43 (325.31 to 645.3) | 463.6 (324.74 to 644.12) | 460.99 (322.5 to 640.23) | 8.4 (-5.21 to 27.49) |
| 283.71 (192.26 to 420.35) | 285.55 (193.51 to 423.07) | 287.18 (194.62 to 425.48) | 288.52 (195.53 to 427.45) | 289.57 (196.25 to 429) | 290.44 (196.84 to 430.28) | 291.23 (197.38 to 431.45) | 291.83 (197.78 to 432.32) | -1.48 (-1.61 to -1.37) |
| 256.43 (170.37 to 379.46) | 260.25 (170.33 to 381.41) | 263.72 (175.56 to 387.43) | 267.64 (179.81 to 387.8) | 271.49 (182.13 to 395.83) | 274.65 (183.49 to 399.09) | 269.2 (181.23 to 402.35) | 277.73 (186.23 to 414.86) | -3.33 (-12.1 to 5.98) |
| 189.69 (129.21 to 277.9) | 189.92 (128.42 to 278.28) | 190.86 (129.26 to 277.84) | 192.47 (130.65 to 279.95) | 193.67 (131.83 to 283.09) | 193.53 (132.1 to 284.06) | 192.65 (131.58 to 282.82) | 191.79 (131.04 to 281.57) | -4.55 (-6.94 to -2.2) |
| 186.51 (123.32 to 270.32) | 187.86 (124.21 to 272.26) | 190.07 (125.67 to 275.46) | 192.3 (127.14 to 278.69) | 193.85 (128.17 to 280.91) | 194.84 (128.83 to 282.31) | 195.27 (129.12 to 282.88) | 194.45 (128.59 to 281.64) | -2.3 (-12.53 to 7.46) |
| 387.55 (261.87 to 578.1) | 389.19 (262.53 to 580.56) | 391.29 (263.94 to 583.68) | 393.58 (265.49 to 587.09) | 395.5 (266.79 to 589.94) | 396.86 (267.71 to 591.96) | 397.97 (268.47 to 593.59) | 398.74 (268.99 to 594.72) | 0.79 (-1.53 to 2.43) |
| 135.92 (91.27 to 201.1) | 137.07 (92.04 to 202.81) | 137.45 (92.29 to 203.38) | 137.08 (92.04 to 202.85) | 136.18 (91.43 to 201.52) | 135.18 (90.75 to 200.06) | 134.41 (90.23 to 198.94) | 133.91 (89.89 to 198.21) | 0.1 (0 to 0.18) |
| 144.14 (95.04 to 216.6) | 146.87 (96.84 to 220.72) | 149.03 (98.27 to 223.99) | 150.31 (99.11 to 225.91) | 150.43 (99.2 to 226.07) | 149.31 (98.47 to 224.35) | 147.16 (97.06 to 221.07) | 144.09 (95.05 to 216.41) | 0.26 (-0.04 to 0.53) |
| 138.96 (93.03 to 207.14) | 141.97 (95.04 to 211.62) | 144.56 (96.78 to 215.49) | 146.56 (98.12 to 218.46) | 147.85 (98.98 to 220.38) | 148.5 (99.43 to 221.36) | 148.65 (99.53 to 221.57) | 148.35 (99.33 to 221.13) | 2.26 (2.06 to 2.42) |
| 166.96 (112.99 to 245.12) | 168.43 (113.85 to 247.43) | 169.28 (115.24 to 249) | 169.17 (115.8 to 249.44) | 168.54 (115.49 to 248.98) | 167.9 (115.29 to 248.61) | 167.11 (113.88 to 247.99) | 165.87 (113.06 to 246.15) | -11.44 (-15.03 to -7.35) |
| 76.02 (50.62 to 112.08) | 76.16 (50.58 to 112.85) | 76.04 (50.5 to 112.64) | 75.89 (50.41 to 112.39) | 75.7 (50.28 to 112.08) | 75.45 (50.12 to 111.68) | 75.15 (49.93 to 111.2) | 74.8 (49.7 to 110.66) | -11.56 (-18.42 to -5.05) |
| 126.48 (84.94 to 187.1) | 126.55 (84.65 to 187.3) | 126.58 (84.66 to 187.33) | 126.52 (84.61 to 187.2) | 126.33 (84.47 to 186.9) | 126.02 (84.25 to 186.4) | 125.6 (83.96 to 185.76) | 125.1 (83.62 to 184.99) | -8.97 (-13.5 to -6.48) |
| 326.14 (221.48 to 481.64) | 329.33 (224.43 to 489.41) | 331.41 (226.34 to 488.9) | 333.89 (229.55 to 490.05) | 338.71 (232.57 to 495.19) | 344.37 (235.65 to 500.68) | 350.68 (240.11 to 509.49) | 355.21 (242.15 to 513.88) | 40.62 (29.55 to 53.05) |
| 160.76 (109.26 to 240.66) | 161.89 (110.03 to 242.34) | 163.01 (110.8 to 244.03) | 164.03 (111.49 to 245.55) | 164.88 (112.06 to 246.81) | 165.52 (112.5 to 247.78) | 165.97 (112.81 to 248.44) | 166.21 (112.97 to 248.8) | 0 (-0.01 to 0.01) |
| 82.17 (53.53 to 122.93) | 82.05 (53.45 to 122.76) | 81.86 (53.33 to 122.47) | 81.61 (53.16 to 122.09) | 81.31 (52.97 to 121.65) | 81 (52.76 to 121.18) | 80.67 (52.55 to 120.7) | 80.34 (52.33 to 120.21) | 0.8 (-4.64 to 5.81) |
| 76.18 (49.97 to 113.35) | 75.94 (49.82 to 112.99) | 75.69 (49.66 to 112.63) | 75.43 (49.49 to 112.24) | 75.15 (49.31 to 111.83) | 74.86 (49.13 to 111.41) | 74.6 (48.96 to 111.02) | 74.35 (48.8 to 110.65) | -3.68 (-4.26 to -3.02) |
| 78.75 (51.94 to 116.43) | 78.76 (51.95 to 116.44) | 78.67 (51.89 to 116.3) | 78.48 (51.76 to 116.02) | 78.2 (51.58 to 115.61) | 77.85 (51.35 to 115.09) | 77.42 (51.07 to 114.46) | 76.93 (50.74 to 113.72) | -0.78 (-0.83 to -0.74) |
| 72.67 (47.23 to 108.46) | 73.14 (47.53 to 109.17) | 73.45 (47.73 to 109.65) | 73.58 (47.82 to 109.86) | 73.54 (47.79 to 109.81) | 73.32 (47.65 to 109.5) | 72.95 (47.4 to 108.95) | 72.45 (47.08 to 108.23) | -1.46 (-1.76 to -1.09) |

Abbreviations: UI, uncertainty interval.

**eTable** **5c. DALYs caused by ADHD by region and year, <20 years, 1990-2021.**

| **Regions** | 1990 | 1991 | 1992 | 1993 | 1994 | 1995 | 1996 | 1997 |
| --- | --- | --- | --- | --- | --- | --- | --- | --- |
| Global | 23.18 (12.03 to 38.79) | 23.39 (12.1 to 39.33) | 23.57 (12.14 to 39.67) | 23.74 (12.17 to 39.98) | 23.88 (12.18 to 40.39) | 23.98 (12.18 to 40.58) | 24.05 (12.26 to 40.52) | 24.11 (12.36 to 40.56) |
| High-income Asia Pacific | 29.17 (14.52 to 49.58) | 29.22 (14.71 to 49.65) | 29.15 (14.72 to 49.58) | 29.08 (14.81 to 49.51) | 28.94 (14.77 to 49.04) | 28.76 (14.65 to 48.79) | 28.62 (14.57 to 48.61) | 28.5 (14.73 to 48.29) |
| High-income North America | 39.39 (20.52 to 67.13) | 40.08 (20.77 to 68.44) | 40.73 (21.25 to 69.47) | 41.36 (21.6 to 71.04) | 41.96 (21.94 to 71.77) | 42.43 (22.16 to 72.73) | 42.78 (22.46 to 73.51) | 43.05 (22.47 to 73.55) |
| Western Europe | 29.65 (15.87 to 49.55) | 29.44 (15.91 to 49.13) | 29.27 (15.89 to 48.87) | 29.12 (15.68 to 49.1) | 28.99 (15.58 to 48.68) | 28.89 (15.38 to 48.6) | 28.88 (15.55 to 48.75) | 28.96 (15.75 to 48.32) |
| Australasia | 62.92 (33.11 to 107.6) | 62.74 (33.46 to 106.28) | 62.65 (32.98 to 106.23) | 62.53 (32.64 to 104.57) | 62.59 (32.62 to 105.02) | 62.83 (32.64 to 105.8) | 63.06 (32.88 to 107.8) | 63.27 (33.62 to 106.92) |
| Andean Latin America | 40.15 (20.61 to 69) | 40.26 (20.64 to 69.67) | 40.21 (20.83 to 69.36) | 40.23 (20.58 to 68.13) | 40.28 (20.67 to 69.22) | 40.34 (20.7 to 67.84) | 40.41 (20.74 to 68.94) | 40.53 (20.86 to 68.53) |
| Tropical Latin America | 41.14 (21.23 to 69.61) | 40.25 (20.82 to 68.2) | 39.43 (20.33 to 66.04) | 38.76 (19.85 to 64.7) | 38.3 (19.61 to 63.93) | 38.1 (19.4 to 63.6) | 38.16 (19.49 to 63.51) | 38.25 (19.55 to 63.77) |
| Central Latin America | 27.31 (14.35 to 46.12) | 27.24 (14.36 to 46.17) | 27.22 (14.11 to 45.64) | 27.22 (14.07 to 45.76) | 27.25 (14.13 to 45.31) | 27.32 (14.06 to 45.39) | 27.4 (13.99 to 45.38) | 27.5 (14.12 to 45.58) |
| Southern Latin America | 26.37 (13.58 to 45.08) | 26.4 (13.49 to 45.91) | 26.46 (13.87 to 45.58) | 26.52 (13.92 to 45.61) | 26.55 (13.8 to 46.05) | 26.66 (13.78 to 46) | 26.76 (13.87 to 46.47) | 26.84 (13.88 to 46.5) |
| Caribbean | 56.5 (29.72 to 95.25) | 56.46 (29.71 to 95.46) | 56.39 (29.44 to 95.44) | 56.53 (29.73 to 95.34) | 56.7 (29.71 to 94.75) | 56.9 (29.86 to 95.66) | 57.13 (29.97 to 96.25) | 57.27 (30.09 to 96.34) |
| Central Europe | 20.57 (10.25 to 35.31) | 20.66 (10.38 to 35.44) | 20.79 (10.38 to 35.83) | 20.92 (10.55 to 36.12) | 21.04 (10.53 to 36.16) | 21.18 (10.7 to 36.23) | 21.28 (10.67 to 36.25) | 21.38 (10.71 to 36.67) |
| Eastern Europe | 20.29 (10.51 to 35.16) | 20.58 (10.71 to 35.76) | 20.94 (10.85 to 36.16) | 21.33 (11.14 to 36.71) | 21.69 (11.31 to 37.67) | 22.04 (11.42 to 38.19) | 22.33 (11.57 to 38.66) | 22.58 (11.61 to 38.94) |
| Central Asia | 18.41 (9.07 to 31.51) | 18.51 (9.04 to 31.93) | 18.62 (9.17 to 32.21) | 18.78 (9.29 to 32.57) | 18.97 (9.34 to 32.86) | 19.2 (9.41 to 33.17) | 19.43 (9.49 to 33.74) | 19.67 (9.67 to 33.94) |
| North Africa and Middle East | 23.91 (12.12 to 40.73) | 24.14 (12.13 to 40.74) | 24.27 (12.24 to 41.22) | 24.33 (12.33 to 41.29) | 24.36 (12.45 to 41.02) | 24.39 (12.29 to 41.11) | 24.55 (12.5 to 41.2) | 24.94 (12.71 to 42.32) |
| South Asia | 10.61 (5.52 to 18.04) | 10.52 (5.42 to 18.08) | 10.42 (5.35 to 17.89) | 10.35 (5.31 to 17.58) | 10.3 (5.31 to 17.43) | 10.29 (5.16 to 17.69) | 10.29 (5.25 to 17.53) | 10.29 (5.2 to 17.46) |
| Southeast Asia | 18.95 (9.61 to 32.13) | 18.92 (9.5 to 32.13) | 18.94 (9.52 to 32.19) | 18.94 (9.49 to 32.41) | 18.92 (9.56 to 32.19) | 18.9 (9.59 to 32.31) | 18.91 (9.67 to 32.34) | 18.91 (9.67 to 32.09) |
| East Asia | 38.23 (19.87 to 64.53) | 39.83 (20.8 to 67.27) | 41.41 (21.53 to 70.23) | 42.96 (22.36 to 73.15) | 44.35 (22.78 to 76.16) | 45.47 (23.29 to 78.05) | 46.3 (23.73 to 78.3) | 47.06 (24.17 to 78.76) |
| Oceania | 20.75 (10.68 to 35.68) | 20.73 (10.62 to 35.93) | 20.77 (10.5 to 35.68) | 20.79 (10.82 to 35.8) | 20.81 (10.69 to 35.78) | 20.77 (10.63 to 36.11) | 20.75 (10.58 to 35.73) | 20.81 (10.73 to 36.02) |
| Western Sub-Saharan Africa | 9.3 (4.69 to 15.99) | 9.32 (4.71 to 16.02) | 9.34 (4.69 to 16.11) | 9.36 (4.71 to 15.88) | 9.38 (4.72 to 15.87) | 9.4 (4.76 to 15.87) | 9.41 (4.79 to 15.91) | 9.43 (4.73 to 15.92) |
| Eastern Sub-Saharan Africa | 9.17 (4.66 to 15.95) | 9.19 (4.69 to 15.89) | 9.21 (4.69 to 15.82) | 9.22 (4.72 to 15.77) | 9.23 (4.73 to 15.83) | 9.24 (4.71 to 15.85) | 9.24 (4.72 to 15.8) | 9.24 (4.79 to 15.82) |
| Central Sub-Saharan Africa | 8.99 (4.58 to 15.27) | 9.01 (4.45 to 15.58) | 9.01 (4.64 to 15.93) | 8.99 (4.5 to 15.59) | 9.05 (4.41 to 15.39) | 9.04 (4.54 to 15.4) | 9.08 (4.6 to 15.56) | 9.05 (4.56 to 15.57) |
| Southern Sub-Saharan Africa | 9.74 (4.97 to 16.88) | 9.78 (4.99 to 16.85) | 9.82 (5.13 to 17.07) | 9.88 (5.13 to 17.19) | 9.92 (5.13 to 16.99) | 9.95 (5.17 to 17.31) | 9.99 (5.14 to 17.38) | 10.04 (5.12 to 17.41) |

| 1998 | 1999 | 2000 | 2001 | 2002 | 2003 | 2004 | 2005 |
| --- | --- | --- | --- | --- | --- | --- | --- |
| 24.14 (12.45 to 40.63) | 24.14 (12.43 to 40.41) | 24.07 (12.48 to 40.21) | 23.95 (12.41 to 40.11) | 23.81 (12.29 to 40.01) | 23.61 (12.24 to 39.68) | 23.39 (12.1 to 39.23) | 23.15 (11.91 to 38.66) |
| 28.39 (14.48 to 47.65) | 28.32 (14.55 to 47.56) | 28.26 (14.88 to 47.85) | 28.22 (14.48 to 47.74) | 28.24 (14.67 to 47.35) | 28.3 (14.8 to 47.47) | 28.35 (14.91 to 47.23) | 28.48 (15 to 47.39) |
| 43.26 (22.75 to 73.87) | 43.43 (22.55 to 74.38) | 43.55 (22.47 to 74.39) | 43.69 (22.55 to 74.54) | 43.87 (22.58 to 75.31) | 44.02 (22.62 to 74.78) | 44.15 (22.46 to 74.14) | 44.14 (22.38 to 73.89) |
| 29.04 (15.66 to 48.42) | 29.13 (15.91 to 48.52) | 29.18 (15.94 to 48.62) | 29.23 (15.76 to 48.33) | 29.37 (16.15 to 48.72) | 29.51 (16.04 to 48.87) | 29.59 (16.16 to 49.3) | 29.63 (16.18 to 49.28) |
| 63.49 (33.73 to 108.75) | 63.95 (33.79 to 108.61) | 64.17 (33.29 to 108.83) | 64.54 (33.53 to 110.03) | 65.03 (35.01 to 107.15) | 65.35 (35.79 to 106.71) | 65.58 (36.24 to 105.77) | 65.62 (35.94 to 104.18) |
| 40.69 (21.09 to 69.44) | 40.76 (21.15 to 68.21) | 40.9 (20.97 to 69.94) | 41.11 (21.2 to 69.94) | 41.27 (21.4 to 70.95) | 41.4 (20.94 to 70.13) | 41.57 (21.06 to 70.81) | 41.73 (21.31 to 71.39) |
| 38.4 (19.8 to 64.71) | 38.56 (19.91 to 65.56) | 38.66 (19.83 to 65.02) | 38.76 (20.27 to 65.61) | 38.94 (20.35 to 65.87) | 39.14 (20.47 to 65.7) | 39.38 (20.72 to 66.75) | 39.52 (20.86 to 67.18) |
| 27.61 (14.15 to 46.04) | 27.73 (14.35 to 46.21) | 27.85 (14.5 to 46.28) | 27.99 (14.41 to 46.49) | 28.09 (14.63 to 46.68) | 28.19 (14.63 to 46.92) | 28.26 (14.67 to 47.38) | 28.37 (14.69 to 47.14) |
| 26.93 (13.91 to 46.3) | 27.01 (14.06 to 47.03) | 27.06 (14.18 to 46.13) | 27.11 (13.77 to 46.27) | 27.18 (13.87 to 46.48) | 27.21 (13.88 to 46.2) | 27.19 (14.29 to 45.49) | 27.24 (14.01 to 45.94) |
| 57.45 (30.29 to 96.84) | 57.64 (30.14 to 96.93) | 57.82 (30.32 to 97.93) | 58 (30.42 to 98.02) | 58.2 (30.27 to 98.99) | 58.4 (30.46 to 98.54) | 58.58 (30.52 to 99.48) | 58.74 (30.77 to 99.09) |
| 21.47 (10.93 to 36.68) | 21.52 (10.94 to 36.84) | 21.55 (10.75 to 36.59) | 21.56 (10.74 to 36.85) | 21.54 (10.81 to 36.92) | 21.47 (10.86 to 36.8) | 21.4 (10.82 to 36.9) | 21.28 (10.7 to 36.28) |
| 22.74 (11.71 to 39.11) | 22.85 (11.89 to 39.22) | 22.89 (11.94 to 39.27) | 22.83 (11.88 to 38.82) | 22.68 (11.85 to 38.94) | 22.45 (11.76 to 38.7) | 22.16 (11.36 to 37.94) | 21.87 (11.23 to 37.57) |
| 19.9 (9.77 to 34.5) | 20.11 (9.73 to 34.79) | 20.27 (9.96 to 34.91) | 20.45 (10.01 to 35.2) | 20.5 (10.08 to 35.48) | 20.54 (10.01 to 35.02) | 20.52 (10.26 to 35.54) | 20.46 (10.18 to 35) |
| 25.34 (12.98 to 42.8) | 25.65 (13.42 to 43.27) | 25.71 (13.22 to 43.42) | 25.55 (13.3 to 42.92) | 25.31 (13.17 to 42.67) | 25.01 (13.05 to 41.76) | 24.74 (12.98 to 41.35) | 24.48 (12.84 to 40.74) |
| 10.29 (5.28 to 17.66) | 10.29 (5.24 to 17.74) | 10.32 (5.24 to 17.86) | 10.36 (5.3 to 17.78) | 10.44 (5.27 to 18.02) | 10.52 (5.3 to 18.23) | 10.6 (5.38 to 18.4) | 10.62 (5.44 to 18.3) |
| 18.89 (9.6 to 32.41) | 18.9 (9.63 to 32.21) | 18.9 (9.61 to 31.9) | 18.88 (9.55 to 32.16) | 18.88 (9.57 to 32.22) | 18.85 (9.49 to 32.06) | 18.84 (9.57 to 32.08) | 18.81 (9.49 to 32.05) |
| 47.68 (24.59 to 79.16) | 48.12 (24.83 to 80.25) | 48.36 (25.04 to 81.53) | 48.33 (25.02 to 80.41) | 48.07 (24.74 to 78.98) | 47.62 (24.74 to 77.57) | 47.16 (24.37 to 76.36) | 46.79 (24.13 to 75.42) |
| 20.8 (10.53 to 36.18) | 20.85 (10.47 to 36.04) | 20.88 (10.65 to 36.24) | 20.95 (10.76 to 36.24) | 20.95 (10.77 to 36.49) | 21.01 (10.64 to 35.93) | 21.06 (10.85 to 36.37) | 21.15 (10.75 to 36.79) |
| 9.44 (4.83 to 16.03) | 9.45 (4.8 to 16.02) | 9.46 (4.79 to 16.02) | 9.49 (4.79 to 16.05) | 9.52 (4.83 to 16.02) | 9.58 (4.81 to 16.07) | 9.62 (4.85 to 16.29) | 9.64 (4.85 to 16.29) |
| 9.23 (4.67 to 15.95) | 9.24 (4.73 to 15.97) | 9.25 (4.68 to 16.04) | 9.24 (4.67 to 15.97) | 9.24 (4.77 to 16.04) | 9.27 (4.7 to 15.99) | 9.27 (4.7 to 15.97) | 9.29 (4.75 to 15.96) |
| 9.07 (4.55 to 15.68) | 9.08 (4.66 to 15.68) | 9.1 (4.77 to 15.69) | 9.1 (4.62 to 15.57) | 9.13 (4.73 to 15.43) | 9.13 (4.63 to 15.57) | 9.1 (4.71 to 15.57) | 9.12 (4.6 to 15.73) |
| 10.08 (5.13 to 17.54) | 10.12 (5.22 to 17.47) | 10.15 (5.27 to 17.62) | 10.18 (5.2 to 18.06) | 10.18 (5.16 to 17.7) | 10.18 (5.19 to 17.64) | 10.15 (5.13 to 17.7) | 10.14 (5.22 to 17.73) |

| 2006 | 2007 | 2008 | 2009 | 2010 | 2011 | 2012 | 2013 |
| --- | --- | --- | --- | --- | --- | --- | --- |
| 22.93 (11.85 to 38.36) | 22.7 (11.75 to 37.82) | 22.48 (11.62 to 37.66) | 22.25 (11.54 to 37.32) | 22.03 (11.51 to 37.01) | 21.82 (11.37 to 36.57) | 21.66 (11.19 to 36.49) | 21.53 (11.12 to 36.2) |
| 28.55 (14.85 to 47.43) | 28.61 (14.86 to 47.81) | 28.62 (14.88 to 47.93) | 28.58 (14.8 to 48.14) | 28.49 (14.64 to 47.27) | 28.5 (14.8 to 47.35) | 28.57 (14.71 to 47.64) | 28.65 (14.96 to 48.34) |
| 44.04 (22.28 to 73.37) | 43.87 (22.28 to 74.02) | 43.74 (22.42 to 74.41) | 43.68 (22.3 to 74.95) | 43.66 (22.27 to 76.18) | 43.67 (22.41 to 74.94) | 43.55 (22.27 to 74.25) | 43.36 (22.25 to 74.07) |
| 29.65 (16.15 to 49.36) | 29.72 (16.02 to 49.41) | 29.83 (16.09 to 49.84) | 29.93 (15.96 to 50.07) | 30.04 (16.05 to 50.06) | 30.17 (16.06 to 50.17) | 30.41 (16.36 to 50.79) | 30.71 (16.46 to 51.41) |
| 65.49 (35.84 to 103.42) | 65.18 (35.77 to 104.05) | 64.94 (35.31 to 102.35) | 64.61 (35.33 to 103.2) | 64.43 (34.78 to 103.41) | 64.33 (34.69 to 103.25) | 64.19 (34.92 to 101.54) | 64.32 (34.62 to 101.73) |
| 41.84 (21.47 to 72.37) | 41.88 (21.51 to 70.96) | 41.86 (21.52 to 70.23) | 41.81 (21.05 to 71.93) | 41.78 (21.24 to 71.71) | 41.7 (21.37 to 71.42) | 41.62 (21.05 to 70.63) | 41.58 (20.86 to 70.74) |
| 39.6 (20.9 to 67.08) | 39.61 (20.39 to 66.41) | 39.62 (20.29 to 66.22) | 39.61 (20.54 to 66.36) | 39.6 (20.29 to 66.03) | 39.73 (20.76 to 66.72) | 40.1 (21.14 to 67.98) | 40.52 (21.32 to 68.78) |
| 28.44 (14.7 to 47.35) | 28.48 (14.82 to 47.5) | 28.51 (14.68 to 47.5) | 28.51 (14.81 to 47.68) | 28.5 (14.81 to 47.26) | 28.44 (14.84 to 47.65) | 28.4 (14.8 to 47.91) | 28.31 (14.69 to 47.32) |
| 27.22 (14.15 to 46.25) | 27.17 (14.03 to 45.94) | 27.18 (13.88 to 45.99) | 27.1 (13.62 to 45.75) | 27.02 (13.91 to 45.43) | 26.9 (13.96 to 45.1) | 26.81 (13.63 to 44.78) | 26.76 (13.72 to 45.08) |
| 58.8 (30.59 to 99.81) | 58.96 (30.85 to 99.85) | 58.98 (30.65 to 99.76) | 58.89 (30.77 to 99.32) | 58.87 (30.62 to 99.81) | 58.85 (30.86 to 99.41) | 58.72 (30.43 to 100.19) | 58.63 (30.8 to 99.32) |
| 21.15 (10.7 to 36.21) | 20.95 (10.68 to 35.64) | 20.75 (10.46 to 35.47) | 20.55 (10.3 to 35.15) | 20.39 (10.16 to 34.91) | 20.3 (10.26 to 34.56) | 20.23 (10.13 to 34.56) | 20.23 (10.08 to 34.77) |
| 21.54 (11.11 to 36.91) | 21.17 (10.8 to 36.38) | 20.81 (10.7 to 35.96) | 20.43 (10.52 to 35.21) | 20.08 (10.5 to 34.91) | 19.8 (10.26 to 34.25) | 19.59 (10.02 to 34.05) | 19.49 (10.1 to 33.84) |
| 20.3 (10.04 to 35.03) | 20.12 (9.97 to 34.67) | 19.92 (9.91 to 34.16) | 19.7 (9.84 to 33.56) | 19.44 (9.61 to 33) | 19.19 (9.46 to 32.78) | 18.97 (9.31 to 32.68) | 18.78 (9.29 to 32.45) |
| 24.24 (12.87 to 40.32) | 23.95 (12.49 to 40.07) | 23.66 (12.34 to 39.58) | 23.38 (12.17 to 39.31) | 23.13 (12.13 to 38.98) | 22.9 (11.91 to 38.19) | 22.72 (11.74 to 38.38) | 22.6 (11.72 to 38.24) |
| 10.65 (5.48 to 18.7) | 10.66 (5.49 to 18.42) | 10.67 (5.41 to 18.47) | 10.68 (5.5 to 18.39) | 10.71 (5.53 to 18.77) | 10.75 (5.54 to 18.66) | 10.88 (5.54 to 18.68) | 11.01 (5.65 to 18.97) |
| 18.77 (9.51 to 32.04) | 18.73 (9.44 to 31.93) | 18.67 (9.52 to 31.72) | 18.63 (9.5 to 31.52) | 18.56 (9.4 to 31.49) | 18.45 (9.29 to 31.28) | 18.4 (9.22 to 31.55) | 18.34 (9.14 to 31.39) |
| 46.65 (24.28 to 75.87) | 46.63 (24.52 to 76.01) | 46.62 (24.66 to 77.08) | 46.56 (24.52 to 76.86) | 46.43 (24.69 to 76.98) | 46.25 (24.42 to 76.12) | 45.83 (24 to 76.39) | 45.3 (23.72 to 75.67) |
| 21.12 (10.82 to 37.16) | 21.17 (10.62 to 36.51) | 21.09 (10.92 to 36.28) | 21.06 (10.79 to 36.09) | 21.03 (10.88 to 35.92) | 20.98 (10.67 to 36.03) | 20.89 (10.81 to 36.35) | 20.85 (10.65 to 35.99) |
| 9.65 (4.9 to 16.27) | 9.64 (4.85 to 16.32) | 9.65 (4.84 to 16.17) | 9.66 (4.84 to 16.28) | 9.67 (4.83 to 16.18) | 9.68 (4.9 to 16.28) | 9.71 (4.88 to 16.34) | 9.74 (4.88 to 16.41) |
| 9.31 (4.75 to 16.01) | 9.33 (4.77 to 16.24) | 9.38 (4.75 to 16.16) | 9.41 (4.76 to 16.3) | 9.43 (4.83 to 16.29) | 9.48 (4.82 to 16.34) | 9.52 (4.85 to 16.32) | 9.55 (4.86 to 16.36) |
| 9.08 (4.6 to 15.37) | 9.11 (4.66 to 15.45) | 9.15 (4.77 to 15.55) | 9.1 (4.57 to 15.45) | 9.16 (4.57 to 15.65) | 9.15 (4.64 to 15.46) | 9.2 (4.64 to 15.46) | 9.2 (4.6 to 15.82) |
| 10.1 (5.1 to 17.73) | 10.06 (5.22 to 17.44) | 10.02 (5.15 to 17.49) | 9.98 (5.12 to 17.17) | 9.96 (5.11 to 17.24) | 9.92 (5.1 to 17.18) | 9.89 (5.15 to 17.01) | 9.88 (5.01 to 17.08) |

| 2014 | 2015 | 2016 | 2017 | 2018 | 2019 | 2020 | 2021 | **Percentage change from 1990 to 2021 (%)** |
| --- | --- | --- | --- | --- | --- | --- | --- | --- |
| 21.43 (11.01 to 35.96) | 21.36 (10.91 to 36) | 21.31 (10.9 to 35.9) | 21.31 (10.86 to 36.14) | 21.37 (10.89 to 36.14) | 21.48 (10.97 to 36.43) | 21.56 (10.92 to 36.45) | 21.81 (11.16 to 37.09) | -5.88 (-8.45 to -3.29) |
| 28.71 (14.8 to 47.55) | 28.69 (14.77 to 48.04) | 28.5 (14.69 to 47.74) | 28.2 (14.53 to 47.02) | 27.97 (14.29 to 46.53) | 27.96 (14.28 to 46.74) | 28.14 (14.36 to 46.91) | 28.71 (14.53 to 48.89) | -1.59 (-6.4 to 3.67) |
| 43.23 (22.03 to 74.22) | 43.14 (21.83 to 74.21) | 43.38 (22.07 to 74.64) | 43.9 (21.98 to 75.87) | 44.41 (22.07 to 76.41) | 44.69 (22.28 to 77.45) | 44.39 (22.23 to 77.36) | 44.44 (22.32 to 77.6) | 12.83 (1.75 to 22.75) |
| 30.97 (16.63 to 51.17) | 31.11 (16.61 to 51.55) | 31.14 (16.41 to 51.59) | 31.07 (16.06 to 52.22) | 31.01 (16.05 to 52.31) | 31.07 (15.8 to 53.05) | 31.14 (15.81 to 52.99) | 31.01 (15.84 to 52.32) | 4.58 (-2.3 to 11.58) |
| 64.49 (34.77 to 102.81) | 64.54 (34.69 to 104.16) | 64.55 (34.58 to 103.08) | 64.73 (34.68 to 102.77) | 64.89 (34.89 to 102.84) | 65.26 (35.54 to 103.75) | 65.58 (34.91 to 105.87) | 65.75 (35.06 to 104.44) | 4.5 (-9.34 to 24.24) |
| 41.53 (20.98 to 71.2) | 41.51 (20.81 to 70.89) | 41.46 (21.13 to 70.86) | 41.45 (21.24 to 71.29) | 41.48 (20.94 to 71.41) | 41.56 (20.93 to 71.96) | 41.57 (21.59 to 70.94) | 41.64 (21.43 to 70.79) | 3.7 (-0.8 to 8.08) |
| 40.83 (21.53 to 69.51) | 40.95 (21.68 to 70.53) | 40.97 (21.62 to 70.42) | 41 (21.64 to 69.95) | 41.05 (21.22 to 69.8) | 41.06 (21.17 to 69.55) | 39.9 (20.15 to 67.06) | 40.84 (21.46 to 70.43) | -0.72 (-9.98 to 9.11) |
| 28.27 (14.62 to 47.35) | 28.28 (14.79 to 47.53) | 28.38 (14.96 to 47.55) | 28.59 (14.97 to 47.63) | 28.77 (15.11 to 47.57) | 28.84 (15.11 to 47.47) | 28.86 (15.24 to 47.91) | 28.97 (15.26 to 48.04) | 6.09 (2.55 to 10.02) |
| 26.64 (13.99 to 44.66) | 26.66 (13.65 to 44.75) | 26.71 (13.86 to 44.71) | 26.77 (13.72 to 44.81) | 26.94 (14.01 to 45.65) | 27.21 (14 to 45.9) | 27.54 (14.43 to 46.11) | 27.81 (14.34 to 46.95) | 5.43 (-5.4 to 17.34) |
| 58.58 (30.71 to 98.98) | 58.52 (30.58 to 98.73) | 58.45 (30.47 to 99.72) | 58.41 (30.17 to 99.76) | 58.37 (30.07 to 99.69) | 58.41 (30.48 to 99.76) | 58.47 (30.42 to 99.99) | 58.5 (30.66 to 99.93) | 3.54 (-0.32 to 6.99) |
| 20.27 (10.3 to 34.74) | 20.31 (10.2 to 34.91) | 20.34 (10.28 to 34.91) | 20.35 (10.36 to 34.96) | 20.38 (10.37 to 35.17) | 20.43 (10.27 to 35.26) | 20.48 (10.27 to 35.12) | 20.56 (10.42 to 35.09) | -0.08 (-2.95 to 3.23) |
| 19.49 (10.03 to 33.87) | 19.55 (10.15 to 33.91) | 19.68 (10.15 to 33.92) | 19.92 (10.25 to 34.52) | 20.26 (10.56 to 35.13) | 20.61 (10.72 to 35.81) | 20.99 (10.85 to 36.18) | 21.34 (10.98 to 37.01) | 5.17 (2.36 to 7.83) |
| 18.65 (9.23 to 32.16) | 18.6 (9.14 to 32.08) | 18.53 (9.16 to 32.16) | 18.56 (9.09 to 32.23) | 18.61 (9.27 to 32.33) | 18.7 (9.3 to 32.43) | 18.76 (9.1 to 32.69) | 18.9 (9.3 to 33.15) | 2.65 (-2.42 to 7.6) |
| 22.53 (11.55 to 38.28) | 22.53 (11.5 to 38.09) | 22.5 (11.6 to 38.09) | 22.41 (11.57 to 37.9) | 22.35 (11.38 to 38.05) | 22.43 (11.38 to 38.12) | 22.57 (11.52 to 38.28) | 22.79 (11.54 to 38.6) | -4.69 (-9.17 to 0.19) |
| 11.13 (5.7 to 18.8) | 11.19 (5.67 to 19.1) | 11.25 (5.64 to 18.97) | 11.3 (5.65 to 19.14) | 11.33 (5.7 to 19.31) | 11.37 (5.72 to 19.15) | 11.41 (5.72 to 19.18) | 11.45 (5.68 to 19.35) | 7.96 (-0.65 to 16.8) |
| 18.31 (9.21 to 31) | 18.28 (9.08 to 31.26) | 18.28 (9.07 to 31.05) | 18.29 (9.17 to 31.1) | 18.31 (9.29 to 30.96) | 18.32 (9.27 to 31.08) | 18.33 (9.17 to 31.59) | 18.35 (9.1 to 31.22) | -3.16 (-8.48 to 0.77) |
| 44.75 (23.23 to 75.29) | 44.26 (22.78 to 75.15) | 43.76 (22.64 to 74.4) | 43.49 (22.44 to 73.34) | 43.67 (22.5 to 73.33) | 44.14 (22.99 to 74.39) | 44.91 (23.33 to 74.81) | 46.49 (24.69 to 79.04) | 21.61 (12.09 to 32.76) |
| 20.82 (10.58 to 36.01) | 20.74 (10.72 to 35.36) | 20.72 (10.27 to 35.67) | 20.69 (10.75 to 36.04) | 20.71 (10.71 to 36) | 20.68 (10.88 to 35.49) | 20.74 (10.33 to 36.23) | 20.67 (10.49 to 35.77) | -0.35 (-8.35 to 7.62) |
| 9.78 (4.84 to 16.48) | 9.82 (4.98 to 16.55) | 9.86 (5.01 to 16.56) | 9.89 (4.99 to 16.64) | 9.94 (4.97 to 16.78) | 9.98 (5 to 16.95) | 10.02 (5.05 to 17) | 10.06 (5.04 to 16.93) | 8.14 (1.97 to 14.73) |
| 9.59 (4.86 to 16.52) | 9.62 (4.87 to 16.63) | 9.67 (4.92 to 16.52) | 9.7 (4.96 to 16.67) | 9.73 (5.09 to 16.65) | 9.77 (4.99 to 16.69) | 9.8 (4.96 to 17.04) | 9.81 (5.06 to 16.91) | 7.03 (2.94 to 11.17) |
| 9.25 (4.58 to 15.99) | 9.3 (4.76 to 15.72) | 9.39 (4.82 to 15.9) | 9.41 (4.71 to 16.02) | 9.51 (4.79 to 16.11) | 9.59 (4.74 to 16.33) | 9.6 (4.8 to 16.21) | 9.7 (4.95 to 16.74) | 7.88 (-2.3 to 19.87) |
| 9.89 (5.02 to 16.94) | 9.91 (5.1 to 17.43) | 9.94 (5.17 to 17.1) | 9.99 (5.14 to 17.21) | 10.03 (5.13 to 17.41) | 10.08 (5.25 to 17.55) | 10.13 (5.17 to 17.77) | 10.18 (5.21 to 17.74) | 4.52 (-0.75 to 10.69) |

Abbreviations: DALYs, disability-adjusted life-years; UI, uncertainty interval.

**eTable** **6a. Prevalence of ADHD by SDI group and year, <20 years, 1990-2021.**

|  | **Prevalence** |  |  |  |  |  |  |  |  |  |
| --- | --- | --- | --- | --- | --- | --- | --- | --- | --- | --- |
|  | **Low SDI** |  | **Low-middle SDI** |  | **Middle SDI** |  | **High-middle SDI** |  | **High SDI** |  |
| **Year** | **Counts** | **Rate  (per 100,000)** | **Counts** | **Rate  (per 100,000)** | **Counts** | **Rate  (per 100,000)** | **Counts** | **Rate  (per 100,000)** | **Counts** | **Rate  (per 100,000)** |
| 1990 | 2395167 (1602159 to 3506218) | 856.71 (573.07 to 1254.12) | 7045768 (4734845 to 10221310) | 1192.13 (801.13 to 1729.42) | 17364631 (11986625 to 24902963) | 2271.21 (1567.79 to 3257.19) | 9362099 (6473102 to 13386868) | 2529.16 (1748.7 to 3616.45) | 6527782 (4440401 to 9282835) | 2597.4 (1766.83 to 3693.63) |
| 1991 | 2447299 (1638348 to 3589215) | 854.61 (572.12 to 1253.38) | 7105031 (4782620 to 10349614) | 1185.71 (798.14 to 1727.17) | 17650922 (12184021 to 25291582) | 2300.02 (1587.65 to 3295.65) | 9607367 (6656383 to 13681523) | 2602.99 (1803.46 to 3706.82) | 6565006 (4474393 to 9361408) | 2619.49 (1785.32 to 3735.27) |
| 1992 | 2505163 (1678538 to 3670954) | 852.89 (571.46 to 1249.79) | 7167967 (4843688 to 10430222) | 1180.07 (797.42 to 1717.13) | 17905281 (12360096 to 25707413) | 2327.67 (1606.8 to 3341.94) | 9831469 (6825300 to 14025640) | 2676.22 (1857.91 to 3817.92) | 6611627 (4512991 to 9455973) | 2643.28 (1804.26 to 3780.42) |
| 1993 | 2569490 (1723357 to 3759985) | 851.71 (571.24 to 1246.33) | 7237269 (4910596 to 10524665) | 1175.48 (797.58 to 1709.41) | 18115324 (12490554 to 26057730) | 2352.32 (1621.93 to 3383.66) | 10027175 (6968294 to 14307326) | 2745.78 (1908.16 to 3917.83) | 6665521 (4554961 to 9563293) | 2668.46 (1823.52 to 3828.55) |
| 1994 | 2632239 (1767087 to 3845233) | 850.76 (571.14 to 1242.81) | 7316438 (4977308 to 10616769) | 1172.45 (797.61 to 1701.33) | 18293688 (12585144 to 26293743) | 2373.7 (1632.99 to 3411.75) | 10186637 (7089064 to 14561638) | 2808.41 (1954.42 to 4014.58) | 6719769 (4589980 to 9660761) | 2692.85 (1839.37 to 3871.41) |
| 1995 | 2696602 (1812171 to 3932325) | 850.12 (571.3 to 1239.68) | 7408472 (5042148 to 10733689) | 1171.53 (797.34 to 1697.36) | 18440958 (12660917 to 26557844) | 2390.28 (1641.08 to 3442.37) | 10294198 (7175961 to 14659655) | 2858.47 (1992.6 to 4070.65) | 6766284 (4614902 to 9740921) | 2713.78 (1850.91 to 3906.82) |
| 1996 | 2768384 (1865675 to 4035047) | 849.46 (572.47 to 1238.13) | 7507012 (5098696 to 10905019) | 1171.8 (795.88 to 1702.21) | 18585834 (12717232 to 26837580) | 2405.73 (1646.1 to 3473.82) | 10358877 (7199685 to 14729131) | 2897.69 (2013.97 to 4120.18) | 6807717 (4650544 to 9775394) | 2731.95 (1866.27 to 3922.88) |
| 1997 | 2843579 (1916019 to 4142142) | 848.72 (571.87 to 1236.29) | 7605377 (5151703 to 11023721) | 1172.13 (793.97 to 1698.96) | 18749711 (12801966 to 27149659) | 2424.04 (1655.09 to 3510.01) | 10399512 (7210433 to 14766013) | 2931.57 (2032.58 to 4162.46) | 6843385 (4666041 to 9810248) | 2747.95 (1873.64 to 3939.29) |
| 1998 | 2920177 (1966919 to 4259823) | 848.3 (571.38 to 1237.46) | 7703369 (5203479 to 11184940) | 1172.72 (792.15 to 1702.74) | 18895112 (12894079 to 27488883) | 2441.18 (1665.87 to 3551.47) | 10405183 (7196259 to 14751036) | 2957.51 (2045.42 to 4192.75) | 6869890 (4673318 to 9846490) | 2761.48 (1878.52 to 3957.97) |
| 1999 | 2997764 (2020513 to 4379124) | 848.25 (571.73 to 1239.12) | 7799633 (5254204 to 11327229) | 1173.6 (790.6 to 1704.4) | 18988446 (12952656 to 27654507) | 2453.2 (1673.41 to 3572.8) | 10368119 (7205327 to 14662077) | 2973.76 (2066.61 to 4205.34) | 6884492 (4670649 to 9901591) | 2773.09 (1881.35 to 3988.39) |
| 2000 | 3075957 (2074345 to 4495350) | 848.5 (572.2 to 1240.03) | 7893261 (5303641 to 11446804) | 1174.74 (789.33 to 1703.6) | 19008095 (12936913 to 27637484) | 2456.66 (1672 to 3571.94) | 10289050 (7189397 to 14511324) | 2979.39 (2081.83 to 4202.04) | 6885178 (4703417 to 9919229) | 2782.74 (1900.95 to 4008.99) |
| 2001 | 3157745 (2130473 to 4613161) | 849.64 (573.23 to 1241.24) | 7991019 (5382021 to 11596158) | 1177.09 (792.78 to 1708.13) | 18955964 (12934823 to 27483324) | 2450.82 (1672.35 to 3553.33) | 10169336 (7141980 to 14267776) | 2971.84 (2087.14 to 4169.55) | 6885793 (4679408 to 9862814) | 2793.07 (1898.1 to 4000.63) |
| 2002 | 3248843 (2192060 to 4745253) | 851.99 (574.86 to 1244.42) | 8093878 (5465313 to 11741171) | 1180.77 (797.31 to 1712.86) | 18843370 (12872810 to 27166286) | 2438.11 (1665.59 to 3515) | 10006822 (7059842 to 13961021) | 2952.32 (2082.87 to 4118.93) | 6890358 (4667357 to 9776071) | 2805.28 (1900.23 to 3980.15) |
| 2003 | 3348297 (2256829 to 4889265) | 854.92 (576.23 to 1248.37) | 8194205 (5542253 to 11884364) | 1184.46 (801.13 to 1717.87) | 18666461 (12770975 to 26626131) | 2420.13 (1655.77 to 3452.11) | 9803224 (6943002 to 13602158) | 2925.32 (2071.82 to 4058.93) | 6890566 (4680170 to 9732180) | 2816 (1912.67 to 3977.3) |
| 2004 | 3448403 (2322128 to 5033281) | 857.42 (577.38 to 1251.48) | 8283891 (5610885 to 12020323) | 1186.98 (803.97 to 1722.37) | 18435311 (12632089 to 26173890) | 2398.81 (1643.69 to 3405.75) | 9570585 (6800126 to 13268255) | 2895.15 (2057.07 to 4013.71) | 6886589 (4703233 to 9714232) | 2824.18 (1928.79 to 3983.79) |
| 2005 | 3545027 (2387590 to 5172906) | 858.89 (578.47 to 1253.3) | 8356018 (5674537 to 12126583) | 1187.33 (806.31 to 1723.1) | 18173812 (12459901 to 25877247) | 2376.34 (1629.21 to 3383.61) | 9330040 (6626220 to 12942278) | 2868.07 (2036.91 to 3978.48) | 6871299 (4703488 to 9683439) | 2827.52 (1935.47 to 3984.71) |
| 2006 | 3642249 (2452946 to 5314978) | 859.89 (579.11 to 1254.81) | 8415136 (5721726 to 12207735) | 1185.99 (806.39 to 1720.5) | 17911015 (12289947 to 25403266) | 2355.89 (1616.53 to 3341.36) | 9102410 (6479211 to 12557997) | 2848.31 (2027.46 to 3929.62) | 6848674 (4678136 to 9637972) | 2826.29 (1930.56 to 3977.37) |
| 2007 | 3742528 (2520327 to 5461404) | 861.09 (579.88 to 1256.57) | 8471101 (5764150 to 12286300) | 1184.29 (805.85 to 1717.67) | 17652153 (12132682 to 25160657) | 2336.97 (1606.25 to 3331.03) | 8887539 (6314679 to 12282275) | 2831.38 (2011.72 to 3912.87) | 6826423 (4645651 to 9624887) | 2823.27 (1921.35 to 3980.65) |
| 2008 | 3845370 (2589418 to 5611433) | 862.45 (580.76 to 1258.55) | 8524942 (5800526 to 12366937) | 1182.41 (804.53 to 1715.29) | 17396895 (11973899 to 24916538) | 2317.26 (1594.92 to 3318.88) | 8676490 (6150189 to 12056252) | 2812.69 (1993.73 to 3908.32) | 6801823 (4610062 to 9611463) | 2820.17 (1911.43 to 3985.11) |
| 2009 | 3952397 (2661375 to 5767321) | 864.1 (581.85 to 1260.9) | 8577354 (5837410 to 12447183) | 1180.42 (803.35 to 1712.99) | 17144450 (11814852 to 24570438) | 2295.96 (1582.23 to 3290.44) | 8465448 (5983950 to 11797128) | 2790.56 (1972.56 to 3888.82) | 6774384 (4590995 to 9597225) | 2818.42 (1910.04 to 3992.83) |
| 2010 | 4060248 (2733881 to 5924143) | 865.71 (582.91 to 1263.13) | 8628523 (5873954 to 12525250) | 1178.41 (802.21 to 1710.59) | 16885878 (11649964 to 24102311) | 2274.15 (1568.99 to 3246.04) | 8263496 (5826967 to 11512951) | 2767.02 (1951.15 to 3855.1) | 6741868 (4568223 to 9599017) | 2818.14 (1909.55 to 4012.45) |
| 2011 | 4173609 (2814804 to 6087434) | 868.56 (585.79 to 1266.85) | 8691971 (5914325 to 12591018) | 1178.35 (801.79 to 1706.93) | 16654596 (11497318 to 23809975) | 2254.58 (1556.42 to 3223.22) | 8084695 (5698827 to 11269301) | 2741.86 (1932.71 to 3821.9) | 6713655 (4543766 to 9557637) | 2818.19 (1907.34 to 4012.01) |
| 2012 | 4296496 (2901426 to 6265690) | 873.25 (589.71 to 1273.49) | 8773485 (5978353 to 12710987) | 1181.19 (804.87 to 1711.3) | 16488455 (11392496 to 23578221) | 2238.06 (1546.36 to 3200.39) | 7937434 (5596157 to 11078000) | 2713.22 (1912.91 to 3786.75) | 6692702 (4519208 to 9546219) | 2818.96 (1903.49 to 4020.86) |
| 2013 | 4422184 (2986004 to 6447678) | 878.48 (593.18 to 1280.85) | 8861944 (6042226 to 12858454) | 1185.57 (808.34 to 1720.22) | 16379391 (11325221 to 23480022) | 2224.53 (1538.11 to 3188.89) | 7830542 (5521097 to 10984820) | 2683.75 (1892.24 to 3764.81) | 6678719 (4499210 to 9599760) | 2819.74 (1899.56 to 4053) |
| 2014 | 4545826 (3068811 to 6627112) | 883.47 (596.42 to 1287.97) | 8945822 (6094048 to 12975832) | 1190.05 (810.68 to 1726.16) | 16316864 (11284798 to 23411599) | 2214.98 (1531.89 to 3178.08) | 7774075 (5481321 to 10980081) | 2660.02 (1875.52 to 3757) | 6669220 (4482043 to 9662461) | 2819.65 (1894.94 to 4085.15) |
| 2015 | 4661223 (3147462 to 6795559) | 887.35 (599.18 to 1293.67) | 9013019 (6137051 to 13073083) | 1193.11 (812.4 to 1730.57) | 16294808 (11265046 to 23480184) | 2207.37 (1526.02 to 3180.74) | 7766206 (5476893 to 10988901) | 2643.24 (1864.07 to 3740.09) | 6663007 (4489339 to 9635160) | 2818.79 (1899.22 to 4076.16) |
| 2016 | 4768450 (3219978 to 6950276) | 890.35 (601.22 to 1297.73) | 9068199 (6169769 to 13147183) | 1195.43 (813.34 to 1733.15) | 16308920 (11270805 to 23490944) | 2200.86 (1520.98 to 3170.07) | 7791269 (5480940 to 11068846) | 2630.9 (1850.76 to 3737.65) | 6668467 (4481348 to 9661239) | 2822.54 (1896.81 to 4089.28) |
| 2017 | 4872447 (3290324 to 7100047) | 893.32 (603.25 to 1301.73) | 9121767 (6201713 to 13219227) | 1198.45 (814.8 to 1736.79) | 16383246 (11310002 to 23617439) | 2200.92 (1519.38 to 3172.76) | 7853675 (5507894 to 11130459) | 2629.12 (1843.84 to 3726.07) | 6686727 (4485753 to 9663047) | 2833.36 (1900.74 to 4094.52) |
| 2018 | 4975039 (3359792 to 7247408) | 896.09 (605.16 to 1305.39) | 9163140 (6226464 to 13259574) | 1201.15 (816.19 to 1738.13) | 16521946 (11396663 to 23790364) | 2210.47 (1524.76 to 3182.91) | 7961435 (5572331 to 11275668) | 2644.86 (1851.18 to 3745.88) | 6707198 (4502497 to 9737617) | 2847.52 (1911.52 to 4134.08) |
| 2019 | 5077535 (3429304 to 7394261) | 898.59 (606.89 to 1308.59) | 9189812 (6248466 to 13265068) | 1203.33 (818.19 to 1736.96) | 16694219 (11473250 to 23939320) | 2226.82 (1530.4 to 3193.23) | 8099608 (5644696 to 11420245) | 2676 (1864.93 to 3773.1) | 6717184 (4515641 to 9737908) | 2860.73 (1923.13 to 4147.2) |
| 2020 | 5178305 (3497685 to 7538404) | 900.93 (608.53 to 1311.54) | 9192600 (6255679 to 13246313) | 1203.04 (818.68 to 1733.55) | 16842084 (11605165 to 24143981) | 2244.51 (1546.6 to 3217.62) | 8253109 (5756945 to 11630084) | 2720.05 (1897.37 to 3833.03) | 6695019 (4517632 to 9500868) | 2863.96 (1932.53 to 4064.23) |
| 2021 | 5275743 (3563791 to 7677637) | 903.06 (610.02 to 1314.2) | 9259353 (6324715 to 13377414) | 1211.34 (827.42 to 1750.08) | 17093577 (11834476 to 24358086) | 2281.6 (1579.63 to 3251.25) | 8508482 (5888314 to 12082520) | 2804.72 (1941.02 to 3982.87) | 6705628 (4520672 to 9523753) | 2881.38 (1942.51 to 4092.32) |
| Percentage change from 1990 to 2021 (%) | 120.27 (114.81 to 125.13) | 5.41 (2.8 to 7.74) | 31.42 (27.39 to 35.86) | 1.61 (-1.5 to 5.05) | -1.56 (-5.83 to 3.29) | 0.46 (-3.9 to 5.41) | -9.12 (-13.48 to -4.21) | 10.9 (5.57 to 16.88) | 2.72 (-1.51 to 6.75) | 10.93 (6.36 to 15.28) |

Abbreviations: SDI, sociodemographic index; UI, uncertainty interval.

**eTable** **6b. Incidence of ADHD by SDI group and year, <20 years, 1990-2021.**

|  | **Prevalence** |  |  |  |  |  |  |  |  |  |
| --- | --- | --- | --- | --- | --- | --- | --- | --- | --- | --- |
|  | **Low SDI** |  | **Low-middle SDI** |  | **Middle SDI** |  | **High-middle SDI** |  | **High SDI** |  |
| **Year** | **Counts** | **Rate  (per 100,000)** | **Counts** | **Rate  (per 100,000)** | **Counts** | **Rate  (per 100,000)** | **Counts** | **Rate  (per 100,000)** | **Counts** | **Rate  (per 100,000)** |
| 1990 | 246568 (163588 to 366543) | 88.19 (58.51 to 131.11) | 673248 (448405 to 981867) | 113.91 (75.87 to 166.13) | 1472295 (1010854 to 2161335) | 192.57 (132.21 to 282.69) | 778782 (533085 to 1153261) | 210.39 (144.01 to 311.55) | 565607 (376876 to 830192) | 225.05 (149.96 to 330.33) |
| 1991 | 250761 (166146 to 372994) | 87.57 (58.02 to 130.25) | 674673 (449825 to 984670) | 112.59 (75.07 to 164.32) | 1521035 (1043165 to 2234594) | 198.2 (135.93 to 291.18) | 812354 (557955 to 1192939) | 220.1 (151.17 to 323.21) | 571275 (381051 to 838834) | 227.94 (152.04 to 334.7) |
| 1992 | 255388 (169263 to 380502) | 86.95 (57.63 to 129.54) | 675878 (451409 to 989911) | 111.27 (74.32 to 162.97) | 1567004 (1075762 to 2294164) | 203.71 (139.85 to 298.24) | 842848 (579291 to 1228207) | 229.43 (157.69 to 334.33) | 576975 (385211 to 845058) | 230.67 (154 to 337.85) |
| 1993 | 260646 (172907 to 388298) | 86.4 (57.31 to 128.71) | 677271 (453248 to 993140) | 110 (73.62 to 161.31) | 1600750 (1092582 to 2337924) | 207.86 (141.87 to 303.58) | 864227 (594551 to 1252519) | 236.65 (162.81 to 342.98) | 581603 (389480 to 849896) | 232.84 (155.92 to 340.25) |
| 1994 | 266021 (176867 to 397132) | 85.98 (57.16 to 128.36) | 679312 (455764 to 996861) | 108.86 (73.04 to 159.75) | 1616488 (1100242 to 2365520) | 209.75 (142.76 to 306.94) | 872353 (600466 to 1272409) | 240.5 (165.55 to 350.8) | 584726 (392886 to 849947) | 234.32 (157.44 to 340.6) |
| 1995 | 271741 (180775 to 405521) | 85.67 (56.99 to 127.84) | 682465 (459070 to 1004595) | 107.92 (72.59 to 158.86) | 1613418 (1095373 to 2372639) | 209.13 (141.98 to 307.54) | 866022 (595878 to 1262620) | 240.47 (165.46 to 350.6) | 585622 (394882 to 847899) | 234.88 (158.38 to 340.07) |
| 1996 | 278332 (185178 to 415340) | 85.4 (56.82 to 127.44) | 686370 (462791 to 1007668) | 107.14 (72.24 to 157.29) | 1598060 (1085064 to 2347269) | 206.85 (140.45 to 303.83) | 849381 (587480 to 1236559) | 237.6 (164.34 to 345.9) | 584777 (393286 to 847331) | 234.67 (157.83 to 340.04) |
| 1997 | 285472 (190182 to 425911) | 85.2 (56.76 to 127.12) | 690566 (466489 to 1012607) | 106.43 (71.89 to 156.06) | 1576905 (1068042 to 2312688) | 203.87 (138.08 to 298.99) | 827528 (573105 to 1201600) | 233.28 (161.56 to 338.73) | 582921 (391371 to 845449) | 234.07 (157.15 to 339.49) |
| 1998 | 292929 (195411 to 436907) | 85.09 (56.77 to 126.92) | 695186 (470598 to 1017290) | 105.83 (71.64 to 154.87) | 1549603 (1051400 to 2268682) | 200.2 (135.84 to 293.11) | 801405 (557031 to 1159907) | 227.79 (158.33 to 329.69) | 579672 (390084 to 841623) | 233.01 (156.8 to 338.31) |
| 1999 | 300651 (200822 to 447821) | 85.07 (56.82 to 126.72) | 700075 (473706 to 1026366) | 105.34 (71.28 to 154.44) | 1516464 (1027085 to 2220944) | 195.92 (132.69 to 286.93) | 772038 (536843 to 1113756) | 221.43 (153.98 to 319.44) | 575307 (386626 to 835173) | 231.74 (155.73 to 336.41) |
| 2000 | 308498 (206132 to 458658) | 85.1 (56.86 to 126.52) | 704916 (476614 to 1035361) | 104.91 (70.93 to 154.09) | 1480449 (1000884 to 2167933) | 191.34 (129.36 to 280.19) | 741903 (514434 to 1066759) | 214.83 (148.96 to 308.9) | 569898 (383456 to 826700) | 230.33 (154.98 to 334.12) |
| 2001 | 316690 (211450 to 471164) | 85.21 (56.89 to 126.77) | 710274 (480983 to 1039437) | 104.62 (70.85 to 153.11) | 1448417 (980526 to 2118366) | 187.27 (126.77 to 273.88) | 714820 (497932 to 1018664) | 208.9 (145.51 to 297.69) | 565337 (380983 to 819202) | 229.32 (154.54 to 332.29) |
| 2002 | 325865 (217226 to 485536) | 85.46 (56.97 to 127.33) | 716314 (483644 to 1050772) | 104.5 (70.56 to 153.29) | 1422093 (966875 to 2069091) | 184 (125.1 to 267.72) | 692039 (482919 to 980476) | 204.17 (142.48 to 289.27) | 562267 (377711 to 812918) | 228.92 (153.78 to 330.96) |
| 2003 | 335920 (223659 to 501269) | 85.77 (57.11 to 127.99) | 722165 (485720 to 1064655) | 104.39 (70.21 to 153.89) | 1400180 (951950 to 2032074) | 181.53 (123.42 to 263.46) | 673406 (469929 to 949798) | 200.95 (140.23 to 283.42) | 560046 (375012 to 806878) | 228.88 (153.26 to 329.75) |
| 2004 | 346055 (230502 to 516642) | 86.04 (57.31 to 128.46) | 727165 (487281 to 1074447) | 104.19 (69.82 to 153.96) | 1381881 (936870 to 2004272) | 179.81 (121.91 to 260.8) | 658725 (459199 to 926192) | 199.27 (138.91 to 280.18) | 558197 (373051 to 805438) | 228.92 (152.99 to 330.31) |
| 2005 | 355796 (237103 to 531391) | 86.2 (57.45 to 128.75) | 730902 (488710 to 1083854) | 103.86 (69.44 to 154.01) | 1366952 (922076 to 1983110) | 178.74 (120.57 to 259.3) | 648077 (450820 to 912503) | 199.22 (138.58 to 280.51) | 556419 (372626 to 805280) | 228.96 (153.33 to 331.37) |
| 2006 | 365390 (243477 to 545683) | 86.26 (57.48 to 128.83) | 733757 (490826 to 1087774) | 103.41 (69.17 to 153.31) | 1357114 (916459 to 1965302) | 178.5 (120.54 to 258.5) | 642388 (444829 to 903784) | 201.01 (139.19 to 282.81) | 555049 (370941 to 802558) | 229.06 (153.08 to 331.2) |
| 2007 | 374958 (249831 to 559932) | 86.27 (57.48 to 128.83) | 736544 (492891 to 1091755) | 102.97 (68.91 to 152.63) | 1353719 (915254 to 1959720) | 179.22 (121.17 to 259.45) | 642014 (442427 to 903751) | 204.53 (140.95 to 287.92) | 554632 (371302 to 802179) | 229.38 (153.56 to 331.76) |
| 2008 | 384486 (256155 to 574120) | 86.23 (57.45 to 128.77) | 739544 (495208 to 1096178) | 102.57 (68.69 to 152.04) | 1356845 (915309 to 1967609) | 180.73 (121.92 to 262.09) | 645961 (443836 to 912059) | 209.4 (143.88 to 295.67) | 555510 (373089 to 807542) | 230.33 (154.69 to 334.82) |
| 2009 | 394193 (262604 to 588634) | 86.18 (57.41 to 128.69) | 743017 (497991 to 1101246) | 102.25 (68.53 to 151.55) | 1365288 (919233 to 1985341) | 182.84 (123.1 to 265.87) | 653127 (447919 to 924360) | 215.3 (147.65 to 304.71) | 557291 (375429 to 817283) | 231.86 (156.19 to 340.02) |
| 2010 | 403630 (268858 to 602751) | 86.06 (57.33 to 128.52) | 747136 (501331 to 1107037) | 102.04 (68.47 to 151.19) | 1374968 (923191 to 1991924) | 185.18 (124.33 to 268.27) | 661610 (453158 to 939143) | 221.54 (151.74 to 314.47) | 559078 (377453 to 826247) | 233.7 (157.78 to 345.38) |
| 2011 | 413243 (274918 to 616598) | 86 (57.21 to 128.32) | 753160 (504831 to 1112420) | 102.1 (68.44 to 150.81) | 1383018 (930987 to 2014635) | 187.22 (126.03 to 272.73) | 669060 (458893 to 953384) | 226.91 (155.63 to 323.33) | 561174 (377439 to 829860) | 235.56 (158.44 to 348.35) |
| 2012 | 423455 (281492 to 630890) | 86.07 (57.21 to 128.23) | 761284 (510321 to 1123958) | 102.49 (68.71 to 151.32) | 1388958 (936544 to 2022339) | 188.53 (127.12 to 274.5) | 673723 (462576 to 962910) | 230.3 (158.12 to 329.15) | 563502 (377378 to 833797) | 237.35 (158.95 to 351.19) |
| 2013 | 433562 (288610 to 645340) | 86.13 (57.33 to 128.2) | 770057 (517704 to 1136504) | 103.02 (69.26 to 152.04) | 1393950 (943291 to 2029394) | 189.32 (128.11 to 275.62) | 677200 (465278 to 971264) | 232.1 (159.46 to 332.88) | 565652 (377305 to 835819) | 238.82 (159.3 to 352.88) |
| 2014 | 443132 (295383 to 659104) | 86.12 (57.41 to 128.1) | 778168 (523510 to 1148245) | 103.52 (69.64 to 152.75) | 1402840 (951830 to 2048066) | 190.43 (129.21 to 278.02) | 684045 (470437 to 984742) | 234.06 (160.97 to 336.94) | 567581 (377132 to 838490) | 239.97 (159.45 to 354.5) |
| 2015 | 451633 (301301 to 671377) | 85.98 (57.36 to 127.81) | 784540 (526830 to 1157582) | 103.85 (69.74 to 153.24) | 1416317 (958880 to 2069542) | 191.86 (129.89 to 280.35) | 695223 (478692 to 1004221) | 236.62 (162.92 to 341.79) | 569375 (377514 to 840697) | 240.87 (159.71 to 355.66) |
| 2016 | 459123 (306253 to 682605) | 85.73 (57.18 to 127.45) | 789486 (531193 to 1161195) | 104.08 (70.03 to 153.08) | 1430412 (970159 to 2093930) | 193.03 (130.92 to 282.57) | 707362 (486128 to 1022326) | 238.86 (164.15 to 345.21) | 570791 (379970 to 839992) | 241.6 (160.83 to 355.54) |
| 2017 | 466089 (310860 to 693050) | 85.45 (56.99 to 127.06) | 793749 (535013 to 1163218) | 104.29 (70.29 to 152.83) | 1445874 (981990 to 2114455) | 194.24 (131.92 to 284.06) | 720101 (493683 to 1039365) | 241.06 (165.27 to 347.94) | 571649 (382486 to 839877) | 242.22 (162.07 to 355.88) |
| 2018 | 472764 (315276 to 703063) | 85.15 (56.79 to 126.63) | 796087 (536993 to 1162267) | 104.35 (70.39 to 152.36) | 1463709 (992663 to 2141221) | 195.83 (132.81 to 286.47) | 734703 (504378 to 1066184) | 244.07 (167.56 to 354.2) | 571882 (383836 to 840594) | 242.79 (162.96 to 356.87) |
| 2019 | 479332 (319621 to 712932) | 84.83 (56.56 to 126.17) | 795685 (535832 to 1157780) | 104.19 (70.16 to 151.6) | 1479735 (1001791 to 2172473) | 197.38 (133.63 to 289.78) | 748476 (512748 to 1087027) | 247.29 (169.41 to 359.14) | 571040 (382082 to 845336) | 243.2 (162.72 to 360.01) |
| 2020 | 485789 (323896 to 722633) | 84.52 (56.35 to 125.72) | 791473 (532695 to 1153069) | 103.58 (69.71 to 150.9) | 1489259 (1006775 to 2183715) | 198.47 (134.17 to 291.02) | 760290 (521371 to 1103317) | 250.58 (171.83 to 363.63) | 566868 (376561 to 830313) | 242.49 (161.08 to 355.19) |
| 2021 | 492088 (328072 to 732081) | 84.23 (56.16 to 125.31) | 791382 (534709 to 1160430) | 103.53 (69.95 to 151.81) | 1490813 (1011900 to 2163491) | 198.99 (135.07 to 288.78) | 769277 (524059 to 1111163) | 253.58 (172.75 to 366.28) | 563965 (375078 to 827459) | 242.33 (161.17 to 355.56) |
| Percentage change from 1990 to 2021 (%) | 99.58 (94.92 to 103.88) | -4.49 (-6.72 to -2.43) | 17.55 (13.99 to 21.22) | -9.11 (-11.86 to -6.27) | 1.26 (-3.55 to 6.32) | 3.33 (-1.57 to 8.51) | -1.22 (-6.07 to 4.37) | 20.53 (14.61 to 27.36) | -0.29 (-4.3 to 3.6) | 7.68 (3.34 to 11.88) |

Abbreviations: SDI, sociodemographic index; UI, uncertainty interval.

**eTable** **6c. DALYs caused by ADHD by SDI group and year, <20 years, 1990-2021.**

|  | **Prevalence** |  |  |  |  |  |  |  |  |  |
| --- | --- | --- | --- | --- | --- | --- | --- | --- | --- | --- |
|  | **Low SDI** |  | **Low-middle SDI** |  | **Middle SDI** |  | **High-middle SDI** |  | **High SDI** |  |
| **Year** | **Counts** | **Rate  (per 100,000)** | **Counts** | **Rate  (per 100,000)** | **Counts** | **Rate  (per 100,000)** | **Counts** | **Rate  (per 100,000)** | **Counts** | **Rate  (per 100,000)** |
| 1990 | 29070 (14898 to 48806) | 10.4 (5.33 to 17.46) | 85913 (44167 to 143648) | 14.54 (7.47 to 24.3) | 212818 (109630 to 357486) | 27.84 (14.34 to 46.76) | 115001 (60065 to 194757) | 31.07 (16.23 to 52.61) | 80054 (41600 to 134665) | 31.85 (16.55 to 53.58) |
| 1991 | 29703 (15321 to 49807) | 10.37 (5.35 to 17.39) | 86672 (44356 to 144946) | 14.46 (7.4 to 24.19) | 216432 (110612 to 364005) | 28.2 (14.41 to 47.43) | 118000 (61830 to 200464) | 31.97 (16.75 to 54.31) | 80564 (41890 to 134524) | 32.15 (16.71 to 53.68) |
| 1992 | 30399 (15511 to 51101) | 10.35 (5.28 to 17.4) | 87341 (44581 to 145240) | 14.38 (7.34 to 23.91) | 219471 (111344 to 369686) | 28.53 (14.47 to 48.06) | 120763 (62959 to 205817) | 32.87 (17.14 to 56.03) | 81126 (42442 to 135866) | 32.43 (16.97 to 54.32) |
| 1993 | 31161 (15876 to 52455) | 10.33 (5.26 to 17.39) | 88212 (44995 to 146784) | 14.33 (7.31 to 23.84) | 222138 (112488 to 373692) | 28.85 (14.61 to 48.52) | 123231 (64227 to 209985) | 33.74 (17.59 to 57.5) | 81782 (42652 to 138191) | 32.74 (17.08 to 55.32) |
| 1994 | 31946 (16176 to 54086) | 10.33 (5.23 to 17.48) | 89201 (45169 to 148978) | 14.29 (7.24 to 23.87) | 224326 (113493 to 377510) | 29.11 (14.73 to 48.98) | 125152 (64351 to 214039) | 34.5 (17.74 to 59.01) | 82452 (43035 to 139917) | 33.04 (17.25 to 56.07) |
| 1995 | 32730 (16670 to 55294) | 10.32 (5.26 to 17.43) | 90350 (45707 to 149851) | 14.29 (7.23 to 23.7) | 226216 (114403 to 379787) | 29.32 (14.83 to 49.23) | 126497 (65045 to 218046) | 35.13 (18.06 to 60.55) | 83025 (43491 to 141029) | 33.3 (17.44 to 56.56) |
| 1996 | 33606 (17264 to 56190) | 10.31 (5.3 to 17.24) | 91586 (46861 to 152634) | 14.3 (7.31 to 23.83) | 227937 (114135 to 382639) | 29.5 (14.77 to 49.53) | 127288 (66534 to 216434) | 35.61 (18.61 to 60.54) | 83535 (43724 to 142648) | 33.52 (17.55 to 57.24) |
| 1997 | 34522 (17798 to 58060) | 10.3 (5.31 to 17.33) | 92824 (46646 to 155245) | 14.31 (7.19 to 23.93) | 230038 (116047 to 386825) | 29.74 (15 to 50.01) | 127812 (66618 to 214794) | 36.03 (18.78 to 60.55) | 83974 (43944 to 142189) | 33.72 (17.65 to 57.1) |
| 1998 | 35441 (18149 to 60234) | 10.3 (5.27 to 17.5) | 94017 (47637 to 158458) | 14.31 (7.25 to 24.12) | 231835 (117384 to 390360) | 29.95 (15.17 to 50.43) | 127874 (67532 to 213277) | 36.35 (19.19 to 60.62) | 84295 (44263 to 142634) | 33.88 (17.79 to 57.33) |
| 1999 | 36400 (18671 to 61729) | 10.3 (5.28 to 17.47) | 95217 (48460 to 160647) | 14.33 (7.29 to 24.17) | 232947 (117976 to 388007) | 30.1 (15.24 to 50.13) | 127442 (67712 to 209907) | 36.55 (19.42 to 60.2) | 84482 (44345 to 143567) | 34.03 (17.86 to 57.83) |
| 2000 | 37402 (19081 to 63600) | 10.32 (5.26 to 17.54) | 96386 (49201 to 162451) | 14.34 (7.32 to 24.18) | 233128 (119232 to 390536) | 30.13 (15.41 to 50.47) | 126439 (66935 to 209663) | 36.61 (19.38 to 60.71) | 84476 (43896 to 142850) | 34.14 (17.74 to 57.73) |
| 2001 | 38387 (19794 to 65610) | 10.33 (5.33 to 17.65) | 97521 (50153 to 165200) | 14.36 (7.39 to 24.33) | 232502 (118703 to 388835) | 30.06 (15.35 to 50.27) | 124918 (66839 to 204127) | 36.51 (19.53 to 59.65) | 84480 (44258 to 142619) | 34.27 (17.95 to 57.85) |
| 2002 | 39483 (20533 to 67233) | 10.35 (5.38 to 17.63) | 98768 (50241 to 167820) | 14.41 (7.33 to 24.48) | 231221 (118168 to 387300) | 29.92 (15.29 to 50.11) | 122939 (64868 to 201484) | 36.27 (19.14 to 59.44) | 84544 (44238 to 143141) | 34.42 (18.01 to 58.28) |
| 2003 | 40699 (21061 to 69068) | 10.39 (5.38 to 17.63) | 99989 (50858 to 170251) | 14.45 (7.35 to 24.61) | 228987 (117652 to 383106) | 29.69 (15.25 to 49.67) | 120383 (63571 to 196717) | 35.92 (18.97 to 58.7) | 84562 (43953 to 141650) | 34.56 (17.96 to 57.89) |
| 2004 | 41933 (21410 to 71493) | 10.43 (5.32 to 17.78) | 101196 (51843 to 171692) | 14.5 (7.43 to 24.6) | 226158 (116453 to 379470) | 29.43 (15.15 to 49.38) | 117536 (62450 to 193061) | 35.56 (18.89 to 58.4) | 84506 (43559 to 140888) | 34.66 (17.86 to 57.78) |
| 2005 | 43103 (21960 to 73248) | 10.44 (5.32 to 17.75) | 102019 (52369 to 172674) | 14.5 (7.44 to 24.54) | 222965 (113953 to 372594) | 29.15 (14.9 to 48.72) | 114560 (60395 to 185992) | 35.22 (18.57 to 57.17) | 84343 (43406 to 141142) | 34.71 (17.86 to 58.08) |
| 2006 | 44298 (22787 to 75530) | 10.46 (5.38 to 17.83) | 102879 (52484 to 174395) | 14.5 (7.4 to 24.58) | 219740 (113153 to 368324) | 28.9 (14.88 to 48.45) | 111856 (58968 to 181217) | 35 (18.45 to 56.71) | 84055 (43082 to 139906) | 34.69 (17.78 to 57.74) |
| 2007 | 45511 (23535 to 77526) | 10.47 (5.41 to 17.84) | 103515 (53066 to 174558) | 14.47 (7.42 to 24.4) | 216564 (112097 to 363743) | 28.67 (14.84 to 48.16) | 109224 (58000 to 177766) | 34.8 (18.48 to 56.63) | 83753 (42847 to 140352) | 34.64 (17.72 to 58.05) |
| 2008 | 46837 (23934 to 80134) | 10.5 (5.37 to 17.97) | 104177 (52779 to 176197) | 14.45 (7.32 to 24.44) | 213417 (110413 to 358493) | 28.43 (14.71 to 47.75) | 106622 (57178 to 174989) | 34.56 (18.54 to 56.73) | 83471 (42993 to 140020) | 34.61 (17.83 to 58.05) |
| 2009 | 48095 (24634 to 81880) | 10.51 (5.39 to 17.9) | 104833 (53722 to 177099) | 14.43 (7.39 to 24.37) | 210370 (109620 to 351884) | 28.17 (14.68 to 47.12) | 104025 (55265 to 169668) | 34.29 (18.22 to 55.93) | 83118 (42691 to 139676) | 34.58 (17.76 to 58.11) |
| 2010 | 49450 (25358 to 84618) | 10.54 (5.41 to 18.04) | 105586 (53885 to 179459) | 14.42 (7.36 to 24.51) | 207163 (108741 to 347275) | 27.9 (14.64 to 46.77) | 101540 (54494 to 167090) | 34 (18.25 to 55.95) | 82694 (42389 to 139790) | 34.57 (17.72 to 58.43) |
| 2011 | 50830 (26065 to 86357) | 10.58 (5.42 to 17.97) | 106234 (54423 to 179779) | 14.4 (7.38 to 24.37) | 204381 (105919 to 341258) | 27.67 (14.34 to 46.2) | 99360 (53404 to 163254) | 33.7 (18.11 to 55.37) | 82368 (42241 to 139230) | 34.58 (17.73 to 58.44) |
| 2012 | 52375 (26660 to 88526) | 10.65 (5.42 to 17.99) | 107280 (54687 to 181188) | 14.44 (7.36 to 24.39) | 202430 (103980 to 338247) | 27.48 (14.11 to 45.91) | 97539 (51806 to 162166) | 33.34 (17.71 to 55.43) | 82083 (42461 to 138913) | 34.57 (17.88 to 58.51) |
| 2013 | 53874 (27660 to 90366) | 10.7 (5.49 to 17.95) | 108395 (55674 to 184132) | 14.5 (7.45 to 24.63) | 201159 (103285 to 338643) | 27.32 (14.03 to 45.99) | 96278 (51054 to 159554) | 33 (17.5 to 54.68) | 81893 (42588 to 137431) | 34.57 (17.98 to 58.02) |
| 2014 | 55377 (28355 to 93676) | 10.76 (5.51 to 18.21) | 109391 (55712 to 183775) | 14.55 (7.41 to 24.45) | 200339 (102188 to 335501) | 27.2 (13.87 to 45.54) | 95563 (50216 to 158749) | 32.7 (17.18 to 54.32) | 81811 (42468 to 137578) | 34.59 (17.96 to 58.17) |
| 2015 | 56782 (28906 to 97180) | 10.81 (5.5 to 18.5) | 110175 (56279 to 184704) | 14.58 (7.45 to 24.45) | 200079 (101550 to 335946) | 27.1 (13.76 to 45.51) | 95508 (49419 to 158993) | 32.51 (16.82 to 54.11) | 81703 (42369 to 137714) | 34.56 (17.92 to 58.26) |
| 2016 | 58170 (29803 to 98277) | 10.86 (5.56 to 18.35) | 111010 (56490 to 186074) | 14.63 (7.45 to 24.53) | 200253 (101636 to 340601) | 27.02 (13.72 to 45.96) | 95871 (49702 to 161252) | 32.37 (16.78 to 54.45) | 81778 (42404 to 137461) | 34.61 (17.95 to 58.18) |
| 2017 | 59406 (30249 to 101385) | 10.89 (5.55 to 18.59) | 111694 (56615 to 187651) | 14.67 (7.44 to 24.65) | 201195 (102383 to 341270) | 27.03 (13.75 to 45.85) | 96584 (49868 to 162830) | 32.33 (16.69 to 54.51) | 82023 (41876 to 138795) | 34.76 (17.74 to 58.81) |
| 2018 | 60690 (30941 to 101925) | 10.93 (5.57 to 18.36) | 112114 (57301 to 187903) | 14.7 (7.51 to 24.63) | 202972 (103042 to 343869) | 27.16 (13.79 to 46.01) | 97943 (50265 to 164625) | 32.54 (16.7 to 54.69) | 82263 (41917 to 139297) | 34.92 (17.8 to 59.14) |
| 2019 | 61980 (31878 to 104597) | 10.97 (5.64 to 18.51) | 112478 (57501 to 187605) | 14.73 (7.53 to 24.57) | 205104 (104076 to 346378) | 27.36 (13.88 to 46.2) | 99656 (51944 to 167957) | 32.92 (17.16 to 55.49) | 82424 (41789 to 139431) | 35.1 (17.8 to 59.38) |
| 2020 | 63131 (32311 to 106461) | 10.98 (5.62 to 18.52) | 112473 (57143 to 188870) | 14.72 (7.48 to 24.72) | 206909 (104682 to 348541) | 27.57 (13.95 to 46.45) | 101508 (52458 to 169506) | 33.45 (17.29 to 55.87) | 82067 (41998 to 140529) | 35.11 (17.97 to 60.11) |
| 2021 | 64320 (32848 to 109173) | 11.01 (5.62 to 18.69) | 113294 (57393 to 189909) | 14.82 (7.51 to 24.84) | 209876 (107401 to 358756) | 28.01 (14.34 to 47.89) | 104671 (55270 to 178232) | 34.5 (18.22 to 58.75) | 82231 (41941 to 140773) | 35.33 (18.02 to 60.49) |
| Percentage change from 1990 to 2021 (%) | 121.26 (113.21 to 128.8) | 5.88 (2.04 to 9.5) | 31.87 (27.03 to 36.96) | 1.96 (-1.78 to 5.9) | -1.38 (-6.05 to 3.15) | 0.64 (-4.12 to 5.27) | -8.98 (-13.27 to -3.21) | 11.06 (5.83 to 18.1) | 2.72 (-1.65 to 6.86) | 10.93 (6.21 to 15.4) |

Abbreviations: SDI, sociodemographic index; DALYs, disability-adjusted life-years; UI, uncertainty interval.

**eTable** **7a. Prevalence of ADHD by region and sex, <20 years, 1990-2021.**

|  | **Prevalence** | | | | | | | |
| --- | --- | --- | --- | --- | --- | --- | --- | --- |
|  | **Male** | | | | **Female** | | | |
|  | **Counts (<20 years)** | | **Rates per 100,000 persons**  **(<20 years)** | | **Counts (<20 years)** | | **Rates per 100,000 persons**  **(<20 years)** | |
| **Regions** | **1990 (95% UI)** | **2021 (95% UI)** | **1990 (95% UI)** | **2021 (95% UI)** | **1990 (95% UI)** | **2021 (95% UI)** | **1990 (95% UI)** | **2021 (95% UI)** |
| Global | 31014307 (21296207 to 44378155) | 34195478 (23419401 to 49088957) | 2679.71 (1840.04 to 3834.38) | 2517.16 (1723.92 to 3613.48) | 11728453 (7994548 to 16938146) | 12695256 (8675052 to 18339791) | 1065.06 (725.99 to 1538.16) | 993.88 (679.15 to 1435.77) |
| High-income Asia Pacific | 924348 (625484 to 1374908) | 555657 (378516 to 812263) | 3572.22 (2417.24 to 5313.45) | 3514.4 (2394.03 to 5137.38) | 273120 (180736 to 400682) | 164048 (112330 to 237445) | 1117.02 (739.18 to 1638.73) | 1095.25 (749.95 to 1585.27) |
| High-income North America | 2025601 (1356510 to 2919824) | 2480446 (1630568 to 3583127) | 4836.6 (3238.99 to 6971.77) | 5416.69 (3560.76 to 7824.68) | 600652 (414145 to 877428) | 767939 (513275 to 1136376) | 1507.31 (1039.28 to 2201.87) | 1754.67 (1172.79 to 2596.52) |
| Western Europe | 1750483 (1224344 to 2454135) | 1713595 (1150560 to 2420739) | 3473.84 (2429.72 to 4870.24) | 3639.56 (2443.71 to 5141.49) | 627648 (442681 to 885150) | 607388 (405315 to 873820) | 1308.82 (923.11 to 1845.79) | 1360.99 (908.2 to 1957.99) |
| Australasia | 237527 (163215 to 339486) | 298908 (219716 to 393103) | 7394.48 (5081.07 to 10568.58) | 7706.76 (5664.97 to 10135.42) | 84665 (60192 to 120469) | 105791 (73905 to 145346) | 2765.98 (1966.46 to 3935.68) | 2887.89 (2017.46 to 3967.67) |
| Andean Latin America | 431230 (289717 to 634389) | 567089 (381143 to 833101) | 4508.81 (3029.19 to 6632.97) | 4642.58 (3120.3 to 6820.34) | 191153 (130411 to 277374) | 237685 (162192 to 344689) | 2035.29 (1388.55 to 2953.32) | 2074.29 (1415.46 to 3008.12) |
| Tropical Latin America | 1586979 (1084826 to 2298584) | 1529325 (1033420 to 2240244) | 4544.59 (3106.59 to 6582.4) | 4502.38 (3042.42 to 6595.35) | 747750 (500199 to 1132228) | 697909 (479657 to 1057294) | 2177.39 (1456.54 to 3296.96) | 2139.48 (1470.42 to 3241.2) |
| Central Latin America | 1252787 (854780 to 1793083) | 1378744 (950200 to 1976220) | 3014.12 (2056.54 to 4314.04) | 3184.96 (2195 to 4565.15) | 588697 (396523 to 851487) | 636288 (433245 to 911717) | 1433.49 (965.54 to 2073.39) | 1515.12 (1031.64 to 2170.97) |
| Southern Latin America | 303647 (202997 to 431250) | 324622 (217552 to 473408) | 3103.63 (2074.87 to 4407.89) | 3264.94 (2188.07 to 4761.38) | 113025 (75027 to 165105) | 117865 (78807 to 172931) | 1177.87 (781.89 to 1720.61) | 1232 (823.74 to 1807.58) |
| Caribbean | 503962 (354367 to 718227) | 531272 (368334 to 771693) | 6643.22 (4671.27 to 9467.67) | 6850.42 (4749.43 to 9950.49) | 195043 (133519 to 285113) | 199743 (135506 to 294908) | 2596.11 (1777.2 to 3794.99) | 2660.67 (1805.01 to 3928.32) |
| Central Europe | 465673 (315300 to 680480) | 280606 (189854 to 409920) | 2323.91 (1573.48 to 3395.89) | 2313.04 (1564.97 to 3378.98) | 192456 (130121 to 278884) | 113893 (76959 to 165022) | 1000.81 (676.66 to 1450.25) | 996.81 (673.56 to 1444.3) |
| Eastern Europe | 785594 (523761 to 1162840) | 569707 (379647 to 842858) | 2292.97 (1528.74 to 3394.06) | 2404.09 (1602.06 to 3556.76) | 325544 (216999 to 475133) | 231865 (154085 to 337968) | 986.07 (657.29 to 1439.17) | 1032.24 (685.97 to 1504.6) |
| Central Asia | 334382 (224663 to 496368) | 379795 (255297 to 564734) | 2085.33 (1401.08 to 3095.53) | 2120.37 (1425.3 to 3152.86) | 140608 (95608 to 207330) | 153706 (104425 to 226944) | 904.53 (615.04 to 1333.75) | 919.69 (624.82 to 1357.9) |
| North Africa and Middle East | 2481474 (1709572 to 3620920) | 3178734 (2180219 to 4607943) | 2739.35 (1887.23 to 3997.21) | 2606.36 (1787.64 to 3778.23) | 972085 (669208 to 1392778) | 1215377 (830278 to 1757567) | 1127.89 (776.47 to 1616.01) | 1061.18 (724.94 to 1534.58) |
| South Asia | 3391751 (2234247 to 4997177) | 4610000 (3067158 to 6673358) | 1205 (793.77 to 1775.36) | 1296.92 (862.87 to 1877.4) | 1338611 (887536 to 1972963) | 1796754 (1198585 to 2657453) | 512.97 (340.11 to 756.06) | 547.73 (365.38 to 810.11) |
| Southeast Asia | 2416004 (1664227 to 3427477) | 2437837 (1676058 to 3506693) | 2162.16 (1489.37 to 3067.36) | 2068.85 (1422.37 to 2975.93) | 984821 (654284 to 1447134) | 989877 (657351 to 1480397) | 910.52 (604.92 to 1337.95) | 888.29 (589.89 to 1328.47) |
| East Asia | 10587761 (7295427 to 15238646) | 9773988 (6833817 to 14032753) | 4431.35 (3053.39 to 6377.91) | 5308.32 (3711.49 to 7621.29) | 3728450 (2576578 to 5350365) | 3244265 (2247289 to 4650809) | 1685.47 (1164.76 to 2418.67) | 2017.25 (1397.34 to 2891.83) |
| Oceania | 41417 (28248 to 61626) | 78485 (53542 to 116754) | 2361.1 (1610.32 to 3513.16) | 2346.95 (1601.06 to 3491.3) | 15770 (10526 to 22998) | 29551 (19723 to 43097) | 978.16 (652.86 to 1426.48) | 971.36 (648.29 to 1416.61) |
| Western Sub-Saharan Africa | 584683 (389089 to 869765) | 1598754 (1046102 to 2339537) | 1102.65 (733.78 to 1640.28) | 1190.97 (779.28 to 1742.81) | 238316 (158057 to 356921) | 616317 (408287 to 920223) | 437.53 (290.18 to 655.28) | 458.79 (303.93 to 685.01) |
| Eastern Sub-Saharan Africa | 597200 (400084 to 876156) | 1306614 (875890 to 1913120) | 1066.23 (714.3 to 1564.27) | 1134.86 (760.76 to 1661.64) | 241131 (160205 to 365262) | 525842 (349561 to 797360) | 439.32 (291.88 to 665.48) | 467.62 (310.86 to 709.08) |
| Central Sub-Saharan Africa | 163069 (109091 to 241858) | 415871 (278302 to 617162) | 1044.97 (699.06 to 1549.86) | 1119.08 (748.89 to 1660.75) | 66476 (43918 to 100290) | 168325 (111356 to 253852) | 432.14 (285.5 to 651.95) | 462.44 (305.93 to 697.41) |
| Southern Sub-Saharan Africa | 148735 (99724 to 217840) | 185427 (124350 to 272090) | 1131.9 (758.92 to 1657.8) | 1176.42 (788.93 to 1726.24) | 62431 (41288 to 92204) | 74827 (49509 to 110377) | 468.69 (309.96 to 692.2) | 482.68 (319.36 to 711.99) |

Abbreviations: UI, uncertainty interval.

**eTable** **7b. Incidence of ADHD by region and sex, <20 years, 1990-2021.**

|  | **Prevalence** | | | | | | | |
| --- | --- | --- | --- | --- | --- | --- | --- | --- |
|  | **Male** | | | | **Female** | | | |
|  | **Counts (<20 years)** | | **Rates per 100,000 persons**  **(<20 years)** | | **Counts (<20 years)** | | **Rates per 100,000 persons**  **(<20 years)** | |
| **Regions** | **1990 (95% UI)** | **2021 (95% UI)** | **1990 (95% UI)** | **2021 (95% UI)** | **1990 (95% UI)** | **2021 (95% UI)** | **1990 (95% UI)** | **2021 (95% UI)** |
| Global | 2729949 (1865881 to 4010135) | 3010780 (2038577 to 4353004) | 235.87 (161.22 to 346.48) | 221.63 (150.06 to 320.43) | 1010625 (683305 to 1474091) | 1100841 (737475 to 1605962) | 91.77 (62.05 to 133.86) | 86.18 (57.73 to 125.73) |
| High-income Asia Pacific | 68328 (45335 to 102989) | 43644 (29055 to 64287) | 264.06 (175.2 to 398.01) | 276.04 (183.77 to 406.6) | 19872 (12988 to 29437) | 12826 (8591 to 19113) | 81.27 (53.12 to 120.39) | 85.63 (57.36 to 127.61) |
| High-income North America | 183611 (122289 to 273323) | 200221 (131112 to 299097) | 438.41 (291.99 to 652.62) | 437.23 (286.32 to 653.16) | 52969 (34997 to 78199) | 60658 (40022 to 91011) | 132.92 (87.82 to 196.24) | 138.6 (91.45 to 207.95) |
| Western Europe | 148292 (100273 to 217165) | 151226 (100864 to 222855) | 294.29 (198.99 to 430.96) | 321.19 (214.23 to 473.33) | 51499 (35405 to 75729) | 51997 (33924 to 77440) | 107.39 (73.83 to 157.92) | 116.51 (76.01 to 173.52) |
| Australasia | 19735 (13423 to 28588) | 25725 (18038 to 34340) | 614.36 (417.88 to 889.98) | 663.26 (465.07 to 885.39) | 6943 (4865 to 10250) | 9042 (6151 to 13148) | 226.83 (158.93 to 334.86) | 246.84 (167.91 to 358.9) |
| Andean Latin America | 38949 (26077 to 58319) | 48358 (32381 to 72394) | 407.24 (272.65 to 609.77) | 395.9 (265.09 to 592.67) | 17204 (11309 to 25902) | 20728 (13626 to 31207) | 183.18 (120.41 to 275.79) | 180.9 (118.92 to 272.35) |
| Tropical Latin America | 135635 (91118 to 201457) | 126738 (84681 to 189232) | 388.41 (260.93 to 576.91) | 373.12 (249.3 to 557.1) | 63363 (41738 to 96585) | 58198 (39465 to 88067) | 184.51 (121.54 to 281.25) | 178.41 (120.98 to 269.97) |
| Central Latin America | 113126 (76800 to 165827) | 111574 (76783 to 162604) | 272.17 (184.78 to 398.97) | 257.74 (177.37 to 375.62) | 52914 (34785 to 77455) | 51993 (34828 to 77339) | 128.85 (84.7 to 188.61) | 123.81 (82.93 to 184.16) |
| Southern Latin America | 28284 (18588 to 42201) | 27951 (18426 to 41394) | 289.1 (189.99 to 431.35) | 281.13 (185.32 to 416.33) | 10285 (6765 to 15286) | 9984 (6595 to 14816) | 107.18 (70.5 to 159.3) | 104.36 (68.94 to 154.86) |
| Caribbean | 43018 (29702 to 63889) | 44072 (29932 to 66677) | 567.07 (391.53 to 842.18) | 568.28 (385.96 to 859.76) | 16718 (11121 to 24984) | 16786 (11105 to 25395) | 222.52 (148.03 to 332.54) | 223.6 (147.93 to 338.27) |
| Central Europe | 37071 (25332 to 54663) | 22398 (15309 to 33051) | 185 (126.42 to 272.79) | 184.62 (126.19 to 272.44) | 15462 (10294 to 22772) | 9149 (6091 to 13474) | 80.41 (53.53 to 118.42) | 80.07 (53.31 to 117.93) |
| Eastern Europe | 68308 (45541 to 102083) | 47232 (31486 to 70638) | 199.38 (132.92 to 297.96) | 199.31 (132.87 to 298.08) | 28380 (18525 to 42584) | 19281 (12590 to 29013) | 85.96 (56.11 to 128.99) | 85.84 (56.05 to 129.16) |
| Central Asia | 32223 (21700 to 48470) | 36572 (24633 to 55001) | 200.96 (135.33 to 302.28) | 204.18 (137.52 to 307.06) | 13592 (8963 to 20294) | 14794 (9757 to 22087) | 87.43 (57.66 to 130.55) | 88.52 (58.38 to 132.15) |
| North Africa and Middle East | 238159 (163676 to 357339) | 283780 (193619 to 419845) | 262.91 (180.69 to 394.47) | 232.68 (158.76 to 344.25) | 92944 (63029 to 135071) | 108489 (73096 to 159639) | 107.84 (73.13 to 156.72) | 94.72 (63.82 to 139.39) |
| South Asia | 329352 (216210 to 493896) | 368679 (241032 to 542281) | 117.01 (76.81 to 175.47) | 103.72 (67.81 to 152.56) | 129415 (84416 to 195835) | 142571 (93237 to 212558) | 49.59 (32.35 to 75.05) | 43.46 (28.42 to 64.8) |
| Southeast Asia | 216034 (150333 to 311751) | 204610 (136156 to 302243) | 193.34 (134.54 to 279) | 173.64 (115.55 to 256.5) | 86166 (57360 to 128994) | 82198 (54564 to 124029) | 79.66 (53.03 to 119.26) | 73.76 (48.96 to 111.3) |
| East Asia | 872265 (594488 to 1309110) | 925649 (630321 to 1351831) | 365.07 (248.81 to 547.91) | 502.73 (342.33 to 734.19) | 290108 (200940 to 429196) | 299665 (203157 to 440244) | 131.15 (90.84 to 194.02) | 186.33 (126.32 to 273.74) |
| Oceania | 4068 (2735 to 6127) | 7722 (5191 to 11630) | 231.93 (155.9 to 349.27) | 230.92 (155.22 to 347.78) | 1527 (1001 to 2287) | 2892 (1896 to 4332) | 94.71 (62.08 to 141.86) | 95.07 (62.32 to 142.4) |
| Western Sub-Saharan Africa | 61418 (40413 to 92173) | 157126 (103264 to 232247) | 115.83 (76.21 to 173.83) | 117.05 (76.93 to 173.01) | 24254 (15930 to 37397) | 58638 (38519 to 89878) | 44.53 (29.25 to 68.66) | 43.65 (28.67 to 66.9) |
| Eastern Sub-Saharan Africa | 61205 (40418 to 89757) | 121003 (79914 to 177476) | 109.27 (72.16 to 160.25) | 105.1 (69.41 to 154.15) | 24396 (16005 to 37602) | 48210 (31612 to 73944) | 44.45 (29.16 to 68.51) | 42.87 (28.11 to 65.76) |
| Central Sub-Saharan Africa | 17072 (11228 to 25334) | 40351 (26542 to 59868) | 109.4 (71.95 to 162.34) | 108.58 (71.42 to 161.1) | 6954 (4515 to 10699) | 16238 (10542 to 24981) | 45.21 (29.35 to 69.55) | 44.61 (28.96 to 68.63) |
| Southern Sub-Saharan Africa | 13795 (9018 to 20519) | 16146 (10575 to 23958) | 104.98 (68.63 to 156.16) | 102.44 (67.09 to 152) | 5661 (3702 to 8663) | 6505 (4257 to 9949) | 42.5 (27.79 to 65.03) | 41.96 (27.46 to 64.18) |

Abbreviations: UI, uncertainty interval.

**eTable** **7c. DALYs caused by ADHD by region and sex, <20 years, 1990-2021.**

|  | **Prevalence** | | | | | | | |
| --- | --- | --- | --- | --- | --- | --- | --- | --- |
|  | **Male** | | | | **Female** | | | |
|  | **Counts (<20 years)** | | **Rates per 100,000 persons**  **(<20 years)** | | **Counts (<20 years)** | | **Rates per 100,000 persons**  **(<20 years)** | |
| **Regions** | **1990 (95% UI)** | **2021 (95% UI)** | **1990 (95% UI)** | **2021 (95% UI)** | **1990 (95% UI)** | **2021 (95% UI)** | **1990 (95% UI)** | **2021 (95% UI)** |
| Global | 380131 (198559 to 636722) | 419703 (212849 to 716123) | 32.84 (17.16 to 55.01) | 30.89 (15.67 to 52.71) | 143304 (73700 to 240376) | 155276 (81352 to 262329) | 13.01 (6.69 to 21.83) | 12.16 (6.37 to 20.54) |
| High-income Asia Pacific | 11341 (5661 to 19359) | 6830 (3456 to 11629) | 43.83 (21.88 to 74.82) | 43.2 (21.86 to 73.55) | 3341 (1690 to 5711) | 2010 (1040 to 3513) | 13.67 (6.91 to 23.36) | 13.42 (6.94 to 23.45) |
| High-income North America | 24848 (12839 to 42512) | 30432 (15136 to 52967) | 59.33 (30.66 to 101.51) | 66.46 (33.05 to 115.67) | 7346 (3870 to 12631) | 9371 (4848 to 16357) | 18.44 (9.71 to 31.7) | 21.41 (11.08 to 37.37) |
| Western Europe | 21491 (11418 to 36059) | 21033 (10578 to 35424) | 42.65 (22.66 to 71.56) | 44.67 (22.47 to 75.24) | 7672 (4131 to 12937) | 7409 (3825 to 12624) | 16 (8.61 to 26.98) | 16.6 (8.57 to 28.29) |
| Australasia | 2915 (1522 to 5022) | 3667 (2007 to 5772) | 90.73 (47.37 to 156.33) | 94.55 (51.75 to 148.82) | 1033 (558 to 1723) | 1292 (682 to 2165) | 33.74 (18.23 to 56.28) | 35.27 (18.61 to 59.11) |
| Andean Latin America | 5282 (2650 to 9119) | 6957 (3507 to 11868) | 55.23 (27.71 to 95.35) | 56.96 (28.71 to 97.16) | 2329 (1192 to 4014) | 2899 (1505 to 4995) | 24.8 (12.7 to 42.74) | 25.3 (13.13 to 43.59) |
| Tropical Latin America | 19405 (9828 to 32431) | 18711 (9727 to 31754) | 55.57 (28.14 to 92.87) | 55.09 (28.64 to 93.48) | 9089 (4612 to 15805) | 8486 (4517 to 14274) | 26.47 (13.43 to 46.02) | 26.01 (13.85 to 43.76) |
| Central Latin America | 15360 (8080 to 26316) | 16922 (8899 to 28651) | 36.96 (19.44 to 63.32) | 39.09 (20.56 to 66.18) | 7204 (3713 to 12121) | 7785 (4070 to 13081) | 17.54 (9.04 to 29.52) | 18.54 (9.69 to 31.15) |
| Southern Latin America | 3733 (1898 to 6417) | 3984 (2035 to 6651) | 38.16 (19.4 to 65.59) | 40.07 (20.47 to 66.89) | 1378 (700 to 2346) | 1441 (753 to 2509) | 14.36 (7.29 to 24.45) | 15.06 (7.87 to 26.23) |
| Caribbean | 6157 (3255 to 10429) | 6496 (3361 to 11142) | 81.16 (42.91 to 137.47) | 83.77 (43.33 to 143.66) | 2374 (1217 to 4069) | 2432 (1241 to 4243) | 31.61 (16.2 to 54.16) | 32.4 (16.53 to 56.51) |
| Central Europe | 5719 (2880 to 9934) | 3447 (1753 to 5892) | 28.54 (14.37 to 49.58) | 28.41 (14.45 to 48.57) | 2360 (1185 to 4047) | 1396 (717 to 2381) | 12.27 (6.16 to 21.05) | 12.21 (6.28 to 20.84) |
| Eastern Europe | 9655 (5060 to 16814) | 7008 (3675 to 12185) | 28.18 (14.77 to 49.08) | 29.57 (15.51 to 51.42) | 3996 (1996 to 7073) | 2842 (1411 to 5045) | 12.1 (6.05 to 21.42) | 12.65 (6.28 to 22.46) |
| Central Asia | 4101 (2029 to 7030) | 4667 (2328 to 8104) | 25.57 (12.65 to 43.84) | 26.06 (13 to 45.24) | 1715 (895 to 3038) | 1878 (939 to 3332) | 11.03 (5.76 to 19.54) | 11.24 (5.62 to 19.94) |
| North Africa and Middle East | 30404 (15237 to 51834) | 39015 (19696 to 65745) | 33.56 (16.82 to 57.22) | 31.99 (16.15 to 53.91) | 11863 (6057 to 20108) | 14878 (7616 to 25402) | 13.76 (7.03 to 23.33) | 12.99 (6.65 to 22.18) |
| South Asia | 41299 (21451 to 69968) | 56432 (28152 to 94821) | 14.67 (7.62 to 24.86) | 15.88 (7.92 to 26.68) | 16239 (8309 to 28106) | 21843 (11019 to 38333) | 6.22 (3.18 to 10.77) | 6.66 (3.36 to 11.69) |
| Southeast Asia | 29633 (15116 to 50807) | 29963 (15075 to 51931) | 26.52 (13.53 to 45.47) | 25.43 (12.79 to 44.07) | 12036 (6284 to 20821) | 12108 (6252 to 21178) | 11.13 (5.81 to 19.25) | 10.87 (5.61 to 19) |
| East Asia | 130137 (67552 to 223241) | 120388 (63418 to 201863) | 54.47 (28.27 to 93.43) | 65.38 (34.44 to 109.63) | 45757 (23636 to 76949) | 39975 (21280 to 67435) | 20.68 (10.68 to 34.79) | 24.86 (13.23 to 41.93) |
| Oceania | 506 (259 to 874) | 959 (476 to 1667) | 28.86 (14.74 to 49.83) | 28.69 (14.22 to 49.85) | 192 (101 to 330) | 361 (188 to 619) | 11.92 (6.27 to 20.47) | 11.86 (6.17 to 20.35) |
| Western Sub-Saharan Africa | 7110 (3525 to 12235) | 19518 (9654 to 32851) | 13.41 (6.65 to 23.07) | 14.54 (7.19 to 24.47) | 2888 (1487 to 5075) | 7494 (3940 to 13172) | 5.3 (2.73 to 9.32) | 5.58 (2.93 to 9.81) |
| Eastern Sub-Saharan Africa | 7240 (3649 to 12319) | 15924 (8053 to 27104) | 12.93 (6.51 to 21.99) | 13.83 (6.99 to 23.54) | 2927 (1533 to 5301) | 6408 (3273 to 11379) | 5.33 (2.79 to 9.66) | 5.7 (2.91 to 10.12) |
| Central Sub-Saharan Africa | 1980 (996 to 3370) | 5083 (2538 to 8720) | 12.69 (6.38 to 21.6) | 13.68 (6.83 to 23.46) | 807 (390 to 1452) | 2053 (1069 to 3783) | 5.25 (2.54 to 9.44) | 5.64 (2.94 to 10.39) |
| Southern Sub-Saharan Africa | 1817 (905 to 3124) | 2268 (1146 to 3934) | 13.83 (6.89 to 23.77) | 14.39 (7.27 to 24.96) | 759 (391 to 1357) | 914 (471 to 1671) | 5.7 (2.94 to 10.19) | 5.9 (3.04 to 10.78) |

Abbreviations: DALYs, disability-adjusted life-years; UI, uncertainty interval.

**eFigure 1.** Flowchart demonstrating non-fatal estimation for ADHD.

**eFigure 2.** Burden of ADHD by sex and year, <20 years, 1990-2021. (A) Prevalence. (B) Incidence. (B) DALYs.

DALYs: disability-adjusted life-years.

**eFigure 3a.** Prevalence rate of ADHD in ages under 20 by sex and region, 2021.

**eFigure 3b.** Incidence rate of ADHD in ages under 20 by sex and region, 2021.

**eFigure 3c.** DALY rate of ADHD in ages under 20 by sex and region, 2021.

DALYs: disability-adjusted life-years.

**eFigure 4a.** Percentage change in prevalence rate of ADHD in ages under 20 by region, 1990-2021.

**eFigure 4b.** Percentage change in incidence rate of ADHD in ages under 20 by region, 1990-2021.

**eFigure 4c.** Percentage change in DALY rate of ADHD in ages under 20 by region, 1990-2021.

DALYs: disability-adjusted life-years.

**eFigure 5.** (A) Prevalence, (B) incidence, and (C) DALY rate in ages under 20 of ADHD by SDI group and year, 1990-2021.

DALYs:disability-adjusted life-years.
